# 15 years of longitudinal genetic, clinical, cognitive, imaging, and biochemical measures in DIAN

**DOI:** 10.1038/s44400-025-00047-7

**Published:** 2026-02-16

**Authors:** Alisha J. Daniels, Eric McDade, Jorge J. Llibre-Guerra, Chengjie Xiong, Richard J. Perrin, Laura Ibanez, Charlene Supnet-Bell, Carlos Cruchaga, Alison Goate, Alan E. Renton, Tammie L. S. Benzinger, Brian A. Gordon, Jason Hassenstab, Celeste Karch, Allan Levey, John C. Morris, Virginia Buckles, Ricardo F. Allegri, Patricio Chrem, Sarah B. Berman, Jasmeer P. Chhatwal, Martin R. Farlow, Nick C. Fox, Gregory S. Day, Takeshi Ikeuchi, Mathias Jucker, Johannes Levin, Jae-Hong Lee, David Aguillon, Leonel Takada, Ana Luisa Sosa, Ralph Martins, Hiroshi Mori, James M. Noble, Stephen Salloway, Edward Huey, Raquel Sánchez-Valle, Peter R. Schofield, Jee Hoon Roh, Randall J. Bateman, the Dominantly Inherited Alzheimer Network, Alisha J. Daniels, Eric McDade, Jorge J. Llibre-Guerra, Chengjie Xiong, Richard J. Perrin, Laura Ibanez, Charlene Supnet-Bell, Carlos Cruchaga, Alison Goate, Alan E. Renton, Brian A. Gordon, Jason Hassenstab, Celeste Karch, Allan Levey, John C. Morris, Virginia Buckles, Ricardo F. Allegri, Patricio Chrem, Sarah B. Berman, Jasmeer P. Chhatwal, Martin R. Farlow, Nick C. Fox, Gregory S. Day, Takeshi Ikeuchi, Mathias Jucker, Johannes Levin, Jae-Hong Lee, David Aguillon, Leonel Takada, Ana Luisa Sosa, Ralph Martins, Hiroshi Mori, James M. Noble, Stephen Salloway, Edward Huey, Raquel Sánchez-Valle, Peter R. Schofield, Jee Hoon Roh, Randall J. Bateman, Tammie L. S. Benzinger, David M. Holtzman, Anne M. Fagan, Erin Franklin, Xiong Xu, Ruijin Lu, Guoqiao Wang, Yan Li, Emily Gremminger, Laura Courtney, Gina Jerome, Elizabeth Herries, Jennifer Stauber, Bryce Baker, Matthew Minton, Danielle M. Picarello, Russ Hornbeck, Allison Chen, Charles Chen, Shaney Flores, Nelly Joseph-Mathurin, Steve Jarman, Kelley Jackson, Sarah Keefe, Deborah Koudelis, Parinaz Massoumzadeh, Austin McCullough, Nicole McKay, Joyce Nicklaus, Christine Pulizos, Qing Wang, Edita Sabaredzovic, Hunter Smith, Jalen Scott, Ashlee Simmons, Jacqueline Rizzo, Jennifer Sullivan, Sarah Stout, Andrew J. Aschenbrenner, Jacob Marsh, Nicolas Barthelemy, Jinbin Xu, Erik C. B. Johnson, Nicholas T. Seyfried, Ezequiel Surace, Silvia Vazquez, Snezana Ikonomovic, Neelesh K. Nadkarni, David M. Cash, Natalie S. Ryan, Neill R. Graff-Radford, Kensaku Kasuga, Christoph Laske, Anna Hofmann, Elke Kuder-Buletta, Susanne Graber-Sultan, Ulrike Obermueller, Yvonne Roedenbeck, Jonathan Voglein, Francisco Lopera, Yudy Milena, Laura Ramirez, William S. Brooks, Jacob A. Bechara, Yoshiki Niimi, Pedro Rosa-Neto, John Ringman, Colin Masters

**Affiliations:** 1 Washington University School of Medicine, St Louis, St Louis, MO USA; 2 Icahn School of Medicine at Mount Sinai, New York, NY USA; 3 Goizueta Alzheimer’s Disease Research Center, Emory University, Atlanta, GA USA; 4 Institute of Neurological Research FLENI, Buenos Aires, Argentina; 5 University of Pittsburgh, Pittsburgh, PA USA; 6 Massachusetts General and Brigham & Women’s Hospitals, Harvard Medical School, Boston, MA USA; 7 Indiana University School of Medicine, Indianapolis, IN USA; 8 UK Dementia Research Institute at University College London, London, UK; 9 University College London, London, UK; 10 Mayo Clinic in Florida, Jacksonville, FL USA; 11 Brain Research Institute, Niigata University, Niigata, Japan; 12 DZNE, German Center for Neurodegenerative Diseases, Tübingen, Germany; 13 DZNE, German Center for Neurodegenerative Diseases, Munich, Germany; 14 Ludwig-Maximilians-Universität München, Munich, Germany; 15 Asan Medical Center, Seoul, South Korea; 16 Universidad de Antioquia, Medellin, Colombia; 17 Fundacao Faculdade de Medicina, Sao Paulo, Brazil; 18 Instituto Nacional de Neurologia y Neurocirugla Innn, Mexico City, Mexico; 19 Edith Cowan University, Joondalup, WA Australia; 20 Osaka City University, Osaka, Japan; 21 Taub Institute for Research on Alzheimer’s Disease and the Aging Brain, Department of Neurology, and GH Sergievsky Center, Columbia University Irving Medical Center, New York, NY USA; 22 Brown University, Butler Hospital, Providence, RI USA; 23 Hospital Clínic de Barcelona. IDIBAPS. University of Barcelona, Barcelona, Spain; 24 Discipline of Psychiatry and Mental Health, University of New South Wales, Sydney, NSW Australia; 25 Korea University, Korea University Anam Hospital, Seoul, South Korea; 26 Unit for Early and Exploratory Clinical Development, The University of Tokyo, Tokyo, Japan; 27 Translational Neuroimaging Laboratory, McGill University Research Centre for Studies in Aging, Montreal, QC Canada; 28 Department of Neurology, Keck School of Medicine of USC, University of Southern California, Los Angeles, CA USA; 29 Florey Institute, The University of Melbourne, Melbourne, VIC Australia

**Keywords:** Genetic markers, Alzheimer's disease, Diagnostic markers, Predictive markers, Prognostic markers, Cognitive neuroscience, Alzheimer's disease

## Abstract

The Dominantly Inherited Alzheimer Network Observational Study (DIAN Obs) is a longitudinal, global cohort study investigating brain aging and autosomal dominant Alzheimer’s disease (ADAD), a rare monogenic form of Alzheimer’s disease (AD). Established in 2008 with support from the National Institute on Aging (NIA), DIAN Obs is designed to collect comprehensive and uniform data with the aim to characterize brain biology and clinical trajectory of individuals at risk for ADAD. Mutations in the amyloid protein precursor (*APP*), presenilin 1 (*PSEN1*), or presenilin 2 (*PSEN2*) genes cause ADAD with virtually full penetrance and a predictable age at symptomatic onset. Participants, both mutation carriers and non-carriers from affected families, undergo longitudinal clinical and cognitive assessments, neurologic and physical examinations, structural and functional neuro-imaging, and amyloid and tau positron emission tomography (PET). Biospecimens include cerebrospinal fluid, plasma, serum, and whole blood for biochemical, genetic and multi-omic analyses, with brain donation upon death. This dataset enables one of the most detailed longitudinal examinations of the human brain across the continuum from presymptomatic to symptomatic AD. The extensive DIAN Obs data and biospecimen repository provides a globally accessible resource to advance understanding of AD pathophysiology, aging, and the development of preventive and therapeutic interventions.

## Introduction

The vast majority of AD dementia is sporadic and generally occurs in older ages, but a small proportion (less than 1%) of AD dementia is caused by mutations in the Aβ precursor protein (*APP*), presenilin 1 (*PSEN1*), or presenilin 2 (*PSEN2*) genes with almost 100% penetrance, generally at predictable younger ages. This form of AD is known as autosomal dominant AD (ADAD). ADAD is thought to have an underlying pathogenic process similar to that of the more common sporadic AD and thus may hold the key to understanding the pathogenesis of AD and identification of effective treatments for both ADAD and sporadic AD. To leverage this population's potential for AD research, the DIAN Obs was established in 2008. It aims to track individuals from families with known ADAD mutations, employing a wide array of cognitive assessments and biomarker tests. Since its inception time, the DIAN Obs has amassed a substantial repository of clinical and biomarker data and samples, facilitating a deeper understanding of ADAD's natural history.

**Fig. 1:**
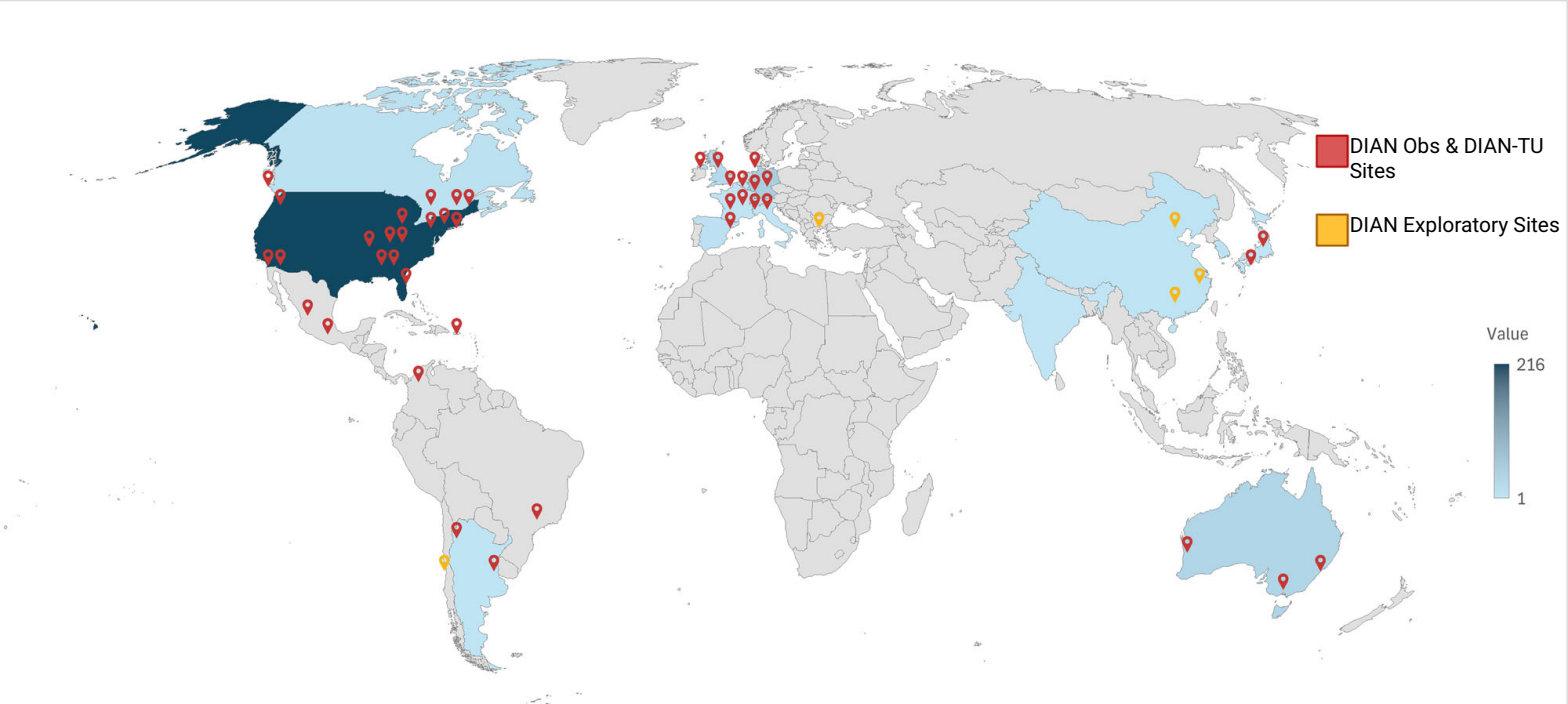
DIAN Obs/DIAN-TU Sites and Resource Request Distribution by Country. DIAN Obs, or DIAN Observational Study and DIAN-TU, or the DIAN Trials Unit, global site locations are represented by the red icons. DIAN EXR, or DIAN Expanded Registry, exploratory areas for potential new sites for DIAN Obs and DIAN-TU are represented by the yellow icons. For the Value bar, data and tissue requests fulfilled is represented by the shading of each country; United States = 216 (darkest shade) and countries in gray reflect zero requests.

The DIAN Obs cohort has been longitudinally followed for over 15 years and is amongst the most deeply phenotyped cohorts of brain aging, function, and AD measures in predominantly 18 to 55-year-old people. The inaugural year of DIAN Obs included ten performance sites in three countries (US, UK, and Australia) with English as the language for all initial sites. The continued success of DIAN Obs has since grown to twenty-three performance sites in eleven countries and supporting seven languages: English, Spanish, German, Japanese, Korean, French, and Portuguese (Fig. 1 and Supplementary Table 1). A total of 664 participants have enrolled in DIAN Obs; currently the study has 314 active participants. Through these participant provided resources, DIAN Obs has fulfilled more than 300 data and tissue requests globally to investigators with hypotheses and aims outside of DIAN Obs (Fig. 1).

DIAN Obs is the key scientific and discovery study for ADAD and also provides natural history information for the DIAN-Trials Unit (DIAN-TU), which is the therapeutic and target validation platform for treatment and prevention trials. The DIAN-TU [dian.wustl.edu/our-research/clinical-trial/]^1^ is a global research effort established in 2012 to design and conduct clinical trials for the prevention or treatment of ADAD^1^. Data from DIAN Obs and DIAN-TU studies were designed to be conducted together, with nearly identical protocols, including cognitive and clinical assessments, biomarker measures, and quality control.

Participant outreach, recruitment, and retention for both DIAN Obs and DIAN-TU are facilitated via the international DIAN Expanded Registry (DIAN EXR) [dian.wustl.edu/our-research/registry], established in 2012 for individuals who are or may be affected by ADAD. DIAN EXR serves as a collaborative research effort not only to facilitate study referral to DIAN Obs and DIAN-TU, but also to support educational and outreach activities with ADAD family members.

**Fig. 2:**
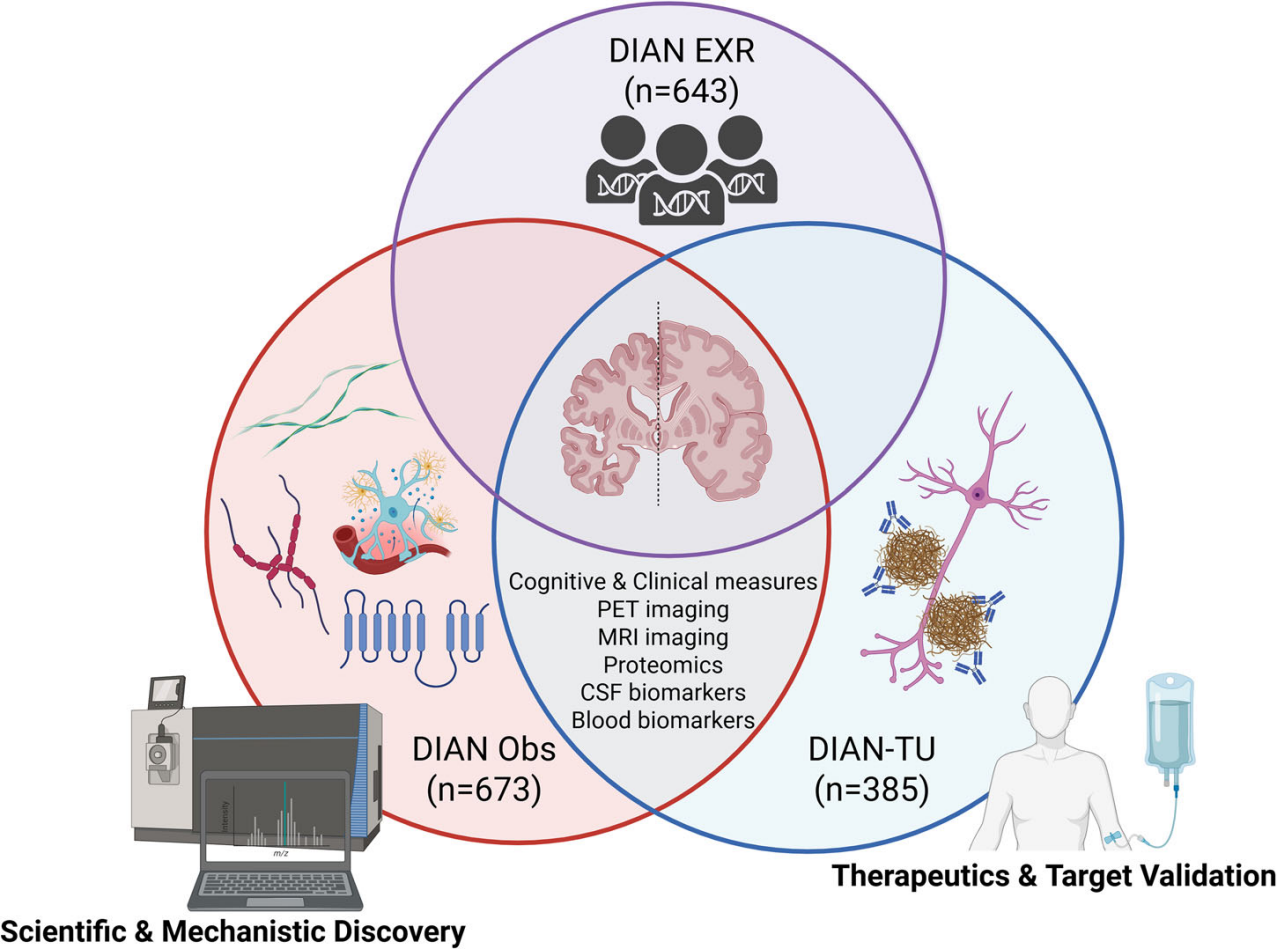
Coordination of DIAN Obs, DIAN-TU, and DIAN EXR. DIAN Obs, or DIAN Observational Study, provides natural history data collection for scientific studies with currently 673 total enrolled participants. DIAN-TU, or DIAN Trials Unit, serves as the therapeutic sector for prevention and treatment clinical trials with 385 total enrolled participants. DIAN EXR, or DIAN Expanded Registry, provides support for education and outreach for participants and family members with 643 registered individuals. These three distinct groups within the network collaborate and support one another with the goal to advance Alzheimer’s disease research and treatment options to the medical community. PET positron emission tomography, MRI magnetic resonance imaging, CSF cerebrospinal fluid.

DIAN Obs and DIAN-TU have distinct purposes with differing eligibility requirements and site locations allowing the DIAN EXR to act as a central mechanism for navigation and referral of interested registrants to potential research opportunities (Fig. 2).

The main scientific hypotheses of DIAN Obs aim to address fundamental questions regarding the pathophysiology of AD. First, longitudinal changes in AD biomarkers will distinguish mutation carriers (MC) many years before the onset of clinical symptoms, supporting the concept of a measurable preclinical phase of AD. Second, the initial biomarker changes in the preclinical stage of ADAD will involve Aβ42, followed sequentially by biomarkers of neurodegeneration and, ultimately, by detectable cognitive decline. Third, the clinical, biomarker, and neuropathological phenotypes of ADAD will parallel those observed in “sporadic” late onset AD (LOAD). DIAN Obs emphasizes longitudinal data as it provides more accurate and precise information on the magnitude and rate of change for biofluid and imaging biomarkers throughout the preclinical stage of ADAD.

In ADAD, the age of symptomatic onset is tightly linked to the specific mutation and family history, allowing researchers to derive an estimated years of onset (EYO) for individual carriers. This permits the use of EYO as a temporal anchor: negative values reflect years remaining before expected symptom onset, zero corresponds to predicted conversion, and positive values indicate years since onset^2^. By aligning participants along this EYO axis, it becomes possible to map biomarker and cognitive trajectories relative to the expected onset point even before clinical symptoms appear.

Additionally, in the DIAN Obs cohort, individuals who do not carry the familial Alzheimer’s mutation provide a rare and valuable control group access early to middle adulthood. Because these non-carriers (NC) are enrolled alongside mutation carriers using identical protocols and longitudinal follow-ups, they offer exceptionally well-matched healthy baselines. This design allows the data and tissue resources generated from the DIAN Obs protocol to contribute not only to understanding ADAD, but also the potential to broader studies of normative aging trajectories and biomarker baselines in younger adults.

**Fig. 3:**
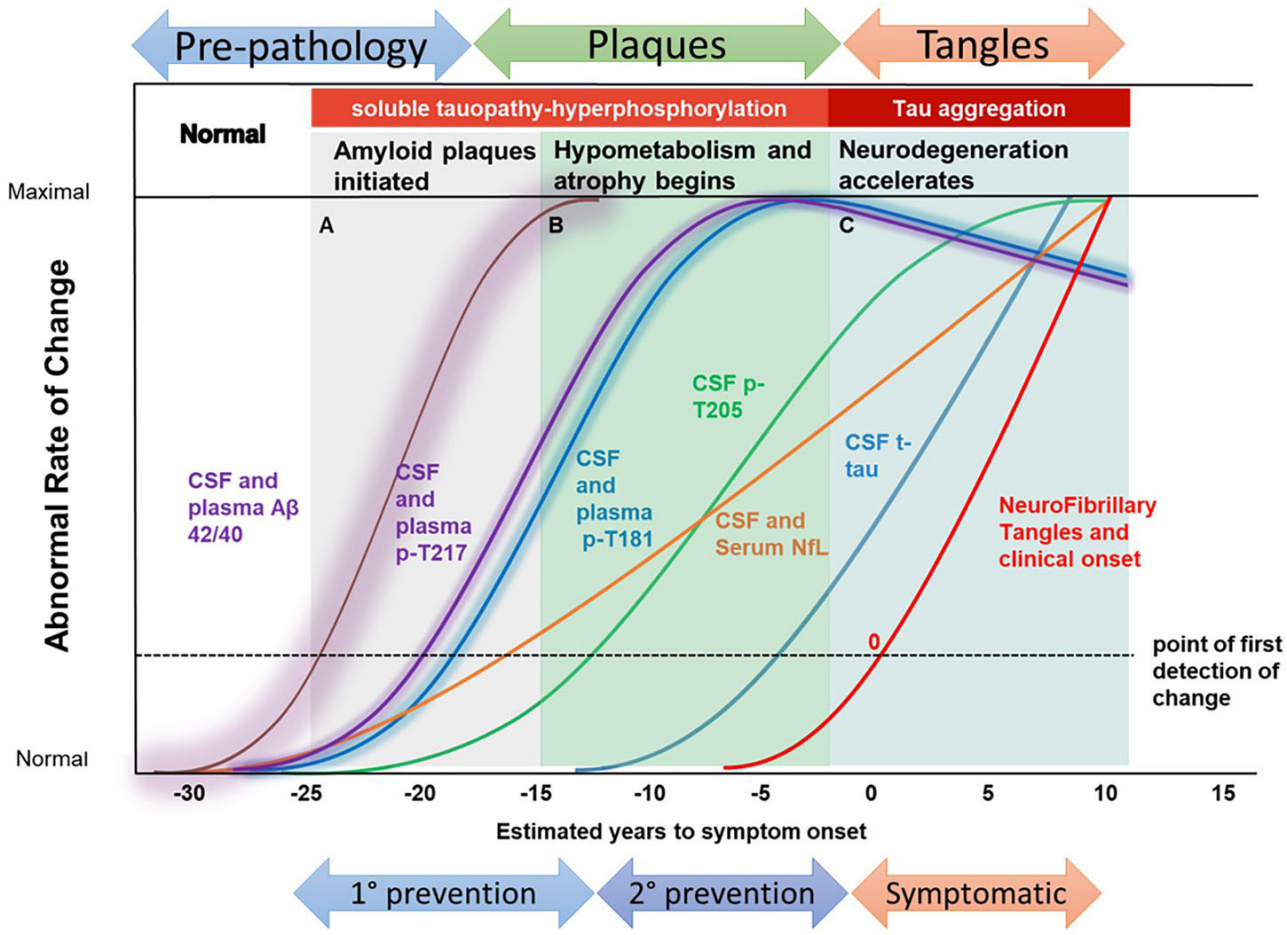
ADAD Onset & Progression compared to Sporadic AD *Adapted from Barthelemy et al., Nat. Med. 2020. The evolution of amyloid and tau pathophysiology is observed through distinct phases in CSF and plasma providing comparison from the DIAN cohort to onset and progression of sporadic Alzheimer’s disease (AD). Measures of different amyloid (Aβ 42/40) and tau (p-T217, p-T181, p-T205) species and aggregate tau demonstrate over 35 years sequential change by stage of disease related to plaque formation, cortical atrophy and metabolism. A Amyloid plaques intiated (brown line). B p-T217 (purple line), p-T181 (dark blue line) and NfL (orange line) levels begin to increase. C With the decrease of cortical metabolism, p-T205 (green line) and soluble t-tau (light blue line) begin to increase and tau PET tangles (red line) begin to develop while p-T217 and p-T181 decrease. CSF cerebrospinal fluid, Aβ amyloid beta, p- phosphorylated, NfL neurofilament light chain, t- total.

DIAN Obs has provided seminal advances in the understanding of brain health, the onset and progression of ADAD, and how this compares to the more common sporadic AD (Fig. 3). In 2012, DIAN Obs described a comprehensive order of clinical, cognitive, imaging, and biomarker changes that occur across a time span two decades before and a decade after symptom onset. Amyloid plaques continuously accumulate for 15–20 years before symptom onset, defining an amyloid growth phase^2–5^, while tau tangles appear and accumulate in the transition to and during the symptomatic phase^6^. A series of biochemical changes in CSF and blood begin with amyloid-beta 42/40 decreasing, followed closely by p-tau217/181/231 associated with amyloid plaques, then p-tau205, neurofilament light chain (NfL), and total tau increasing before the appearance of tangles. Cortical hypometabolism begins up to 18 years prior to symptom onset, and cortical atrophy up to 13 years prior to symptom onset^3,4,7,8^. Finally, changes in cognitive performance are detected several years before onset.

DIAN-TU implements therapeutic trials in a trial platform with the goal to slow, delay, or prevent dementia in the ADAD population^1,9^. DIAN Obs and DIAN-TU implement harmonization efforts to ensure the combination and comparability of DIAN-TU and DIAN Obs protocols, including International Council for Harmonization Good Clinical Practice (ICH GCP) guideline compliance. These harmonization efforts increase longitudinal data and sample resources that can be combined across DIAN Obs and DIAN-TU. The Biostatistics Core enables the ability to link coded participant data across the DIAN Obs and DIAN-TU, enabling combining and comparing across the studies. Key measures harmonized include electronic capture of clinical and cognitive assessments, sample collection protocols, and imaging (MRI and PET).

## Results

### Study description

With over 15 years of follow-up, the DIAN has enrolled more than 600 participants from families carrying mutations in *APP*, *PSEN1*, or *PSEN2*, including both mutation carriers and non-carriers. Longitudinal assessments across clinical, cognitive, imaging, and fluid biomarker domains revealed a consistent temporal sequence of Alzheimer’s disease (AD) pathophysiological changes. Amyloid accumulation on PET and reductions in CSF Aβ42 were observed up to two decades before estimated symptom onset, followed by increases in CSF total-tau and phosphorylated-tau, cortical thinning, and metabolic decline on PET approximately 10–15 years prior to expected onset. Cognitive decline, as measured by global and domain-specific composites, emerged roughly 5–10 years before symptomatic conversion, paralleling clinical diagnostic progression from asymptomatic to prodromal and dementia stages. Collectively, these findings provide a comprehensive 15-year longitudinal framework describing the natural history of dominantly inherited AD and establish the foundation for prevention and interventional trials targeting the earliest stages of disease.

## Discussion

Washington University in St. Louis is the recipient of a U19 grant from the NIA and serves as the DIAN Obs Coordinating Center, which oversees both the scientific and administrative center and serves as a performance site. The DIAN Obs Coordinating Center consists of eight Cores: Administration, Clinical, Genetics, Cognition, Imaging, Biomarker, Neuropathology, and Biostatistics and three scientific Projects: Amyloid-β, Tau, and Novel Mechanisms. All performance sites have access to adequate numbers of potential DIAN Obs participants and the resources and capabilities to conduct all elements of the DIAN Obs protocol.

**Fig. 4:**
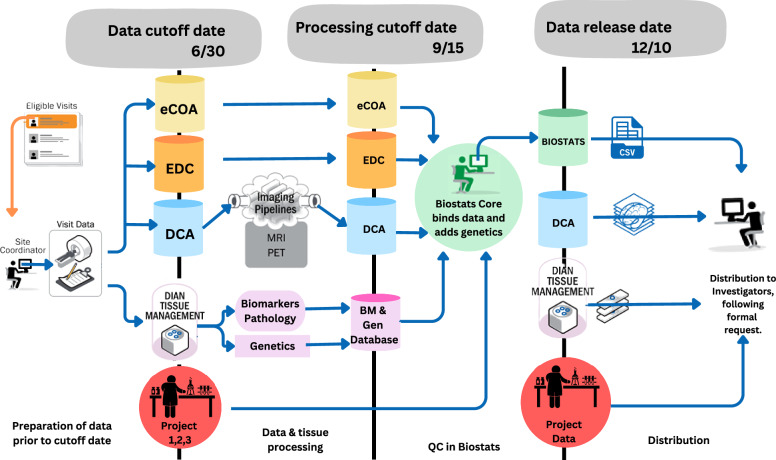
DIAN Obs Data Freeze Process. eCOA electronic Clinical Outcome Assessment, EDC electronic data capture, DCA DIAN Central Archive, MRI magnetic resonance imaging, PET positron emission tomography, BM Biomarker, Gen Genetics, QC quality control.

The human data outlined in this manuscript are stored in the DIAN Repository managed by the Division of Biomedical Statistics and Informatics at Washington University in St. Louis. Data freezes are completed annually, synchronizing the study’s efforts to collect, process, and review data for distribution. The amount of data in this natural history study does not currently have a cap as it is collected upon study start and longitudinally every other year for asymptomatic participants and annually for symptomatic participants, with no data collection gap and no study end planned. Each data freeze produces a blinded cumulative snapshot of all available vetted data across all study modalities and consists of the following stages: (1) data cut-off, (2) processing cut-off, (3) quality control of raw and derived data, (4) outlier quality control, and (5) dataset preparation and blinding (see Fig. 4). With the NIH Data Management and Sharing Policy implemented in January 2023, the study’s next grant cycle will transition the human data to the Becker Medical Library’s Digital Commons Data Repository and Research Infrastructure.

The study’s primary human data is highly sensitive and will be protected. Access will require a data request, followed by review and approval before access is granted. Investigators who wish to submit a data request may review the types of data that are available and complete a data request form on the DIAN website (https://dian.wustl.edu/). Each request is received by the study data sharing committee, which will review and upon approval, a Data Use Agreement (DUA) is required to be in place prior to data release. Data file types and formats include: .tiff, .lif, .fastq, .tsv, .mzML, .mtx, .csv, .rsd., and .dcm. Raw data files will be analyzed to generate .csv or .tsv files for statistical analysis.

## Methods

This observational, natural history study is managed through the DIAN Obs Coordinating Center based at Washington University in St. Louis and is conducted at sites globally with all sites requiring a current Institutional Review Board (IRB)/Independent Ethics Committee (IEC) approval to conduct study visits in accordance with the DIAN Obs protocol. Participants review and discuss the consent form with the performance site study team prior to being asked to sign the informed consent for study participation. A copy of the consent is provided to participants, and the original is maintained in the participant’s research record. For this multi-center study, the local IRB/IEC committees are: Advarra (single IRB for U.S based sites and Coordinating Center)/IRB reference #: Pro00065069, Comité de Bioética de la Facultad de Medicina, Comite de Etica en Ivestigacion en Investigacion en Salud de UCASAL, Comite de Etica en Ivestigacion INNN MSC, Eberhard Karls Universitat/ Univerity Hosptials Tubingen, Ethikkomission bei der Ludwig-Maximillians Unversitat Munchen, FLENI Comite de etica en Investigaciones biomedicas, McGill Institutional Review Board, NHS Health Research Authority; Berkshire REC Centre, Niigata University, Ramsay Health Care WA SA HREC, Seoul Asan Medical Center IRB, South Eastern Sydney Local Health District HREC, and The University of Tokyo Research Ethics Committee.

### Administration core

The DIAN Obs Administration Core provides oversight and management of the DIAN Obs project, including: coordinating activities of the other Cores, scientific research Projects, and subcontractors; managing and supporting the DIAN Obs Steering Committee; seeking and facilitating feedback from the External Advisory Committee; interacting with the NIA liaisons; and managing activation, maintenance, and data collection of all performance sites.

**Fig. 5:**
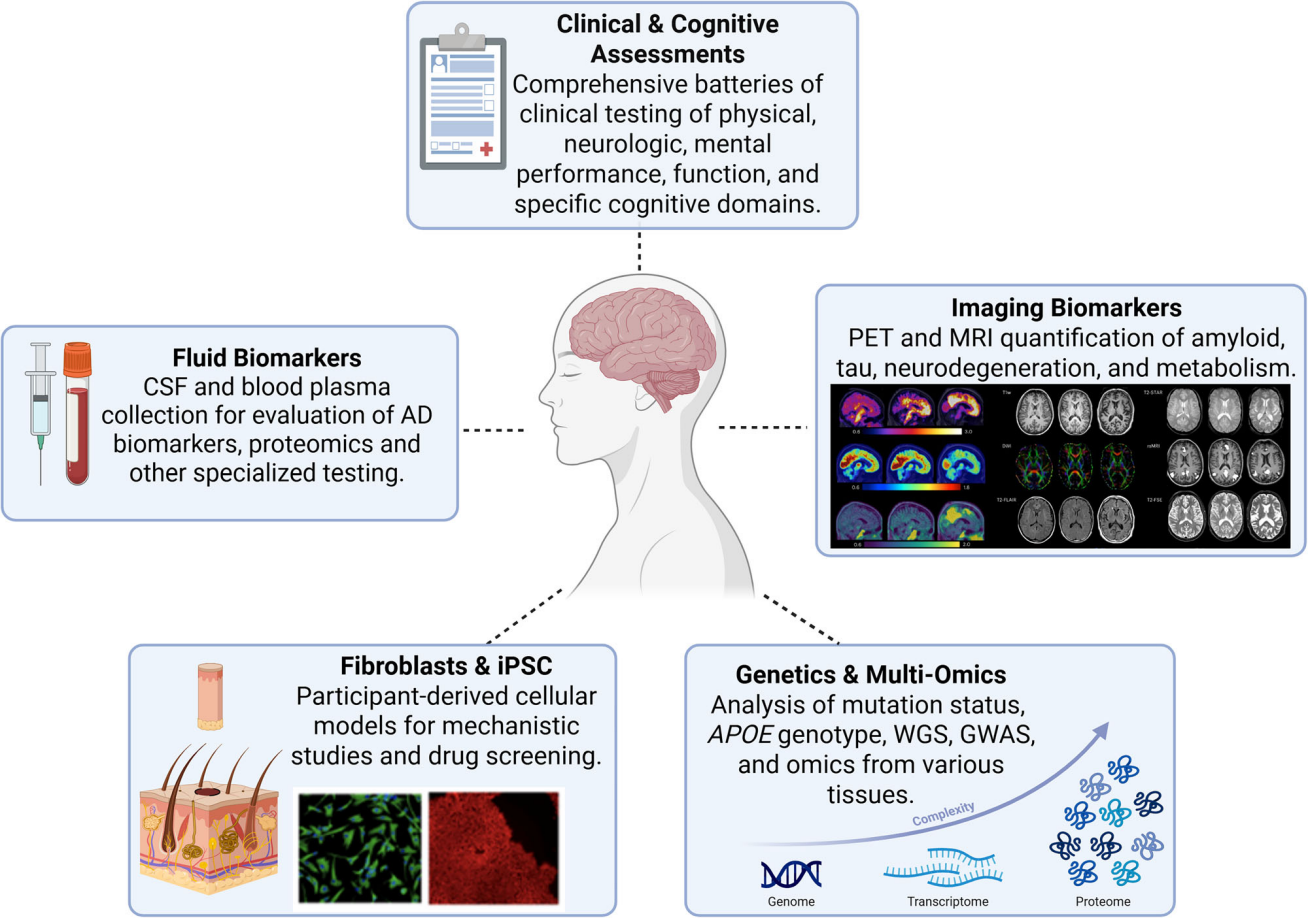
Overview of DIAN Obs Resource Requests. A summary of DIAN Obs, or DIAN Observational Study, data types available to researchers. PET positron emission tomography, MRI magentic resonance imaging, CSF cerebrospinal fluid, AD Alzheimer’s disease, iPSC induced pluripotent stem cells, WGS whole genome sequencing, GWAS genome-wide association study.

The Administration Core has established a web-based system to support data and tissue resource dissemination to investigators. The DIAN Obs data and biospecimen application form is available on the DIAN website (dian.wustl.edu). All requests are reviewed by relevant Core Leaders, the Study Director, and DIAN Obs Steering Committee. Upon receiving request approval, with appropriate institutional review board (IRB)/institutional ethics committee (IEC) approvals and data and tissue sharing agreements, data and/or biospecimens are shared with support from the related Cores and Biostatistics Core. The DIAN Obs Data and Tissue Sharing, Notifications, Publications, and Authorship Policies govern the sharing of DIAN Obs resources and guidelines for publications^10^. As of December 2022, DIAN Obs has received 288 data requests and 114 tissue requests, with 231 and 85 requests fulfilled, respectively. An overview of resources that may be requested is provided in Fig. 5.

### Clinical core

The Clinical Core oversees clinical protocol execution and associated activities, encompassing participant recruitment, retention, clinical evaluations, CSF and blood sample acquisition, safety protocols, and quality control initiatives. Enrollment eligibility criteria include being 18 years of age or older and having a known pathogenic ADAD mutation in the family with a risk of inheriting the mutation. All DIAN Obs participants have access to genetic counseling and testing.

Upon participant consent, a visit anniversary date is established, initiating the DIAN Obs visit schedule with a frequency of one to three years. In-person site visits commence with the initial assessment and subsequently alternate with remote assessments for asymptomatic participants. Symptomatic participants undergo annual in-person evaluations under the current protocol. Table 1 details clinical and cognitive data collection procedures and data availability. A comprehensive list of current and former DIAN Obs performance sites is included as Supplementary Table 1.

**Table 1:** Clinical core available resources & data

Data:	Number of participants	Total assessment count	Number of participants with only 1 assessment	Number of participants with > 1 assessment	Mean assessments among participants with > 1 assessment	Distribution of assessment count^b^
Estimated Parental Age at Onset	671	1653	227	444	3.21	
Demographics	673	1431	288	385	2.97	
Health history	673	1659	229	444	3.22	
Family history	673	673	673	NA	NA	NA
Medications	605	NA	NA	NA	NA	NA
Physical & Neurologic exam findings	673	1647	231	442	3.20	
Clinician Judgment of Symptoms	673	1659	229	444	3.22	
Clinician Diagnosis, including Cognitive Status & Dementia	673	1659	229	444	3.22	
Informant Demographics	673	1432	287	386	2.97	
Exercise Questionnaire	673	1659	229	444	3.22	
Hollingshead Index of Social Position	673	1145	376	297	2.59	
Clinical Dementia Rating (CDR)	673	1659	229	444	3.22	
Geriatric Depression Scale (GDS)	672	1653	230	442	3.22	
Functional Assessment Questionnaire (FAQ)	670	1648	230	440	3.22	
Mini-mental Status Exam (MMSE)	671	1621	239	432	3.20	
Digit Span	673	1658	230	443	3.22	
Category fluency for animals & vegetable	673	1658	230	443	3.22	
Neuropsychiatric Inventory-Q (NPI-Q)	672	1652	231	441	3.22	
United Parkinson’s Disease Rating Scale (UPDRS)-Motor	673	1640	233	440	3.20	
Hachinski Ischemic Score & Cerebrovascular Risk Factors	673	1639	234	439	3.20	

*NA* Not applicable

^a^DIAN Obs Data Freeze 17 (DF17) was used to compute these numbers. There are 673 participants in DF17. The cutoff for DF17 was 6/30/2023.

^b^The leftmost bar represents participants with exactly 1 assessment, 2nd leftmost bar represents participants with exactly 2 assessments, etc.

### Genetics core

The goals of the Genetics Core are to obtain and bank tissue for genetic and multi-omic studies, as well as to generate, process, and/or harmonize genetic and multi-omic data. At each DIAN Obs in-person initial visit, whole blood is collected and used for ADAD mutation status, *APOE* genotype, genome-wide association study (GWAS) genotyping array, and whole genome sequencing (WGS). At each DIAN Obs in-person initial and follow-up visit, buffy coat blood and PAXgene blood tubes are collected for longitudinal assessment of DNA methylomics and RNA transcriptomics, respectively. Additionally, the Core obtains and banks dermal fibroblasts and then generates induced pluripotent stem cells (iPSCs) (Table 2). These resources support DIAN Obs Projects and are available to the research community with an approved data and/or tissue request.

**Table 2:** DIAN Obs mutation status distribution

*PSEN1* (*N* = 436, 89 unique variants)	
mutation	272
no mutation	164
***PSEN2*** **(*N*** = **50, 8 unique variants)**	
mutation	28
no mutation	22
***APP*** **(*N*** = **120, 14 unique variants)**	
mutation	68
no mutation	52

The Genetics Core provides central determination and confirmation of gene sequence, whether normal or disease-causing mutation carrier, and *APOE* genotype on each of the 673 total study participants derived from 251 families. Table 3 presents the distribution of genetic variants and carrier status, categorized by gene. The Core, in coordination with the Clinical Core and DIAN EXR, maintains and curates a list of pathogenic mutations as well as confirms that new DIAN families carry an ADAD mutation.

**Table 3:** Genetics core available resources & data

Data:
ADAD Mutation Status
*APOE* Genotype
Genome-wide association Study (GWAS) genotyping array
Whole Genome Sequencing (WGS)
Polygenic Risk Scores for:
○ AD (risk, onset, progression),
○ Parkinson’s disease (risk)
○ Frontotemporal dementia (risk)
Multi-omics:
○ DNA methylomics: brain & blood
○ RNA transcriptomics: brain & blood
○ Proteomics: brain, CSF, & plasma
○ Metabolomics & lipidomics: brain, CSF, & plasma
**Tissue & Cell Lines:**
DNA/Cell lines
Dermal Fibroblasts
Induced Pluripotent Stem Cells (iPSCs)

The Genetics Core generates GWAS genotyping arrays, WGS, and *APOE* genotype data for all individuals. This data has been leveraged to generate polygenic risk scores (PRSs) for LOAD risk, onset, and progression, as well as Parkinson’s disease risk. Recent studies indicate that in DIAN Obs ADAD, LOAD risk PRS is not significantly associated with mutations status, but is associated with levels of CSF Aβ, total-tau, and p-tau, suggesting that known AD risk variants may modify age at onset (AAO) in the ADAD population. Polygenic risk score of sporadic late-onset Alzheimer's disease reveals a shared architecture with the familial and early-onset forms^11^.

Finally, the Genetics Core serves as a central repository for increasingly rich multi-omic data^12,13^. Currently, this catalog includes: DNA methylomics (Illumina MethylationEPIC 850k array) for brain (44 participants) and buffy coat blood (790 longitudinal samples from 266 participants); RNA-seq for brain (44 participants) and blood (575 longitudinal samples from 319 participants); and proteomics (SomaLogic 7k) and metabolomics/lipidomics (Metabolon HD4) for brain (44 participants), CSF (495 participants), and plasma (495 participants). These data have been used to identify circular RNAs in brain associated with AD and AD pathology^14,15^, as well as to identify proteins associated with carrying an ADAD mutation and change between 20 to -30 years before the onset, some of them even before some of the validated biomarkers.

### Cognition core

A primary goal of the Core is to maintain the cognitive assessment battery to align with scientific aims and to incorporate novel measures and novel assessment methodologies that are more sensitive to early cognitive changes in ADAD. The Cognition Core serves the overall grant by overseeing rater training and maintaining rigorous quality control (QC) and documentation standards that ensure the fidelity of longitudinal cognitive assessments. In addition, the Cognition Core plays a pivotal role in maintaining the consistency of cognitive assessments across various languages, ensuring culturally relevant translations and adaptations across different sites and countries. These methodologies will improve the reliability in the measurement of the key features of ADAD. The assessment of cognition is central for achieving the scientific aims of all DIAN Obs Projects and Cores. The Cognition Core works with the Project and Core leaders to ensure that fully validated cognitive data is available for DIAN Obs data freezes and provides guidance on appropriate cognitive measures and data analyses to support Project and Core aims. Refer to Table 4 for Core data availability. Novel methods implemented in the Cognition Core include the use of remote cognitive testing via Ambulatory Research in Cognition (ARC), and the development of novel remote cognitive tasks, including tests of long-term forgetting and statistical learning paradigms.

**Table 4:** Cognition Core Available Resources & Data

Data:	Number of participants	Total assessment count	Number of participants with only 1 assessment	Number of participants with > 1 assessment	Mean assessments among participants with > 1 assessment	Distribution of assessment count^b^
Trailmaking A & B	673	1648	232	441	3.21	
Wechsler Adult Intelligence Scale-Revised (Logical Memory)	673	1655	232	441	3.22	
Word list recall (immediate & delayed) designed specifically for DIAN	572	1275	198	374	2.88	
Letter Fluency for F-A-S	572	1275	198	374	2.88	
Boston Naming test^c^	587	1365	196	391	2.99	
International Personality Item Pool (IPIP)^d^	393	820	185	208	3.05	

NA Not Applicable

^a^DIAN Obs Data Freeze 17 (DF17) was used to compute these numbers. There are 673 participants in DF17. The cutoff for DF17 was 6/30/2023.

^b^The leftmost bar represents participants with exactly 1 assessment, 2nd leftmost bar represents participants with exactly 2 assessments, etc.

^c^The last Boston Naming test was collected on 10/6/2020.

^d^The last IPIP was collected on 10/25/2021.

### Imaging core

The Imaging Core is responsible for the acquisition, QC, processing, and analysis of the MRI and PET neuroimaging data for DIAN Obs. The imaging data set collected in DIAN Obs participants to date represents a highly valuable resource for AD research. It has supported cross-sectional analysis of PET and MRI data to develop a timeline for imaging biomarkers in ADAD. Carriers of AD-causing mutations and their non-carrier (NC) siblings are enrolled and followed in the Clinical Core through the international DIAN Obs performance sites. Participants undergo structural and functional MRI, amyloid PET, tau PET, and metabolic PET imaging in conjunction with their clinical visits. The Core obtains and analyzes longitudinal imaging data that is fully integrated with clinical, psychometric, and CSF biomarkers, and allows for mutation-specific genotype-phenotype analysis. MRI data are processed using the Freesurfer Imaging suite to derive regions of interest. These regions are then used to process the PET data. Imaging Core data formats available are outlined in Table 5. A neuroimaging-specific resource paper detailing in-depth imaging protocols has recently been published^5,16^.

**Table 5:** Imaging core available resources & data

Data:	Number of participants	Total assessment count	Number of participants with only 1 assessment	Number of participants with > 1 assessment	Mean assessments among participants with > 1 assessment	Distribution of assessment count^b^
MRI scans	643	1541	226	417	3.15	
MRI quantitative measurements^c^	593	1409	201	392	3.08	
AV-1451 Tau PET scans and quantitative measurements^d^	67	99	44	23	2.39	
MK-6240 Tau PET scans and quantitative measurements	31	37	26	5	2.20	
PiB PET scans	600	1318	234	366	2.96	
PiB PET quantitative measurements	549	1169	220	329	2.88	
FDG PET scans	577	1221	233	344	2.87	
FDG PET quantitative measurements	540	1117	223	317	2.82	

^a^DIAN Obs Data Freeze 17 (DF17) was used to compute these numbers. There are 673 participants in DF17. The cutoff for DF17 was 6/30/2023.

^b^The leftmost bar represents participants with exactly 1 assessment, 2nd leftmost bar represents participants with exactly 2 assessments, etc.

^c^Quantitative measurements from MRI scans include the volume and thickness of various brain regions.

^d^Quantitative measurements from PET scans include the concentration of amyloid-beta plaques measured by PiB PET scans, tau tangles measured by Tau PET scans, and metabolic activity assessed by FDG PET scans within various brain regions.

For tau PET, sites obtain scans with two tracers [18F]-MK-6240 or [18F]-AV-1451 (aka Flortaucipir [FTP], T807). Because there is no single tracer with the international distribution to reach all DIAN sites, each site has the ability to include one or both of the two tau PET tracers. While the use of multiple tracers maximizes the number of sites that can perform tau PET imaging, it is recognized that the limitations of using multiple tracers in the same study. The Imaging Core has performed preliminary analyses directly comparing head-to-head FTP and MK-6240 data collected from ADAD participants. Prior work has demonstrated that the tau PET signal only goes up with the onset of impairment^3,17^. The regional correlations seen between tracers are low in non-carriers and asymptomatic mutation carriers, and in individuals who are symptomatic, the correlations between tracers are very high, particularly in areas known to have high deposition. Images released through data requests will be defaced using the Mayo Imaging Pipeline (see Fig. 4).

### Biomarker core

The DIAN Obs Biomarker Core is a high-capacity biorepository enabling high-throughput processing while maintaining high-quality, gold-standard biomarker measurements of cerebrospinal fluid (CSF) and plasma samples available to investigators upon completion and approval through the DIAN tissue request process. The Biomarker Core obtains measures of the following biomarker analytes using the Lumipulse automated assay platform: CSF (Aβ1-40, Aβ1-42, total tau [t-tau], p-tau 181). Data and sample availability is outlined in Table 6. Core samples may be leveraged in a longitudinal manner, in conjunction with extensive clinical and biological data, to study both traditional and exploratory biomarkers. With the main priority of the Biomarker Core to evaluate fluid biomarker profiles in ADAD participants comparing MCs to NC, DIAN Obs, along with others, have helped give insight to expected biomarker trajectories given the availability of expected AAO in MC individuals^3,11^. The Biomarker Core has demonstrated that fluid biomarkers changes begin during the preclinical period (20-30 years before expected symptom onset). However, collection and analysis of additional longitudinal samples are required to define the patterns of change of known and novel fluid biomarkers that happen right when an individual progresses from asymptomatic (or preclinical) to symptomatic. Given the minimally invasive nature of phlebotomy, the field is invested in the identification of plasma biomarkers. In response to the needs in the field, DIAN Obs’ scientific Projects will measure and analyze established markers of amyloid (Aβ and %p-tau217 ratio) and tau (MTBR243) deposition, inflammation (GFAP, sTREM2), and neurodegeneration (NfL) in CSF and plasma.

**Table 6:** Biomarker core available resources & data

Data:	Number of participants	Total assessment count	Number of participants with only 1 assessment	Number of participants with > 1 assessment	Mean assessments among participants with > 1 assessment	Distribution of assessment count^b^
CSF	596	1260	249	347	2.91	
Plasma	665	1620	231	434	3.20	
INNOTEST CSF Aβ40	462	843	224	238	2.60	
INNOTEST CSF Aβ42	462	844	223	239	2.60	
INNO-BIA AlzBio3 CSF Aβ42	461	839	222	239	2.58	
INNO-BIA AlzBio3 CSF t-Tau	460	832	229	231	2.61	
INNO-BIA AlzBio3 CSF p-Tau181	460	840	222	238	2.60	
LUMIPULSE CSF Aβ40 & Aβ42	577	1231	239	338	2.93	
LUMIPULSE CSF t-Tau	550	1128	246	304	2.90	
LUMIPULSE CSF p-Tau181	570	1209	240	330	2.94	
INNO-BIA Plasma Aβ40 & Aβ42	547	1199	191	356	2.83	

^a^DIAN Obs Data Freeze 17 (DF17) was used to compute these numbers. There are 673 participants in DF17. The cutoff for DF17 was 6/30/2023.

^b^The leftmost bar represents participants with exactly 1 assessment, 2nd leftmost bar represents participants with exactly 2 assessments, etc.

### Neuropathology core

The Neuropathology Core houses the network’s post-mortem tissue. DIAN sites are supported in providing intact fixed hemi-brain specimens for uniform neuropathologic examination. Neuropathology Core efforts include maintaining unfixed frozen and formalin-fixed tissue; a resource supporting DIAN’s Projects and available to the research community with an approved tissue request.

Fixed hemibrains are prepared in standard fashion (hemispheres coronally; cerebelli parasagittally; brainstems axially), digitally photographed, and sampled for histology, generating a set of 17 formalin-fixed, paraffin-embedded (FFPE) tissue blocks, representing the following areas: Middle frontal gyrus; anterior cingulate gyrus at the level of the genu of the corpus callosum; precentral gyrus; superior and middle temporal gyri; inferior parietal lobe (angular gyrus); occipital lobe (including the calcarine sulcus and peristriate cortex); posterior cingulate gyrus and precuneus at the level of the splenium; amygdala and entorhinal cortex; hippocampus and parahippocampal gyrus at the level of the lateral geniculate nucleus; striatum (caudate nucleus and putamen with nucleus accumbens) and olfactory cortex; lentiform nuclei (globus pallidus and putamen) at the level of the anterior commissure with the nucleus basalis of Meynert; thalamus with subthalamic nucleus; midbrain; pons; medulla oblongata; cerebellum with dentate nucleus; and cervical spinal cord. Remaining wet formalin-fixed tissue is kept in formalin in perpetuity as a research resource.

The Core prepares histology slides from a uniform set of seventeen FFPE blocks from each case. These are stained with hematoxylin and eosin for histomorphologic assessment, and with immunohistochemistry (IHC) for the more common neurodegenerative lesions, using antibodies for Aβ (10D5, Eli Lilly), phosphorylated tau (PHF1, Feinstein Institute for Medical Research, Manhasset, NY), α-synuclein (LB509, MilliporeSigma), and phosphorylated TAR DNA-binding protein of 43 kDa (pTDP-43, Cosmo Bio USA). This protocol enables the Neuropathology Core to identify and rigorously stage the pathological underpinnings of the major classes of neurodegenerative diseases. Histological slides are then reviewed and scored, using published semi-quantitative scoring criteria for histopathological lesions. These data inform the formulation of diagnoses for each case (using consensus staging and neuropathological criteria for AD [Khachaturian, CERAD, NIA-Reagan Institute, and NIA-AA]^18–24^ and for non-AD disorders^25–33^). To date, a total of 41 DIAN participant specimens have been secured. Core data and sample availability are outlined in Table 7.

**Table 7:** Neuropathology core available resources & data

Data	Value	Frequency (Percentage)
A Score (Thal phase)	5	33 (100)
B Score (Braak)	6	32 (97)
B Score (Braak)	5	1 (3)
C Score (CERAD)	3	33 (100)
Sex	Male	17 (52)
Race	White	28 (85)
Race	Other	4 (12)
Race	Asian	1 (3)
Hispanic	Yes	4 (12)

^a^DIAN Obs Data Freeze 17 (DF17) was used to compute these numbers. There are 673 participants and data from 33 brain autopsies in DF17. The cutoff for DF17 was 6/30/2023.

### Biostatistics core

The activities of the Biostatistics Core enhance the research objectives of DIAN by imparting a smooth transition from the database to statistical analyses, providing appropriate statistical analysis resources to all Cores and Projects, and developing longitudinal statistical models to test the preclinical hypotheses of DIAN on all major biomarkers of AD. The Core provides application of methodological significance as it is a necessity of state-of-the-art longitudinal statistical models to adequately estimate and compare the longitudinal rates of change on multi-modal biomarkers during the preclinical and symptomatic stages, and to assess their predictive power to cognitive decline.

The high dimensional data from Imaging Core from many modalities (MRI, PiB PET, Tau PET over a large number of brain regions) and the omics data from the Genetics and Multi-Omics Core present another unique analytic challenge to DIAN Obs. The Biostatistics Core seeks biologically meaningful dimension reduction, and conduct analyses to combine imaging markers and omics markers into composites for the test of critical hypotheses. Principal component analyses and partial least square analyses^34,35^ will be implemented, as well as methodologies developed by the Core^36,37^. The Biostatistics Core also analyzes longitudinal rates of change for these biomarkers jointly through general linear mixed models and correlate the rates of changes across modalities.

The Biostatistics Core have recently published multiple novel statistical methods driven by DIAN Obs database: analysis of biomarkers subject to detection limits^38^, correlations with family-clustered design^39^, diagnostic accuracy with ROC surface^40^, detection of unknown changepoints (in age) from multiple longitudinal biomarkers^41^, and a novel Bayesian ADAD progression model^42^. The Core continues to expand these models, and tackle other emerging analytic challenges from DIAN Obs: measurement errors in the EYO, small sample inferences on MCs who ‘escaped’ from their expected AAO, and high dimensional longitudinal data from imaging and omics. To control the false discovery rate (FDR), the Benjamini and Hochberg procedure is utilized^43^.

### Scientific projects

In 2019, DIAN Obs added three scientific Projects to the study: Project 1: Amyloid-beta, Project 2: Tau, and Project 3: Novel Mechanisms. The goal of these scientific projects is to uniquely address central scientific questions that require significant DIAN Obs Core involvement.

Project 1 aimed to define the impact of ADAD mutations and amyloidosis on amyloid β proteoforms in CSF and plasma. This was accomplished using an IP-mass spectrometry approach to capture major Aβ proteoforms in plasma, CSF, and brain. Specifically, this Project monitored Aβ37, 38, 39, 40, 42, and 43 in CSF, plasma, and brain homogenates and observed that Aβ isoform patterns of change differ. Additionally, Project 1 aimed to describe the impact of ADAD mutations in human iPSC-derived neurons and the relationship of brain proteoforms with histologic amyloid structure.

The next phase of this project will combine cell-based characterizations of individual ADAD variants with mass-spectrometry, IHC, and ELISA-based measures of Aβ burden in brain parenchyma, cerebrovasculature, CSF, and blood from ADAD pathogenic variant carriers participating in DIAN Obs and DIAN-TU. This will provide a path toward the understanding of molecular composition and variant-level diversity of deposited and soluble Aβ species, and compare these to the biochemical properties of each variant. These studies will offer a unique bench-to-bedside investigation of which types of Aβ are likely to be pathogenic, which are likely to deposit in brain and vessel walls, and how anti-amyloid therapies alter the balance of soluble Aβ species in the CNS and peripheral circulation.

The goal of Project 2 is to quantify the amount and regional distribution of tau pathology utilizing PET to illustrate differences between mutation carriers and non-carriers, investigate connections between tau pathology and other biomarkers, as well as cognitive decline. The Project also aims to validate the specificity and sensitivity of tau PET tracers (MK6240, AV1451, & PI2620) in postmortem tissue. Project 2 works to measure using mass spectrometry tau proteoforms in CSF, brain tissue, and iPSC-derived neurons, relating them to mutation status, EYO, AD biomarkers, and cognitive measures.

Initial work done within the Project used samples collected through 2017. During the study’s current grant cycle, analyses have expanded to CSF on samples collected since 2017. This expanded analysis included 411 total samples, which captured 67 individuals with longitudinal visits. From these samples, the Project derived measures of p-tau phosphorylated at different sites. Also, these samples were used to generate MTBR-tau243 data.

Project 3 aims to map molecular interactions, providing a greater explanation of how ADAD mutations, inflammation, synaptic function, and associated therapeutic targets may influence one another. The molecular profiling is completed by transcriptomics and mass spectrometry-based proteomics. Project 3 also explores defining profiles of targeted and novel fluid markers of neuroinflammation and injury to evaluate biomarker levels pre-clinically to progression to predict cognitive decline. Targeted inflammation markers in CSF include YKL-40, sTREM2, and progranulin (via immunoassay), and neuronal injury markers include CSF VILIP-1, neurogranin, SNAP-25, and NfL and plasma NfL.

### Future initiatives

DIAN Obs has led major scientific advances in the understanding of AD stages, CSF and plasma biomarkers, mechanistic links to therapeutic targets, and enabled ground-breaking prevention and interventional trials^2,9,44^. DIAN Obs has helped define the sequence, timing, and magnitude of longitudinal AD biomarker changes decades before symptoms begin^2,4,5,16^. This work directly led to the development and implementation of primary and secondary prevention trials for ADAD and the validation of the amyloid-tau-neurodegeneration (ATN) criteria^45^. DIAN Obs intends to build on these advances to further understand major contributors to disease progression, resilience, and heterogeneity, and target validation for future therapeutics.

As DIAN prepares for its next phase, study hypotheses will expand and move to be supported by home-based remote assessments, smartphone-based applications, and wearable technologies. Home health nurse visits are being incorporated into the DIAN protocol, allowing for clinical and cognitive assessments and biospecimen samples to be collected at visits occurring in years between a participant’s in-person visit. Additionally, the Cognition Core will extend the use the ARC smartphone application to include novel measures of long-term forgetting rates, which have been shown to be highly sensitive in preclinical ADAD^14,46^. Another novel task is a measure of statistical learning that assesses and evaluates learning rates over several consecutive days. Pilot data show that this is extremely sensitive to AD biomarkers in a sporadic AD population and is well tolerated by participants. Finally, DIAN will increase the generation and leverage of omic technologies to not only answer the main questions of the study but also generate new hypotheses and discover new biomarkers.

DIAN will continue its outreach to new families and regions of the world. The success demonstrated with DIAN’s South American performance sites has laid the groundwork to explore additional sites in Chile and Puerto Rico. There is a need to expand diversity in populations minimally represented in the DIAN cohort, with discussions initiated with potential collaborators in South Africa, Morocco, and Nigeria. There is also interest in re-establishing performance sites in the Western United States serving families previously identified in the region by former DIAN sites.

DIAN will maintain its lead role in defining the profiles of targeted and novel fluid markers of neuroinflammation and neuronal and synaptic injury over the course of the disease and evaluate the ability of biomarker levels at baseline and longitudinal change over time to predict cognitive decline. The landscape of biomarkers is expected to change rapidly due to amyloid removal treatment approaches. During the next years, DIAN plans to explore different omic layers to better characterize ADAD, but also to identify novel biomarkers.

**Fig. 6:**
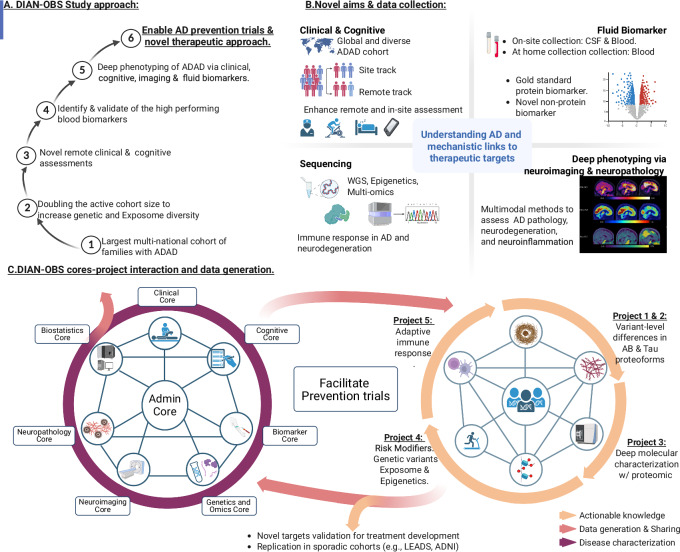
DIAN Obs Comprehensive Study Approach. A DIAN Obs, or DIAN Observational Study, progressive approach from establishing global regions through providing data to prevention and treatment trials. B Study aims and data types collected to understand Alzheimer’s disease’s mechanistic links to therapeutic targets. C DIAN Obs is multi-interdisciplinary with eight Cores and varying scientific projects. ADAD autosomal dominant Alzheimer’s disease, AD Alzheimer’s disease, WGS whole genome sequencing, CSF cerebrospinal fluid, Admin Administration, Aβ amyloid beta.

The DIAN has provided seminal discoveries in AD pathophysiology and helped define the current understanding of the sequence of events that begin two decades before the first symptom onset, and progress through a decade of dementia. These advances are made on one of the world’s largest, deeply phenotyped cohorts of both normal brain aging and AD progression. The data and sample sets are available through a comprehensive study approach (Fig. 6) to address questions and hypotheses on human brain function, aging, and AD, and with further utilization, promises to have even larger impacts.

## Supplementary information

Supplementary Information

## Data Availability

The datasets generated during the current study are not publicly available due to the sensitive nature of this human subjects data set, but are available from the corresponding author on reasonable request. All requests for data must be submitted in writing via the electronic data request form available on the DIAN website (https://dian.wustl.edu/our-research/for-investigators/dian-observational-study-investigator-resources/](https:/dian.wustl.edu/our-research/for-investigators/dian-observational-study-investigator-resources). Currently human data generated in this research is quality controlled and preserved with the DIAN Obs Biostatistics Core and in process to transition to the Washington University Becker Medical Library’s Digital Commons Data Repository and the Research Infrastructure Service (RIS). This repository meets the desirable characteristics of an acceptable NIH repository under the NIH Data Management and Sharing Policy implemented on January 25, 2023. The Core and Project primary data is highly sensitive and will be protected. Access will require a data request, followed by review and approval before access is granted. Those who wish to submit a data request will be able to see the types of data that are available, but a formal online request will be required to protect our subjects and for tracking purposes. The request form will include investigator affiliation, contact information, funding support, institutional review board (IRB) approval (if applicable), an NIH-style biosketch, and a brief description of the project, including specific aims, study design, characteristics of the data requested, and analysis plans. Investigators requesting data will also be required to sign a data user agreement and an acknowledgement agreement. The request will be received by the study data sharing committee, which will review for appropriateness. Upon their approval to release the data, a DUA will be required to be in place before the requested data is released. Each request is tracked by the DIAN Administration Core, and a data sharing report will be generated for progress and final reports to the National Institute on Aging (NIA). Each data request will specify the data elements required for the planned analyses. The Biostatistics Core personnel will prepare a file containing only these data elements, together with a participant identification number (not the DIAN ID# but an identifier recoded to protect confidentiality), so that questions about particular individuals can be resolved without the investigator’s knowledge of the participant’s identity. Image data will be available to approved investigators in post-processed formats via the DIAN Central Neuroimaging Data Archive (CNDA) after a formal DUA has been signed by both institutions. For data generated in this study, the following software may be useful for manipulating and utilizing the shared data: SAS, R, STATA, Python and MATLAB.

## Code availability

No custom code was used to generate the data described in this manuscript. Precautions to ensure confidentiality are taken by DIAN and recipients of DIAN Obs data. The final dataset is stripped of DIAN Obs identifiers and re-coded with dummy IDs prior to release and transferred only with encryption and password protection by the Biostatistics Core. For all analyses, data is provided in a blinded way with the outcome variables and then unblinded after the results of the analysis are returned to the DIAN Biostatistics Core. This ensures blinding, avoids bias and multiple comparisons (p-hacking). The Biostatistics Core enables the ability to link coded participant data across the DIAN Obs and DIAN-TU, enabling combining and comparing across the studies.
