# Supplementary material for: 15 years of longitudinal genetic, clinical, cognitive, imaging, and biochemical measures in DIAN
Source: NPJ Dement. 2026 Feb 16;2(1):13. doi: 10.1038/s44400-025-00047-7 (PMC12909123; doi:10.1038/s44400-025-00047-7)
Supplement: Supplementary file 1 — Supplementary Information [file 44400_2025_47_MOESM1_ESM.pdf]

Supplementary Materials for 15 Years of Longitudinal Genetic, Clinical, Cognitive, Imaging, and Biochemical Measures in DIAN

Alisha J. Daniels*[,1], Eric McDade[1], Jorge J. Llibre-Guerra[1], Chengjie Xiong[1], Richard J. Perrin[1], Laura Ibanez[1], Charlene Supnet-Bell[1], Carlos Cruchaga[1], Alison Goate[2], Alan E. Renton[2], Tammie L.S. Benzinger[1], Brian A. Gordon[1], Jason Hassenstab[1], Celeste Karch[1], Allan Levey[3], John C. Morris[1], Virginia Buckles[1], Ricardo F. Allegri[4], Patricio Chrem[4], Sarah B. Berman[5], Jasmeer P. Chhatwal[6], Martin R. Farlow[7], Nick C. Fox[8,9], Gregory S. Day[10], Takeshi Ikeuchi[11], Mathias Jucker[12], Johannes Levin[13,14], Jae-Hong Lee[15],David Aguillon[16], Leonel Takada[17], Ana Luisa Sosa[18], Ralph Martins[19], Hiroshi Mori[20], James M. Noble[21], Stephen Salloway[22], Edward Huey[22], Raquel Sánchez-Valle[23], Peter R. Schofield[24], Jee Hoon Roh[25], Randall J. Bateman[1] & the Dominantly Inherited Alzheimer Network.

*Correspondence to: alisha.daniels@wustl.edu

Affiliations

1 Washington University School of Medicine, St Louis, St Louis, MO, USA
2 Icahn School of Medicine at Mount Sinai, New York, NY USA
3 Goizueta Alzheimer's Disease Research Center, Emory University, Atlanta, GA, USA
4 Institute of Neurological Research FLENI, Buenos Aires, Argentina
5 University of Pittsburgh, Pittsburgh, PA, USA
6 Massachusetts General and Brigham & Women's Hospitals, Harvard Medical School, Boston MA, USA
7 Indiana University School of Medicine, Indianapolis, IN, USA
8 UK Dementia Research Institute at University College London, London, United Kingdom
9 University College London, London, United Kingdom
10 Mayo Clinic in Florida Jacksonville, FL, USA
11 Brain Research Institute, Niigata University, Niigata, Japan
12 DZNE, German Center for Neurodegenerative Diseases, Tübingen, Germany
13 DZNE, German Center for Neurodegenerative Diseases, Munich, Germany
14 Ludwig-Maximilians-Universität München, Munich, Germany
15 Asan Medical Center, Seoul, South Korea
16 Universidad de Antioquia, Medellin, Colombia
17 Fundacao Faculdade de Medicina, Sao Paulo, Brazil
18 Instituto Nacional de Neurologia y Neurocirugla Innn, Mexico City, Mexico
19 Edith Cowan University, Western Australia, Australia
20 Osaka City University, Osaka, Japan
21 Taub Institute for Research on Alzheimer's Disease and the Aging Brain, Department of Neurology, and GH Sergievsky Center, Columbia University Irving Medical Center, New York, NY, USA
22 Brown University, Butler Hospital, Providence, RI, USA
23 Hospital Clínic de Barcelona. IDIBAPS. University of Barcelona, Barcelona, Spain
24 Discipline of Psychiatry and Mental Health, University of New South Wales, Sydney, NSW, Australia
25 Korea University, Korea University Anam Hospital, Seoul, South Korea

Table of Contents

A. Supplementary Table 1 – DIAN Obs Clinical Sites (current & former) 3
B. Supplementary Table 2 – DIAN Obs Participant Entry Characteristics 4
C. Supplementary Figure 1 – DIAN Obs Organizational Structure 5
D. Supplementary File 1 – DIAN Obs Generated Publications 6
E. Supplementary File 2 – DIAN Obs Consortium Author List 35
F. Supplementary File 3 – DIAN Obs Data Dictionaries 43

A. Supplementary Table 1 – DIAN Obs Clinical Sites (current & former)

Site	Location	Site Leader	Activation Year	Status	# of Enrolled Participants
**Washington University**[i]	St. Louis, MO USA	Randall Bateman	2008	Current	124
**University of California Los Angeles**	Los Angeles, CA USA	John Ringman	2008	Former	52
**University of Munich**	Munich, Germany	Johannes Levin	2013	Current	51
**University of Tubingen**	Tubingen, Germany	Mathias Jucker	2012	Current	45
**University College London**	London, UK	Nicolas Fox	2008	Current	41
**University of Pittsburgh**	Pittsburgh, PA, USA	Sarah Berman	2012	Current	40
**Indiana University**	Indianapolis, IN USA	Martin Farlow	2008	Current	39
**Edith Cowan University**	Perth, Australia	Ralph Martins	2008	Current	38
**Brigham & Women's Hospital**	Boston, MA USA	Jasmeer Chhatwal	2008	Current	35
**University of New South Wales**	Sydney, Australia	Peter Schofield	2008	Current	31
**Columbia University**	New York, NY USA	James Noble	2008	Former	30
**Butler Hospital**	Providence, RI USA	John Huey	2008	Current	28
**Grupo Neurociencias de Antioquia**	Medellin, Colombia	David Aguillon	2021	Current	27
**University of Melbourne**	Melbourne, Australia	Colin Masters	2008	Former	24
**FLENI**[ii]	Buenos Aires, Argentina<br>Salta, Argentina	Ricardo Allegri<br>Patricio Chrem	2015<br>2021	Current<br>Current	20<br>11
**Mayo Clinic**	Jacksonville, FL USA	Gregory Day	2013	Current	9
**Asan University**[iii]	Seoul, South Korea	Jae-Hong Lee	2015	Current	8
**University of Osaka**	Osaka, Japan	Hiroshi Mori	2017	Former	7
**Hirosaki City University**	Hirosaki, Japan	Mikio Shoji	2017	Former	5
**University of Tokyo**	Tokyo, Japan	Yoshiki Niimi	2017	Current	5
**University of Southern California**	Los Angeles, CA USA	John Ringman	2016	Former	4
**Niigata University**	Niigata, Japan	Takeshi Ikeuchi	2017	Current	4
**Hospital Clinic i Provincial**	Barcelona, Spain	Raquel Sanchez-Valle	2019	Former	2
**Institute Nacional de Neuro**	Mexico City, Mexico	Ana Luisa Sosa	2023	Current	0
**University of Guadalajara**	Guadalajara, Mexico	Victor Sanchez	2023	Pending	0
**University de Sao Paulo**	Sao Paulo, Brazil	Leonel Takada	2023	Pending	0
**McGill University**	Montreal, Canada	Pedro Neto-Rosa	2023	Current	0
				**Total Enrolled:**	**673**

i. Washington University in St. Louis is also the DIAN Coordinating Center.
ii. FLENI Salta satellite site
iii. Asan University reactivated in 2022.

B. Supplementary Table 2 – DIAN Obs Participant Entry Characteristics at Enrollment

N = 673* *(Target 80% Asymptomatic, 20% Symptomatic) (*Table based on 657 participants. 16 Mutations in Process)*	**Asymptomatic** *510 (75.78%) 457 with confirmed mutation status*		**Symptomatic** *163(24.22%) 144 with confirmed mutation status*	
	243 (NC) (48.80%)	255 **(MC)** (51.20%)	17 (NC) (10.69%)	142 **(MC)** (89.31%)
**Age, Mean (SD)**	36.51 (10.95)	33.39 (9.17)	39.59 (12.56)	45.96 (9.78)
**Gender (% Female)**	142 (58.44%)	146 (57.25%)	9 (52.94%)	77 (54.23%)
**Parental Age of Onset, Mean (SD)**	47.56 (6.46)	48.20 (7.21)	46.00 (6.26)	45.47 (8.72)
**Education, Mean (SD)**	14.89 (2.82)	14.75 (2.85)	11.88 (3.69)	13.60(3.40)
**MMSE, Mean (SD)**	29.05 (1.31)	29.08 (1.21)	27.88 (1.50)	22.08 (6.99)
**ApoE4+** ***1 E4***	69 (28.40%)	71 (27.84%)	4 (23.53%)	35 (24.65%)
2 E4	3 (1.23%)	2 (0.78%)	0 (0.0%)	7 (4.93%)

MC = Mutation Carrier; NC = Non-carrier
*Table statistics based on 657 participants with confirmed mutation data available as of 06/30/2023(inclusive). Of them 397 (60.43%) are mutation carriers, 260 (39.57%) are mutation non-carriers.

C. Supplementary Figure 1 – DIAN Obs Organizational Structure

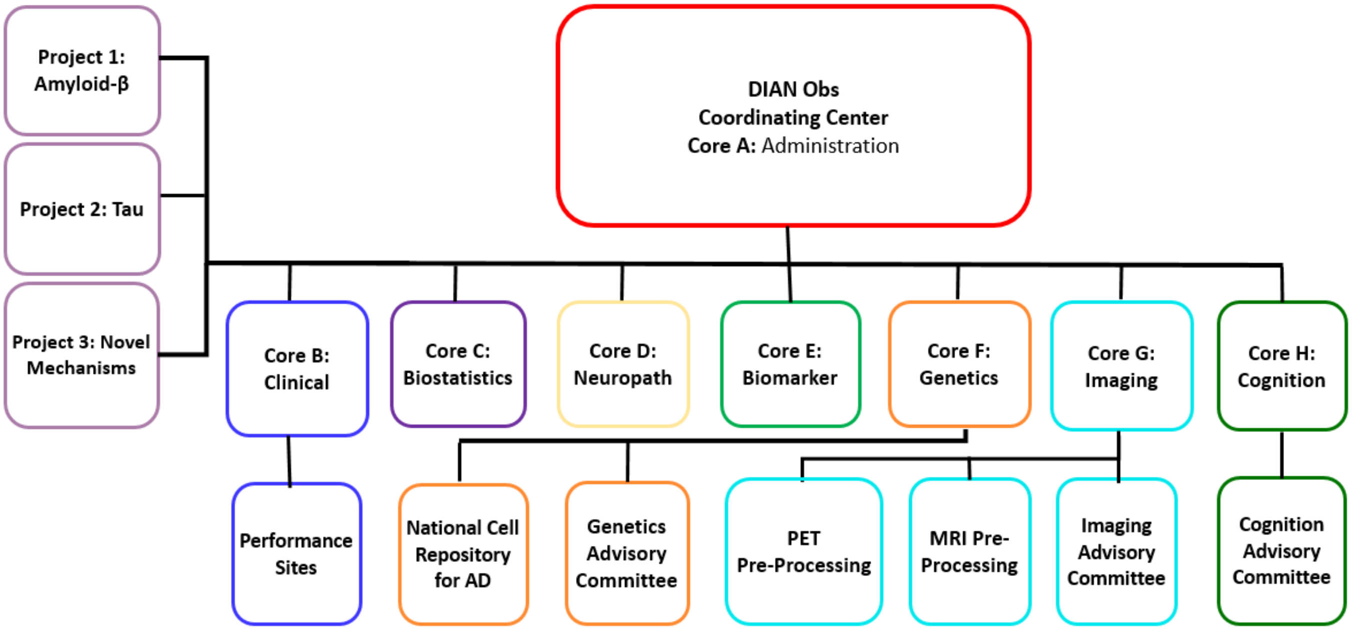

D. Supplementary File 1 - DIAN Obs Generated Publications

The following list provides a comprehensive compilation of publications generated by or utilizing data from the DIAN Obs. It encompasses seminal papers from DIAN's Cores, including Clinical, Imaging, Biomarker, Biostatistics, Neuropathology, and Genetics, that have collectively advanced the understanding of AD pathophysiology. The listed works span landmark discoveries such as the *temporal cascade of AD biomarkers*, *tau PET characterization*, *polygenic risk analyses*, and *cognitive progression modeling*. Together, these publications demonstrate how DIAN Obs data have been instrumental in defining the preclinical stages of AD, validating fluid and imaging biomarkers, informing prevention trials, and shaping current diagnostic criteria for AD.

1. Mills SM, Mallmann J, Santacruz AM, et al. Preclinical trials in autosomal dominant AD: Implementation of the DIAN-TU trial. *Rev Neurol (Paris)*. 2013;169(10):10.1016/j.neurol.2013.07.017. doi:10.1016/j.neurol.2013.07.017

2. Bateman RJ, Xiong C, Benzinger TLS, et al. Clinical and biomarker changes in dominantly inherited Alzheimer's disease. *N Engl J Med*. 2012;367(9):795-804. doi:10.1056/NEJMoa1202753

3. Benzinger TLS, Blazey T, Jack CR, et al. Regional variability of imaging biomarkers in autosomal dominant Alzheimer's disease. *Proc Natl Acad Sci*. 2013;110(47):E4502-E4509. doi:10.1073/pnas.1317918110

4. Gordon BA, Blazey TM, Su Y, et al. Spatial patterns of neuroimaging biomarker change in individuals from families with autosomal dominant Alzheimer disease: a longitudinal study. *Lancet Neurol*. 2018;17(3):241-250. doi:10.1016/S1474-4422(18)30028-0

5. McDade E, Wang G, Gordon BA, et al. Longitudinal cognitive and biomarker changes in dominantly inherited Alzheimer disease. *Neurology*. 2018;91(14):e1295-e1306. doi:10.1212/WNL.0000000000006277

6. Gordon BA, Blazey TM, Christensen J, et al. Tau PET in autosomal dominant Alzheimer's disease: relationship with cognition, dementia and other biomarkers. *Brain J Neurol*. 2019;142(4):1063-1076. doi:10.1093/brain/awz019

7. Gordon BA, Blazey TM, Su Y, Hari-Raj A, Dincer A, Flores S, Christensen J, McDade E, Wang G, Xiong C, Cairns NJ, Hassenstab J, Marcus DS, Fagan AM, Jack CR Jr, Hornbeck RC, Paumier KL, Ances BM, Berman SB, Brickman AM, Cash DM, Chhatwal JP, Correia S, Förster S, Fox NC, Graff-Radford NR, la Fougère C, Levin J, Masters CL, Rossor MN, Salloway S, Saykin AJ, Schofield PR, Thompson PM, Weiner MM, Holtzman DM, Raichle ME, Morris JC, Bateman RJ, Benzinger TLS. Spatial patterns of neuroimaging biomarker change in individuals from families with autosomal dominant Alzheimer's disease: a longitudinal study. Lancet Neurol. 2018 Mar;17(3):241-250. doi: 10.1016/S1474-4422(18)30028-0. Epub 2018 Feb 1. PubMed PMID: 29397305; PubMed Central PMCID: PMC5816717

8. Quiroz YT, Stern CE, Reiman EM, Brickhouse M, Ruiz A, Sperling RA, Lopera F, Dickerson BC. Cortical atrophy in presymptomatic Alzheimer's disease presenilin 1 mutation carriers. J Neurol Neurosurg Psychiatry. 2013 May;84(5):556-61. doi: 10.1136/jnnp-2012-303299. Epub 2012 Nov 7. PMID: 23134660; PMCID: PMC3632663.

9. Ridha BH, Barnes J, Bartlett JW, Godbolt A, Pepple T, Rossor MN, Fox NC. Tracking atrophy progression in familial Alzheimer's disease: a serial MRI study. Lancet Neurol. 2006 Oct;5(10):828-34. doi: 10.1016/S1474-4422(06)70550-6. PMID: 16987729.

10. Bateman RJ, Benzinger TL, Berry S, et al. The DIAN-TU Next Generation Alzheimer's prevention trial: Adaptive design and disease progression model. *Alzheimers Dement J Alzheimers Assoc*. 2017;13(1):8-19. doi:10.1016/j.jalz.2016.07.005

11. Washington University in St. Louis. (2025). Publication Policy. Dominantly Inherited Alzheimer Network (DIAN). https://dian.wustl.edu/for-investigators/dian-observational-study-investigator-resources/publication-policy/

12. Cruchaga C, Del-Aguila JL, Saef B, Black K, Fernandez MV, Budde J, Ibanez L, Deming Y, Kapoor M,

Tosto G, Mayeux RP, Holtzman DM, Fagan AM, Morris JC, Bateman RJ, Goate AM, Harari O. Polygenic risk score of sporadic late-onset Alzheimer's disease reveals a shared architecture with the familial and early-onset forms. Alzheimers Dement. 2018 Feb;14(2):205-214. doi: 10.1016/j.jalz.2017.08.013. Epub 2017 Sep 21. PubMed PMID: 28943286; PubMed Central PMCID: PMC5803427

13. Timsina J, Gomez-Fonseca D, Wang L, et al. Comparative Analysis of Alzheimer's Disease Cerebrospinal Fluid Biomarkers Measurement by Multiplex SOMAscan Platform and Immunoassay-Based Approach. *J Alzheimers Dis JAD*. 2022;89(1):193-207. doi:10.3233/JAD-220399

14. Sung YJ, Yang C, Norton J, et al. Proteomics of brain, CSF, and plasma identifies molecular signatures for distinguishing sporadic and genetic Alzheimer's disease. *Sci Transl Med*. 2023;15(703):eabq5923. doi:10.1126/scitranslmed.abq5923

15. Kurylo DD, Corkin S, Iii JFR. Greater relative impairment of object recognition than of visuospatial abilities in Alzheimer's disease. Neuropsychology. 1996; 10(1):74-81.

16. Llibre-Guerra JJ, Li Y, Schindler SE, Gordon BA, Fagan AM, Morris JC, Benzinger TLS, Hassenstab J, Wang G, Allegri R, Berman SB, Chhatwal J, Farlow MR, Holtzman DM, Jucker M, Levin J, Noble JM, Salloway S, Schofield P, Karch C, Fox NC, Xiong C, Bateman RJ, McDade E. Association of Longitudinal Changes in Cerebrospinal Fluid Total Tau and Phosphorylated Tau 181 and Brain Atrophy With Disease Progression in Patients With Alzheimer Disease. JAMA Netw Open. 2019 Dec 2;2(12):e1917126. doi: 10.1001/jamanetworkopen.2019.17126. PubMed PMID: 31825500; PubMed Central PMCID: PMC6991202

17. McKay NS, Gordon BA, Hornbeck RC, et al. Positron emission tomography and magnetic resonance imaging methods and datasets within the Dominantly Inherited Alzheimer Network (DIAN). *Nat Neurosci*. 2023;26(8):1449-1460. doi:10.1038/s41593-023-01359-8

18. O'Connor A, et al. Tau acculuation in autosomal dominant Alzheimer's disease: a longitudinal [18F]flortaucipir study. *Alzheimers Res Ther* 15, 1-11 (2023). PMCID: PMC10210376

19. Braak H and Braak E. Neuropathological stageing of Alzheimer-related changes. *Acta*

Neuropathologica 1991; 82: 239-259

20. Braak H, Alafuzoff I, Arzberger T, Kretzschmar H, Tredici KD. Staging of Alzheimer disease-associated neurofibrillary pathology using paraffin sections and immunocytochemistry. *Acta Neuropathologica* 2006; 112: 389-404

21. Thal DR, Rub U, Orantes M, Braak H. Phases of amyloid-beta deposition in the human brain and its

relevance for the development of AD. *Neurology*. 2002;58:1791

22. Khachaturian ZS. Diagnosis of Alzheimer's disease. *Archives of Neurology* 1985; 42: 1097-1105

23. Mirra SS, Heyman A, McKeel D, Sumi SM, Crain BJ, Brownlee LM, Vogel FS, Hughes JP, van BG, Berg L. The Consortium to Establish a Registry for Alzheimer's Disease (CERAD). Part II. Standardization of the neuropathologic assessment of Alzheimer's disease. *Neurology* 1991;41:479-486. PMID: 2011243

24. National Institute on Aging and Reagan Institute Working Group. Consensus recommendations for

the postmortem diagnosis of Alzheimer's disease. *Neurobiology of Aging* 1997; 18: S1-S2.5.4

25. Montine TJ, Phelps CH, Beach TG, Bigio EH, Cairns NJ, Dickson DW, Duyckaerts C, Frosch MP, Masliah E, Mirra SS, Nelson PT, Schneider JA, Thal DR, Trojanowski JQ, Vinters HV, Hyman BT. National Institute on Aging-Alzheimer's Association guidelines for the neuropathologic assessment of Alzheimer's disease: a practical approach. *Acta Neuropathol* 2012;123:1-11. PMCID: PMC3268003

26. McKeith IG, Galasko D, Kosaka K, Perry EK, Dickson DW, Hansen LA, Salmon DP, Lowe J, Mirra SS,

Byrne EJ, Lennox G, Quinn NP, Edwardson JA, Ince PG, Bergeron C, Burns A, Miller BL, Lovestone S, Collerton D, Jansen EN, Ballard C, de Vos RA, Wilcock GK, Jellinger KA, Perry RH. Consensus guidelines for the clinical and pathologic diagnosis of dementia with Lewy bodies (DLB): report of the consortium on DLB international workshop. *Neurology* 1996;47:1113-1124. PMID: 8909416

27. McKeith I, Dickson DW, Lowe J, Emre M, O'Brien JT, Feldman H, Cummings J, Duda JE, Lippa C, Perry EK, Aarsland D, Arai H, Ballard CG, Boeve B, Burn DJ, Costa D, Del Ser T, Dubois B, Galasko D, Gauthier S, Goetz CG, Gomez-Tortosa E, Halliday G, Hansen LA, Hardy J, Iwatsubo T, Kalaria RN, Kaufer D, Kenny RA, Korczyn A, Kosaka K, Lee VMY, Lees A, Litvan I, Londos E, Lopez OL, Minoshima S, Mizuno Y, Molina JA, Mukaetova-Ladinska EB, Pasquier F, Perry RH, Schulz JB, Trojanowski JQ, Yamada M; Consortium on DLB Diagnosis and management of dementia with Lewy bodies: Third report of the DLB consortium. *Neurology* 2005; 65: 1863-1872

28. McKeith IG. Consensus guidelines for the clinical and pathologic diagnosis of dementia with Lewy bodies (DLB): report of the Consortium on DLB International Workshop. *J Alzheimers Dis* 2006;9:417-423. PMID: 16914880

29. Braak H, Tredici KD, Rub U, de Vos RAI, Steur ENHJ, Braak E. Staging of brain pathology related to sporadic Parkinson's disease. *Neurobiology of Aging* 2003; 24: 197-211

30. Attems J, Toledo JB, Walker L, Gelpi E, Gentleman S, Halliday G, Hortobagyi T, Jellinger K, Kovacs GG, Lee EB, Love S, McAleese KE, Nelson PT, Neumann M, Parkkinen L, Polvikoski T, Sikorska B, Smith C, Grinberg LT, Thal DR, Trojanowski JQ, McKeith IG. Neuropathological consensus criteria for the evaluation of Lewy pathology in post-mortem brains: a multi-centre study. *Acta Neuropathol*. 2021 Feb;141(2):159-172. doi: 10.1007/s00401-020-02255-2. Epub 2021 Jan 5. PMID: 33399945

31. Cairns NJ, Bigio EH, Mackenzie IR, Neumann M, Lee VM, Hatanpaa KJ, White CL, III, Schneider JA, Grinberg LT, Halliday G, Duyckaerts C, Lowe JS, Holm IE, Tolnay M, Okamoto K, Yokoo H, Murayama S, Woulfe J, Munoz DG, Dickson DW, Ince PG, Trojanowski JQ, Mann DM. Neuropathologic diagnostic and nosologic criteria for frontotemporal lobar degeneration: consensus of the Consortium for Frontotemporal Lobar Degeneration. *Acta Neuropathol* 2007;114:5-22. PMCID: PMC3268

32. Mackenzie IR, Neumann M, Bigio EH, Cairns NJ, Alafuzoff I, Kril J, Kovacs GG, Ghetti B, Halliday G, Holm IE, Ince PG, Kamphorst W, Revesz T, Rozemuller AJ, Kumar-Singh S, Akiyama H, Baborie A, Spina S, Dickson DW, Trojanowski JQ, Mann DM. Nomenclature and nosology for neuropathologic subtypes of frontotemporal lobar degeneration: an update. *Acta Neuropathol* 2010;119:1-4. PMCID: PMC2799633

33. Josephs KA, Murray ME, Whitwell JL, Tosakulwong N, Weigand SD, Petrucelli L, Liesinger AM, Petersen RC, Parisi JE, Dickson DW. Updated TDP-43 in Alzheimer's disease staging scheme. *Acta Neuropathol*. 2016 Apr;131(4):571-85. Doi: 10.1007/s00401-016-1537-1. Epub 2016 Jan 25. PMID: 26810071

34. Nelson PT, Lee EB, Cykowski MD, Alafuzoff I, Arfanakis K, Attems J, Brayne C, Corrada MM, Dugger BN, Flanagan ME, Ghetti B, Grinberg LT, Grossman M, Grothe MJ, Halliday GM, Hasegawa M, Hokkanen SRK, Hunter S, Jellinger K, Kawas CH, Keene CD, Kouri N, Kovacs GG, Leverenz JB, Latimer CS, Mackenzie IR, Mao Q, McAleese

KE, Merrick R, Montine TJ, Murray ME, Myllykangas L, Nag S, Neltner JH, Newell KL, Rissman RA, Saito Y, Sajjadi SA, Schwetye KE, Teich AF, Thal DR, Tomé SO, Troncoso JC, Wang SJ, White CL 3rd, Wisniewski T, Yang HS, Schneider JA, Dickson DW, Neumann M. LATE-NC staging in routine neuropathologic diagnosis: an update. *Acta Neuropathol.* 2023 Feb; 145(2):159-173. doi: 10.1007/s00401-022-02524-2. Epub 2022 Dec 13. PMID: 36512061

35. Gregoria Mateos-Aparicio (2011) Partial Least Squares (PLS) Methods: Origins, Evolution, and Application to Social Sciences, Communications in Statistics - Theory and Methods, 40:13, 2305-2317, DOI: 10.1080/03610921003778225

36. Krishnan A, Williams LJ, McIntosh AR, Abdi H. Partial Least Squares (PLS) methods for neuroimaging: a tutorial and review. Neuroimage 2011; 56(2):455-75

37. Xiong C, McKeel DW, Miller JP, Morris JC. Combining correlated diagnostic tests---application to neuropathologic diagnosis of Alzheimer's disease. Medical Decision Making. 2004, 24 (6): 659-669

38. Xiong C, van Belle G, Chen K, Tian L, Luo J, Gao F, Yan Y, Chen L, Morris JC, Crane P. Combining Multiple Markers to Optimize the Longitudinal Rate of Progression-Application to Clinical Trials on the Early Stage of Alzheimer's Disease. Statistics in Biopharmaceutical Research DOI: 10.1080/19466315.2012.756662, 2013; 5(1):54-66

39. Xiong C, Luo J, Agboola F, Grant E, Morris JC. A family of estimators to diagnostic accuracy when candidate tests are subject to detection limits-Application to diagnosing early stage Alzheimer disease. Stat Methods Med Res. 2022 May;31(5):882-898. doi: 10.1177/09622802211072511. Epub 2022 Jan 19. PMID: 35044258; PMCID: PMC9018582

40. Luo J, Gao F, Lu J, Wang G, Chen L, Fagan AM, Day GS, Voglein J, Chhatwal JP, Xiong C. Statistical Estimation and Comparison of Group-Specific Bivariate Correlation Coefficients in Family-type Cluster Studies. Journal of Applied Statistics. 2021 Feb 28. PMID: 35755087; PMCID: PMC9225315

41. Xiong C, Luo J, Chen L, Gao F, Liu J, Wang G, Bateman R, Morris JC. Estimating diagnostic accuracy for clustered ordinal diagnostic groups in the three-class case--application to the early diagnosis of Alzheimer disease. Statistical Methods in Medical Research, 2018 27(3):701-714. PubMed PMID: 29182052; PubMed Central PMCID: PMC5841923

42. Luo J, Agboola F, Grant E, Morris JC, Masters CL, Albert MS, Johnson SC, McDade EM, Fagan AM, Benzinger TLS, Hassenstab J, Bateman RJ, Perrin RJ, Wang G, Li Y, Gordon B, Cruchaga C, Day GS, Levin J, Vöglein J, Ikeuchi T, Suzuki K, Allegri RF, Xiong C; Dominantly Inherited Alzheimer Network (DIAN OBS). Accelerated longitudinal changes and ordering of Alzheimer disease biomarkers across the adult lifespan. Brain. 2022 Dec 19;145(12):4459-4473. doi: 10.1093/brain/awac238. PMID: 35925685

43. Wang G, Berry S, Xiong C, Hassenstab J, Quintana M, McDade EM, Delmar P, Vestrucci M, Sethuraman G, Bateman RJ; Dominantly Inherited Alzheimer Network Trials Unit. A novel cognitive disease progression model for clinical trials in autosomal- dominant Alzheimer's disease. Stat Med. 2018 May 14. doi: 10.1002/sim.7811

44. Benjamini Y, Hochberg Y. Controlling the false discovery rate: a practical and powerful approach to multiple testing. Journal of the Royal Statistical Society, Series B 1995; 57: 289–300

45. Johnson, E.C.B., Bian, S., Haque, R.U. *et al.* Cerebrospinal fluid proteomics define the natural history of autosomal dominant Alzheimer's disease. *Nat Med* **29**, 1979–1988 (2023). https://doi.org/10.1038/s41591-023-02476-4

46. Jack CR, Andrews JS, Beach TG, et al. Revised criteria for diagnosis and staging of Alzheimer's disease: Alzheimer's Association Workgroup. *Alzheimer's Dement*. 2024; 20: 5143–5169. https://doi.org/10.1002/alz.13859

47. Suzuki A, Shinozaki J, Yazawa S, Ueki Y, Matsukawa N, Shimohama S, Nagamine T. Establishing a new screening system for mild cognitive impairment and Alzheimer's disease with mental rotation tasks that evaluate visuospatial function. Journal Alzheimer's Dis. 2018Jan 23; 61 (4): 1653-1665. PMID:29376869

48. Aschenbrenner AJ, James BD, McDade E, et al. Awareness of Genetic Risk in the Dominantly Inherited Alzheimer Network (DIAN). *Alzheimers Dement J Alzheimers Assoc*. 2020;16(1):219-228. doi:10.1002/alz.12010

49. McKay NS, Dincer A, Mehrotra V, Aschenbrenner AJ, Balota D, Hornbeck RC, Hassenstab J, Morris JC, Benzinger TLS, Gordon BA. Beta-amyloid moderates the relationship between cortical thickness and attentional control in middle- and older-aged adults. Neurobiology of aging. Neurobiol Aging. 2022 Apr;112:181-190. doi: 10.1016/j.neurobiolaging.2021.12.012. Epub 2022 Jan 10. PMID: 35227946

50. Morenas-Rodríguez E, Li Y, Nuscher B, Franzmeier N, Xiong C, Suárez-Calvet M, Fagan AM, Schultz S, Gordon BA, Benzinger TLS, Hassenstab J, McDade E, Feederle R, Karch CM, Schlepckow K, Morris JC, Kleinberger G, Nellgard B, Vöglein J, Blennow K, Zetterberg H, Ewers M, Jucker M, Levin J, Bateman RJ, Haass C. Soluble TREM2 in CSF and its association with other biomarkers and cognition in autosomal-dominant Alzheimer's disease: a longitudinal observational study. The Lancet. Neurology. 2022 April;21(4):329-341. PubMed PMID: 35305339; PubMed Central PMCID: PMC8926925; DOI: 10.1016/S1474-4422(22)00027-8

51. Chen HH, Eteleeb A, Wang C, Fernandez MV, Budde JP, Bergmann K, Norton J, Wang F, Ebl C, Morris JC, Perrin RJ, Bateman RJ, McDade E, Xiong C, Goate A, Farlow M, Chhatwal J, Schofield PR, Chui H, Harari O, Cruchaga C, Ibanez L. Circular RNA detection identifies circPSEN1 alterations in brain specific to autosomal dominant Alzheimer's disease. Acta neuropathologica communications. 2022 March 4;10(1):29. PubMed PMID: 35246267; PubMed Central PMCID: PMC8895634; DOI: 10.1186/s40478-022-01328-5

52. Lim YY, Maruff P, Barthélemy NR, Goate A, Hassenstab J, Sato C, Fagan AM, Benzinger TLS, Xiong C, Cruchaga C, Levin J, Farlow MR, Graff-Radford NR, Laske C, Masters CL, Salloway S, Schofield PR, Morris JC, Bateman RJ, McDade E. Association of BDNF Val66Met With Tau Hyperphosphorylation and Cognition in Dominantly Inherited Alzheimer Disease. JAMA neurology. 2022 March 1;79(3):261-270. PubMed PMID: 35099506; PubMed Central PMCID: PMC8804973; DOI: 10.1001/jamaneurol.2021.5181

53. Raman F, Fang YD, Grandhi S, Murchison CF, Kennedy RE, Morris JC, Massoumzadeh P, Benzinger T, Roberson ED, McConathy J. Dynamic Amyloid PET: Relationships to 18FFlortaucipir Tau PET Measures. Journal of nuclear medicine : official publication, Society of Nuclear Medicine. 2022 February;63(2):287-293. PubMed PMID: 34049986; PubMed Central PMCID: PMC8805772; DOI: 10.2967/jnumed.120.254490

54. Chhatwal JP, Schultz SA, McDade E, Schultz AP, Liu L, Hanseeuw BJ, Joseph-Mathurin N, Feldman R, Fitzpatrick CD, Sparks KP, Levin J, Berman SB, Renton AE, Esposito BT, Fernandez MV, Sung YJ, Lee JH, Klunk WE, Hofmann A, Noble JM, Graff-Radford N, Mori H, Salloway SM, Masters CL, Martins R, Karch CM, Xiong C, Cruchaga C, Perrin RJ, Gordon BA, Benzinger TLS, Fox NC, Schofield PR, Fagan AM, Goate AM, Morris JC, Bateman RJ, Johnson KA, Sperling RA. Variant-dependent heterogeneity in amyloid β burden in autosomal dominant Alzheimer's disease: cross-sectional and longitudinal analyses of an observational study. The Lancet. Neurology. 2022 February;21(2):140-152. PubMed PMID: 35065037; PubMed Central PMCID: PMC8956209; DOI: 10.1016/S1474-4422(21)00375-6.

55. Hubbard EE, Heil LR, Merrihew GE, Chhatwal JP, Farlow MR, McLean CA, Ghetti B, Newell KL, Frosch MP, Bateman RJ, Larson EB, Keene CD, Perrin RJ, Montine TJ, MacCoss MJ, Julian RR. Does Data-Independent Acquisition Data Contain Hidden Gems? A Case Study Related to Alzheimer's Disease. Journal of proteome research. 2022 January 7;21(1):118-131. PubMed PMID: 34818016; PubMed Central PMCID: PMC8741752; DOI:10.1021/acs.jproteome.1c00558

56. Koenig LN, LaMontagne P, Glasser MF, Bateman R, Holtzman D, Yakushev I, Chhatwal J, Day GS, Jack C, Mummery C, Perrin RJ, Gordon BA, Morris JC, Shimony JS, Benzinger TLS. Regional age-related atrophy after screening for preclinical alzheimer disease. Neurobiology of aging. 2022 January;109:43-51. PubMed PMID: 34655980; PubMed Central PMCID: PMC9009406; DOI: 10.1016/j.neurobiolaging.2021.09.010

57. Buckles VD, Xiong C, Bateman RJ, Hassenstab J, Allegri R, Berman SB, Chhatwal JP, Danek A, Fagan AM, Ghetti B, Goate A, Graff-Radford N, Jucker M, Levin J, Marcus DS, Masters CL, McCue L, McDade E, Mori H, Moulder KL, Noble JM, Paumier K, Preische O, Ringman JM, Fox NC, Salloway S, Schofield PR, Martins R, Vöglein J, Morris JC. Different rates of cognitive decline in autosomal dominant and late-onset Alzheimer disease. Alzheimer's & dementia : the journal of the Alzheimer's Association. 2021 December 2. PubMed PMID: 34854530; DOI: 10.1002/alz.12505

58. Therneau TM, Knopman DS, Lowe VJ, Botha H, Graff-Radford J, Jones DT, Vemuri P, Mielke MM, Schwarz CG, Senjem ML, Gunter JL, Petersen RC, Jack CR Jr. Relationships between β-amyloid and tau in an elderly population: An accelerated failure time model. NeuroImage. 2021 November 15;242:118440. PubMed PMID: 34333107; PubMed Central PMCID: PMC8499700; DOI: 10.1016/j.neuroimage.2021.118440

59. Lucey BP, Wisch J, Boerwinkle AH, Landsness EC, Toedebusch CD, McLeland JS, Butt OH, Hassenstab J, Morris JC, Ances BM, Holtzman DM. Sleep and longitudinal cognitive performance in preclinical and early symptomatic Alzheimer's disease. Brain : a journal of neurology. 2021 October 22;144(9):2852-2862. PubMed PMID: 34668959; PubMed Central PMCID: PMC8536939; DOI: 10.1093/brain/awab272

60. Chen CD, Joseph-Mathurin N, Sinha N, Zhou A, Li Y, Friedrichsen K, McCullough A, Franklin EE, Hornbeck R, Gordon B, Sharma V, Cruchaga C, Goate A, Karch C, McDade E, Xiong C, Bateman RJ, Ghetti B, Ringman JM, Chhatwal J, Masters CL, McLean C, Lashley T, Su Y, Koeppe R, Jack C, Klunk WE, Morris JC, Perrin RJ, Cairns NJ, Benzinger TLS. Comparing amyloid-β plaque burden with antemortem PiB PET in autosomal dominant and late-onset Alzheimer disease. Acta neuropathologica. 2021 October;142(4):689-706. PubMed PMID: 34319442; PubMed Central PMCID: PMC8815340; DOI: 10.1007/s00401-021-02342-y

61. Gonneaud J, Baria AT, Pichet Binette A, Gordon BA, Chhatwal JP, Cruchaga C, Jucker M, Levin J, Salloway S, Farlow M, Gauthier S, Benzinger TLS, Morris JC, Bateman RJ, Breitner JCS, Poirier J, Vachon-Presseau E, Villeneuve S. Accelerated functional brain aging in preclinical familial Alzheimer's disease. Nature communications. 2021 September 9;12(1):5346. PubMed PMID: 34504080; PubMed Central PMCID: PMC8429427; DOI: 10.1038/s41467-021- 25492-9

62. Zhou Y, Flores S, Mansor S, Hornbeck RC, Tu Z, Perlmutter JS, Ances B, Morris JC, Gropler RJ, Benzinger TLS. Spatially constrained kinetic modeling with dual reference tissues improves 18F-flortaucipir PET in studies of Alzheimer disease. European journal of nuclear medicine and molecular imaging. 2021 September;48(10):3172-3186. PubMed PMID: 33599811; PubMed Central PMCID: PMC8371062; DOI: 10.1007/s00259-020-05134-w

63. Ewers M, Luan Y, Frontzkowski L, Neitzel J, Rubinski A, Dichgans M, Hassenstab J, Gordon BA, Chhatwal JP, Levin J, Schofield P, Benzinger TLS, Morris JC, Goate A, Karch CM, Fagan AM, McDade E, Allegri R, Berman S, Chui H, Cruchaga C, Farlow M, Graff-Radford N, Jucker M, Lee JH, Martins RN, Mori H, Perrin R, Xiong C, Rossor M, Fox NC, O'Connor A, Salloway S, Danek A, Buerger K, Bateman RJ, Habeck C, Stern Y,

Franzmeier N. Segregation of functional networks is associated with cognitive resilience in Alzheimer's disease. Brain: a journal of neurology. 2021 August 17;144(7):2176-2185. PubMed PMID: 33725114; PubMed Central PMCID: PMC8370409; DOI: 10.1093/brain/awab112

64. Fagan AM, Henson RL, Li Y, Boerwinkle AH, Xiong C, Bateman RJ, Goate A, Ances BM, Doran E, Christian BT, Lai F, Rosas HD, Schupf N, Krinsky-McHale S, Silverman W, Lee JH, Klunk WE, Handen BL, Allegri RF, Chhatwal JP, Day GS, Graff-Radford NR, Jucker M, Levin J, Martins RN, Masters CL, Mori H, Mummery CJ, Niimi Y, Ringman JM, Salloway S, Schofield PR, Shoji M, Lott IT. Comparison of CSF biomarkers in Down syndrome and autosomal dominant Alzheimer's disease: a cross-sectional study. The Lancet. Neurology. 2021 August;20(8):615-626. PubMed PMID: 34302786; PubMed Central PMCID: PMC8496347; DOI: 10.1016/S1474-4422(21)00139-3

65. McDade E, Llibre-Guerra JJ, Holtzman DM, Morris JC, Bateman RJ. The informed road map to prevention of Alzheimer Disease: A call to arms. Molecular neurodegeneration. 2021 July 21;16(1):49. PubMed PMID: 34289882; PubMed Central PMCID: PMC8293489; DOI: 10.1186/s13024-021-00467-y

66. Salloway S, Farlow M, McDade E, Clifford DB, Wang G, Llibre-Guerra JJ, Hitchcock JM, Mills SL, Santacruz AM, Aschenbrenner AJ, Hassenstab J, Benzinger TLS, Gordon BA, Fagan AM, Coalier KA, Cruchaga C, Goate AA, Perrin RJ, Xiong C, Li Y, Morris JC, Snider BJ, Mummery C, Surti GM, Hannequin D, Wallon D, Berman SB, Lah JJ, Jimenez-Velazquez IZ, Roberson ED, van Dyck CH, Honig LS, Sánchez-Valle R, Brooks WS, Gauthier S, Galasko DR, Masters CL, Brosch JR, Hsiung GR, Jayadev S, Formaglio M, Masellis M, Clarnette R, Pariente J, Dubois B, Pasquier F, Jack CR Jr, Koeppe R, Snyder PJ, Aisen PS, Thomas RG, Berry SM, Wendelberger BA, Andersen SW, Holdridge KC, Mintun MA, Yaari R, Sims JR, Baudler M, Delmar P, Doody RS, Fontoura P, Giacobino C, Kerchner GA, Bateman RJ. A trial of gantenerumab or solanezumab in dominantly inherited Alzheimer's disease. Nature medicine. 2021 July;27(7):1187-1196. PubMed PMID: 34155411; PubMed Central PMCID: PMC8988051; DOI: 10.1038/s41591-021-01369-8

67. Chen Y, Ying C, Binkley MM, Juttukonda MR, Flores S, Laforest R, Benzinger TLS, An H. Deep learning-based T1-enhanced selection of linear attenuation coefficients (DL-TESLA) for PET/MR attenuation correction in dementia neuroimaging. Magnetic resonance in medicine. 2021 July;86(1):499-513. PubMed PMID: 33559218; PubMed Central PMCID: PMC8091494; DOI: 10.1002/mrm.28689

68. Day GS, Yarbrough MY, Körtvelyessy P, Prüss H, Bucelli RC, Fritzler MJ, Mason W, Tang-Wai DF, Steriade C, Hébert J, Henson RL, Herries EM, Ladenson JH, Lopez Chiriboga AS, Graff-Radford NR, Morris JC, Fagan A. Prospective Quantification of CSF Biomarkers in Antibody-Mediated Encephalitis. Neurology. 2021 May 18;96(20):e2546-e2557. PubMed PMID: 33795390; PubMed Central PMCID: PMC8205475; DOI: 10.1212/WNL.0000000000011937

69. Pichet Binette A, Theaud G, Rheault F, Roy M, Collins DL, Levin J, Mori H, Lee JH, Farlow MR, Schofield P, Chhatwal JP, Masters CL, Benzinger T, Morris J, Bateman R, Breitner JC, Poirier J, Gonneaud J, Descoteaux M, Villeneuve S. Bundle-specific associations between white matter microstructure and Aβ and tau pathology in preclinical Alzheimer's disease. eLife. 2021 May 13;10. PubMed PMID: 33983116; PubMed Central PMCID: PMC8169107; DOI:10.7554/eLife.62929

70. Martin WRW, Miles M, Zhong Q, Hartlein J, Racette BA, Norris SA, Ushe M, Maiti B, Criswell S, Davis AA, Kotzbauer PT, Cairns NJ, Perrin RJ, Perlmutter JS. Is Levodopa Response a Valid Indicator of Parkinson's Disease?. Movement disorders : official journal of the Movement Disorder Society. 2021 April;36(4):948-954. PubMed PMID: 33253432; PubMed Central PMCID: PMC8046721; DOI: 10.1002/mds.28406

71. Liu J, Xiong C, Liu L, Wang G, Jingqin L, Gao F, Chen L, Li Y. Relative efficiency of equal versus unequal cluster sizes in cluster randomized trials with a small number of clusters. Journal of biopharmaceutical

statistics. 2021 March;31(2):191-206. PubMed PMID: 32970522; PubMed Central PMCID: PMC8734433; DOI: 10.1080/10543406.2020.1814795

72. Pannee J, Shaw LM, Korecka M, Waligorska T, Teunissen CE, Stoops E, Vanderstichele HMJ, Mauroo K, Verberk IMW, Keshavan A, Pesini P, Sarasa L, Pascual-Lucas M, Fandos N, Allué JA, Portelius E, Andreasson U, Yoda R, Nakamura A, Kaneko N, Yang SY, Liu HC, Palme S, Bittner T, Mawuenyega KG, Ovod V, Bollinger J, Bateman RJ, Li Y, Dage JL, Stomrud E, Hansson O, Schott JM, Blennow K, Zetterberg H. The global Alzheimer's Association round robin study on plasma amyloid β methods. Alzheimer's & dementia (Amsterdam, Netherlands). 2021;13(1):e12242. PubMed PMID: 34692980; PubMed Central PMCID: PMC8515356; DOI: 10.1002/dad2.12242

73. Keret O, Staffaroni AM, Ringman JM, Cobigo Y, Goh SM, Wolf A, Allen IE, Salloway S, Chhatwal J, Brickman AM, Reyes-Dumeyer D, Bateman RJ, Benzinger TLS, Morris JC, Ances BM, Joseph-Mathurin N, Perrin RJ, Gordon BA, Levin J, Vöglein J, Jucker M, la Fougère C, Martins RN, Sohrabi HR, Taddei K, Villemagne VL, Schofield PR, Brooks WS, Fulham M, Masters CL, Ghetti B, Saykin AJ, Jack CR, Graff-Radford NR, Weiner M, Cash DM, Allegri RF, Chrem P, Yi S, Miller BL, Rabinovici GD, Rosen HJ; Dominantly Inherited Alzheimer Network. Pattern and degree of individual brain atrophy predicts dementia onset in dominantly inherited Alzheimer's disease. Alzheimer's & dementia (Amsterdam, Netherlands). 2021;13(1):e12197. PubMed PMID: 34258377; PubMed Central PMCID: PMC8256623; DOI:10.1002/dad2.12197

74. Franzmeier N, Ren J, Damm A, Monté-Rubio G, Boada M, Ruiz A, Ramirez A, Jessen F, Düzel E, Rodríguez Gómez O, Benzinger T, Goate A, Karch CM, Fagan AM, McDade E, Buerger K, Levin J, Duering M, Dichgans M, Suárez-Calvet M, Haass C, Gordon BA, Lim YY, Masters CL, Janowitz D, Catak C, Wolfsgruber S, Wagner M, Milz E, Moreno-Grau S, Teipel S, Grothe MJ, Kilimann I, Rossor M, Fox N, Laske C, Chhatwal J, Falkai P, Perneczky R, Lee JH, Spottke A, Boecker H, Brosseron F, Fliessbach K, Heneka MT, Nestor P, Peters O, Fuentes M, Menne F, Priller J, Spruth EJ, Franke C, Schneider A, Westerteicher C, Speck O, Wiltfang J, Bartels C, Araque Caballero MÁ, Metzger C, Bittner D, Salloway S, Danek A, Hassenstab J, Yakushev I, Schofield PR, Morris JC, Bateman RJ, Ewers M. The $BDNF_{Val66Met}$ SNP modulates the association between beta-amyloid and hippocampal disconnection in Alzheimer's disease. Mol Psychiatry. 2021 Feb;26(2):614-628. doi: 10.1038/s41380-019-0404-6. Epub 2019 Mar 21. PubMed PMID: 30899092; PubMed Central PMCID: PMC6754794

75. Chatterjee P, Fagan AM, Xiong C, McKay M, Bhatnagar A, Wu Y, Singh AK, Taddei K, Martins I, Gardener SL, Molloy MP, Multhaup G, Masters CL, Schofield PR, Benzinger TLS, Morris JC, Bateman RJ, Greenberg SM, Wermer MJH, van Buchem MA, Sohrabi HR, Martins RN. Presymptomatic Dutch-Type Hereditary Cerebral Amyloid Angiopathy-Related Blood Metabolite Alterations. J Alzheimers Dis. 2021;79(2):895-903. doi: 10.3233/JAD-201267. PubMed PMID: 33361604

76. Luo J, Agboola F, Grant E, Masters CL, Albert MS, Johnson SC, McDade EM, Vöglein J, Fagan AM, Benzinger T, Massoumzadeh P, Hassenstab J, Bateman RJ, Morris JC, Perrin RJ, Chhatwal J, Jucker M, Ghetti B, Cruchaga C, Graff-Radford NR, Schofield PR, Mori H, Xiong C. Sequence of Alzheimer disease biomarker changes in cognitively normal adults: A cross-sectional study. Neurology. 2020 Dec 8;95(23):e3104-e3116. doi: 10.1212/WNL.0000000000010747. Epub 2020 Sep 1. PubMed PMID: 32873693; PubMed Central PMCID: PMC7734923

77. Finsterwalder S, Vlegels N, Gesierich B, Araque Caballero MÁ, Weaver NA, Franzmeier N, Georgakis MK, Konieczny MJ, Koek HL, Karch CM, Graff-Radford NR, Salloway S, Oh H, Allegri RF, Chhatwal JP, Jessen F, Düzel E, Dobisch L, Metzger C, Peters O, Incesoy EI, Priller J, Spruth EJ, Schneider A, Fließbach K, Buerger K, Janowitz D, Teipel SJ, Kilimann I, Laske C, Buchmann M, Heneka MT, Brosseron F, Spottke A, Roy N, Ertl-Wagner B, Scheffler K, Seo SW, Kim Y, Na DL, Kim HJ, Jang H, Ewers M, Levin J, Schmidt R, Pasternak O,

Dichgans M, Biessels GJ, Duering M. Small vessel disease more than Alzheimer's disease determines diffusion MRI alterations in memory clinic patients. Alzheimer's & dementia: the journal of the Alzheimer's Association. 2020 November;16(11):1504-1514. PubMed PMID: 32808747; PubMed Central PMCID: PMC8102202; DOI: 10.1002/alz.12150

78. Montal V, Vilaplana E, Pegueroles J, Bejanin A, Alcolea D, Carmona-Iragui M, Clarimón J, Levin J, Cruchaga C, Graff-Radford NR, Noble JM, Lee JH, Allegri R, Karch CM, Laske C, Schofield PR, Salloway S, Ances B, Benzinger T, McDale E, Bateman R, Blesa R, Sánchez-Valle R, Lleó A, Fortea J. Biphasic cortical macro- and microstructural changes in autosomal dominant Alzheimer's disease. Alzheimers Dement. 2020 Nov 16;. doi: 10.1002/alz.12224. [Epub ahead of print] PubMed PMID: 33196147

79. Chen CD, Holden TR, Gordon BA, Franklin EE, Li Y, Coble DW, Luo H, Bateman RJ, Ances BM, Perrin RJ, Benzinger TLS, Cairns NJ, Morris JC. Ante- and postmortem tau in autosomal dominant and late-onset Alzheimer's disease. Ann Clin Transl Neurol. 2020 Nov 5;. doi: 10.1002/acn3.51237. [Epub ahead of print] PubMed PMID: 33150749; PubMed Central PMCID: PMC7732239

80. Gonneaud J, Bedetti C, Pichet Binette A, Benzinger TLS, Morris JC, Bateman RJ, Poirier J, Breitner JCS, Villeneuve S. Association of education with Aβ burden in preclinical familial and sporadic Alzheimer disease. Neurology. 2020 Sep 15;95(11):e1554-e1564. doi: 10.1212/WNL.0000000000010314. Epub 2020 Aug 5. PubMed PMID: 32759192; PubMed Central PMCID: PMC7713743

81. Vermunt L, Dicks E, Wang G, Dincer A, Flores S, Keefe SJ, Berman SB, Cash DM, Chhatwal JP, Cruchaga C, Fox NC, Ghetti B, Graff-Radford NR, Hassenstab J, Karch CM, Laske C, Levin J, Masters CL, McDade E, Mori H, Morris JC, Noble JM, Perrin RJ, Schofield PR, Xiong C, Scheltens P, Visser PJ, Bateman RJ, Benzinger TLS, Tijms BM, Gordon BA. Single-subject grey matter network trajectories over the disease course of autosomal dominant Alzheimer's disease. Brain Commun. 2020;2(2):fcaa102. doi: 10.1093/braincomms/fcaa102. eCollection 2020. PubMed PMID: 32954344; PubMed Central PMCID: PMC7475695

82. Hsu S, Pimenova AA, Hayes K, Villa JA, Rosene MJ, Jere M, Goate AM, Karch CM. Systematic validation of variants of unknown significance in APP, PSEN1 and PSEN2. Neurobiol Dis. 2020 Jun;139:104817. doi: 10.1016/j.nbd.2020.104817. Epub 2020 Feb 19. PubMed PMID: 32087291; PubMed Central PMCID: PMC7236786

83. Castillo-Barnes D, Su L, Ramírez J, Salas-Gonzalez D, Martinez-Murcia FJ, Illan IA, Segovia F, Ortiz A, Cruchaga C, Farlow MR, Xiong C, Graff-Radford NR, Schofield PR, Masters CL, Salloway S, Jucker M, Mori H, Levin J, Gorriz JM. Autosomal Dominantly Inherited Alzheimer Disease: Analysis of genetic subgroups by Machine Learning. Inf Fusion. 2020 Jun;58:153-167. doi: 10.1016/j.inffus.2020.01.001. Epub 2020 Jan 7. PubMed PMID: 32284705; PubMed Central PMCID: PMC7153760

84. Gordon BA. Neurofilaments in disease: what do we know?. Curr Opin Neurobiol. 2020 Apr;61:105-115. doi: 10.1016/j.conb.2020.02.001. Epub 2020 Mar 6. Review. PubMed PMID: 32151970; PubMed Central PMCID: PMC7198337

85. Barthélemy NR, Li Y, Joseph-Mathurin N, Gordon BA, Hassenstab J, Benzinger TLS, Buckles V, Fagan AM, Perrin RJ, Goate AM, Morris JC, Karch CM, Xiong C, Allegri R, Mendez PC, Berman SB, Ikeuchi T, Mori H, Shimada H, Shoji M, Suzuki K, Noble J, Farlow M, Chhatwal J, Graff-Radford NR, Salloway S, Schofield PR, Masters CL, Martins RN, O'Connor A, Fox NC, Levin J, Jucker M, Gabelle A, Lehmann S, Sato C, Bateman RJ, McDade E. A soluble phosphorylated tau signature links tau, amyloid and the evolution of stages of dominantly inherited Alzheimer's disease. Nat Med. 2020 Mar;26(3):398-407. doi: 10.1038/s41591-020-0781-z. Epub 2020 Mar 11. PubMed PMID: 32161412; PubMed Central PMCID: PMC7309367

86. Franzmeier N, Koutsouleris N, Benzinger T, Goate A, Karch CM, Fagan AM, McDade E, Duering M, Dichgans M, Levin J, Gordon BA, Lim YY, Masters CL, Rossor M, Fox NC, O'Connor A, Chhatwal J, Salloway S, Danek A, Hassenstab J, Schofield PR, Morris JC, Bateman RJ, Ewers M. Predicting sporadic Alzheimer's disease progression via inherited Alzheimer's disease-informed machine-learning. Alzheimers Dement. 2020 Mar;16(3):501-511. doi: 10.1002/alz.12032. Epub 2020 Feb 11. PubMed PMID: 32043733; PubMed Central PMCID: PMC7222030

87. Pichet Binette A, Vachon-Presseau É, Morris J, Bateman R, Benzinger T, Collins DL, Poirier J, Breitner JCS, Villeneuve S. Amyloid and Tau Pathology Associations With Personality Traits, Neuropsychiatric Symptoms, and Cognitive Lifestyle in the Preclinical Phases of Sporadic and Autosomal Dominant Alzheimer's Disease. Biol Psychiatry. 2020 Feb 6;. doi: 10.1016/j.biopsych.2020.01.023. [Epub ahead of print] PubMed PMID: 32228870; PubMed Central PMCID: PMC7415608

88. Day GS, Rappai T, Sathyan S, Morris JC. Deciphering the factors that influence participation in studies requiring serial lumbar punctures. Alzheimers Dement (Amst). 2020;12(1):e12003. doi: 10.1002/dad2.12003. eCollection 2020. PubMed PMID: 32211499; PubMed Central PMCID: PMC7085282

89. Aschenbrenner AJ, James BD, McDade E, Wang G, Lim YY, Benzinger TLS, Cruchaga C, Goate A, Xiong C, Perrin R, Buckles V, Allegri R, Berman SB, Chhatwal JP, Fagan A, Farlow M, O'Connor A, Ghetti B, Graff-Radford N, Goldman J, Gräber S, Karch CM, Lee JH, Levin J, Martins RN, Masters C, Mori H, Noble J, Salloway S, Schofield P, Morris JC, Bateman RJ, Hassenstab J. Awareness of genetic risk in the Dominantly Inherited Alzheimer Network (DIAN). Alzheimers Dement. 2020 Jan;16(1):219-228. doi: 10.1002/alz.12010. PubMed PMID: 31914221; PubMed Central PMCID: PMC7206736

90. Ng KP, Richard-Devantoy S, Bertrand JA, Jiang L, Pascoal TA, Mathotaarachchi S, Therriault J, Yatawara C, Kandiah N, Greenwood CMT, Rosa-Neto P, Gauthier S. Suicidal ideation is common in autosomal dominant Alzheimer's disease at-risk persons. Int J Geriatr Psychiatry. 2020 Jan;35(1):60-68. doi: 10.1002/gps.5215. Epub 2019 Oct 22. PubMed PMID: 31642105; PubMed Central PMCID: PMC7232741

91. Lessov-Schlaggar CN, Del Rosario OL, Morris JC, Ances BM, Schlaggar BL, Constantino JN. Adaptation of the Clinical Dementia Rating Scale for adults with Down syndrome. J Neurodev Disord. 2019 Dec 16;11(1):39. doi: 10.1186/s11689-019-9300-2. PubMed PMID: 31842726; PubMed Central PMCID: PMC6912998

92. Maserejian N, Bian S, Wang W, Jaeger J, Syrjanen JA, Aakre J, Jack CR Jr, Mielke MM, Gao F. Practical algorithms for amyloid β probability in subjective or mild cognitive impairment. Alzheimers Dement (Amst). 2019 Dec;11:180. doi: 10.1016/j.dadm.2019.09.001. eCollection 2019 Dec. PubMed PMID: 31700988; PubMed Central PMCID: PMC6827360

93. Su Y, Flores S, Wang G, Hornbeck RC, Speidel B, Joseph-Mathurin N, Vlassenko AG, Gordon BA, Koeppe RA, Klunk WE, Jack CR Jr, Farlow MR, Salloway S, Snider BJ, Berman SB, Roberson ED, Brosch J, Jimenez-Velazques I, van Dyck CH, Galasko D, Yuan SH, Jayadev S, Honig LS, Gauthier S, Hsiung GR, Masellis M, Brooks WS, Fulham M, Clarnette R, Masters CL, Wallon D, Hannequin D, Dubois B, Pariente J, Sanchez-Valle R, Mummery C, Ringman JM, Bottlaender M, Klein G, Milosavljevic-Ristic S, McDade E, Xiong C, Morris JC, Bateman RJ, Benzinger TLS. Comparison of Pittsburgh compound B and florbetapir in cross-sectional and longitudinal studies. Alzheimers Dement (Amst). 2019 Dec;11:180-190. doi: 10.1016/j.dadm.2018.12.008. eCollection 2019 Dec. PubMed PMID: 30847382; PubMed Central PMCID: PMC6389727

94. Gallagher M, Okonkwo OC, Resnick SM, Jagust WJ, Benzinger TLS, Rapp PR. What are the threats to successful brain and cognitive aging?. Neurobiol Aging. 2019 Nov;83:130-134. doi: 10.1016/j.neurobiolaging.2019.04.016. PubMed PMID: 31732016; PubMed Central PMCID: PMC6859944

95. Dube U, Del-Aguila JL, Li Z, Budde JP, Jiang S, Hsu S, Ibanez L, Fernandez MV, Farias F, Norton J, Gentsch J, Wang F, Salloway S, Masters CL, Lee JH, Graff-Radford NR, Chhatwal JP, Bateman RJ, Morris JC, Karch CM, Harari O, Cruchaga C. An atlas of cortical circular RNA expression in Alzheimer disease brains demonstrates clinical and pathological associations. Nat Neurosci. 2019 Nov;22(11):1903-1912. doi: 10.1038/s41593-019-0501-5. Epub 2019 Oct 7. PubMed PMID: 31591557; PubMed Central PMCID: PMC6858549

96. Xiong C, Luo J, Agboola F, Li Y, Albert M, Johnson SC, Koscik RL, Masters CL, Soldan A, Villemagne VL, Li QX, McDade EM, Fagan AM, Massoumzadeh P, Benzinger T, Hassenstab J, Bateman RJ, Morris JC. A harmonized longitudinal biomarkers and cognition database for assessing the natural history of preclinical Alzheimer's disease from young adulthood and for designing prevention trials. Alzheimers Dement. 2019 Nov;15(11):1448-1457. doi: 10.1016/j.jalz.2019.06.4955. Epub 2019 Sep 7. PubMed PMID: 31506247; PubMed Central PMCID: PMC6874758

97. Carpenter CR, McFarland F, Avidan M, Berger M, Inouye SK, Karlawish J, Lin FR, Marcantonio E, Morris JC, Reuben DB, Shah RC, Whitson HE, Asthana S, Verghese J. Impact of Cognitive Impairment Across Specialties: Summary of a Report From the U13 Conference Series. J Am Geriatr Soc. 2019 Oct;67(10):2011-2017. doi: 10.1111/jgs.16093. Epub 2019 Aug 22. PubMed PMID: 31436318; PubMed Central PMCID: PMC6800784

98. Schultz AP, Kloet RW, Sohrabi HR, van der Weerd L, van Rooden S, Wermer MJH, Moursel LG, Yaqub M, van Berckel BNM, Chatterjee P, Gardener SL, Taddei K, Fagan AM, Benzinger TL, Morris JC, Sperling R, Johnson K, Bateman RJ, Gurol ME, van Buchem MA, Martins R, Chhatwal JP, Greenberg SM. Amyloid imaging of dutch-type hereditary cerebral amyloid angiopathy carriers. Ann Neurol. 2019 Oct;86(4):616-625. doi: 10.1002/ana.25560. Epub 2019 Aug 12. PubMed PMID: 31361916; PubMed Central PMCID: PMC6876775

99. Deming Y, Filipello F, Cignarella F, Cantoni C, Hsu S, Mikesell R, Li Z, Del-Aguila JL, Dube U, Farias FG, Bradley J, Budde J, Ibanez L, Fernandez MV, Blennow K, Zetterberg H, Heslegrave A, Johansson PM, Svensson J, Nellgård B, Lleo A, Alcolea D, Clarimon J, Rami L, Molinuevo JL, Suárez-Calvet M, Morenas-Rodríguez E, Kleinberger G, Ewers M, Harari O, Haass C, Brett TJ, Benitez BA, Karch CM, Piccio L, Cruchaga C. The *MS4A* gene cluster is a key modulator of soluble TREM2 and Alzheimer's disease risk. Sci Transl Med. 2019 Aug 14;11(505). doi: 10.1126/scitranslmed.aau2291. PubMed PMID: 31413141; PubMed Central PMCID: PMC6697053

100. Navid J, Day GS, Strain J, Perrin RJ, Bucelli RC, Dincer A, Wisch JK, Soleimani-Meigooni D, Morris JC, Benzinger TLS, Ances BM. Structural signature of sporadic Creutzfeldt-Jakob disease. Eur J Neurol. 2019 Aug;26(8):1037-1043. doi: 10.1111/ene.13930. Epub 2019 Mar 25. PubMed PMID: 30735286; PubMed Central PMCID: PMC6615963

101. Buckley RF, Mormino EC, Chhatwal J, Schultz AP, Rabin JS, Rentz DM, Acar D, Properzi MJ, Dumurgier J, Jacobs H, Gomez-Isla T, Johnson KA, Sperling RA, Hanseeuw BJ. Associations between baseline amyloid, sex, and APOE on subsequent tau accumulation in cerebrospinal fluid. Neurobiol Aging. 2019 Jun;78:178-185. doi: 10.1016/j.neurobiolaging.2019.02.019. Epub 2019 Mar 7. PubMed PMID: 30947113; PubMed Central PMCID: PMC6545139

102. Vöglein J, Paumier K, Jucker M, Preische O, McDade E, Hassenstab J, Benzinger TL, Noble JM, Berman SB, Graff-Radford NR, Ghetti B, Farlow MR, Chhatwal J, Salloway S, Xiong C, Karch CM, Cairns N, Mori H,

Schofield PR, Masters CL, Goate A, Buckles V, Fox N, Rossor M, Chrem P, Allegri R, Ringman JM, Höglinger G, Steiner H, Dieterich M, Haass C, Laske C, Morris JC, Bateman RJ, Danek A, Levin J. Clinical, pathophysiological and genetic features of motor symptoms in autosomal dominant Alzheimer's disease. Brain. 2019 May 1;142(5):1429-1440. doi: 10.1093/brain/awz050. PubMed PMID: 30897203; PubMed Central PMCID: PMC6735903

103. Schindler SE, Li Y, Todd KW, Herries EM, Henson RL, Gray JD, Wang G, Graham DL, Shaw LM, Trojanowski JQ, Hassenstab JJ, Benzinger TLS, Cruchaga C, Jucker M, Levin J, Chhatwal JP, Noble JM, Ringman JM, Graff-Radford NR, Holtzman DM, Ladenson JH, Morris JC, Bateman RJ, Xiong C, Fagan AM. Emerging cerebrospinal fluid biomarkers in autosomal dominant Alzheimer's disease. Alzheimers Dement. 2019 May;15(5):655-665. doi: 10.1016/j.jalz.2018.12.019. Epub 2019 Mar 4. PubMed PMID: 30846386; PubMed Central PMCID: PMC6511459

104. Gordon BA, Blazey TM, Christensen J, Dincer A, Flores S, Keefe S, Chen C, Su Y, McDade EM, Wang G, Li Y, Hassenstab J, Aschenbrenner A, Hornbeck R, Jack CR, Ances BM, Berman SB, Brosch JR, Galasko D, Gauthier S, Lah JJ, Masellis M, van Dyck CH, Mintun MA, Klein G, Ristic S, Cairns NJ, Marcus DS, Xiong C, Holtzman DM, Raichle ME, Morris JC, Bateman RJ, Benzinger TLS. Tau PET in autosomal dominant Alzheimer's disease: relationship with cognition, dementia and other biomarkers. Brain. 2019 Apr 1;142(4):1063-1076. doi: 10.1093/brain/awz019. PubMed PMID: 30753379; PubMed Central PMCID: PMC6439328

105. Han JY, Besser LM, Xiong C, Kukull WA, Morris JC. Cholinesterase Inhibitors May Not Benefit Mild Cognitive Impairment and Mild Alzheimer Disease Dementia. Alzheimer Dis Assoc Disord. 2019 Apr-Jun;33(2):87-94. doi: 10.1097/WAD.0000000000000291. PubMed PMID: 30633043; PubMed Central PMCID: PMC6542289

106. Wang G, Coble D, McDade EM, Hassenstab J, Fagan AM, Benzinger TLS, Bateman RJ, Morris JC, Xiong C. Staging biomarkers in preclinical autosomal dominant Alzheimer's disease by estimated years to symptom onset. Alzheimers Dement. 2019 Apr;15(4):506-514. doi: 10.1016/j.jalz.2018.12.008. Epub 2019 Feb 15. PubMed PMID: 30773445; PubMed Central PMCID: PMC6461496

107. Vöglein J, Noachtar S, McDade E, Quaid KA, Salloway S, Ghetti B, Noble J, Berman S, Chhatwal J, Mori H, Fox N, Allegri R, Masters CL, Buckles V, Ringman JM, Rossor M, Schofield PR, Sperling R, Jucker M, Laske C, Paumier K, Morris JC, Bateman RJ, Levin J, Danek A. Seizures as an early symptom of autosomal dominant Alzheimer's disease. Neurobiol Aging. 2019 Apr;76:18-23. doi: 10.1016/j.neurobiolaging.2018.11.022. Epub 2018 Dec 5. PubMed PMID: 30616208; PubMed Central PMCID: PMC6572755

108. Wang Q, Wang Y, Liu J, Sutphen CL, Cruchaga C, Blazey T, Gordon BA, Su Y, Chen C, Shimony JS, Ances BM, Cairns NJ, Fagan AM, Morris JC, Benzinger TLS. Quantification of white matter cellularity and damage in preclinical and early symptomatic Alzheimer's disease. Neuroimage Clin. 2019;22:101767. doi: 10.1016/j.nicl.2019.101767. Epub 2019 Mar 13. PubMed PMID: 30901713; PubMed Central PMCID: PMC6428957

109. Bussy A, Snider BJ, Coble D, Xiong C, Fagan AM, Cruchaga C, Benzinger TLS, Gordon BA, Hassenstab J, Bateman RJ, Morris JC. Effect of apolipoprotein E4 on clinical, neuroimaging, and biomarker measures in noncarrier participants in the Dominantly Inherited Alzheimer Network. Neurobiol Aging. 2019 Mar;75:42-50. doi: 10.1016/j.neurobiolaging.2018.10.011. Epub 2018 Oct 13. PubMed PMID: 30530186; PubMed Central PMCID: PMC6385602

110. Preische O, Schultz SA, Apel A, Kuhle J, Kaeser SA, Barro C, Gräber S, Kuder-Buletta E, LaFougere C, Laske C, Vöglein J, Levin J, Masters CL, Martins R, Schofield PR, Rossor MN, Graff-Radford NR, Salloway S, Ghetti B, Ringman JM, Noble JM, Chhatwal J, Goate AM, Benzinger TLS, Morris JC, Bateman RJ, Wang G, Fagan AM, McDade EM, Gordon BA, Jucker M. Serum neurofilament dynamics predicts neurodegeneration and clinical progression in presymptomatic Alzheimer's disease. Nat Med. 2019 Feb;25(2):277-283. doi: 10.1038/s41591-018-0304-3. Epub 2019 Jan 21. PubMed PMID: 30664784; PubMed Central PMCID: PMC636700

111. Properzi MJ, Buckley RF, Chhatwal JP, Donohue MC, Lois C, Mormino EC, Johnson KA, Sperling RA, Schultz AP. Nonlinear Distributional Mapping (NoDiM) for harmonization across amyloid-PET radiotracers. Neuroimage. 2019 Feb 1;186:446-454. doi: 10.1016/j.neuroimage.2018.11.019. Epub 2018 Nov 17. PubMed PMID: 30458305; PubMed Central PMCID: PMC6338495

112. Toedebusch CD, McLeland JS, Schaibley CM, Banks IR, Boyd J, Morris JC, Holtzman DM, Lucey BP. Multi-Modal Home Sleep Monitoring in Older Adults. J Vis Exp. 2019 Jan 26;(143). doi: 10.3791/58823. PubMed PMID: 30741255; PubMed Central PMCID: PMC6377867

113. Day GS, Gordon BA, Perrin RJ, Ances BM. Author response: In vivo [^{18}F]-AV-1451 tau-PET imaging in sporadic Creutzfeldt-Jakob disease. Neurology. 2019 Jan 15;92(3):150. doi: 10.1212/WNL.0000000000006771. PubMed PMID: 30643031

114. Lucey BP, McCullough A, Landsness EC, Toedebusch CD, McLeland JS, Zaza AM, Fagan AM, McCue L, Xiong C, Morris JC, Benzinger TLS, Holtzman DM. Reduced non-rapid eye movement sleep is associated with tau pathology in early Alzheimer's disease. Sci Transl Med. 2019 Jan 9;11(474). doi: 10.1126/scitranslmed.aau6550. PubMed PMID: 30626715; PubMed Central PMCID: PMC6342564

115. Roe CM, Stout SH, Rajasekar G, Ances BM, Jones JM, Head D, Benzinger TLS, Williams MM, Davis JD, Ott BR, Warren DK, Babulal GM. A 2.5-Year Longitudinal Assessment of Naturalistic Driving in Preclinical Alzheimer's Disease. J Alzheimers Dis. 2019;68(4):1625-1633. doi: 10.3233/JAD-181242. PubMed PMID: 30958365; PubMed Central PMCID: PMC6488385

116. Barroeta-Espar I, Weinstock LD, Perez-Nievas BG, Meltzer AC, Siao Tick Chong M, Amaral AC, Murray ME, Moulder KL, Morris JC, Cairns NJ, Parisi JE, Lowe VJ, Petersen RC, Kofler J, Ikonomovic MD, López O, Klunk WE, Mayeux RP, Frosch MP, Wood LB, Gomez-Isla T. Distinct cytokine profiles in human brains resilient to Alzheimer's pathology. Neurobiol Dis. 2019 Jan;121:327-337. doi: 10.1016/j.nbd.2018.10.009. Epub 2018 Oct 15. PubMed PMID: 30336198; PubMed Central PMCID: PMC6437670

117. Veitch DP, Weiner MW, Aisen PS, Beckett LA, Cairns NJ, Green RC, Harvey D, Jack CR Jr, Jagust W, Morris JC, Petersen RC, Saykin AJ, Shaw LM, Toga AW, Trojanowski JQ. Understanding disease progression and improving Alzheimer's disease clinical trials: Recent highlights from the Alzheimer's Disease Neuroimaging Initiative. Alzheimers Dement. 2019 Jan;15(1):106-152. doi: 10.1016/j.jalz.2018.08.005. Epub 2018 Oct 13. Review. PubMed PMID: 30321505

118. Jiang S, Wen N, Li Z, Dube U, Del Aguila J, Budde J, Martinez R, Hsu S, Fernandez MV, Cairns NJ, Harari O, Cruchaga C, Karch CM. Integrative system biology analyses of CRISPR-edited iPSC-derived neurons and human brains reveal deficiencies of presynaptic signaling in FTLD and PSP. Transl Psychiatry. 2018 Dec 13;8(1):265. doi: 10.1038/s41398-018-0319-z. PubMed PMID: 30546007; PubMed Central PMCID: PMC6293323

119. Wang G, Xiong C, McDade EM, Hassenstab J, Aschenbrenner AJ, Fagan AM, Benzinger TLS, Gordon BA, Morris JC, Li Y, Bateman RJ. Simultaneously evaluating the effect of baseline levels and longitudinal

changes in disease biomarkers on cognition in dominantly inherited Alzheimer's disease. Alzheimers Dement (N Y). 2018;4:669-676. doi: 10.1016/j.trci.2018.10.009. eCollection 2018. PubMed PMID: 30569014; PubMed Central PMCID: PMC6288312

120. Suárez-Calvet M, Capell A, Araque Caballero MÁ, Morenas-Rodríguez E, Fellerer K, Franzmeier N, Kleinberger G, Eren E, Deming Y, Piccio L, Karch CM, Cruchaga C, Paumier K, Bateman RJ, Fagan AM, Morris JC, Levin J, Danek A, Jucker M, Masters CL, Rossor MN, Ringman JM, Shaw LM, Trojanowski JQ, Weiner M, Ewers M, Haass C. CSF progranulin increases in the course of Alzheimer's disease and is associated with sTREM2, neurodegeneration and cognitive decline. EMBO Mol Med. 2018 Dec;10(12). doi: 10.15252/emmm.201809712. PubMed PMID: 30482868; PubMed Central PMCID: PMC6284390

121. Twohig D, Rodriguez-Vieitez E, Sando SB, Berge G, Lauridsen C, Møller I, Grøntvedt GR, Bråthen G, Patra K, Bu G, Benzinger TLS, Karch CM, Fagan A, Morris JC, Bateman RJ, Nordberg A, White LR, Nielsen HM. The relevance of cerebrospinal fluid α-synuclein levels to sporadic and familial Alzheimer's disease. Acta Neuropathol Commun. 2018 Nov 26;6(1):130. doi: 10.1186/s40478-018-0624-z. PubMed PMID: 30477568; PubMed Central PMCID: PMC6260771

122. Müller S, Preische O, Sohrabi HR, Gräber S, Jucker M, Ringman JM, Martins RN, McDade E, Schofield PR, Ghetti B, Rossor M, Fox NN, Graff-Radford NR, Levin J, Danek A, Vöglein J, Salloway S, Xiong C, Benzinger T, Buckles V, Masters CL, Sperling R, Bateman RJ, Morris JC, Laske C. Relationship between physical activity, cognition, and Alzheimer pathology in autosomal dominant Alzheimer's disease. Alzheimers Dement. 2018 Nov;14(11):1427-1437. doi: 10.1016/j.jalz.2018.06.3059. Epub 2018 Sep 25. PubMed PMID: 30266303; PubMed Central PMCID: PMC6322213

123. Schindler SE, Gray JD, Gordon BA, Xiong C, Batrla-Utermann R, Quan M, Wahl S, Benzinger TLS, Holtzman DM, Morris JC, Fagan AM. Cerebrospinal fluid biomarkers measured by Elecsys assays compared to amyloid imaging. Alzheimers Dement. 2018 Nov;14(11):1460-1469. doi: 10.1016/j.jalz.2018.01.013. Epub 2018 Mar 2. PubMed PMID: 29501462; PubMed Central PMCID: PMC6119652

124. McDade E, Wang G, Gordon BA, Hassenstab J, Benzinger TLS, Buckles V, Fagan AM, Holtzman DM, Cairns NJ, Goate AM, Marcus DS, Morris JC, Paumier K, Xiong C, Allegri R, Berman SB, Klunk W, Noble J, Ringman J, Ghetti B, Farlow M, Sperling RA, Chhatwal J, Salloway S, Graff-Radford NR, Schofield PR, Masters C, Rossor MN, Fox NC, Levin J, Jucker M, Bateman RJ. Longitudinal cognitive and biomarker changes in dominantly inherited Alzheimer disease. Neurology. 2018 Oct 2;91(14):e1295-e1306. doi: 10.1212/WNL.0000000000006277. Epub 2018 Sep 14. PubMed PMID: 30217935; PubMed Central PMCID: PMC6177272

125. Besser L, Kukull W, Knopman DS, Chui H, Galasko D, Weintraub S, Jicha G, Carlsson C, Burns J, Quinn J, Sweet RA, Rascovsky K, Teylan M, Beekly D, Thomas G, Bollenbeck M, Monsell S, Mock C, Zhou XH, Thomas N, Robichaud E, Dean M, Hubbard J, Jacka M, Schwabe-Fry K, Wu J, Phelps C, Morris JC. Version 3 of the National Alzheimer's Coordinating Center's Uniform Data Set. Alzheimer Dis Assoc Disord. 2018 Oct-Dec;32(4):351-358. doi: 10.1097/WAD.0000000000000279. Review. PubMed PMID: 30376508; PubMed Central PMCID: PMC6249084

126. Araque Caballero MÁ, Suárez-Calvet M, Duering M, Franzmeier N, Benzinger T, Fagan AM, Bateman RJ, Jack CR, Levin J, Dichgans M, Jucker M, Karch C, Masters CL, Morris JC, Weiner M, Rossor M, Fox NC, Lee JH, Salloway S, Danek A, Goate A, Yakushev I, Hassenstab J, Schofield PR, Haass C, Ewers M. White matter diffusion alterations precede symptom onset in autosomal dominant Alzheimer's disease. Brain. 2018 Oct 1;141(10):3065-3080. doi: 10.1093/brain/awy229. PubMed PMID: 30239611; PubMed Central PMCID: PMC6158739

127. Day GS, Musiek ES, Morris JC. Rapidly Progressive Dementia in the Outpatient Clinic: More Than Prions. Alzheimer Dis Assoc Disord. 2018 Oct-Dec;32(4):291-297. doi: 10.1097/WAD.0000000000000276. PubMed PMID: 30222606; PubMed Central PMCID: PMC6249048

128. Joseph-Mathurin N, Su Y, Blazey TM, Jasielec M, Vlassenko A, Friedrichsen K, Gordon BA, Hornbeck RC, Cash L, Ances BM, Veale T, Cash DM, Brickman AM, Buckles V, Cairns NJ, Cruchaga C, Goate A, Jack CR Jr, Karch C, Klunk W, Koeppe RA, Marcus DS, Mayeux R, McDade E, Noble JM, Ringman J, Saykin AJ, Thompson PM, Xiong C, Morris JC, Bateman RJ, Benzinger TLS. Utility of perfusion PET measures to assess neuronal injury in Alzheimer's disease. Alzheimers Dement (Amst). 2018;10:669-677. doi: 10.1016/j.dadm.2018.08.012. eCollection 2018. PubMed PMID: 30417072; PubMed Central PMCID: PMC6215983

129. Wang G, Berry S, Xiong C, Hassenstab J, Quintana M, McDade EM, Delmar P, Vestrucci M, Sethuraman G, Bateman RJ. A novel cognitive disease progression model for clinical trials in autosomal-dominant Alzheimer's disease. Stat Med. 2018 Sep 20;37(21):3047-3055. doi: 10.1002/sim.7811. Epub 2018 May 14. PubMed PMID: 29761523; PubMed Central PMCID: PMC6105413

130. Lim YY, Hassenstab J, Goate A, Fagan AM, Benzinger TLS, Cruchaga C, McDade E, Chhatwal J, Levin J, Farlow MR, Graff-Radford NR, Laske C, Masters CL, Salloway S, Schofield P, Morris JC, Maruff P, Bateman RJ. Effect of BDNFVal66Met on disease markers in dominantly inherited Alzheimer's disease. Ann Neurol. 2018 Sep;84(3):424-435. doi: 10.1002/ana.25299. Epub 2018 Aug 25. PubMed PMID: 30014553; PubMed Central PMCID: PMC6153076

131. Buckley RF, Mormino EC, Amariglio RE, Properzi MJ, Rabin JS, Lim YY, Papp KV, Jacobs HIL, Burnham S, Hanseeuw BJ, Doré V, Dobson A, Masters CL, Waller M, Rowe CC, Maruff P, Donohue MC, Rentz DM, Kirn D, Hedden T, Chhatwal J, Schultz AP, Johnson KA, Villemagne VL, Sperling RA. Sex, amyloid, and APOE ε4 and risk of cognitive decline in preclinical Alzheimer's disease: Findings from three well-characterized cohorts. Alzheimers Dement. 2018 Sep;14(9):1193-1203. doi: 10.1016/j.jalz.2018.04.010. Epub 2018 May 24. PubMed PMID: 29803541; PubMed Central PMCID: PMC6131023

132. Karch CM, Hernández D, Wang JC, Marsh J, Hewitt AW, Hsu S, Norton J, Levitch D, Donahue T, Sigurdson W, Ghetti B, Farlow M, Chhatwal J, Berman S, Cruchaga C, Morris JC, Bateman RJ, Pébay A, Goate AM. Human fibroblast and stem cell resource from the Dominantly Inherited Alzheimer Network. Alzheimers Res Ther. 2018 Jul 25;10(1):69. doi: 10.1186/s13195-018-0400-0. PubMed PMID: 30045758; PubMed Central PMCID: PMC6060509

133. Hsu S, Gordon BA, Hornbeck R, Norton JB, Levitch D, Louden A, Ziegemeier E, Laforce R Jr, Chhatwal J, Day GS, McDade E, Morris JC, Fagan AM, Benzinger TLS, Goate AM, Cruchaga C, Bateman RJ, Karch CM. Discovery and validation of autosomal dominant Alzheimer's disease mutations. Alzheimers Res Ther. 2018 Jul 18;10(1):67. doi: 10.1186/s13195-018-0392-9. PubMed PMID: 30021643; PubMed Central PMCID: PMC6052673

134. Gabel M, Gooblar J, Roe CM, Selsor NJ, Morris JC. Political Ideology, Confidence in Science, and Participation in Alzheimer Disease Research Studies. Alzheimer Dis Assoc Disord. 2018 Jul-Sep;32(3):179-184. doi: 10.1097/WAD.0000000000000244. PubMed PMID: 29351092; PubMed Central PMCID: PMC6051930

135. Vlassenko AG, Gordon BA, Goyal MS, Su Y, Blazey TM, Durbin TJ, Couture LE, Christensen JJ, Jafri H, Morris JC, Raichle ME, Benzinger TL. Aerobic glycolysis and tau deposition in preclinical Alzheimer's disease. Neurobiol Aging. 2018 Jul;67:95-98. doi: 10.1016/j.neurobiolaging.2018.03.014. Epub 2018 Mar 20. PubMed PMID: 29655050; PubMed Central PMCID: PMC5955846

136. Li Z, Del-Aguila JL, Dube U, Budde J, Martinez R, Black K, Xiao Q, Cairns NJ, Dougherty JD, Lee JM, Morris JC, Bateman RJ, Karch CM, Cruchaga C, Harari O. Genetic variants associated with Alzheimer's disease confer different cerebral cortex cell-type population structure. Genome Med. 2018 Jun 8;10(1):43. doi: 10.1186/s13073-018-0551-4. PubMed PMID: 29880032; PubMed Central PMCID: PMC5992755

137. Carvalho DZ, St Louis EK, Knopman DS, Boeve BF, Lowe VJ, Roberts RO, Mielke MM, Przybelski SA, Machulda MM, Petersen RC, Jack CR Jr, Vemuri P. Association of Excessive Daytime Sleepiness With Longitudinal β-Amyloid Accumulation in Elderly Persons Without Dementia. JAMA Neurol. 2018 Jun 1;75(6):672-680. doi: 10.1001/jamaneurol.2018.0049. PubMed PMID: 29532057; PubMed Central PMCID: PMC5885188

138. Lee S, Zimmerman ME, Narkhede A, Nasrabady SE, Tosto G, Meier IB, Benzinger TLS, Marcus DS, Fagan AM, Fox NC, Cairns NJ, Holtzman DM, Buckles V, Ghetti B, McDade E, Martins RN, Saykin AJ, Masters CL, Ringman JM, Förster S, Schofield PR, Sperling RA, Johnson KA, Chhatwal JP, Salloway S, Correia S, Jack CR Jr, Weiner M, Bateman RJ, Morris JC, Mayeux R, Brickman AM. White matter hyperintensities and the mediating role of cerebral amyloid angiopathy in dominantly-inherited Alzheimer's disease. PLoS One. 2018;13(5):e0195838. doi: 10.1371/journal.pone.0195838. eCollection 2018. PubMed PMID: 29742105; PubMed Central PMCID: PMC5942789

139. Oxtoby NP, Young AL, Cash DM, Benzinger TLS, Fagan AM, Morris JC, Bateman RJ, Fox NC, Schott JM, Alexander DC. Data-driven models of dominantly-inherited Alzheimer's disease progression. Brain. 2018 May 1;141(5):1529-1544. doi: 10.1093/brain/awy050. PubMed PMID: 29579160; PubMed Central PMCID: PMC5920320

140. Chhatwal JP, Schultz AP, Johnson KA, Hedden T, Jaimes S, Benzinger TLS, Jack C Jr, Ances BM, Ringman JM, Marcus DS, Ghetti B, Farlow MR, Danek A, Levin J, Yakushev I, Laske C, Koeppe RA, Galasko DR, Xiong C, Masters CL, Schofield PR, Kinnunen KM, Salloway S, Martins RN, McDade E, Cairns NJ, Buckles VD, Morris JC, Bateman R, Sperling RA. Preferential degradation of cognitive networks differentiates Alzheimer's disease from ageing. Brain. 2018 May 1;141(5):1486-1500. doi: 10.1093/brain/awy053. PubMed PMID: 29522171; PubMed Central PMCID: PMC5917745

141. Villeneuve S, Vogel JW, Gonneaud J, Pichet Binette A, Rosa-Neto P, Gauthier S, Bateman RJ, Fagan AM, Morris JC, Benzinger TLS, Johnson SC, Breitner JCS, Poirier J. Proximity to Parental Symptom Onset and Amyloid-β Burden in Sporadic Alzheimer Disease. JAMA Neurol. 2018 May 1;75(5):608-619. doi: 10.1001/jamaneurol.2017.5135. PubMed PMID: 29482212; PubMed Central PMCID: PMC5885216

142. Petok JR, Myers CE, Pa J, Hobel Z, Wharton DM, Medina LD, Casado M, Coppola G, Gluck MA, Ringman JM. Impairment of memory generalization in preclinical autosomal dominant Alzheimer's disease mutation carriers. Neurobiol Aging. 2018 May;65:149-157. doi: 10.1016/j.neurobiolaging.2018.01.022. Epub 2018 Feb 8. PubMed PMID: 29494861; PubMed Central PMCID: PMC5871602

143. Stout SH, Babulal GM, Ma C, Carr DB, Head DM, Grant EA, Williams MM, Holtzman DM, Fagan AM, Morris JC, Roe CM. Driving cessation over a 24-year period: Dementia severity and cerebrospinal fluid biomarkers. Alzheimers Dement. 2018 May;14(5):610-616. doi: 10.1016/j.jalz.2017.11.011. Epub 2018 Jan 10. PubMed PMID: 29328928; PubMed Central PMCID: PMC5938126

144. Su Y, Flores S, Hornbeck RC, Speidel B, Vlassenko AG, Gordon BA, Koeppe RA, Klunk WE, Xiong C, Morris JC, Benzinger TLS. Utilizing the Centiloid scale in cross-sectional and longitudinal PiB PET studies. Neuroimage Clin. 2018;19:406-416. doi: 10.1016/j.nicl.2018.04.022. eCollection 2018. PubMed PMID: 30035025; PubMed Central PMCID: PMC6051499

145. Allison S, Babulal GM, Stout SH, Barco PP, Carr DB, Fagan AM, Morris JC, Roe CM, Head D. Alzheimer Disease Biomarkers and Driving in Clinically Normal Older Adults: Role of Spatial Navigation Abilities. Alzheimer Dis Assoc Disord. 2018 Apr-Jun;32(2):101-106. doi: 10.1097/WAD.0000000000000257. PubMed PMID: 29578861; PubMed Central PMCID: PMC5963990

146. Franzmeier N, Düzel E, Jessen F, Buerger K, Levin J, Duering M, Dichgans M, Haass C, Suárez-Calvet M, Fagan AM, Paumier K, Benzinger T, Masters CL, Morris JC, Perneczky R, Janowitz D, Catak C, Wolfsgruber S, Wagner M, Teipel S, Kilimann I, Ramirez A, Rossor M, Jucker M, Chhatwal J, Spottke A, Boecker H, Brosseron F, Falkai P, Fliessbach K, Heneka MT, Laske C, Nestor P, Peters O, Fuentes M, Menne F, Priller J, Spruth EJ, Franke C, Schneider A, Kofler B, Westerteicher C, Speck O, Wiltfang J, Bartels C, Araque Caballero MÁ, Metzger C, Bittner D, Weiner M, Lee JH, Salloway S, Danek A, Goate A, Schofield PR, Bateman RJ, Ewers M. Left frontal hub connectivity delays cognitive impairment in autosomal-dominant and sporadic Alzheimer's disease. Brain. 2018 Apr 1;141(4):1186-1200. doi: 10.1093/brain/awy008. PubMed PMID: 29462334; PubMed Central PMCID: PMC5888938

147. Day GS, Gordon BA, Perrin RJ, Cairns NJ, Beaumont H, Schwetye K, Ferguson C, Sinha N, Bucelli R, Musiek ES, Ghoshal N, Ponisio MR, Vincent B, Mishra S, Jackson K, Morris JC, Benzinger TLS, Ances BM. In vivo [^{18}F]-AV-1451 tau-PET imaging in sporadic Creutzfeldt-Jakob disease. Neurology. 2018 Mar 6;90(10):e896-e906. doi: 10.1212/WNL.0000000000005064. Epub 2018 Feb 7. PubMed PMID: 29438042; PubMed Central PMCID: PMC5863493

148. Jacobs HIL, Hedden T, Schultz AP, Sepulcre J, Perea RD, Amariglio RE, Papp KV, Rentz DM, Sperling RA, Johnson KA. Structural tract alterations predict downstream tau accumulation in amyloid-positive older individuals. Nat Neurosci. 2018 Mar;21(3):424-431. doi: 10.1038/s41593-018-0070-z. Epub 2018 Feb 5. PubMed PMID: 29403032; PubMed Central PMCID: PMC5857215

149. Xiong C, Luo J, Chen L, Gao F, Liu J, Wang G, Bateman R, Morris JC. Estimating diagnostic accuracy for clustered ordinal diagnostic groups in the three-class case-Application to the early diagnosis of Alzheimer disease. Stat Methods Med Res. 2018 Mar;27(3):701-714. doi: 10.1177/0962280217742539. Epub 2017 Nov 28. PubMed PMID: 29182052; PubMed Central PMCID: PMC5841923

150. Owens TE, Machulda MM, Duffy JR, Strand EA, Clark HM, Boland S, Martin PR, Lowe VJ, Jack CR, Whitwell JL, Josephs KA. Patterns of Neuropsychological Dysfunction and Cortical Volume Changes in Logopenic Aphasia. J Alzheimers Dis. 2018;66(3):1015-1025. doi: 10.3233/JAD-171175. PubMed PMID: 30372673; PubMed Central PMCID: PMC6322407

151. Luo J, Weng H, Morris JC, Xiong C. Minimizing the Sample Sizes of Clinical Trials on Preclinical and Early Symptomatic Stage of Alzheimer Disease. J Prev Alzheimers Dis. 2018;5(2):110-119. doi: 10.14283/jpad.2018.16. PubMed PMID: 29616704; PubMed Central PMCID: PMC6429951

152. Martins RN, Villemagne V, Sohrabi HR, Chatterjee P, Shah TM, Verdile G, Fraser P, Taddei K, Gupta VB, Rainey-Smith SR, Hone E, Pedrini S, Lim WL, Martins I, Frost S, Gupta S, O'Bryant S, Rembach A, Ames D, Ellis K, Fuller SJ, Brown B, Gardener SL, Fernando B, Bharadwaj P, Burnham S, Laws SM, Barron AM, Goozee K, Wahjoepramono EJ, Asih PR, Doecke JD, Salvado O, Bush AI, Rowe CC, Gandy SE, Masters CL. Alzheimer's Disease: A Journey from Amyloid Peptides and Oxidative Stress, to Biomarker Technologies and Disease Prevention Strategies-Gains from AIBL and DIAN Cohort Studies. J Alzheimers Dis. 2018;62(3):965-992. doi: 10.3233/JAD-171145. Review. PubMed PMID: 29562546; PubMed Central PMCID: PMC5870031

153. Weintraub S, Besser L, Dodge HH, Teylan M, Ferris S, Goldstein FC, Giordani B, Kramer J, Loewenstein D, Marson D, Mungas D, Salmon D, Welsh-Bohmer K, Zhou XH, Shirk SD, Atri A, Kukull WA, Phelps C, Morris

JC. Version 3 of the Alzheimer Disease Centers' Neuropsychological Test Battery in the Uniform Data Set (UDS). Alzheimer Dis Assoc Disord. 2018 Jan-Mar;32(1):10-17. doi: 10.1097/WAD.0000000000000223. PubMed PMID: 29240561; PubMed Central PMCID: PMC5821520

154. Yan L, Liu CY, Wong KP, Huang SC, Mack WJ, Jann K, Coppola G, Ringman JM, Wang DJJ. Regional association of pCASL-MRI with FDG-PET and PiB-PET in people at risk for autosomal dominant Alzheimer's disease. Neuroimage Clin. 2018;17:751-760. doi: 10.1016/j.nicl.2017.12.003. eCollection 2018. PubMed PMID: 29527482; PubMed Central PMCID: PMC5842754

155. Kinnunen KM, Cash DM, Poole T, Frost C, Benzinger TLS, Ahsan RL, Leung KK, Cardoso MJ, Modat M, Malone IB, Morris JC, Bateman RJ, Marcus DS, Goate A, Salloway SP, Correia S, Sperling RA, Chhatwal JP, Mayeux RP, Brickman AM, Martins RN, Farlow MR, Ghetti B, Saykin AJ, Jack CR Jr, Schofield PR, McDade E, Weiner MW, Ringman JM, Thompson PM, Masters CL, Rowe CC, Rossor MN, Ourselin S, Fox NC. Presymptomatic atrophy in autosomal dominant Alzheimer's disease: A serial magnetic resonance imaging study. Alzheimers Dement. 2018 Jan;14(1):43-53. doi: 10.1016/j.jalz.2017.06.2268. Epub 2017 Jul 22. PubMed PMID: 28738187; PubMed Central PMCID: PMC5751893

156. Schindler SE, Sutphen CL, Teunissen C, McCue LM, Morris JC, Holtzman DM, Mulder SD, Scheltens P, Xiong C, Fagan AM. Upward drift in cerebrospinal fluid amyloid β 42 assay values for more than 10 years. Alzheimers Dement. 2018 Jan;14(1):62-70. doi: 10.1016/j.jalz.2017.06.2264. Epub 2017 Jul 12. PubMed PMID: 28710906; PubMed Central PMCID: PMC5750131

157. Brown BM, Sohrabi HR, Taddei K, Gardener SL, Rainey-Smith SR, Peiffer JJ, Xiong C, Fagan AM, Benzinger T, Buckles V, Erickson KI, Clarnette R, Shah T, Masters CL, Weiner M, Cairns N, Rossor M, Graff-Radford NR, Salloway S, Vöglein J, Laske C, Noble J, Schofield PR, Bateman RJ, Morris JC, Martins RN. Habitual exercise levels are associated with cerebral amyloid load in presymptomatic autosomal dominant Alzheimer's disease. Alzheimers Dement. 2017 Nov;13(11):1197-1206. doi: 10.1016/j.jalz.2017.03.008. Epub 2017 May 11. PubMed PMID: 28501451; PubMed Central PMCID: PMC5675772

158. Ringman JM, Casado M, Van Berlo V, Pa J, Joseph-Mathurin N, Fagan AM, Benzinger T, Bateman RJ, Morris JC. A novel PSEN1 (S230N) mutation causing early-onset Alzheimer's Disease associated with prosopagnosia, hoarding, and Parkinsonism. Neurosci Lett. 2017 Sep 14;657:11-15. doi: 10.1016/j.neulet.2017.07.046. Epub 2017 Jul 29. PubMed PMID: 28764909; PubMed Central PMCID: PMC5731478

159. Gao F, Philip Miller J, Xiong C, Luo J, Beiser JA, Chen L, Gordon MO. Estimating correlation between multivariate longitudinal data in the presence of heterogeneity. BMC Med Res Methodol. 2017 Aug 17;17(1):124. doi: 10.1186/s12874-017-0398-1. PubMed PMID: 28818061; PubMed Central PMCID: PMC5561646

160. Goyal MS, Vlassenko AG, Blazey TM, Su Y, Couture LE, Durbin TJ, Bateman RJ, Benzinger TL, Morris JC, Raichle ME. Loss of Brain Aerobic Glycolysis in Normal Human Aging. Cell Metab. 2017 Aug 1;26(2):353-360.e3. doi: 10.1016/j.cmet.2017.07.010. PubMed PMID: 28768174; PubMed Central PMCID: PMC5573225

161. Shimada H, Shoji M, Ikeuchi T, Suzuki K, Senda M, Ishii K, Matsuda H, Iwata A, Ihara R, Iwatsubo T, Mutoh K, Nakazawa E, Sekijima Y, Mori E, Ikeda M, Ikeda M, Kawakatsu S, Nakanishi A, Hashimoto M, Nunomura A, Matsubara E, Fukui M, Shirato T, Hirai K, Sakamoto M, Fujii H, Mori H. [DIAN/DIAN-J/DIAN-TU]. Brain Nerve. 2017 Jul;69(7):701-709. doi: 10.11477/mf.1416200811. PubMed PMID: 28739983

162. Weng H, Bateman R, Morris JC, Xiong C. Validity and power of minimization algorithm in longitudinal analysis of clinical trials. Biostat Epidemiol. 2017;1(1):59-77. doi: 10.1080/24709360.2017.1331822. Epub 2017 Jun 13. PubMed PMID: 29250611; PubMed Central PMCID: PMC5730087

163. Xiong C, Luo J, Morris JC, Bateman R. Linear Combinations of Multiple Outcome Measures to Improve the Power of Efficacy Analysis —Application to Clinical Trials on Early Stage Alzheimer Disease. Biostat Epidemiol. 2017;1(1):36-58. doi: 10.1080/24709360.2017.1331821. Epub 2017 Jun 2. PubMed PMID: 29546251; PubMed Central PMCID: PMC5849424

164. Ng KP, Pascoal TA, Mathotaarachchi S, Chung CO, Benedet AL, Shin M, Kang MS, Li X, Ba M, Kandiah N, Rosa-Neto P, Gauthier S. Neuropsychiatric symptoms predict hypometabolism in preclinical Alzheimer disease. Neurology. 2017 May 9;88(19):1814-1821. doi: 10.1212/WNL.0000000000003916. Epub 2017 Apr 12. PubMed PMID: 28404803; PubMed Central PMCID: PMC5419982

165. Müller S, Preische O, Sohrabi HR, Gräber S, Jucker M, Dietzsch J, Ringman JM, Martins RN, McDade E, Schofield PR, Ghetti B, Rossor M, Graff-Radford NR, Levin J, Galasko D, Quaid KA, Salloway S, Xiong C, Benzinger T, Buckles V, Masters CL, Sperling R, Bateman RJ, Morris JC, Laske C. Decreased body mass index in the preclinical stage of autosomal dominant Alzheimer's disease. Sci Rep. 2017 Apr 27;7(1):1225. doi: 10.1038/s41598-017-01327-w. PubMed PMID: 28450713; PubMed Central PMCID: PMC5430642

166. Day GS, Gordon BA, Jackson K, Christensen JJ, Rosana Ponisio M, Su Y, Ances BM, Benzinger TLS, Morris JC. Tau-PET Binding Distinguishes Patients With Early-stage Posterior Cortical Atrophy From Amnestic Alzheimer Disease Dementia. Alzheimer Dis Assoc Disord. 2017 Apr-Jun;31(2):87-93. doi: 10.1097/WAD.0000000000000196. PubMed PMID: 28394771; PubMed Central PMCID: PMC5443698

167. Su Y, Vlassenko AG, Couture LE, Benzinger TL, Snyder AZ, Derdeyn CP, Raichle ME. Quantitative hemodynamic PET imaging using image-derived arterial input function and a PET/MR hybrid scanner. J Cereb Blood Flow Metab. 2017 Apr;37(4):1435-1446. doi: 10.1177/0271678X16656200. Epub 2016 Jan 1. PubMed PMID: 27401805; PubMed Central PMCID: PMC5453463

168. Jack CR Jr, Wiste HJ, Weigand SD, Therneau TM, Lowe VJ, Knopman DS, Gunter JL, Senjem ML, Jones DT, Kantarci K, Machulda MM, Mielke MM, Roberts RO, Vemuri P, Reyes DA, Petersen RC. Defining imaging biomarker cut points for brain aging and Alzheimer's disease. Alzheimers Dement. 2017 Mar;13(3):205-216. doi: 10.1016/j.jalz.2016.08.005. Epub 2016 Sep 30. PubMed PMID: 27697430; PubMed Central PMCID: PMC5344738

169. Natelson Love M, Clark DG, Cochran JN, Den Beste KA, Geldmacher DS, Benzinger TL, Gordon BA, Morris JC, Bateman RJ, Roberson ED. Clinical, imaging, pathological, and biochemical characterization of a novel presenilin 1 mutation (N135Y) causing Alzheimer's disease. Neurobiol Aging. 2017 Jan;49:216.e7-216.e13. doi: 10.1016/j.neurobiolaging.2016.09.020. Epub 2016 Oct 3. PubMed PMID: 27793474; PubMed Central PMCID: PMC5154842

170. Bateman RJ, Benzinger TL, Berry S, Clifford DB, Duggan C, Fagan AM, Fanning K, Farlow MR, Hassenstab J, McDade EM, Mills S, Paumier K, Quintana M, Salloway SP, Santacruz A, Schneider LS, Wang G, Xiong C. The DIAN-TU Next Generation Alzheimer's prevention trial: Adaptive design and disease progression model. Alzheimers Dement. 2017 Jan;13(1):8-19. doi: 10.1016/j.jalz.2016.07.005. Epub 2016 Aug 29. PubMed PMID: 27583651; PubMed Central PMCID: PMC5218895

171. Gurney J, Olsen T, Flavin J, Ramaratnam M, Archie K, Ransford J, Herrick R, Wallace L, Cline J, Horton W, Marcus DS. The Washington University Central Neuroimaging Data Archive. Neuroimage. 2017 Jan;144(Pt

B):287-293. doi: 10.1016/j.neuroimage.2015.09.060. Epub 2015 Oct 9. PubMed PMID: 26439514; PubMed Central PMCID: PMC4967044

172. Suárez-Calvet M, Araque Caballero MÁ, Kleinberger G, Bateman RJ, Fagan AM, Morris JC, Levin J, Danek A, Ewers M, Haass C. Early changes in CSF sTREM2 in dominantly inherited Alzheimer's disease occur after amyloid deposition and neuronal injury. Sci Transl Med. 2016 Dec 14;8(369):369ra178. doi: 10.1126/scitranslmed.aag1767. PubMed PMID: 27974666; PubMed Central PMCID: PMC5385711

173. Tang M, Ryman DC, McDade E, Jasielec MS, Buckles VD, Cairns NJ, Fagan AM, Goate A, Marcus DS, Xiong C, Allegri RF, Chhatwal JP, Danek A, Farlow MR, Fox NC, Ghetti B, Graff-Radford NR, Laske C, Martins RN, Masters CL, Mayeux RP, Ringman JM, Rossor MN, Salloway SP, Schofield PR, Morris JC, Bateman RJ. Neurological manifestations of autosomal dominant familial Alzheimer's disease: a comparison of the published literature with the Dominantly Inherited Alzheimer Network observational study (DIAN-OBS). Lancet Neurol. 2016 Dec;15(13):1317-1325. doi: 10.1016/S1474-4422(16)30229-0. Epub 2016 Oct 21. Review. PubMed PMID: 27777020; PubMed Central PMCID: PMC5116769

174. Soosman SK, Joseph-Mathurin N, Braskie MN, Bordelon YM, Wharton D, Casado M, Coppola G, McCallum H, Nuwer M, Coutin-Churchman P, Apostolova LG, Benzinger T, Ringman JM. Widespread white matter and conduction defects in PSEN1-related spastic paraparesis. Neurobiol Aging. 2016 Nov;47:201-209. doi: 10.1016/j.neurobiolaging.2016.07.030. Epub 2016 Aug 8. PubMed PMID: 27614114; PubMed Central PMCID: PMC5075491

175. Chen L, Sun J, Xiong C. A multiple imputation approach to the analysis of clustered interval-censored failure time data with the additive hazards model. Comput Stat Data Anal. 2016 Nov;103:242-249. doi: 10.1016/j.csda.2016.05.011. Epub 2016 May 28. PubMed PMID: 27773956; PubMed Central PMCID: PMC5072417

176. Babulal GM, Ghoshal N, Head D, Vernon EK, Holtzman DM, Benzinger TLS, Fagan AM, Morris JC, Roe CM. Mood Changes in Cognitively Normal Older Adults are Linked to Alzheimer Disease Biomarker Levels. Am J Geriatr Psychiatry. 2016 Nov;24(11):1095-1104. doi: 10.1016/j.jagp.2016.04.004. Epub 2016 Apr 19. PubMed PMID: 27426238; PubMed Central PMCID: PMC5069099

177. Lim YY, Hassenstab J, Cruchaga C, Goate A, Fagan AM, Benzinger TL, Maruff P, Snyder PJ, Masters CL, Allegri R, Chhatwal J, Farlow MR, Graff-Radford NR, Laske C, Levin J, McDade E, Ringman JM, Rossor M, Salloway S, Schofield PR, Holtzman DM, Morris JC, Bateman RJ. BDNF Val66Met moderates memory impairment, hippocampal function and tau in preclinical autosomal dominant Alzheimer's disease. Brain. 2016 Oct;139(Pt 10):2766-2777. doi: 10.1093/brain/aww200. Epub 2016 Aug 12. PubMed PMID: 27521573; PubMed Central PMCID: PMC5815565

178. Su Y, Blazey TM, Owen CJ, Christensen JJ, Friedrichsen K, Joseph-Mathurin N, Wang Q, Hornbeck RC, Ances BM, Snyder AZ, Cash LA, Koeppe RA, Klunk WE, Galasko D, Brickman AM, McDade E, Ringman JM, Thompson PM, Saykin AJ, Ghetti B, Sperling RA, Johnson KA, Salloway SP, Schofield PR, Masters CL, Villemagne VL, Fox NC, Förster S, Chen K, Reiman EM, Xiong C, Marcus DS, Weiner MW, Morris JC, Bateman RJ, Benzinger TL. Correction: Quantitative Amyloid Imaging in Autosomal Dominant Alzheimer's Disease: Results from the DIAN Study Group. PLoS One. 2016;11(9):e0163669. doi: 10.1371/journal.pone.0163669. eCollection 2016. PubMed PMID: 27649320; PubMed Central PMCID: PMC5029931

179. Day GS, Musiek ES, Roe CM, Norton J, Goate AM, Cruchaga C, Cairns NJ, Morris JC. Phenotypic Similarities Between Late-Onset Autosomal Dominant and Sporadic Alzheimer Disease: A Single-Family Case-Control

Study. JAMA Neurol. 2016 Sep 1;73(9):1125-32. doi: 10.1001/jamaneurol.2016.1236. PubMed PMID: 27454811; PubMed Central PMCID: PMC5025942

180. Muenchhoff J, Poljak A, Thalamuthu A, Gupta VB, Chatterjee P, Raftery M, Masters CL, Morris JC, Bateman RJ, Fagan AM, Martins RN, Sachdev PS. Changes in the plasma proteome at asymptomatic and symptomatic stages of autosomal dominant Alzheimer's disease. Sci Rep. 2016 Jul 6;6:29078. doi: 10.1038/srep29078. PubMed PMID: 27381087; PubMed Central PMCID: PMC4933916

181. Miller-Thomas MM, Sipe AL, Benzinger TL, McConathy J, Connolly S, Schwetye KE. Multimodality Review of Amyloid-related Diseases of the Central Nervous System. Radiographics. 2016 Jul-Aug;36(4):1147-63. doi: 10.1148/rg.2016150172. Review. PubMed PMID: 27399239; PubMed Central PMCID: PMC4976469

182. Lee S, Viqar F, Zimmerman ME, Narkhede A, Tosto G, Benzinger TL, Marcus DS, Fagan AM, Goate A, Fox NC, Cairns NJ, Holtzman DM, Buckles V, Ghetti B, McDade E, Martins RN, Saykin AJ, Masters CL, Ringman JM, Ryan NS, Förster S, Laske C, Schofield PR, Sperling RA, Salloway S, Correia S, Jack C Jr, Weiner M, Bateman RJ, Morris JC, Mayeux R, Brickman AM. White matter hyperintensities are a core feature of Alzheimer's disease: Evidence from the dominantly inherited Alzheimer network. Ann Neurol. 2016 Jun;79(6):929-39. doi: 10.1002/ana.24647. Epub 2016 Apr 27. PubMed PMID: 27016429; PubMed Central PMCID: PMC4884146

183. Su Y, Blazey TM, Owen CJ, Christensen JJ, Friedrichsen K, Joseph-Mathurin N, Wang Q, Hornbeck RC, Ances BM, Snyder AZ, Cash LA, Koeppe RA, Klunk WE, Galasko D, Brickman AM, McDade E, Ringman JM, Thompson PM, Saykin AJ, Ghetti B, Sperling RA, Johnson KA, Salloway SP, Schofield PR, Masters CL, Villemagne VL, Fox NC, Förster S, Chen K, Reiman EM, Xiong C, Marcus DS, Weiner MW, Morris JC, Bateman RJ, Benzinger TL. Quantitative Amyloid Imaging in Autosomal Dominant Alzheimer's Disease: Results from the DIAN Study Group. PLoS One. 2016;11(3):e0152082. doi: 10.1371/journal.pone.0152082. eCollection 2016. PubMed PMID: 27010959; PubMed Central PMCID: PMC4807073

184. Ringman JM, Monsell S, Ng DW, Zhou Y, Nguyen A, Coppola G, Van Berlo V, Mendez MF, Tung S, Weintraub S, Mesulam MM, Bigio EH, Gitelman DR, Fisher-Hubbard AO, Albin RL, Vinters HV. Neuropathology of Autosomal Dominant Alzheimer Disease in the National Alzheimer Coordinating Center Database. J Neuropathol Exp Neurol. 2016 Mar;75(3):284-90. doi: 10.1093/jnen/nlv028. Epub 2016 Feb 17. PubMed PMID: 26888304; PubMed Central PMCID: PMC4934612

185. Guerreiro R, Escott-Price V, Darwent L, Parkkinen L, Ansorge O, Hernandez DG, Nalls MA, Clark L, Honig L, Marder K, van der Flier W, Holstege H, Louwersheimer E, Lemstra A, Scheltens P, Rogaeva E, St George-Hyslop P, Londos E, Zetterberg H, Ortega-Cubero S, Pastor P, Ferman TJ, Graff-Radford NR, Ross OA, Barber I, Braae A, Brown K, Morgan K, Maetzler W, Berg D, Troakes C, Al-Sarraj S, Lashley T, Compta Y, Revesz T, Lees A, Cairns NJ, Halliday GM, Mann D, Pickering-Brown S, Powell J, Lunnon K, Lupton MK, Dickson D, Hardy J, Singleton A, Bras J. Genome-wide analysis of genetic correlation in dementia with Lewy bodies, Parkinson's and Alzheimer's diseases. Neurobiol Aging. 2016 Feb;38:214.e7-214.e10. doi: 10.1016/j.neurobiolaging.2015.10.028. Epub 2015 Nov 2. PubMed PMID: 26643944; PubMed Central PMCID: PMC4759606

186. Chatterjee P, Lim WL, Shui G, Gupta VB, James I, Fagan AM, Xiong C, Sohrabi HR, Taddei K, Brown BM, Benzinger T, Masters C, Snowden SG, Wenk MR, Bateman RJ, Morris JC, Martins RN. Plasma Phospholipid and Sphingolipid Alterations in Presenilin1 Mutation Carriers: A Pilot Study. J Alzheimers Dis. 2016;50(3):887-94. doi: 10.3233/JAD-150948. PubMed PMID: 26836186; PubMed Central PMCID: PMC4943576

187. Reiman EM, Langbaum JB, Tariot PN, Lopera F, Bateman RJ, Morris JC, Sperling RA, Aisen PS, Roses AD, Welsh-Bohmer KA, Carrillo MC, Weninger S. CAP–advancing the evaluation of preclinical Alzheimer disease treatments. Nat Rev Neurol. 2016 Jan;12(1):56-61. doi: 10.1038/nrneurol.2015.177. Epub 2015 Sep 29. Review. PubMed PMID: 26416539; PubMed Central PMCID: PMC4847536

188. Luo J, D'Angela G, Gao F, Ding J, Xiong C. Bivariate correlation coefficients in family-type clustered studies. Biom J. 2015 Nov;57(6):1084-109. doi: 10.1002/bimj.201400131. Epub 2015 Sep 11. PubMed PMID: 26360805; PubMed Central PMCID: PMC4741284

189. Mielke MM, Machulda MM, Hagen CE, Edwards KK, Roberts RO, Pankratz VS, Knopman DS, Jack CR Jr, Petersen RC. Performance of the CogState computerized battery in the Mayo Clinic Study on Aging. Alzheimers Dement. 2015 Nov;11(11):1367-76. doi: 10.1016/j.jalz.2015.01.008. Epub 2015 Apr 6. PubMed PMID: 25858683; PubMed Central PMCID: PMC4595161

190. Wang F, Gordon BA, Ryman DC, Ma S, Xiong C, Hassenstab J, Goate A, Fagan AM, Cairns NJ, Marcus DS, McDade E, Ringman JM, Graff-Radford NR, Ghetti B, Farlow MR, Sperling R, Salloway S, Schofield PR, Masters CL, Martins RN, Rossor MN, Jucker M, Danek A, Förster S, Lane CA, Morris JC, Benzinger TL, Bateman RJ. Cerebral amyloidosis associated with cognitive decline in autosomal dominant Alzheimer disease. Neurology. 2015 Sep 1;85(9):790-8. doi: 10.1212/WNL.0000000000001903. Epub 2015 Aug 5. PubMed PMID: 26245925; PubMed Central PMCID: PMC4553024

191. Patterson BW, Elbert DL, Mawuenyega KG, Kasten T, Ovod V, Ma S, Xiong C, Chott R, Yarasheski K, Sigurdson W, Zhang L, Goate A, Benzinger T, Morris JC, Holtzman D, Bateman RJ. Age and amyloid effects on human central nervous system amyloid-beta kinetics. Ann Neurol. 2015 Sep;78(3):439-53. doi: 10.1002/ana.24454. Epub 2015 Jul 20. PubMed PMID: 26040676; PubMed Central PMCID: PMC4546566

192. Quiroz YT, Schultz AP, Chen K, Protas HD, Brickhouse M, Fleisher AS, Langbaum JB, Thiyyagura P, Fagan AM, Shah AR, Muniz M, Arboleda-Velasquez JF, Munoz C, Garcia G, Acosta-Baena N, Giraldo M, Tirado V, Ramírez DL, Tariot PN, Dickerson BC, Sperling RA, Lopera F, Reiman EM. Brain Imaging and Blood Biomarker Abnormalities in Children with Autosomal Dominant Alzheimer Disease: A Cross-Sectional Study. JAMA Neurol. 2015 Aug;72(8):912-9. doi: 10.1001/jamaneurol.2015.1099. PubMed PMID: 26121081; PubMed Central PMCID: PMC4625544

193. Yau WW, Tudorascu DL, McDade EM, Ikonomovic S, James JA, Minhas D, Mowrey W, Sheu LK, Snitz BE, Weissfeld L, Gianaros PJ, Aizenstein HJ, Price JC, Mathis CA, Lopez OL, Klunk WE. Longitudinal assessment of neuroimaging and clinical markers in autosomal dominant Alzheimer's disease: a prospective cohort study. Lancet Neurol. 2015 Aug;14(8):804-813. doi: 10.1016/S1474-4422(15)00135-0. Epub 2015 Jun 29. PubMed PMID: 26139022; PubMed Central PMCID: PMC4519011

194. Cairns NJ, Perrin RJ, Franklin EE, Carter D, Vincent B, Xie M, Bateman RJ, Benzinger T, Friedrichsen K, Brooks WS, Halliday GM, McLean C, Ghetti B, Morris JC. Neuropathologic assessment of participants in two multi-center longitudinal observational studies: the Alzheimer Disease Neuroimaging Initiative (ADNI) and the Dominantly Inherited Alzheimer Network (DIAN). Neuropathology. 2015 Aug;35(4):390-400. doi: 10.1111/neup.12205. Epub 2015 May 12. PubMed PMID: 25964057; PubMed Central PMCID: PMC4521391

195. Grill JD, Bateman RJ, Buckles V, Oliver A, Morris JC, Masters CL, Klunk WE, Ringman JM. A survey of attitudes toward clinical trials and genetic disclosure in autosomal dominant Alzheimer's disease. Alzheimers Res Ther. 2015;7(1):50. doi: 10.1186/s13195-015-0135-0. eCollection 2015. PubMed PMID: 26203303; PubMed Central PMCID: PMC4511231

196. Franklin EE, Perrin RJ, Vincent B, Baxter M, Morris JC, Cairns NJ. Brain collection, standardized neuropathologic assessment, and comorbidity in Alzheimer's Disease Neuroimaging Initiative 2 participants. Alzheimers Dement. 2015 Jul;11(7):815-22. doi: 10.1016/j.jalz.2015.05.010. Review. PubMed PMID: 26194314; PubMed Central PMCID: PMC4511380

197. Bateman RJ, Morris JC. Factors Contributing to the Post-Lumbar Puncture Headache—Reply. JAMA Neurol. 2015 Jul;72(7):835. doi: 10.1001/jamaneurol.2015.0691. PubMed PMID: 26167902; PubMed Central PMCID: PMC4943573

198. Papp KV, Amariglio RE, Mormino EC, Hedden T, Dekhytar M, Johnson KA, Sperling RA, Rentz DM. Free and cued memory in relation to biomarker-defined abnormalities in clinically normal older adults and those at risk for Alzheimer's disease. Neuropsychologia. 2015 Jul;73:169-75. doi: 10.1016/j.neuropsychologia.2015.04.034. Epub 2015 May 19. PubMed PMID: 26002757; PubMed Central PMCID: PMC4479270

199. Schindler SE, Fagan AM. Autosomal Dominant Alzheimer Disease: A Unique Resource to Study CSF Biomarker Changes in Preclinical AD. Front Neurol. 2015;6:142. doi: 10.3389/fneur.2015.00142. eCollection 2015. Review. PubMed PMID: 26175713; PubMed Central PMCID: PMC4483518

200. Su Y, Blazey TM, Snyder AZ, Raichle ME, Hornbeck RC, Aldea P, Morris JC, Benzinger TL. Quantitative amyloid imaging using image-derived arterial input function. PLoS One. 2015;10(4):e0122920. doi: 10.1371/journal.pone.0122920. eCollection 2015. PubMed PMID: 25849581; PubMed Central PMCID: PMC4388540

201. Laske C, Sohrabi HR, Jasielec MS, Müller S, Koehler NK, Gräber S, Förster S, Drzezga A, Mueller-Sarnowski F, Danek A, Jucker M, Bateman RJ, Buckles V, Saykin AJ, Martins RN, Morris JC, Dominantly Inherited Alzheimer Network Dian. Diagnostic Value of Subjective Memory Complaints Assessed with a Single Item in Dominantly Inherited Alzheimer's Disease: Results of the DIAN Study. Biomed Res Int. 2015;2015:828120. doi: 10.1155/2015/828120. Epub 2015 Apr 2. PubMed PMID: 25922840; PubMed Central PMCID: PMC4398930

202. Ringman JM, Liang LJ, Zhou Y, Vangala S, Teng E, Kremen S, Wharton D, Goate A, Marcus DS, Farlow M, Ghetti B, McDade E, Masters CL, Mayeux RP, Rossor M, Salloway S, Schofield PR, Cummings JL, Buckles V, Bateman R, Morris JC. Early behavioural changes in familial Alzheimer's disease in the Dominantly Inherited Alzheimer Network. Brain. 2015 Apr;138(Pt 4):1036-45. doi: 10.1093/brain/awv004. Epub 2015 Feb 15. PubMed PMID: 25688083; PubMed Central PMCID: PMC4963801

203. Monserrate AE, Ryman DC, Ma S, Xiong C, Noble JM, Ringman JM, Morris JC, Danek A, Müller-Sarnowski F, Clifford DB, McDade EM, Brooks WS, Darby DG, Masters CL, Weston PS, Farlow MR, Graff-Radford NR, Salloway SP, Fagan AM, Oliver A, Bateman RJ. Factors associated with the onset and persistence of post-lumbar puncture headache. JAMA Neurol. 2015 Mar;72(3):325-32. doi: 10.1001/jamaneurol.2014.3974. PubMed PMID: 25622095; PubMed Central PMCID: PMC4364538

204. Fleisher AS, Chen K, Quiroz YT, Jakimovich LJ, Gutierrez Gomez M, Langois CM, Langbaum JB, Roontiva A, Thiyyagura P, Lee W, Ayutyanont N, Lopez L, Moreno S, Muñoz C, Tirado V, Acosta-Baena N, Fagan AM, Giraldo M, Garcia G, Huentelman MJ, Tariot PN, Lopera F, Reiman EM. Associations between biomarkers and age in the presenilin 1 E280A autosomal dominant Alzheimer disease kindred: a cross-sectional study. JAMA Neurol. 2015 Mar;72(3):316-24. doi: 10.1001/jamaneurol.2014.3314. PubMed PMID: 25580592; PubMed Central PMCID: PMC4355261

205. Su Y, Blazey TM, Snyder AZ, Raichle ME, Marcus DS, Ances BM, Bateman RJ, Cairns NJ, Aldea P, Cash L, Christensen JJ, Friedrichsen K, Hornbeck RC, Farrar AM, Owen CJ, Mayeux R, Brickman AM, Klunk W, Price JC, Thompson PM, Ghetti B, Saykin AJ, Sperling RA, Johnson KA, Schofield PR, Buckles V, Morris JC, Benzinger TLS. Partial volume correction in quantitative amyloid imaging. Neuroimage. 2015 Feb 15;107:55-64. doi: 10.1016/j.neuroimage.2014.11.058. Epub 2014 Dec 5. PubMed PMID: 25485714; PubMed Central PMCID: PMC4300252

206. Wang LS, Naj AC, Graham RR, Crane PK, Kunkle BW, Cruchaga C, Murcia JD, Cannon-Albright L, Baldwin CT, Zetterberg H, Blennow K, Kukull WA, Faber KM, Schupf N, Norton MC, Tschanz JT, Munger RG, Corcoran CD, Rogaeva E, Lin CF, Dombroski BA, Cantwell LB, Partch A, Valladares O, Hakonarson H, St George-Hyslop P, Green RC, Goate AM, Foroud TM, Carney RM, Larson EB, Behrens TW, Kauwe JS, Haines JL, Farrer LA, Pericak-Vance MA, Mayeux R, Schellenberg GD, Albert MS, Albin RL, Apostolova LG, Arnold SE, Barber R, Barmada M, Barnes LL, Beach TG, Becker JT, Beecham GW, Beekly D, Bennett DA, Bigio EH, Bird TD, Blacker D, Boeve BF, Bowen JD, Boxer A, Burke JR, Buxbaum JD, Cairns NJ, Cao C, Carlson CS, Carroll SL, Chui HC, Clark DG, Cribbs DH, Crocco EA, DeCarli C, DeKosky ST, Demirci FY, Dick M, Dickson DW, Duara R, Ertekin-Taner N, Fallon KB, Farlow MR, Ferris S, Frosch MP, Galasko DR, Ganguli M, Gearing M, Geschwind DH, Ghetti B, Gilbert JR, Glass JD, Graff-Radford NR, Growdon JH, Hamilton RL, Hamilton-Nelson KL, Harrell LE, Head E, Honig LS, Hulette CM, Hyman BT, Jarvik GP, Jicha GA, Jin LW, Jun G, Jun G, Kamboh MI, Karydas A, Kaye JA, Kim R, Koo EH, Kowall NW, Kramer JH, LaFerla FM, Lah JJ, Leverenz JB, Levey AI, Li G, Lieberman AP, Lopez OL, Lunetta KL, Lyketsos CG, Mack WJ, Marson DC, Martin ER, Martiniuk F, Mash DC, Masliah E, McCormick WC, McCurry SM, McDavid AN, McKee AC, Mesulam WM, Miller BL, Miller CA, Miller JW, Montine TJ, Morris JC, Murrell JR, Olichney JM, Parisi JE, Perry W, Peskind E, Petersen RC, Pierce A, Poon WW, Potter H, Quinn JF, Raj A, Raskind M, Reiman EM, Reisberg B, Reitz C, Ringman JM, Roberson ED, Rosen HJ, Rosenberg RN, Sano M, Saykin AJ, Schneider JA, Schneider LS, Seeley WW, Smith AG, Sonnen JA, Spina S, Stern RA, Tanzi RE, Thornton-Wells TA, Trojanowski JQ, Troncoso JC, Tsuang DW, Van Deerlin VM, Van Eldik LJ, Vardarajan BN, Vinters HV, Vonsattel JP, Weintraub S, Welsh-Bohmer KA, Williamson J, Wishnek S, Woltjer RL, Wright CB, Younkin SG, Yu CE, Yu L. Rarity of the Alzheimer disease-protective APP A673T variant in the United States. JAMA Neurol. 2015 Feb;72(2):209-16. doi: 10.1001/jamaneurol.2014.2157. PubMed PMID: 25531812; PubMed Central PMCID: PMC4324097

207. Chatterjee P, Gupta VB, Fagan AM, Jasielec MS, Xiong C, Sohrabi HR, Dhaliwal S, Taddei K, Bourgeat P, Brown BM, Benzinger T, Bateman RJ, Morris JC, Martins RN. Decreased platelet APP isoform ratios in autosomal dominant Alzheimer's disease: baseline data from a DIAN cohort subset. Curr Alzheimer Res. 2015;12(2):157-64. doi: 10.2174/1567205012666150204125732. PubMed PMID: 25654503; PubMed Central PMCID: PMC4383703

208. Klunk WE, Koeppe RA, Price JC, Benzinger TL, Devous MD Sr, Jagust WJ, Johnson KA, Mathis CA, Minhas D, Pontecorvo MJ, Rowe CC, Skovronsky DM, Mintun MA. The Centiloid Project: standardizing quantitative amyloid plaque estimation by PET. Alzheimers Dement. 2015 Jan;11(1):1-15.e1-4. doi: 10.1016/j.jalz.2014.07.003. Epub 2014 Oct 28. PubMed PMID: 25443857; PubMed Central PMCID: PMC4300247

209. Karch CM, Goate AM. Alzheimer's disease risk genes and mechanisms of disease pathogenesis. Biol Psychiatry. 2015 Jan 1;77(1):43-51. doi: 10.1016/j.biopsych.2014.05.006. Epub 2014 May 17. Review. PubMed PMID: 24951455; PubMed Central PMCID: PMC4234692

210. Xiong C, Weng H, Bennett DA, Boyle PA, Shah RC, Fague S, Hall CB, Lipton RB, Morris JC. Subsets of a large cognitive battery better power clinical trials on early stage Alzheimer's

disease. Neuroepidemiology. 2014;43(2):131-9. doi: 10.1159/000365733. Epub 2014 Nov 5. PubMed PMID: 25376544; PubMed Central PMCID: PMC4237272

211. Raman MR, Wiste HJ, Senjem ML, Ward CP, Jack CR Jr, Kantarci K. Spontaneous amyloid-related imaging abnormalities in a cognitively normal adult. Neurology. 2014 Nov 4;83(19):1771-2. doi: 10.1212/WNL.0000000000000957. PubMed PMID: 25367059; PubMed Central PMCID: PMC4239833

212. Ringman JM, Goate A, Masters CL, Cairns NJ, Danek A, Graff-Radford N, Ghetti B, Morris JC. Genetic heterogeneity in Alzheimer disease and implications for treatment strategies. Curr Neurol Neurosci Rep. 2014 Nov;14(11):499. doi: 10.1007/s11910-014-0499-8. Review. PubMed PMID: 25217249; PubMed Central PMCID: PMC4162987

213. Brier MR, Thomas JB, Snyder AZ, Wang L, Fagan AM, Benzinger T, Morris JC, Ances BM. Unrecognized preclinical Alzheimer disease confounds rs-fcMRI studies of normal aging. Neurology. 2014 Oct 28;83(18):1613-9. doi: 10.1212/WNL.0000000000000939. Epub 2014 Sep 26. PubMed PMID: 25261500; PubMed Central PMCID: PMC4223085

214. Pepe A, Dinov I, Tohka J. An automatic framework for quantitative validation of voxel based morphometry measures of anatomical brain asymmetry. Neuroimage. 2014 Oct 15;100:444-59. doi: 10.1016/j.neuroimage.2014.06.029. Epub 2014 Jun 18. PubMed PMID: 24952229; PubMed Central PMCID: PMC4457344

215. Thomas JB, Brier MR, Bateman RJ, Snyder AZ, Benzinger TL, Xiong C, Raichle M, Holtzman DM, Sperling RA, Mayeux R, Ghetti B, Ringman JM, Salloway S, McDade E, Rossor MN, Ourselin S, Schofield PR, Masters CL, Martins RN, Weiner MW, Thompson PM, Fox NC, Koeppe RA, Jack CR Jr, Mathis CA, Oliver A, Blazey TM, Moulder K, Buckles V, Hornbeck R, Chhatwal J, Schultz AP, Goate AM, Fagan AM, Cairns NJ, Marcus DS, Morris JC, Ances BM. Functional connectivity in autosomal dominant and late-onset Alzheimer disease. JAMA Neurol. 2014 Sep;71(9):1111-22. doi: 10.1001/jamaneurol.2014.1654. PubMed PMID: 25069482; PubMed Central PMCID: PMC4240274

216. Ryman DC, Acosta-Baena N, Aisen PS, Bird T, Danek A, Fox NC, Goate A, Frommelt P, Ghetti B, Langbaum JB, Lopera F, Martins R, Masters CL, Mayeux RP, McDade E, Moreno S, Reiman EM, Ringman JM, Salloway S, Schofield PR, Sperling R, Tariot PN, Xiong C, Morris JC, Bateman RJ. Symptom onset in autosomal dominant Alzheimer disease: a systematic review and meta-analysis. Neurology. 2014 Jul 15;83(3):253-60. doi: 10.1212/WNL.0000000000000596. Epub 2014 Jun 13. Review. PubMed PMID: 24928124; PubMed Central PMCID: PMC4117367

217. Yu P, Sun J, Wolz R, Stephenson D, Brewer J, Fox NC, Cole PE, Jack CR Jr, Hill DL, Schwarz AJ. Operationalizing hippocampal volume as an enrichment biomarker for amnestic mild cognitive impairment trials: effect of algorithm, test-retest variability, and cut point on trial cost, duration, and sample size. Neurobiol Aging. 2014 Apr;35(4):808-18. doi: 10.1016/j.neurobiolaging.2013.09.039. Epub 2013 Oct 3. PubMed PMID: 24211008; PubMed Central PMCID: PMC4201941

218. Fagan AM, Xiong C, Jasielec MS, Bateman RJ, Goate AM, Benzinger TL, Ghetti B, Martins RN, Masters CL, Mayeux R, Ringman JM, Rossor MN, Salloway S, Schofield PR, Sperling RA, Marcus D, Cairns NJ, Buckles VD, Ladenson JH, Morris JC, Holtzman DM. Longitudinal change in CSF biomarkers in autosomal-dominant Alzheimer's disease. Sci Transl Med. 2014 Mar 5;6(226):226ra30. doi: 10.1126/scitranslmed.3007901. PubMed PMID: 24598588; PubMed Central PMCID: PMC4038930

219. Dong T, Kang L, Hutson A, Xiong C, Tian L. Confidence interval estimation of the difference between two sensitivities to the early disease stage. Biom J. 2014 Mar;56(2):270-86. doi: 10.1002/bimj.201200012. Epub 2013 Nov 22. PubMed PMID: 24265123; PubMed Central PMCID: PMC4349212

220. Grill JD, Monsell SE. Choosing Alzheimer's disease prevention clinical trial populations. Neurobiol Aging. 2014 Mar;35(3):466-71. doi: 10.1016/j.neurobiolaging.2013.09.001. Epub 2013 Oct 9. PubMed PMID: 24119546; PubMed Central PMCID: PMC3864603

221. Armstrong RA, Kotzbauer PT, Perlmutter JS, Campbell MC, Hurth KM, Schmidt RE, Cairns NJ. A quantitative study of α-synuclein pathology in fifteen cases of dementia associated with Parkinson disease. J Neural Transm (Vienna). 2014 Feb;121(2):171-81. doi: 10.1007/s00702-013-1084-z. Epub 2013 Aug 31. PubMed PMID: 23996276; PubMed Central PMCID: PMC4041534

222. Cruchaga C, Karch CM, Jin SC, Benitez BA, Cai Y, Guerreiro R, Harari O, Norton J, Budde J, Bertelsen S, Jeng AT, Cooper B, Skorupa T, Carrell D, Levitch D, Hsu S, Choi J, Ryten M, Sassi C, Bras J, Gibbs RJ, Hernandez DG, Lupton MK, Powell J, Forabosco P, Ridge PG, Corcoran CD, Tschanz JT, Norton MC, Munger RG, Schmutz C, Leary M, Demirci FY, Bamne MN, Wang X, Lopez OL, Ganguli M, Medway C, Turton J, Lord J, Braae A, Barber I, Brown K, Pastor P, Lorenzo-Betancor O, Brkanac Z, Scott E, Topol E, Morgan K, Rogaeva E, Singleton A, Hardy J, Kamboh MI, George-Hyslop PS, Cairns N, Morris JC, Kauwe JSK, Goate AM. Rare coding variants in the phospholipase D3 gene confer risk for Alzheimer's disease. Nature. 2014 Jan 23;505(7484):550-554. doi: 10.1038/nature12825. Epub 2013 Dec 11. PubMed PMID: 24336208; PubMed Central PMCID: PMC4050701

223. Gordon BA, Blazey T, Benzinger TL. Regional variability in Alzheimer's disease biomarkers. Future Neurol. 2014;9(2):131-134. doi: 10.2217/fnl.14.9. PubMed PMID: 25309132; PubMed Central PMCID: PMC4192718

224. Ting SK, Benzinger T, Kepe V, Fagan A, Coppola G, Porter V, Hecimovic S, Chakraverty S, Alvarez-Retuerto AI, Goate A, Ringman JM. A novel PSEN1 mutation (I238M) associated with early-onset Alzheimer's disease in an African-American woman. J Alzheimers Dis. 2014;40(2):271-5. doi: 10.3233/JAD-131844. PubMed PMID: 24413619; PubMed Central PMCID: PMC3972314

225. Xiong C, Luo J, Gao F, Morris JC. Optimizing parameters in clinical trials with a randomized start or withdrawal design. Comput Stat Data Anal. 2014 Jan 1;69:101-113. doi: 10.1016/j.csda.2013.07.013. PubMed PMID: 24159249; PubMed Central PMCID: PMC3804275

226. Storandt M, Balota DA, Aschenbrenner AJ, Morris JC. Clinical and psychological characteristics of the initial cohort of the Dominantly Inherited Alzheimer Network (DIAN). Neuropsychology. 2014 Jan;28(1):19-29. doi: 10.1037/neu0000030. Epub 2013 Nov 11. PubMed PMID: 24219606; PubMed Central PMCID: PMC3877741

227. Kang L, Xiong C, Tian L. Estimating confidence intervals for the difference in diagnostic accuracy with three ordinal diagnostic categories without a gold standard. Comput Stat Data Anal. 2013 Dec;68. doi: 10.1016/j.csda.2013.07.007. PubMed PMID: 24415817; PubMed Central PMCID: PMC3883051

228. Royle NA, Booth T, Valdés Hernández MC, Penke L, Murray C, Gow AJ, Maniega SM, Starr J, Bastin ME, Deary IJ, Wardlaw JM. Estimated maximal and current brain volume predict cognitive ability in old age. Neurobiol Aging. 2013 Dec;34(12):2726-33. doi: 10.1016/j.neurobiolaging.2013.05.015. Epub 2013 Jul 11. PubMed PMID: 23850342; PubMed Central PMCID: PMC3988920

229. Benzinger TL, Blazey T, Jack CR Jr, Koeppe RA, Su Y, Xiong C, Raichle ME, Snyder AZ, Ances BM, Bateman RJ, Cairns NJ, Fagan AM, Goate A, Marcus DS, Aisen PS, Christensen JJ, Ercole L, Hornbeck RC, Farrar AM,

Aldea P, Jasielec MS, Owen CJ, Xie X, Mayeux R, Brickman A, McDade E, Klunk W, Mathis CA, Ringman J, Thompson PM, Ghetti B, Saykin AJ, Sperling RA, Johnson KA, Salloway S, Correia S, Schofield PR, Masters CL, Rowe C, Villemagne VL, Martins R, Ourselin S, Rossor MN, Fox NC, Cash DM, Weiner MW, Holtzman DM, Buckles VD, Moulder K, Morris JC. Regional variability of imaging biomarkers in autosomal dominant Alzheimer's disease. Proc Natl Acad Sci U S A. 2013 Nov 19;110(47):E4502-9. doi: 10.1073/pnas.1317918110. Epub 2013 Nov 5. PubMed PMID: 24194552; PubMed Central PMCID: PMC3839740

230. Su Y, D'Angelo GM, Vlassenko AG, Zhou G, Snyder AZ, Marcus DS, Blazey TM, Christensen JJ, Vora S, Morris JC, Mintun MA, Benzinger TL. Quantitative analysis of PiB-PET with FreeSurfer ROIs. PLoS One. 2013;8(11):e73377. doi: 10.1371/journal.pone.0073377. eCollection 2013. PubMed PMID: 24223109; PubMed Central PMCID: PMC3819320

231. Peters KR, Lynn Beattie B, Feldman HH, Illes J. A conceptual framework and ethics analysis for prevention trials of Alzheimer Disease. Prog Neurobiol. 2013 Nov;110:114-23. doi: 10.1016/j.pneurobio.2012.12.001. Epub 2013 Jan 21. Review. PubMed PMID: 23348495

232. Moulder KL, Snider BJ, Mills SL, Buckles VD, Santacruz AM, Bateman RJ, Morris JC. Dominantly Inherited Alzheimer Network: facilitating research and clinical trials. Alzheimers Res Ther. 2013;5(5):48. doi: 10.1186/alzrt213. eCollection 2013. Review. PubMed PMID: 24131566; PubMed Central PMCID: PMC3978584

233. Cash DM, Ridgway GR, Liang Y, Ryan NS, Kinnunen KM, Yeatman T, Malone IB, Benzinger TL, Jack CR Jr, Thompson PM, Ghetti BF, Saykin AJ, Masters CL, Ringman JM, Salloway SP, Schofield PR, Sperling RA, Cairns NJ, Marcus DS, Xiong C, Bateman RJ, Morris JC, Rossor MN, Ourselin S, Fox NC. The pattern of atrophy in familial Alzheimer disease: volumetric MRI results from the DIAN study. Neurology. 2013 Oct 15;81(16):1425-33. doi: 10.1212/WNL.0b013e3182a841c6. Epub 2013 Sep 18. PubMed PMID: 24049139; PubMed Central PMCID: PMC3806583

234. Shimada H. [The DIAN study]. Brain Nerve. 2013 Oct;65(10):1179-84. Review. PubMed PMID: 24101429

235. Wang L, Brier MR, Snyder AZ, Thomas JB, Fagan AM, Xiong C, Benzinger TL, Holtzman DM, Morris JC, Ances BM. Cerebrospinal fluid Aβ42, phosphorylated Tau181, and resting-state functional connectivity. JAMA Neurol. 2013 Oct;70(10):1242-8. doi: 10.1001/jamaneurol.2013.3253. PubMed PMID: 23959173; PubMed Central PMCID: PMC3836828

236. Frost SM, Kanagasingam Y, Sohrabi HR, Taddei K, Bateman R, Morris J, Benzinger T, Goate A, Masters CL, Martins RN. Pupil response biomarkers distinguish amyloid precursor protein mutation carriers from non-carriers. Curr Alzheimer Res. 2013 Oct;10(8):790-6. doi: 10.2174/15672050113109990154. PubMed PMID: 23919771; PubMed Central PMCID: PMC3879087

237. Mills SM, Mallmann J, Santacruz AM, Fuqua A, Carril M, Aisen PS, Althage MC, Belyew S, Benzinger TL, Brooks WS, Buckles VD, Cairns NJ, Clifford D, Danek A, Fagan AM, Farlow M, Fox N, Ghetti B, Goate AM, Heinrichs D, Hornbeck R, Jack C, Jucker M, Klunk WE, Marcus DS, Martins RN, Masters CM, Mayeux R, McDade E, Morris JC, Oliver A, Ringman JM, Rossor MN, Salloway S, Schofield PR, Snider J, Snyder P, Sperling RA, Stewart C, Thomas RG, Xiong C, Bateman RJ. Preclinical trials in autosomal dominant AD: implementation of the DIAN-TU trial. Rev Neurol (Paris). 2013 Oct;169(10):737-43. doi: 10.1016/j.neurol.2013.07.017. Epub 2013 Sep 6. Review. PubMed PMID: 24016464; PubMed Central PMCID: PMC3880800

238. Duchek JM, Balota DA, Thomas JB, Snyder AZ, Rich P, Benzinger TL, Fagan AM, Holtzman DM, Morris JC, Ances BM. Relationship between Stroop performance and resting state functional connectivity in cognitively normal older adults. Neuropsychology. 2013 Sep;27(5):516-28. doi: 10.1037/a0033402. PubMed PMID: 24040929; PubMed Central PMCID: PMC3837537

239. Hooper M, Grill JD, Rodriguez-Agudelo Y, Medina LD, Fox M, Alvarez-Retuerto AI, Wharton D, Brook J, Ringman JM. The impact of the availability of prevention studies on the desire to undergo predictive testing in persons at risk for autosomal dominant Alzheimer's disease. Contemp Clin Trials. 2013 Sep;36(1):256-62. doi: 10.1016/j.cct.2013.07.006. Epub 2013 Jul 19. PubMed PMID: 23876673; PubMed Central PMCID: PMC3858206

240. Chhatwal JP, Schultz AP, Johnson K, Benzinger TL, Jack C Jr, Ances BM, Sullivan CA, Salloway SP, Ringman JM, Koeppe RA, Marcus DS, Thompson P, Saykin AJ, Correia S, Schofield PR, Rowe CC, Fox NC, Brickman AM, Mayeux R, McDade E, Bateman R, Fagan AM, Goate AM, Xiong C, Buckles VD, Morris JC, Sperling RA. Impaired default network functional connectivity in autosomal dominant Alzheimer disease. Neurology. 2013 Aug 20;81(8):736-44. doi: 10.1212/WNL.0b013e3182a1aafe. Epub 2013 Jul 24. PubMed PMID: 23884042; PubMed Central PMCID: PMC3776464

241. Liu CY, Krishnan AP, Yan L, Smith RX, Kilroy E, Alger JR, Ringman JM, Wang DJ. Complexity and synchronicity of resting state blood oxygenation level-dependent (BOLD) functional MRI in normal aging and cognitive decline. J Magn Reson Imaging. 2013 Jul;38(1):36-45. doi: 10.1002/jmri.23961. Epub 2012 Dec 7. PubMed PMID: 23225622; PubMed Central PMCID: PMC3610850

242. Xiong C, van Belle G, Chen K, Tian L, Luo J, Gao F, Yan Y, Chen L, Morris JC, Crane P. Combining Multiple Markers to Improve the Longitudinal Rate of Progression-Application to Clinical Trials on the Early Stage of Alzheimer's Disease. Stat Biopharm Res. 2013 Jan 1;5(1). doi: 10.1080/19466315.2012.756662. PubMed PMID: 24363827; PubMed Central PMCID: PMC3868484

243. Luo J, Xiong C. Youden index and Associated Cut-points for Three Ordinal Diagnostic Groups. Commun Stat Simul Comput. 2013 Jan;42(6):1213-1234. doi: 10.1080/03610918.2012.661906. PubMed PMID: 23794784; PubMed Central PMCID: PMC3685301

244. Morris JC, Aisen PS, Bateman RJ, Benzinger TL, Cairns NJ, Fagan AM, Ghetti B, Goate AM, Holtzman DM, Klunk WE, McDade E, Marcus DS, Martins RN, Masters CL, Mayeux R, Oliver A, Quaid K, Ringman JM, Rossor MN, Salloway S, Schofield PR, Selsor NJ, Sperling RA, Weiner MW, Xiong C, Moulder KL, Buckles VD. Developing an international network for Alzheimer research: The Dominantly Inherited Alzheimer Network. Clin Investig (Lond). 2012 Oct 1;2(10):975-984. doi: 10.4155/cli.12.93. PubMed PMID: 23139856; PubMed Central PMCID: PMC3489185

245. Luo J, Xiong C. DiagTest3Grp: An R Package for Analyzing Diagnostic Tests with Three Ordinal Groups. J Stat Softw. 2012 Oct;51(3):1-24. doi: 10.18637/jss.v051.i03. Epub 2012 Sep 22. PubMed PMID: 23504300; PubMed Central PMCID: PMC3595562

246. Ryan NS, Bastos-Leite AJ, Rohrer JD, Werring DJ, Fox NC, Rossor MN, Schott JM. Cerebral microbleeds in familial Alzheimer's disease. Brain. 2012 Jan;135(Pt 1):e201; author reply e202. doi: 10.1093/brain/awr126. Epub 2011 Jun 17. PubMed PMID: 21685457; PubMed Central PMCID: PMC3859452

247. Ryan NS, Rossor MN. Defining and describing the pre-dementia stages of familial Alzheimer's disease. Alzheimers Res Ther. 2011 Sep 27;3(5):29. doi: 10.1186/alzrt91. PubMed PMID: 21952009; PubMed Central PMCID: PMC3218806

248. Medina LD, Rodriguez-Agudelo Y, Geschwind DH, Gilbert PE, Liang LJ, Cummings JL, Ringman JM. Propositional density and apolipoprotein E genotype among persons at risk for familial Alzheimer's disease. Dement Geriatr Cogn Disord. 2011;32(3):188-92. doi: 10.1159/000333023. Epub 2011 Aug 30. PubMed PMID: 22134129; PubMed Central PMCID: PMC3542946

249. Holtzman DM, Morris JC, Goate AM. Alzheimer's disease: the challenge of the second century. Sci Transl Med. 2011 Apr 6;3(77):77sr1. doi: 10.1126/scitranslmed.3002369. Review. PubMed PMID: 21471435; PubMed Central PMCID: PMC3130546

250. Xiong C, van Belle G, Miller JP, Morris JC. Designing clinical trials to test disease-modifying agents: application to the treatment trials of Alzheimer's disease. Clin Trials. 2011 Feb;8(1):15-26. doi: 10.1177/1740774510392391. PubMed PMID: 21335587; PubMed Central PMCID: PMC3146242

251. Bateman RJ, Aisen PS, De Strooper B, Fox NC, Lemere CA, Ringman JM, Salloway S, Sperling RA, Windisch M, Xiong C. Autosomal-dominant Alzheimer's disease: a review and proposal for the prevention of Alzheimer's disease. Alzheimers Res Ther. 2011 Jan 6;3(1):1. doi: 10.1186/alzrt59. PubMed PMID: 21211070; PubMed Central PMCID: PMC3109410

252. Ryan NS, Rossor MN. Correlating familial Alzheimer's disease gene mutations with clinical phenotype. Biomark Med. 2010 Feb;4(1):99-112. doi: 10.2217/bmm.09.92. Review. PubMed PMID: 20387306; PubMed Central PMCID: PMC3937872

253. Ringman JM, Grill J, Rodriguez-Agudelo Y, Chavez M, Xiong C. Commentary on "a roadmap for the prevention of dementia II: Leon Thal Symposium 2008." Prevention trials in persons at risk for dominantly inherited Alzheimer's disease: opportunities and challenges. Alzheimers Dement. 2009 Mar;5(2):166-71. doi: 10.1016/j.jalz.2008.12.002. Review. PubMed PMID: 19328453; PubMed Central PMCID: PMC2746429.

E. Supplementary File 2 - DIAN Obs Consortium Author List

Last Name	First Name	Site	Institution	Affiliation	Core	Role
Bateman	Randall	011	Washington University	Washington University School of Medicine in St. Louis	Administration	Site PI/DIAN Co-Director
Daniels	Alisha J.		Washington University	Washington University in St. Louis	Administration	DIAN Obs Executive Director
Courtney	Laura		Washington University	Washington University in St. Louis	Administration	Clinical Research Manager
Ziegemeier	Angela		Washington University	Washington University in St. Louis	Administration	Project Manager
Skrbec	Karina		Washington University	Washington University in St. Louis	Administration	Clinical Research Coordinator II
Hellm	Cortaiga		Washington University	Washington University in St. Louis	Administration	Clinical Research Coordinator II
Martin	Mariana		Washington University	Washington University in St. Louis	Administration	Clinical Research Coordinator II
Ziegemeier	Ellen		Washington University	Washington University in St. Louis	DIAN EXR	Clinical Research Specialist
Bartzel	Jamie		Washington University	Washington University in St. Louis	DIAN EXR	Clinical Research Coordinator II
McDade	Eric		Washington University	Washington University School of Medicine in St. Louis, Department of Neurology	Administration	DIAN Obs Director/PI
Llibre-Guerra	Jorge J.		Washington University	Dominantly Inherited Alzheimer's Network Department of Neurology, Washington University School of Medicine in St. Louis	Administration/Clinical	DIAN Obs Assistant Director/Core Leader
Supnet-Bell	Charlene		Washington University	Washington University in St. Louis, School of Medicine, Department of Neurology	Administration	DIAN Obs Associate Director
Xiong	Chengie		Washington University	Washington University in St. Louis, School of Medicine	Biostatistics	Core Leader
Xu	Xiong		Washington University	Washington University in St. Louis, School of Medicine	Biostatistics	Senior Statistical Data Analyst
Lu	Ruijin		Washington University	Washington University in St. Louis, School of Medicine	Biostatistics	Asst. Professor of Biostatistics
Wang	Guoqiao		Washington University	Washington University in St. Louis, School of Medicine	Biostatistics	Assoc Prof of Neurology
Li	Yan		Washington University	Washington University in St. Louis, School of Medicine	Biostatistics	Assoc Prof of Neurology
Nie	Yuzheng		Washington University	Washington University in St. Louis, School of Medicine	Biostatistics	Data Analyst
Gremminger	Emily		Washington University	Washington University in St. Louis, School of Medicine	Biostatistics	Research Lab Manager
Arora	Jyoti		Washington University	Washington University in St. Louis, School of Medicine	Biostatistics	Data Analyst
Perrin	Richard J.		Washington University	Department of Pathology and Immunology, Department of Neurology, Knight Alzheimer Disease Research Center, Washington University School of Medicine, Saint Louis, MO, USA,	Neuropath	Core Leader
Franklin	Erin E.		Washington University	Department of Pathology and Immunology, Washington University in St. Louis	Neuropath	Associate Director of Clinical Research
Ibanez	Laura		Washington University	Washington University in St. Louis, Departments of Psychiatry & Neurology; NeuroGenomics and Informatics Center	Biomarker	Core Leader
Jerome	Gina		Washington University	Washington University in St. Louis, School of Medicine, Department of Psychiatry	Biomarker	Staff Scientist
Stauber	Jennifer		Washington University	Washington University in St. Louis, School of Medicine, Department of Psychiatry	Biomarker	Clinic Research Specialist
Baker	Bryce		Washington University	Washington University in St. Louis, School of Medicine, Department of Psychiatry	Biomarker	Senior Lab Tech

E. DIAN Obs Consortium Author List

Minton	Matthew		Washington University	Washington University in St. Louis, School of Medicine, Department of Psychiatry	Biomarker	Staff Scientist
Preminger	Sam		Washington University	Washington University in St. Louis, School of Medicine, Department of Psychiatry	Biomarker	Clinical Research Specialist
Cruchaga	Carlos		Washington University	1. Department of Psychiatry, Washington University School of Medicine, St. Louis, MO, USA<br><br>2. NeuroGenomics and Informatics Center, Washington University School of Medicine, St. Louis, MO, USA	Genetics	Co-Core Leader
Goate	Alison M.		Mount Sinai	1. Jean C. & James W. Crystal Professor and Chair<br>2. Director, Ronald M. Loeb Center for Alzheimer's disease<br>3. Dept. of Genetics & Genomic Sciences, Icahn Genomics Institute<br>4. Icahn School of Medicine at Mount Sinai, New York, NY	Genetics	Co-Core Leader
Renton	Alan E.		Mount Sinai	Ronald M. Loeb Center for Alzheimer's Disease, Dept of Genetics and Genomic Sciences, Icahn School of Medicine at Mount Sinai, New York, NY, USA	Genetics	Co-Core Leader
Picarello	Danielle M.		Mount Sinai	Ronald M. Loeb Center for Alzheimer's Disease, Dept of Genetics and Genomic Sciences and Nash Family Dept of Neuroscience, Icahn School of Medicine at Mount Sinai, New York, NY, USA	Genetics	Lab Tech/Analyst
Fulton-Howard	Brian		Mount Sinai	Ronald M. Loeb Center for Alzheimer's Disease, Dept of Genetics and Genomic Sciences and Nash Family Dept of Neuroscience, Icahn School of Medicine at Mount Sinai, New York, NY, USA	Genetics	
Benzinger	Tammie L.S.		Washington University	Washington University in St. Louis	Imaging	Core Leader
Gordon	Brian A.		Washington University	Washington University in St. Louis	Imaging	Co-Core Leader
Banks	Jessica		Washington University	Washington University in St. Louis	Imaging	Program Coordinator
Hornbeck	Russ		Washington University	Washington University in St. Louis	Imaging	Director IT
Chen	Allison		Washington University	Washington University in St. Louis	Imaging	Senior Scientist
Chen	Charles		Washington University	Washington University in St. Louis	Imaging	Postdoc Fellow
Flores	Shaney		Washington University	Washington University in St. Louis	Imaging	Instructor in Radiology
Goyal	Manu		Washington University	Washington University in St. Louis	Imaging	Assoc. Professor of Radiology
Joseph-Mathurin	Nelly		Washington University	Washington University in St. Louis	Imaging	Asst. Professor of Radiology
Jackson	Kelley		Washington University	Washington University in St. Louis	Imaging	Clinical Trials Manager
Keefe	Sarah		Washington University	Washington University in St. Louis	Imaging	Application Developer II
Koudelis	Deborah		Washington University	Washington University in St. Louis	Imaging	Clinical Trials Manager
Massoumzadeh	Parinaz		Washington University	Washington University in St. Louis	Imaging	Senior Scientist
McKay	Nicole		Washington University	Washington University in St. Louis	Imaging	Instructor in Radiology
Wang	Qing		Washington University	Washington University in St. Louis	Imaging	Asst. Professor of Radiology

E. DIAN Obs Consortium Author List

Sabaredzovic	Edita		Washington University	Washington University in St. Louis	Imaging	Senior Clinical Research Coordinator
Scott	Jalen		Washington University	Washington University in St. Louis	Imaging	Neuroimaging Engineer
Simmons	Ashlee		Washington University	Washington University in St. Louis	Imaging	Research Tech II
Rizzo	Jacqueline		Washington University	Washington University in St. Louis	Imaging	Research Tech II
Vlassenko	Andrei		Washington University	Washington University in St. Louis	Imaging	Assoc. Professor of Radiology
Wang	Yong		Washington University	Washington University in St. Louis	Imaging	Investigator
Smith	Thomas		Washington University	Washington University in St. Louis	Imaging	Senior Neuroimaging Engineer
Murphy	Mei		Washington University	Washington University in St. Louis	Imaging	PhD Candidate
Ances	Beau		Washington University	Washington University in St. Louis	Imaging	Professory of Neurology
Dombrowski	Kaitlyn		Washington University	Washington University in St. Louis	Imaging	Neuroimaging Research Technician II
Hoagey	David		Washington University	Washington University in St. Louis	Imaging	Postdoc Fellow
Millar	Peter		Washington University	Washington University in St. Louis	Imaging	Instructor in Neurology
Powles	Savannah		Washington University	Washington University in St. Louis	Imaging	PhD Candidate
Melson	Griffin		Washington University	Washington University in St. Louis	Imaging	Business & Technology Application Tech I
Hassenstab	Jason		Washington University	Norman J. Stupp Professor of Neurology; Professor of Psychological & Brain Sciences Washington University in St. Louis	Cognition	Core Leader
Smith	Jennifer		Washington University	Department of Neurology, Washington University in St. Louis	Cognition	Manager -Clinical Trials
Stout	Sarah		Washington University	Department of Neurology, Washington University in St. Louis	Cognition	Manager -Clinical Trials
Vila-Castelar	Clara		Washington University	Department of Neurology, Washington University in St. Louis	Cognition	Assistant Professor of Neurology
Frank	Colleen		Washington University	Department of Neurology, Washington University in St. Louis	Cognition	Postdoc Fellow
Aschenbrenner	Andrew J.	N/A	Kansas University Medical Center	Assistant Professor of Neurology	Cognition	Asst. Professor of Neurology
Karch	Celeste M.		Washington University	Department of Psychiatry, Washington University in St Louis	Administration Core/Project 1 Leader	DIAN Obs Scientific Director
Marsh	Jacob		Washington University	DIAN Fibroblast and Stem Cell Bank, Washington University in St. Louis	Genetics	Research Lab Manager
Morris	John C.		Washington University	Washington University in St. Louis, Department of Neurology and the Knight Alzheimer Disease Research Center		DIAN Obs Associate Director
Holtzman	David M.		Washington University	Department of Neurology, Knight Alzheimer's Disease Research Center, Hope Center for Neurological Disorders, Washington University in St. Louis		DIAN Obs Associated Director
Barthélemy	Nicolas R.		Washington University	1. Washington University in St. Louis, School of Medicine, Department of Neurology 2. Tracy Family Stable Isotope Labeling Quantitation (SILQ) Center, Washington University School of Medicine, St. Louis, MO, USA	Neurology	
Xu	Jinbin		Washington University	Washington University in St. Louis		
Berman	Sarah B.	024	University of Pittsburgh	University of Pittsburgh, Departments of Medicine and Neurology		Site PI

E. DIAN Obs Consortium Author List

Nadkarni	Neelesh	024	University of Pittsburgh	University of Pittsburgh, Departments of Medicine		Co-Investigator
Ikonomovic	Snezana	024	University of Pittsburgh	University of Pittsburgh, Department of Neurology		Clinical Coordinator
Day	Gregory S.	036	Mayo	Mayo Clinic in Florida; Jacksonville, FL, USA, Department of Neurology		Site PI
Lachner	Christian	036	Mayo	Mayo Clinic in Florida; Jacksonville, FL, USA, Department of Neurology and Departments of Psychiatry and Psychology		Sub Investigator
Farlow	Martin	037	Indiana University	1. Indiana School of Medicine<br>2. Indiana University Health		Site PI
Chhatwal	Jasmeer P.	094	BWH	Massachusetts General Hospital, Brigham and Women's Hospital, Harvard Medical School		Site PI
Pinilla	Valentina	094	BWH	Massachusetts General Hospital, Brigham and Women's Hospital, Harvard Medical School		Site Co-Coordinator
Maa	Courtney	094	BWH	Massachusetts General Hospital, Brigham and Women's Hospital, Harvard Medical School		Site Coordinator
Ikeuchi	Takeshi	720	Niigata	Brain Research Institute, Niigata University		Site Co-PI
Ishiguro	Takanobu	720	Niigata	Brain Research Institute, Niigata University		Site PI
Aoyama	Azusa	720	Niigata	Brain Research Institute, Niigata University		Investigator
Ishii	Kenji	720	Niigata	Tokyo Metropolitan Institute of Gerontology		PET site leader
Senda	Michio	720	Niigata	Kobe City Medical Center General Hospital		PET site leader
Niimi	Yoshiki	730	Tokyo	Unit for Early and Exploratory Clinical Development, The University of Tokyo Hospital		Site PI
Huey	Edward D.	941	Butler	Department of Psychiatry and Human Behavior, Alpert Medical School, Brown University		Site PI
Bodge	Courtney	941	Butler	Memory and Aging Program, Butler Hospital,		Research Manager
Salloway	Stephen	941	Butler	Memory and Aging Program, Butler Hospital, Departments of Psychiatry and Human Behavior and Neurology, Alpert Medical School, Brown University		
Devenney	Emma	950	Sydney	1 Neuroscience Research Australia, Sydney NSW 2031 Australia<br>2 School of Clinical Medicine, University of New South Wales, Sydney NSW 2052 Australia		Site PI
Schofield	Peter R.	950	Sydney	1 Neuroscience Research Australia, Sydney NSW 2031 Australia<br>2 School of Biomedical Sciences, University of New South Wales, Sydney NSW 2052 Australia		Advisor
Brooks	William S.	950	Sydney	1 Neuroscience Research Australia, Sydney NSW 2031 Australia; and<br>2 School of Clinical Medicine, University of New South Wales, Sydney NSW 2052 Australia		Clinician
Bechara	Jacob A.	950	Sydney	Neuroscience Research Australia, Sydney NSW 2031 Australia		Site Coordinator

E. DIAN Obs Consortium Author List

Martins	Ralph N.	952	Perth	1. Centre of Excellence for Alzheimer's Disease Research and Care, School of Medical and Health Sciences, Edith Cowan University, Joondalup, Western Australia, Australia.<br>2. Alzheimer's Research Australia, Ralph and Patricia Sarich Neuroscience Research Institute, Nedlands, Western Australia, Australia.<br>3.Department of Biomedical Sciences, Macquarie University, Sydney, New South Wales, Australia.		Site PI
Sohrabi	Hamid R.	952	Perth	1. Alzheimer's Research Australia, Ralph and Patricia Sarich Neuroscience Research Institute, Nedlands, Western Australia, Australia. 2. Centre for Healthy Ageing, Health Futures Institute, Murdoch University, Murdoch, Western Australia, Australia. 3. School of Psychology, Murdoch University, Murdoch, Western Australia, Australia.		Site Clinical NP Lead
Taddei	Kevin	952	Perth	1. Centre of Excellence for Alzheimer's Disease Research and Care, School of Medical and Health Sciences, Edith Cowan University, Joondalup, Western Australia, Australia.<br>2. Alzheimer's Research Australia, Ralph and Patricia Sarich Neuroscience Research Institute, Nedlands, Western Australia, Australia.		Site Project Manager
Gardener	Samanatha L.	952	Perth			Site Coordinator
Fox	Nick C.	953	UCL	1. Dementia Research Centre, UCL Queen Square Institute of Neurology, London, United Kingdom<br>2. UK Dementia Research Institute at UCL, London, United Kingdom		Site PI
Cash	David M.	953	UCL	1. Dementia Research Centre, UCL Queen Square Institute of Neurology, London, United Kingdom<br>2. UK Dementia Research Institute at UCL, London, United Kingdom		Imaging Lead
Ryan	Natalie S.	953	UCL	1. Dementia Research Centre, UCL Queen Square Institute of Neurology, London, United Kingdom<br>2. UK Dementia Research Institute at UCL, London, United Kingdom		Clinical Investigator/Sub-PI
Jucker	Mathias	954	Tübingen	1.German Center for Neurodegenerative Diseases (DZNE), Tübingen, Germany<br>2.Hertie-Institute for Clinical Brain Research, University of Tübingen, Tübingen, Germany		Site PI/Coordinator of DIAN in Germany

Laske	Christoph	954	Tübingen	1.German Center for Neurodegenerative Diseases (DZNE), Tübingen, Germany 2.Hertie-Institute for Clinical Brain Research, University of Tübingen, Tübingen, Germany 3. Department of Psychiatry and Psychotherapy, University of Tübingen, Tübingen, Germany		Clinical Lead
Forkavets	Oksana	954	Tübingen	1. German Center for Neurodegenerative Diseases (DZNE), Tübingen, Germany 2. Hertie-Institute for Clinical Brain Research, University of Tübingen, Tübingen, Germany		Study Physician
Spring	Beatrice	954	Tübingen	1. German Center for Neurodegenerative Diseases (DZNE), Tübingen, Germany 2. Hertie-Institute for Clinical Brain Research, University of Tübingen, Tübingen, Germany		Study Nurse/Study Coordinator
Graber-Sultan	Susanne	954	Tübingen	1. German Center for Neurodegenerative Diseases (DZNE), Tübingen, Germany 2. Hertie-Institute for Clinical Brain Research, University of Tübingen, Tübingen, Germany		Neuropsychologist
la Fougère	Christian	954	Tübingen	1. German Center for Neurodegenerative Diseases (DZNE), Tübingen, Germany 2. Department of Nuclear Medicine and Clinical Molecular Imaging, University of Tübingen, Tübingen, Germany		Imaging
Reischl	Gerald	954	Tübingen	1. German Center for Neurodegenerative Diseases (DZNE), Tübingen, Germany 2. Werner Siemens Imaging Center, Department of Preclinical Imaging and Radiopharmacy, University of Tübingen, Tübingen, Germany		Radiopharmacy
Obermueller	Ulrike	954	Tubingen	1. German Center for Neurodegenerative Diseases (DZNE), Tübingen, Germany 2. Hertie-Institute for Clinical Brain Research, University of Tübingen, Tübingen, Germany		Research Assistance(biospecimen/biomarker)
Levin	Johannes	955	Munich	1) German Center for Neurodegenerative Diseases, site Munich; 2) Department of Neurology, Ludwig-Maximilians-Universität München, Munich, Germany; 3) Munich Cluster for Systems Neurology (SyNergy), Munich, Germany		Site PI
Rude	Ilona	955	Munich	1) German Center for Neurodegenerative Diseases, site Munich		Site Coordinator
Vöglein	Jonathan	955	Munich	1. Department of Neurology, LMU University Hospital, LMU Munich, Munich, Germany 2. German Center for Neurodegenerative Diseases (DZNE), Munich, Germany 3.Munich Cluster for Systems Neurology (SyNergy, Munich, Germany		Site PI
Lee	Jae-Hong	957	Seoul	Asan Medical Center, Department of Neurology, Seoul, Republic of Korea		Site PI

E. DIAN Obs Consortium Author List

Roh	Jee Hoon	957	Seoul	1. Korea University Anam Hospital, Department of Neurology, Seoul, Republic of Korea 2.Korea University College of Medicine, Department of Physiology and Department of Biomedical Sciences, Seoul, Republic of Korea		Secondary Site PI
Vitali	Paolo	963	McGill	McGill University Research Centre for Studies on Aging Douglas Mental Health University Institute Department of Neurology and Neurosurgery, McGill University, Montreal, Canada		Site PI
Allegri	Ricardo F.	956/966	FLENI/Salta	Instituto Neurológico Fleni, Buenos Aires, Argentina		Site PI
Chrem Mendez	Patricio	956	FLENI	Instituto Neurológico Fleni, Buenos Aires, Argentina		Site Sub PI
Surace	Ezequiel	956	FLENI	Instituto Neurológico Fleni, Buenos Aires, Argentina, Department of Molecular Biology and Neuropathology,		
Vigo	Gabriela	966	Salta	Instituto Neurológico Fleni, Buenos Aires, Argentina		Site Coordinator
Aguillon	David	968	Medellin	Grupo de Neurociencias de Antioquia (GNA), Facultad de Medicina, Universidad de Antioquia, Medellín, Colombia.		Site PI
Guerrero	Alejandro	968	Medellin	Grupo de Neurociencias de Antioquia (GNA), Facultad de Medicina, Universidad de Antioquia, Medellín, Colombia.		Sub-Investigator
Leon	Yudy Milena	968	Medellin	Grupo de Neurociencias de Antioquia (GNA), Facultad de Medicina, Universidad de Antioquia, Medellín, Colombia.		Research Coordinator
Ramirez	Laura	968	Medellin	Grupo de Neurociencias de Antioquia (GNA), Facultad de Medicina, Universidad de Antioquia, Medellín, Colombia.		Sub-Investigator
Serna	Laura	968	Medellin	Grupo de Neurociencias de Antioquia (GNA), Facultad de Medicina, Universidad de Antioquia, Medellín, Colombia.		Lab Lead
Bocanegra	Yamile	968	Medellin	Grupo de Neurociencias de Antioquia (GNA), Facultad de Medicina, Universidad de Antioquia, Medellín, Colombia.		Neuropsychologist
Levey	Allan I.		Emory	Goizueta Alzheimer’s Disease Research Center, Department of Neurology, Emory University, Atlanta, GA 30329	Project 3 Leader	
Johnson	Erik C.B		Emory	Goizueta Alzheimer’s Disease Research Center, Emory University, Atlanta, GA 30329	Project 3	
Seyfried	Nicholas T.		Emory	Goizueta Alzheimer’s Disease Research Center, Emory University, Atlanta, GA 30329	Project 3	
Ringman	John		University of Southern California	Department of Neurology, Keck School of Medicine of USC, Universty of Southern California		
Fagan	Anne M.		Washington University	Department of Neurology, Washington University in St. Louis		
Mori	Hiroshi			Osaka Metropolitan University		
Masters	Colin		University of Melbourne	Florey Institute, The University of Melbourne		
Noble	James M.		Columbia University	Taub Institute for Research on Alzheimer's Disease and the Aging Brain, G.H. Sergievsky Center, Department of Neurology, Columbia University Irving Medical Center		

E. DIAN Obs Consortium Author List

Sanchez-Valle	Raquel	962	Barcelona	Alzheimer's disease and other cognitive disorders group. Neurology Service. Hospital Clínic de Barcelona. FRCB-IDIBAPS. University of Barcelona, Barcelona (Spain)		
Lopera	Francisco	968	Medellin	Grupo de Neurociencias de Antioquia (GNA), Facultad de Medicina, Universidad de Antioquia, Medellín, Colombia.		

F. Supplementry File 3 - DIAN Obs Data Dictionaires

Washington University School of Medicine
Institute for Informatics (I2)

DIAN GENE Mutation

Codebook

Data Dictionary Codebook

12/04/2023 1:43pm

#	Variable / Field Name	Field Label *Field Note*	Field Attributes (Field Type, Validation, Choices, Calculations, etc.)
Instrument: **Dian Demographics** (dian_demographics)			
1	dian_id	DIAN ID	text, Required
2	rec_date	Date sample received? *(yyyy-mm-dd)*	text (date_ymd)

3	site_id	Site ID	dropdown<br>003 | 003, USC<br>005 | 005, UCSD<br>010 | 010, Columbia University<br>011 | 011, Washington University<br>024 | 024, University of Pittsburgh<br>035 | 035, UCLA<br>036 | 036, MAYO - Jacksonville<br>037 | 037, Indiana<br>094 | 094, Brigham & Women's<br>941 | 941, Butler<br>950 | 950, Univ. of New South Wales<br>951 | 951, Melbourne<br>952 | 952, Perth<br>953 | 953, University College - London<br>954 | 954, University of Tübingen - Tübingen Germany<br>955 | 955, LMU - Munich Germany<br>956 | 956, FLENI - Argentina<br>957 | 957, Asan Medical Center - South Korea<br>968 | 968, University of Antioquia - Medellin Colombia<br>700 | 700, Osaka City University (OCU) - Japan<br>710 | 710, Hirosaki University (HUGSM) - Japan<br>720 | 720, Niigata University (NIGH) - Japan<br>730 | 730, University of Tokyo (UTYO) - Japan
4	map_id	MAP ID	text (number)
5	fam_id	Family ID<br>*3 digit site - 4 digit family id: (xxx-xxxx)*	text
6	master_famid	Unifying Family ID	text
7	draw_date	Date of Draw<br>*(yyyy-mm-dd)*	text (date_ymd, Min: 2007)

8	dob_yr	Section Header: *DEMOGRAPHIC INFORMATION*<br>Year of Birth<br>*(yyyy)*	text (integer, Min: 1888, Max: 2010)
9	gender	Gender	dropdown<br>0 Female<br>1 Male
10	fam_mutation	Known Family Mutation.	dropdown<br>1 PS1<br>2 PS2<br>3 APP
11	fam_mutation_chg	Known Family Mutation Change	text
12	fam_mutation_chg_v2	Known Family Mutation Change v2	text
13	coding_dna	Coding DNA	text
14	dutch_mut	Is this the Dutch mutation?	dropdown<br>0 No<br>1 Yes
15	insertion	Insertion	notes
16	deletion	Deletion	notes
17	demographic_note	Collection Notes	notes
18	csf_buffy_rcvdate	Section Header: *CSF BUFFY*<br>Date Buffy Received<br>*(yyyy-mm-dd)*	text (date_ymd)
19	csf_buffy_rcvdate2<br>Show the field ONLY if:<br>[csf_buffy_rcvdate] >= '2009-01-01'	Date Buffy Received (2)<br>*(yyyy-mm-dd)*	text (date_ymd)
20	csf_buffy_rcvdate3<br>Show the field ONLY if:<br>[csf_buffy_rcvdate] >= '2009-01-01' and [csf_buffy_rcvdate2] >= '2009-01-01'	Date Buffy Received (3)<br>*(yyyy-mm-dd)*	text (date_ymd)

21	csf_buffy_rcvdate4<br><br>Show the field ONLY if:<br>[csf_buffy_rcvdate] >= '2009-01-01' and [csf_buffy_rcvdate2] >= '2009-01-01' and [csf_buffy_rcvdate3] >= '2009-01-01'	Date Buffy Received (4)<br>*(yyyy-mm-dd)*	text (date_ymd)
22	csf_buffy_rcvdate5<br><br>Show the field ONLY if:<br>[csf_buffy_rcvdate] >= '2009-01-01' and [csf_buffy_rcvdate2] >= '2009-01-01' and [csf_buffy_rcvdate3] >= '2009-01-01' and [csf_buffy_rcvdate4] >= '2009-01-01'	Date Buffy Received (5)<br>*(yyyy-mm-dd)*	text (date_ymd)
23	csf_buffy_rcvdate6<br><br>Show the field ONLY if:<br>[csf_buffy_rcvdate] >= '2009-01-01' and [csf_buffy_rcvdate2] >= '2009-01-01' and [csf_buffy_rcvdate3] >= '2009-01-01' and [csf_buffy_rcvdate4] >= '2009-01-01' and [csf_buffy_rcvdate5] >= '2009-01-01'	Date Buffy Received (6)<br>*(yyyy-mm-dd)*	text (date_ymd)
24	csf_buffy_rcvdate7<br><br>Show the field ONLY if:<br>[csf_buffy_rcvdate] >= '2009-01-01' and [csf_buffy_rcvdate2] >= '2009-01-01' and [csf_buffy_rcvdate3] >= '2009-01-01' and [csf_buffy_rcvdate4] >= '2009-01-01' and [csf_buffy_rcvdate5] >= '2009-01-01' and [csf_buffy_rcvdate6] >= '2009-01-01'	Date Buffy Received (7)<br>*(yyyy-mm-dd)*	text (date_ymd)

25	csf_buffy_rcvdate8<br><br>Show the field ONLY if:<br>[csf_buffy_rcvdate] >= '2009-01-01' and [csf_buffy_rcvdate2] >= '2009-01-01' and [csf_buffy_rcvdate3] >= '2009-01-01' and [csf_buffy_rcvdate4] >= '2009-01-01' and [csf_buffy_rcvdate5] >= '2009-01-01' and [csf_buffy_rcvdate6] >= '2009-01-01' and [csf_buffy_rcvdate7] >= '2009-01-01'	Date Buffy Received (8)<br>*(yyyy-mm-dd)*	text (date_ymd)
26	csf_buffy_rcvdate9<br><br>Show the field ONLY if:<br>[csf_buffy_rcvdate] >= '2009-01-01' and [csf_buffy_rcvdate2] >= '2009-01-01' and [csf_buffy_rcvdate3] >= '2009-01-01' and [csf_buffy_rcvdate4] >= '2009-01-01' and [csf_buffy_rcvdate5] >= '2009-01-01' and [csf_buffy_rcvdate6] >= '2009-01-01' and [csf_buffy_rcvdate7] >= '2009-01-01' and [csf_buffy_rcvdate8] >= '2009-01-01'	Date Buffy Received (9)<br>*(yyyy-mm-dd)*	text (date_ymd)

27	csf_buffy_rcvdate10<br><br>Show the field ONLY if:<br>[csf_buffy_rcvdate] >= '2009-01-01' and [csf_buffy_rcvdate2] >= '2009-01-01' and [csf_buffy_rcvdate3] >= '2009-01-01' and [csf_buffy_rcvdate4] >= '2009-01-01' and [csf_buffy_rcvdate5] >= '2009-01-01' and [csf_buffy_rcvdate6] >= '2009-01-01' and [csf_buffy_rcvdate7] >= '2009-01-01' and [csf_buffy_rcvdate8] >= '2009-01-01' and [csf_buffy_rcvdate9] >= '2009-01-01'	Date Buffy Received (10)<br>*(yyyy-mm-dd)*	text (date_ymd)
28	fibro_rcvdate	Section Header: *BIOPSY*<br><br>Date Skin Biopsy for Fibroblast Received<br>*(yyyy-mm-dd)*	text (date_ymd)
29	fibro_cell_line	Fibroblast cell line successful?	dropdown<br>0 NO<br>1 YES
30	pax_gene_rcvdate1	Section Header: *PAX Gene*<br><br>PAX Gene Tube Received (1)<br>*(yyyy-mm-dd)*	text (date_ymd)
31	pax_gene_rcvdate2<br><br>Show the field ONLY if:<br>[pax_gene_rcvdate1] > '2009-01-01'	PAX Gene Tube Received (2)<br>*(yyyy-mm-dd)*	text (date_ymd)
32	pax_gene_rcvdate3<br><br>Show the field ONLY if:<br>[pax_gene_rcvdate2] > '2009-01-01'	PAX Gene Tube Received (3)<br>*(yyyy-mm-dd)*	text (date_ymd)
33	pax_gene_rcvdate4<br><br>Show the field ONLY if:<br>[pax_gene_rcvdate3] > '2009-01-01'	PAX Gene Tube Received (4)<br>*(yyyy-mm-dd)*	text (date_ymd)

34	pax_gene_rcvdate5<br>Show the field ONLY if: [pax_gene_rcvdate4] > '2009-01-01'	PAX Gene Tube Received (5)<br>*(yyyy-mm-dd)*	text (date_ymd)
35	pax_gene_rcvdate6<br>Show the field ONLY if: [pax_gene_rcvdate5] > '2009-01-01'	PAX Gene Tube Received (6)<br>*(yyyy-mm-dd)*	text (date_ymd)
36	pax_gene_rcvdate7<br>Show the field ONLY if: [pax_gene_rcvdate6] > '2009-01-01'	PAX Gene Tube Received (7)<br>*(yyyy-mm-dd)*	text (date_ymd)
37	pax_gene_rcvdate8<br>Show the field ONLY if: [pax_gene_rcvdate7] > '2009-01-01'	PAX Gene Tube Received (8)<br>*(yyyy-mm-dd)*	text (date_ymd)
38	pax_gene_rcvdate9<br>Show the field ONLY if: [pax_gene_rcvdate8] > '2009-01-01'	PAX Gene Tube Received (9)<br>*(yyyy-mm-dd)*	text (date_ymd)
39	pax_gene_rcvdate10<br>Show the field ONLY if: [pax_gene_rcvdate9] > '2009-01-01'	PAX Gene Tube Received (10)<br>*(yyyy-mm-dd)*	text (date_ymd)
40	braintissue	Section Header: *Brain Tissue*<br>Was Brain Tissue received?	dropdown<br>0 No<br>1 Yes
41	braintissuenotes	Notes on Brain Tissue.	notes
42	dian_demographics_complete	Section Header: *Form Status*<br>Complete?	dropdown<br>0 Incomplete<br>1 Unverified<br>2 Complete
Instrument: **Dian Mutations** (dian_mutations)			
43	dna_conc	Section Header: *SAMPLE DATA*<br>DNA Concentration<br>*ng/ul*	text (number, Min: 0, Max: 500)

44	volume	Total Sample Volume<br>*ml*	text (number, Min: 0, Max: 500)
45	od260_280	Optical Density (260/280)	text
46	apoe	Apoe Values<br>*(22,23,24,32,33,34,42,43,44)*	text (number, Min: 22, Max: 44)
47	gender_chk	Section Header: *MUTATION DATA*<br>Reported Gender (check)	text
48	zygosity	Genetic Gender?	dropdown<br>1 XX (Female)<br>2 XY (Male)<br>3 XO<br>4 XXY<br>5 XYY
49	mutation_aa_change_check	Reported Mutation AA Change (Check)	text
50	genfammrk	Genetic Family Marker (genetic family code)	text
51	genfamid	Genetic Family ID (Unique Genetic Family ID, e.g., PS101, PS205, APP02, etc.)Note: this will allow family grouping based upon genetic family rather than site and/or pedigree info alone.	text
52	ps1exon<br>Show the field ONLY if:<br>[fam_mutation] = 1	PS1 exon<br>*(i.e., 7)*	text (number)
53	ps1mutation<br>Show the field ONLY if:<br>[fam_mutation] = 1	PS1 Mutation?	dropdown<br>0 NO<br>1 YES
54	ps1wt<br>Show the field ONLY if:<br>([fam_mutation] = "1") AND ([ps1mutation] = "0")	PS1 Wild Type	text
55	ps2exon<br>Show the field ONLY if:<br>[fam_mutation] = 2	PS2 exon<br>*(i.e., 7)*	text (number)

56	ps2mutation<br><br>Show the field ONLY if:<br>[fam_mutation] = 2	PS2 mutation?	dropdown<br>0 NO<br>1 YES
57	ps2wt<br><br>Show the field ONLY if:<br>([fam_mutation] = "2") AND ([ps2mutation] = "0")	PS2 Wild Type	text
58	appexon<br><br>Show the field ONLY if:<br>[fam_mutation] = 3	APP exon<br>*(i.e., 7)*	text
59	appmutation<br><br>Show the field ONLY if:<br>[fam_mutation] =3	APP Mutation?	dropdown<br>0 NO<br>1 YES
60	appwt<br><br>Show the field ONLY if:<br>([fam_mutation] = "3") AND ([appmutation] = "0")	APP Wild Type	text
61	ps1_other<br><br>Show the field ONLY if:<br>([ps1mutation] = "0") OR ([ps2mutation] = "0") OR ([appmutation] = "0")	Screen Other PS1 exons?	dropdown<br>0 NO<br>1 YES
62	ps1exon_chg<br><br>Show the field ONLY if:<br>[ps1_other] = 1	PS1 Exon change	text
63	ps1aa_delta<br><br>Show the field ONLY if:<br>[ps1_other] = 1	PS1 (AA Change)<br>*(e.g., Gly217Arg)*	text
64	ps1base_delta<br><br>Show the field ONLY if:<br>[ps1_other] = 1	PS1 Base Change<br>*(e.g., agg>acg)*	text

65	ps2_other<br><br>Show the field ONLY if:<br>([ps1mutation] = "0") OR ([ps2mutation] = "0") OR ([appmutation] = "0")	Screen Other PS2 exons?	dropdown<br>0 NO<br>1 YES
66	ps2exon_chg<br><br>Show the field ONLY if:<br>[ps2_other] = 1	PS2 Exon change	text
67	ps2aa_delta<br><br>Show the field ONLY if:<br>[ps2_other] = 1	PS2 (AA Change)<br>*(e.g., Gly217Arg)*	text
68	ps2base_delta<br><br>Show the field ONLY if:<br>[ps2_other] = 1	PS2 Base Change<br>*(e.g., agg>acg)*	text
69	app_other<br><br>Show the field ONLY if:<br>([ps1mutation] = "0") OR ([ps2mutation] = "0") OR ([appmutation] = "0")	Screen Other APP exons?	dropdown<br>0 NO<br>1 YES
70	appexon_chg<br><br>Show the field ONLY if:<br>[app_other] = 1	APP Exon change	text
71	appaa_delta<br><br>Show the field ONLY if:<br>[app_other] = 1	APP (AA Change)<br>*(e.g., Gly217Arg)*	text
72	appbase_delta<br><br>Show the field ONLY if:<br>[app_other] = 1	APP Base Change<br>*(e.g., agg>acg)*	text
73	bdnf_assay_type	Section Header: *Other Genetics Data*<br><br>BDNF assay type	dropdown<br>01 Exome Array<br>02 TaqMan Assay

74	bdnf_rs6265_genotype	BDNF rs6265 Genotype	dropdown<br>1 AA<br>2 AG<br>3 GG
75	mut_confirm	Section Header: *Mutation Confirmation Information*<br>Mutation Status Confirmed?<br>*If answer is NO then please explain in Comment box below.*	dropdown<br>0 NO<br>1 YES
76	mut_confirm_source	Mutation Confirmation Source	dropdown<br>1 NCRAD<br>2 Fasted Blood
77	apoe_confirm	APOE Confirmed?<br>*If answer is NO then please explain in Comment box below.*	dropdown<br>0 NO<br>1 YES
78	apoe_confirm_source	APOE Confirmation Source	dropdown<br>1 NCRAD<br>2 Fasted Blood
79	gender_confirm	Gender Confirmed?<br>*If answer is NO then please explain in Comment box below.*	dropdown<br>0 NO<br>1 YES
80	gender_confirm_source	Gender Confirmation Source	dropdown<br>1 NCRAD<br>2 Fasted Blood
81	notes	Comments/Notes	notes
82	dian_mutations_complete	Section Header: *Form Status*<br>Complete?	dropdown<br>0 Incomplete<br>1 Unverified<br>2 Complete

DIAN Obs - DF17 Fluid Biomarker Core Data Dictionary

Variable Name	Variable Label	text
ACCESSION_ID	ACCESSION_ID	Unique ID for each sample in the Fluid Biomarker Core Group, generated by Freezerworks software.
DIAN_ID	DIAN_ID	DIAN Observational Study ID for each participant
LP_DATE	LP_DATE	Date of lumbar puncture/CSF collection
LUMIPULSE_CSF_Assay_Date	LUMIPULSE_CSF_Assay_Date	Date of Lumipulse CSF assay.
LUMIPULSE_CSF_AB40	LUMIPULSE_CSF_AB40	CSF AB40 analyte (pg/ml) determination by Lumipulse. If empty no reportable/quantifiable concentration available.
LUMIPULSE_CSF_AB40+QCFAILS	LUMIPULSE_CSF_AB40+QCFAILS	pg/ml quantitation threshold for either of the QC fails: below lower limit of quantitation (BLLOQ) or above upper limit of quantitation (AULOQ) for the AB40 assay.
LUMIPULSE_CSF_AB40_Comments	LUMIPULSE_CSF_AB40_Comments	Indicates a QC fail for AB40 analyte determination either by falling below lower limit of quantitation (BLLOQ) or above upper limit of quantitation (AULOQ) for the assay.
LUMIPULSE_CSF_AB40_Lot	LUMIPULSE_CSF_AB40_Lot	Lot number of Lumipulse CSF AB40 immuonreaction cartridge.
LUMIPULSE_CSF_AB42	LUMIPULSE_CSF_AB42	CSF AB42 analyte (pg/ml) determination by Lumipulse. If empty no reportable/quantifiable concentration available.
LUMIPULSE_CSF_AB42+QCFAILS	LUMIPULSE_CSF_AB42+QCFAILS	pg/ml quantitation threshold for either of the QC fails: below lower limit of quantitation (BLLOQ) or above upper limit of quantitation (AULOQ) for the AB42 assay.
LUMIPULSE_CSF_AB42_Comments	LUMIPULSE_CSF_AB42_Comments	Indicates a QC fail for AB42 analyte determination either by falling below lower limit of quantitation (BLLOQ) or above upper limit of quantitation (AULOQ) for the assay.
LUMIPULSE_CSF_AB42_Lot	LUMIPULSE_CSF_AB42_Lot	Lot number of Lumipulse CSF AB42 immuonreaction cartridge.
LUMIPULSE_CSF_pTau	LUMIPULSE_CSF_pTau	CSF pTau analyte (pg/ml) determination by Lumipulse. If empty no reportable/quantifiable concentration available.
LUMIPULSE_CSF_pTau+QCFAILS	LUMIPULSE_CSF_pTau+QCFAILS	pg/ml quantitation threshold for either of the QC fails: below lower limit of quantitation (BLLOQ) or above upper limit of quantitation (AULOQ) for the pTAU assay.
LUMIPULSE_CSF_pTau_Comments	LUMIPULSE_CSF_pTau_Comments	Indicates a QC fail for pTau analyte determination either by falling below lower limit of quantitation (BLLOQ) or above upper limit of quantitation (AULOQ) for the assay.
LUMIPULSE_CSF_pTau_Lot	LUMIPULSE_CSF_pTau_Lot	Lot number of Lumipulse CSF pTau immuonreaction cartridge.
LUMIPULSE_CSF_tTau	LUMIPULSE_CSF_tTau	CSF tTau analyte (pg/ml) determination by Lumipulse. If empty no reportable/quantifiable concentration available.
LUMIPULSE_CSF_tTau+QCFAILS	LUMIPULSE_CSF_tTau+QCFAILS	pg/ml quantitation threshold for either of the QC fails: below lower limit of quantitation (BLLOQ) or above upper limit of quantitation (AULOQ) for the tTAU assay.
LUMIPULSE_CSF_tTau_Comments	LUMIPULSE_CSF_tTau_Comments	Indicates a QC fail for tTau analyte determination either by falling below lower limit of quantitation (BLLOQ) or above upper limit of quantitation (AULOQ) for the assay.
LUMIPULSE_CSF_tTau_Lot	LUMIPULSE_CSF_tTau_Lot	Lot number of Lumipulse CSF tTau immuonreaction cartridge.

DIAN Obs - DF17 Fluid Biomarker Core Data Dictionary

Variable Name	Variable Label	text
ACCESSION_ID_bg04	ACCESSION_ID_bg04	Unique ID for each sample in the Fluid Biomarker Core, generated by Freezerworks software. Specifically identifies the third bridging sample set (04) run by the Biomarker Core.
DIAN_ID_bg04	DIAN_ID_bg04	DIAN Observational Study ID for each participant. Specifically identifies the third bridging sample set (04) run by the Biomarker Core.
LP_DATE_bg04	LP_DATE_bg04	Date of lumbar puncture/CSF collection. Specifically identifies the third bridging sample set (04) run by the Biomarker Core.
LUMIPULSE_CSF_Assay_Date_bg04	LUMIPULSE_CSF_Assay_Date_bg04	Date of Lumipulse CSF assay. Specifically identifies the third bridging sample set (04) run by the Biomarker Core.
LUMIPULSE_CSF_AB40_bg04	LUMIPULSE_CSF_AB40_bg04	CSF AB40 analyte (pg/ml) determination by Lumipulse. If empty no reportable/quantifiable concentration available. Specifically identifies the third bridging sample set (04) run by the Biomarker Core.
LUMIPULSE_CSF_AB40_QCFAILS_bg04	LUMIPULSE_CSF_AB40_QCFAILS_bg04	pg/ml quantitation threshold for either of the QC fails: below lower limit of quantitation (BLLOQ) or above upper limit of quantitation (AULOQ) for the AB40 assay. Specifically identifies the third bridging sample set (04) run by the Biomarker Core.
LUMIPULSE_CSF_AB40_Comments_bg04	LUMIPULSE_CSF_AB40_Comments_bg04	Indicates a QC fail for AB40 analyte determination either by falling below lower limit of quantitation (BLLOQ) or above upper limit of quantitation (AULOQ) for the assay. Specifically identifies the third bridging sample set (04) run by the Biomarker Core.
LUMIPULSE_CSF_AB40_Lot_bg04	LUMIPULSE_CSF_AB40_Lot_bg04	Lot number of Lumipulse CSF AB40 immuonreaction cartridge. Specifically identifies the third bridging sample set (04) run by the Biomarker Core.
LUMIPULSE_CSF_AB42_bg04	LUMIPULSE_CSF_AB42_bg04	CSF AB42 analyte (pg/ml) determination by Lumipulse. If empty no reportable/quantifiable concentration available. Specifically identifies the third bridging sample set (04) run by the Biomarker Core.
LUMIPULSE_CSF_AB42_QCFAILS_bg04	LUMIPULSE_CSF_AB42_QCFAILS_bg04	pg/ml quantitation threshold for either of the QC fails: below lower limit of quantitation (BLLOQ) or above upper limit of quantitation (AULOQ) for the AB42 assay. Specifically identifies the third bridging sample set (04) run by the Biomarker Core.
LUMIPULSE_CSF_AB42_Comments_bg04	LUMIPULSE_CSF_AB42_Comments_bg04	Indicates a QC fail for AB42 analyte determination either by falling below lower limit of quantitation (BLLOQ) or above upper limit of quantitation (AULOQ) for the assay. Specifically identifies the third bridging sample set (04) run by the Biomarker Core.
LUMIPULSE_CSF_AB42_Lot_bg04	LUMIPULSE_CSF_AB42_Lot_bg04	Lot number of Lumipulse CSF AB42 immuonreaction cartridge. Specifically identifies the third bridging sample set (04) run by the Biomarker Core.
LUMIPULSE_CSF_tTau_bg04	LUMIPULSE_CSF_tTau_bg04	CSF pTau analyte (pg/ml) determination by Lumipulse. If empty no reportable/quantifiable concentration available. Specifically identifies the third bridging sample set (04) run by the Biomarker Core.
LUMIPULSE_CSF_tTau_QCFAILS_bg04	LUMIPULSE_CSF_tTau_QCFAILS_bg04	pg/ml quantitation threshold for either of the QC fails: below lower limit of quantitation (BLLOQ) or above upper limit of quantitation (AULOQ) for the pTAU assay. Specifically identifies the third bridging sample set (04) run by the Biomarker Core.
LUMIPULSE_CSF_tTau_Comments_bg04	LUMIPULSE_CSF_tTau_Comments_bg04	Indicates a QC fail for pTau analyte determination either by falling below lower limit of quantitation (BLLOQ) or above upper limit of quantitation (AULOQ) for the assay. Specifically identifies the third bridging sample set (04) run by the Biomarker Core.
LUMIPULSE_CSF_tTau_Lot_bg04	LUMIPULSE_CSF_tTau_Lot_bg04	Lot number of Lumipulse CSF pTau immuonreaction cartridge. Specifically identifies the third bridging sample set (04) run by the Biomarker Core.
LUMIPULSE_CSF_pTau_bg04	LUMIPULSE_CSF_pTau_bg04	CSF tTau analyte (pg/ml) determination by Lumipulse. If empty no reportable/quantifiable concentration available. Specifically identifies the third bridging sample set (04) run by the Biomarker Core.
LUMIPULSE_CSF_pTau_QCFAILS_bg04	LUMIPULSE_CSF_pTau_QCFAILS_bg04	pg/ml quantitation threshold for either of the QC fails: below lower limit of quantitation (BLLOQ) or above upper limit of quantitation (AULOQ) for the tTAU assay. Specifically identifies the third bridging sample set (04) run by the Biomarker Core.
LUMIPULSE_CSF_pTau_Comments_bg04	LUMIPULSE_CSF_pTau_Comments_bg04	Indicates a QC fail for tTau analyte determination either by falling below lower limit of quantitation (BLLOQ) or above upper limit of quantitation (AULOQ) for the assay. Specifically identifies the third bridging sample set (04) run by the Biomarker Core.
LUMIPULSE_CSF_pTau_Lot_bg04	LUMIPULSE_CSF_pTau_Lot_bg04	Lot number of Lumipulse CSF tTau immuonreaction cartridge. Specifically identifies the third bridging sample set (04) run by the Biomarker Core.

DIAN COGCORE DATA CODEBOOK

Version 1.7

Table of Contents

eCOA TESTS** _______________ *3

Mini-Mental State Examination (MMSE) _______________ 3

DIAN Memory Complaint Questionnaire (MAC-Q) _______________ 3

Free and Cued Selective Reminding Test with Immediate Recall Form A (Grapes) _______________ 4

Free and Cued Selective Reminding Test with Immediate Recall Form B (Spider) _______________ 5

Free and Cued Selective Reminding Test with Immediate Form C (Bear) _______________ 6

Wechsler Memory Scale – (WMS-R) – Logical Memory I (Immediate) _______________ 7

Alternate Paragraph For Logical Memory I - Version A (Immediate) _______________ 7

Alternate Paragraph For Logical Memory I - Version B (Immediate) _______________ 8

Category Fluency (CFT) _______________ 8

Wechsler Adult Intelligence Scale – WAIS-R (DSST) _______________ 9

Trails A and B _______________ 9

Digit Span _______________ 10

Wechsler Memory Scale – (WMS-R) – Logical Memory II (Delayed) _______________ 11

Alternate Paragraph For Logical Memory II - Version A (Delayed) _______________ 11

Alternate Paragraph For Logical Memory II - Version B (Delayed) _______________ 12

***PEN AND PAPER TESTS** _______________ 13*

MINI MENTAL STATE EXAM _______________ 13

WMS-R LOGICAL MEMORY IA - IMMEDIATE _______________ 13

WMS-R LOGICAL MEMORY IIA - DELAYED _______________ 13

WMS-R DIGIT SPAN FORWARD _______________ 14

WMS-R DIGIT SPAN BACKWARD _______________ 14

CATEGORY FLUENCY - ANIMALS _______________ 15

TRAILMAKING A AND B _______________ 15

WAIS-R DIGIT SYMBOL _______________ 16

***DISCONTINUED TESTS** _______________ 17*

CATEGORY FLUENCY - VEGETABLES _______________ 17

LETTER FLUENCY-FAS _______________ 17

WORD LIST RECALL - Immediate 18
WORD LIST RECALL - Delayed 18
PAIR BINDING 19
PAPER FOLDING 19
READING SPAN 20
SPATIAL RELATIONS 21
SIMON TASK 21
SWITCHING 22
COMPUTATION SPAN 22
SEMANTIC CATEGORIZATION 23
INTERNATIONAL PERSONALITY ITEM POOL (IPIP) 24
INTERNATIONAL SHOPPING LIST TASK – IMMEDIATE* 27
INTERNATIONAL SHOPPING LIST TASK – DELAYED* 27
GROTON MAZE TIMED CHASE TEST (Chase the Target)* 28
GROTON MAZE LEARNING TEST (Find the Hidden Pathway)* 28
GROTON MAZE LEARNING TEST (Find the Hidden Pathway) DELAYED RECALL* 29
GROTON MAZE LEARNING TEST (Find the Hidden Pathway) DELAYED RECALL REVERSE* 29
DETECTION TEST (Has the Card Turned Over?)* 30
IDENTIFICATION TEST (Is the Card Red?)* 30
ONE CARD LEARNING TEST (Have You Seen this Card Before?)* 31
ONE-BACK MEMORY (Is the Card the Same as the Previous Card?)* 31
BOSTON NAMING TEST - 30 (ODD NUMBERED ITEMS) 32
MULTILINGUAL NAMING TEST 33
VERSION HISTORY *34*

eCOA TESTS*

Mini-Mental State Examination (MMSE)

Date Added: 2023 (Upon Amendment 9 Approval)

Version: Ver. 4.0 27Dec2017 Reproduced by special permission of the Publisher, Psychological Assessment Resources, Inc., 16204 North Florida Avenue, Lutz, Florida 33549, from the Mini Mental State Examination, by Marshal Folstein and Susan Folstein, AU1.0. 14Jul2014

Copyright: Copyright 1975,1998, 2001 by Mini Mental LLC, Inc. Published 2001 by Psychological Assessment Resources, Inc

Author: Folstein MF; Folstein SE; McHugh PR

MMSETOT Total Score: This score is used to evaluate the overall capabilities of the patient.

Range: 0 - 30 High score = good

DIAN Memory Complaint Questionnaire (MAC-Q)

Date Added: 2023 (Upon Amendment 9 Approval)

Version: Adapted from Crook, T.H, Feher, E. P., and Larrabee, G. J. (1992). Assessment of Memory Complaint in Age- Associated Memory Impairment: the MAC-Q. International Psychogeriatrics, Vol 4, No. 2, 165-176.

Copyright: ADAPTED FROM CROOK, T. H., FEHER, E. P., & LARRABEE, G. J. (1992). ASSESSMENT OF MEMORY COMPLAINT IN AGE-ASSOCIATED MEMORY IMPAIRMENT: THE MAC-Q. INTERNATIONAL PSYCHOGERIATRICS, 4(2), 165–176 Cambridge University Press

Author: T.H. Crook, E.P. Feher, G.J. Larrabee

MACQTOT Total Score: Used by clinicians to determine a patient's memory as compared to when they were young.

Range: 7-35 High Score = bad

FREE AND CUED SELECTIVE REMINDING TEST WITH IMMEDIATE RECALL FORM A (GRAPES)

Date Added: 2023 (Upon Amendment 9 Approval)

Version: International research version FCSRT-IR with delayed recall. Form A (Grapes)

Copyright: FCSRT© Albert Einstein College of Medicine, 1996-2000. All rights reserved.

Author: Buschke H; Grober E

Free Recall: Total number of words on all four cards recalled with no prompting.

Range 0 – 16 High score = good

Trial 1 Free Recall: **FCSA158**	FCSA01-Free Recall Trial 1
Trial 2 Free Recall: **FCSA178**	FCSA01-Free Recall Trial 2
Trial 3 Free Recall: **FCSA197**	FCSA01-Free Recall Trial 3

Cued Recall: Number of words recalled after prompting with the category.

Range 0 – 16 High score = good

Trial 1 Cued Recall: **FCSA175**	FCSA01-Cued Recall Trial 1
Trial 2 Cued Recall: **FCSA195**	FCSA01-Cued Recall Trial 2
Trial 3 Cued Recall: **FCSA214**	FCSA01-Cued Recall Trial 3

Total Recall: Total number of words on all four cards recalled either with no prompting, or after prompting with the category.

Range 0 – 16 High score = good

Trial 1 Total Recall: **FCSA176**	FCSA01-Total Recall Trial 1
Trial 2 Total Recall: **FCSA195b**	FCSA01-Total Recall Trial 2
Trial 3 Total Recall: **FCSA215**	FCSA01-Total Recall Trial 3

FREE AND CUED SELECTIVE REMINDING TEST WITH IMMEDIATE RECALL FORM B (SPIDER)

Date Added: 2023 (Upon Amendment 9 Approval)

Version: International research version FCSRT-IR with delayed recall. Form B (Spider)

Copyright: FCSRT© Albert Einstein College of Medicine, 1996-2000. All rights reserved.

Author: Buschke H; Grober E

Free Recall: Total number of words on all four cards recalled with no prompting.

Range 0 – 16 High score = good

Trial 1 Free Recall: FCSB158 FCSB01-Free Recall Trial 1
Trial 2 Free Recall: FCSB178 FCSB01-Free Recall Trial 2
Trial 3 Free Recall: FCSB198 FCSB01-Free Recall Trial 3

Cued Recall: Number of words recalled after prompting with the category.

Range 0 – 16 High score = good

Trial 1 Cued Recall: FCSB175 FCSB01-Cued Recall Trial 1
Trial 2 Cued Recall: FCSB195 FCSB01-Cued Recall Trial 2
Trial 3 Cued Recall: FCSB215 FCSB01-Cued Recall Trial 3

Total Recall: Total number of words on all four cards recalled either with no prompting, or after prompting with the category.

Range 0 – 16 High score = good

Trial 1 Total Recall: FCSB176 FCSB01-Total Recall Trial 1
Trial 2 Total Recall: FCSB196 FCSB01-Total Recall Trial 2
Trial 3 Total Recall: FCSB216 FCSB01-Total Recall Trial 3

FREE AND CUED SELECTIVE REMINDING TEST WITH IMMEDIATE FORM C (BEAR)

Date Added: 2023 (Upon Amendment 9 Approval)

Version: International research version FCSRT-IR with delayed recall. Form C (Bear)

Copyright: FCSRT© Albert Einstein College of Medicine, 1996-2000. All rights reserved.

Author: Buschke H; Grober E

Free Recall: Total number of words on all four cards recalled with no prompting.

Range 0 – 16 High score = good

Trial 1 Free Recall: **FCSC157** FCSC01-Free Recall Trial 1
Trial 2 Free Recall: **FCSC177** FCSC01-Free Recall Trial 2
Trial 3 Free Recall: **FCSC197** FCSC01-Free Recall Trial 3

Cued Recall: Number of words recalled after prompting with the category.

Range 0 – 16 High score = good

Trial 1 Cued Recall: **FCSC174** FCSC01-Cued Recall Trial 1
Trial 2 Cued Recall: **FCSC194** FCSC01-Cued Recall Trial 2
Trial 3 Cued Recall: **FCSC214** FCSC01-Cued Recall Trial 3

Total Recall: Total number of words on all four cards recalled either with no prompting, or after prompting with the category.

Range 0 – 16 High score = good

Trial 1 Total Recall: **FCSC175** FCSC01-Total Recall Trial 1
Trial 2 Total Recall: **FCSC195** FCSC01-Total Recall Trial 2
Trial 3 Total Recall: **FCSC215** FCSC01-Total Recall Trial 3

WECHSLER MEMORY SCALE – (WMS-R) – LOGICAL MEMORY I (IMMEDIATE)

Date Added: 2023 (Upon Amendment 9 Approval)

Version: Story A (Anna Thompson),

Copyright: Copyright ©1945, renewed 1974, 1987 NCS Pearson, Inc. Adapted and reproduced with permission of publisher NCS Pearson, inc. All rights reserved.

Author: David Wechsler, PhD

LOGIMEM Total Score: Overall total of all story units recalled correctly.

Range 0 – 25 High score = good

ALTERNATE PARAGRAPH FOR LOGICAL MEMORY I - VERSION A (IMMEDIATE)

Date Added: 2023 (Upon Amendment 9 Approval)

Version: Greg Fortune

Copyright: Morris, J., Kunka, J.M., and Rossini, E.D. (1997). Alternate paragraphs for the Logical Memory subtest of the Wechsler scale-revised. The Clinical Neuropsychologist, 11 (4).

Author: Morris, J., Kunka, J.M., and Rossini, E.D.

LOGIMEMA Total Score: Overall total of all story units recalled correctly.

Range 0 – 25 High score = good

ALTERNATE PARAGRAPH FOR LOGICAL MEMORY I - VERSION B (IMMEDIATE)

Date Added: 2023 (Upon Amendment 9 Approval)

Version: Martha Jackson

Copyright: Morris, J., Kunka, J.M., and Rossini, E.D. (1997). Alternate paragraphs for the Logical Memory subtest of the Wechsler Scale-Revised. The Clinical Neuropsychologist, 11 (4).

Author: Morris, J., Kunka, J.M., and Rossini, E.D.

LOGIMEMB Total Score: Overall total of all story units recalled correctly.

Range 0 – 25 High score = good

CATEGORY FLUENCY (CFT)

Date Added: 2023 (Upon Amendment 9 Approval)

Version: Animals Acevedo A, Loewenstein DA, Barker WW, Harwood DG, Luis C, Bravo M, Hurwitz DA, Aguero H,

Copyright: Public Domain

Author: Acevedo A, Loewenstein DA, Barker WW, Hardwood DG, Luis C, Bravo M, Hurwitz DA, Aguero H, Greenfield L, Duara R.

ANIMALS Number of acceptable words recalled

Range: 0 and above High score = Good

CF0103 Number of Intrusion Errors

Range: 0 and above High score = Bad

CF0104 Number of Perseverative Errors

Range: 0 and above High score = Bad

WECHSLER ADULT INTELLIGENCE SCALE – WAIS-R (DSST)

Date Added: 2023 (Upon Amendment 9 Approval)

Version: N/A

Copyright: Copyright© 1981 NCS Pearson, Inc. Adapted and reproduced with permission of publisher. All rights reserved.

Author: David Wechsler

**WAIS**	Total Score: The total raw score is the number of correctly filled in items.	
	Range 0 – 93	High score = Good

TRAILS A AND B

Date Added: 2023 (Upon Amendment 9 Approval)

Version: N/A

Copyright: Public Domain

Author: Reitan RM

**TRAILA**	Trails A – Time to completion in total seconds	
	Range 0 – 150	High score = Bad
**TRAILARR**	Trails A – Total number of commission errors	
	Range 0 and above	High score = Bad
**TRAILB**	Trails B – Time to completion in total seconds	
	Range 0 – 300	High score = Bad

TRAILBRR Trails B – Total number of commission errors

Range 0 and above High score = Bad

DIGIT SPAN

Date Added: 2023 (Upon Amendment 9 Approval)

Version: Digit Forward, Digit Backward

Copyright: Copyright © 1945, renewed 1974, 1987 NCS Pearson, Inc.

Author: David Wechsler, PhD

DIGIF Digit Forward Scoring: Each response is given a score of 0 for incorrect or 1 for correct. This total is the sum of all the responses for the digit forward test.

Range 0 – 12 High score = Good

DIGIB Digit Backward Scoring: Each response is given a score of 0 for incorrect or 1 for correct. This total is the sum of all the responses for the digit backward test.

Range 0 – 12 High score = Good

DIGIFLEN Digit Forward Length Scoring: Each response is given a digit for the length of the sequence answered correctly.

Range 0 – 8 High score = Good

DIGIBLEN Digit Backward Length Scoring: Each response is given a digit for the length of the sequence answered correctly.

Range 0 - 7 High score = Good

WECHSLER MEMORY SCALE – (WMS-R) – LOGICAL MEMORY II (DELAYED)

Date Added: 2023 (Upon Amendment 9 Approval)

Version: Story A (Anna Thompson)

Copyright: Copyright ©1945, renewed 1974, 1987 NCS Pearson, Inc. Adapted and reproduced with permission of publisher NCS Pearson, Inc. All rights reserved

Author: David Wechsler, PhD

MEMUNITS Overall total of all story units recalled correctly.

Range 0 – 25 High score = good

ALTERNATE PARAGRAPH FOR LOGICAL MEMORY II - VERSION A (DELAYED)

Date Added: 2023 (Upon Amendment 9 Approval)

Version: Greg Fortune

Copyright: Morris, J., Kunka, J.M., and Rossini, E.D. (1997). Alternate paragraphs for the Logical Memory subtest of the Wechsler Scale-Revised. The Clinical Neuropsychologist, 11 (4).

Author: Morris, J., Kunka, J.M., and Rossini, E.D.

MEMUNITSA Overall total of all story units recalled correctly.

Range 0 – 25 High score = good

ALTERNATE PARAGRAPH FOR LOGICAL MEMORY II - VERSION B (DELAYED)

Date Added: 2023 (Upon Amendment 9 Approval)

Version: Martha Jackson

Copyright: Morris, J., Kunka, J.M., and Rossini, E.D. (1997). Alternate paragraphs for the Logical Memory subtest of the Wechsler Scale-Revised. The Clinical Neuropsychologist, 11 (4).

Author: Morris, J., Kunka, J.M., and Rossini, E.D.

MEMUNITSB	Overall total of all story units recalled correctly.	
	Range 0 – 25	High score = good

* Refer to Signant Health's Data Transfer Specifications for more detail.

PEN AND PAPER TESTS

MINI MENTAL STATE EXAM

Date Added: 1/09

Reference: Folstein, M.F., Folstein, S.E., & McHugh, P.R. (1975). Mini-mental State: A practical method for grading the cognitive state of patients for the clinicians. *Journal of Psychiatric Research, 12,* 189-198.

MMSE Scored according to the UDS guidebook.

Range: 0 - 30 High score = good

WMS-R LOGICAL MEMORY IA - IMMEDIATE

Date Added: 1/09

Reference: Wechsler, D. (1987). *Manual: Wechsler Memory Scale-Revised.* San Antonio, Texas: Psychological Corporation.

LOGIMEM Only Story A is administered. Scored according to WMS-R manual

Range: 0-25 High score = good

WMS-R LOGICAL MEMORY IIA - DELAYED

Date Added: 1/09

Reference: Wechsler, D. (1987). *Manual: Wechsler Memory Scale-Revised.* San Antonio, Texas: Psychological Corporation.

MEMUNITS Administered after WAIS-R Digit Symbol in prescribed UDS order, and scored according to WMS-R manual

Range: 0-25 High score = good

WMS-R DIGIT SPAN FORWARD

Date Added: 1/09

Reference: Wechsler, D. (1987). *Manual: Wechsler Memory Scale-Revised.* San Antonio, Texas: Psychological Corporation.

Administered according to WMS-R manual. Scored according to UDS guidebook, which yields two scores:

DIGIF Total number of trials correct prior to two consecutive errors at the same digit length

Range: 0 - 12 High score = good

DIGIFLEN Digit span forward length

Range: 0 - 8 High score = good

WMS-R DIGIT SPAN BACKWARD

Date Added: 1/09

Reference: Wechsler, D. (1987). *Manual: Wechsler Memory Scale-Revised.* San Antonio, Texas: Psychological Corporation.

Administered according to WMS-R manual. Scored according to UDS guidebook, which yields two scores:

DIGIB Total number of trials correct prior to two consecutive errors at the same digit length

Range: 0 - 12 High score = good

DIGIBLEN Digit span backward length

Range: 0 - 7 High score = good

CATEGORY FLUENCY - ANIMALS

Date Added: 1/09

Reference: Goodglass, H. & Kaplan, E. (1983). *Boston Diagnostic Aphasia Examination Booklet,* III, ORAL EXPRESSION, J. Animal Naming (Fluency in Controlled Association). Philadelphia: Lea & Febiger.

ANIMALS Participants name as many different animals as they can for 1 minute.

Range: 0 and above High score = good

TRAILMAKING A AND B

Date Added: 1/09

Reference: Armitage, S.G. (1945). An analysis of certain psychological tests used for the evaluation of brain injury. *Psychological Monographs, 60* (1, Whole No. 177), 1-48.

TRAILA The score is the number of seconds spent in connecting 25 numbered circles in sequential order. Time limit is 150 seconds.

Range: 0 - 150 High score = poor

TRAILARR Number of commission errors

The score is the number of errors of commission made while connecting 25 numbered circles in sequential order within the 150-second time limit.

Range: 0 – 40 High score = poor

TRAILALI Number of correct lines

The score is the number of lines correctly connected to 25 numbered circles in sequential order within the 150 second time limit.

Range: 0 – 24 High score = good

TRAILB The score is the number of seconds spent connecting numbered circles (1-13) to letters of the alphabet (A-L) in alternating sequential order. Time limit is 300 seconds.

Range: 0 - 300 High score = poor

TRAILBRR Number of commission errors

The score is the number of errors of commission made while connecting numbered circles (1-13) to letters of the alphabet (A-L) in alternating sequential order within the 300-second time limit.

Range: 0 – 40 High score = poor

TRAILBLI Number of correct lines

The score is the number of lines correctly connected between numbered circles (1-13) and letters of the alphabet (A-L) in alternating sequential order within the 300-second time limit.

Range: 0 – 24 High score = good

WAIS-R DIGIT SYMBOL

Date Added: 1/09

Reference: Wechsler, D. (1981). *Manual: Wechsler Adult Intelligence Scale - Revised*. New York: Psychological Corporation.

WAIS This is an enlarged Digit Symbol form that measures 15 x 24 cm rather than 9.5 x 13 cm as in the standard WAIS-R. Otherwise administered and raw scored according to WAIS-R manual.

Range: 0 - 93 High score = good

DISCONTINUED TESTS

CATEGORY FLUENCY - VEGETABLES

Date Added: 1/09 Date Discontinued: 11/17 for US sites,
4/18 for non-US sites
Never used at Japan sites

VEG Similar to Category Fluency-Animals.

Range: 0 and above High score = good

LETTER FLUENCY-FAS

Date Added: 1/09 Date Discontinued: 11/17 for US Sites
4/18 for non-US sites
Never used at Japan sites

Reference: Modeled after word fluency test developed by:
Thurstone, L. E., & Thurstone, T. G., (1949). *Examiner manual for the SRA Primary Mental Abilities Test.* Chicago: Science Research Associates.

FLUF LETTER FLUENCY LETTER F

Participants name as many words beginning with the letter F as they can for 1 minute.

Range: 0 and above High score = good

FLUA LETTER FLUENCY LETTER A

Participants name as many words beginning with the letter A as they can for 1 minute.

Range: 0 and above High score = good

FLUS LETTER FLUENCY LETTER S

Participants name as many words beginning with the letter S as they can for 1 minute.

Range: 0 and above High score = good

LETFLU FLUF + FLUA + FLUS

WORD LIST RECALL - IMMEDIATE

Date Added: 1/09 Date Discontinued: 11/17 for US Sites
4/18 for non-US sites
Never used at Japan sites

Reference: Designed for this project by David A. Balota.

Participant listens as the examiner reads one of six lists of 16 unrelated words at the rate of approximately 1 per second and then recalls as many of the 16 words as possible in any order. Participants hear a different list at subsequent assessments.

LIST Number of the list read to participants at this assessment.

Range: 1-6

WORDIM Number of words recalled.

Range: 0 - 16 High score = good

WORD LIST RECALL - DELAYED

Date Added: 1/09 Date Discontinued: 11/17 for US sites

4/18 for non-US sites
Never used at Japan sites

WORDDEL Number of words from word list recalled after delay interval.

Range: 0 - 16 High score = good

PAIR BINDING

Date Added: 1/09 Date Discontinued: 11/17 for US sites
4/18 for non-US sites
Never used at Japan sites

Reference: Naveh-Benjamin, M. (2000). Adult age differences in memory performance: Tests of an associative deficit hypothesis. *Journal of Experimental Psychology: Learning, Memory, and Cognition, 26*, 1170–1187.

Participants study a list of 24 unrelated word pairs presented one pair at a time with each pair on the screen for 3 seconds. Then participants see 36 pairs, again presented one pair at a time; 12 are intact studied pairs, 12 are rearranged pairs of studied words, and 12 are new pairs. Participants are to press the P key if the pair is an intact studied pair and Q if it is not.

INTACT Number of intact pairs correctly identified (i.e., a P response).

Range: 0 - 12 High score = good

MIXED Number of mixed (rearranged) correctly identified (i.e., a Q response).

Range: 0 - 12 High score = good

NEW Number of new pairs correctly identified (i.e., a Q response).

Range: 0 - 12 High score = good

PAPER FOLDING

(also referred to as Visual Spatial Test 2 until July 1, 2012)

Date Added: 1/09 Date Discontinued: 11/17 for US Sites
4/18 at non-US sites
Never used at Japan sites

Reference: Salthouse, T.A., Mitchell, D.R., Skovronek, E., & Babcock, R.L. (1989). Effects of adult age and working memory on reasoning and spatial abilities. *Journal of Experimental Psychology: Learning, Memory, and Cognition, 15*, 507-516.

Consider a square piece of paper that is folded from one to four times; punch a hole in the folded paper. Then unfold the paper and spread it flat. There will be an array of holes on the unfolded paper. Participants see a representation of the

unfolded paper on the computer screen. They must mentally fold the display into an object that represents the folded paper. Participants indicate their responses by choosing one of five multiple choice answers. Time limit is 10 min.

The score is the number correct.

Range: 0 - 12 High score = good

READING SPAN

Date Added: 1/09 Date Discontinued :11/17 for US sites
4/18 at non-US sites
Never used at Japan sites

Reference: Daneman, M., & Carpenter, P.A. (1980). Individual differences in working memory and reading. *Journal of Verbal Learning and Verbal Behavior, 19*, 450-466.

Participants must remember the last word of sentences presented on the computer screen while judging if the sentence makes a statement that is true or false. The number of sentences read prior to recall increases from 1 to 7 in blocks of three trials for each span length (i.e., number of sentences read prior to recall). For example, on each trial in the first block, participants read the sentence and judge if it is true or false; the next screen displays question marks and the participants immediately recall the last word of the sentence. On each trial of the second block, participants read the first sentence and judge if it is true or false, then read the second sentence and judge if it is true or false, are presented with the screen with question marks and then recall the last word of each of the two preceding sentences. For a trial to be scored as correct the order of the recalled words must be the same as the order in which the sentences were presented. The test is discontinued when participants fail to get at least two correct trials in a block of three trials. One of two scores can be used: readspan or readtot.

.

readspan Reading span length

The number of sentences in each trial for the last block of trials for which participants had at least two correct trials.

Range: 0 – 7 High score = good

readtot Reading span total correct trials

The total number of correct span trials through the block for which participants had at least two correct trials (i.e., block that determined the variable readspan).

Range: 0 – 21 High score = good

SPATIAL RELATIONS

(also referred to as **Visual Spatial Test 1**)

Date Added: 1/09 Date Discontinued from DIAN battery: 7/12

Reference: Salthouse, T.A., Mitchell, D.R., Skovronek, E., & Babcock, R.L. (1989). Effects of adult age and working memory on reasoning and spatial abilities. *Journal of Experimental Psychology: Learning, Memory, and Cognition, 15*, 507-516.

Participants see a picture of a paper display on the computer screen; they must mentally fold the display into an object. Participants indicate their response by choosing one of four multiple choice answers. Time limit is 10 min.

The score is the number correct.

Range: 0 - 20 High score = good

SIMON TASK

Date Added: 1/09 Date Discontinued: 1/19

References: Simon, J.R. (1969). Reactions toward the source of stimulation. *Journal of Experimental Psychology, 81*, 174-176.

Castel, A.D., Balota, D.A., Hutchison, K.A., Logan, J.M., & Yap, M.J. (in press). Spatial attention and response control in healthy younger and older adults and individuals with Alzheimer's disease: Evidence for disproportionate selection breakdowns in the Simon task. *Neuropsychology*.

The participants sees a large arrow pointing to the right (60 trials) or left (60 trials) on the computer and press the P key when the arrow points right and the Q key when it points left. One third of the trials represent the neutral condition; the arrows (half pointing left, half point right) are shown in the middle of the screen. One third of the trials represent the congruent condition; arrows pointing right are shown on the right side of the screen and arrows pointing left are shown on the left side of the screen. The remaining third of the trials reflect a mismatch between the direction of the arrow and the position on the screen; arrows pointing right are on the left side and arrows pointing left are on the right side. Practice trials (12 trials) are not included in the scoring.

simon Percentage correct on all 120 trials.

Range: 0 to 100 High score = good

SWITCHING

Date Added: 1/09 Date Discontinued 1/19

Reference: Rogers, R.D., & Monsell, S. (1995). Costs of a predictable switch between simple cognitive tasks. *Journal of Experimental Psychology: General, 124,* 207-231.

Participants see letter-digit pairs (e.g., N14) in the center of the screen. In the first block of 50 trials (10 practice, 40 test) they press the P key if the letter is a vowel and the Q key if it a consonant. For the next 50 trials (10 practice, 40 test) they press the P key if the digit is even and the Q key if it is odd. In the final block of 62 mixed trials (10 practice, 52 test) the instructions that are shown in the lower right and lower corners of the screen change every two trials so that the participants make consonant vowel decisions for two trials and then the odd even decisions and so forth. Practice trials are not included in the scoring.

switch Percentage correct on all 132 trials. High score = good

Range: 0 to 100 High score = good

COMPUTATION SPAN

Date Added: 1/09 Date Discontinued 1/19

Reference: Conway, A.R.A., Kane, M.J., Bunting, M.F., Hambrick, D.Z., Wilheim, O., & Engle, R.W. (2005). Working memory span tasks: A methodological review and user's guide. Psychonomic Bulletin & Review, 12, 769-786.

Participants see an addition or subtraction problem (e.g., 7 - 4 = 3) and press the P key if the answer is correct and the Q key if it is wrong. Participants are asked to remember the second number of the problem (e.g., 4). Participants then recall the second number of the equation by entering 4. There are two additional trials involving one equation. Then the number of equations in a trial increases to two (i.e., two second numbers must be recalled). There are 3 trials at each of seven span lengths (1, 2, 3, 4, 5, 6, and 7). For a trial to be scored as correct the order of the recalled numbers must be the same as the order in which the equations were presented. The test is discontinued when the participants fail to get at least two correct trials in a block of three trials.

cspan Computation span length

The number of equations in each trial for the last block of trials for which participants had at least two correct trials.

Range: 0 – 7 High score = good

cspantot Computation span total correct trials

The total number of correct span trials through the block for which participants had at least two correct trials (i.e., block that determined the variable cspan).

Range: 0 to 21 High score = good

SEMANTIC CATEGORIZATION

Date Added: 1/09 Date Discontinued 1/19

Reference: Smith, E. E., Shoben, E. J. & Rips, L. J. (1974). Structure and process in semantic memory: A featural model for semantic decisions. *Psychological Review, 1*, 214-241.

Participants read a category label (e.g., fruit) and press the P key if the word following the category label (e.g., apple) belongs to that category and the Q key if it does not (e.g., lettuce). There are 6 practice trials, which are not counted in the scoring. Then there are 80 *yes* and 80 *no* trials.

categorization

Percentage of correct responses on 160 trials.

Range: 0 to 100 High score = good

INTERNATIONAL PERSONALITY ITEM POOL (IPIP)

Date Added: 1/09 Date changed to paper version only: 1/19

Discontinued: 5/ 2021

Reference: Goldberg, L. R. (1999). A broad-bandwidth, public domain, personality inventory measuring the lower-level facets of several five-factor models. In I. Mervielde, I. Deary, F. De Fruyt, & F. Ostendorf (Eds.), *Personality Psychology in Europe*, Vol. 7 (pp. 7-28). Tilburg, The Netherlands: Tilburg University Press.

Administered and scored 120 items according to instructions on web site: http://ipip.ori.org

Factor scores

NEUR	Neuroticism factor score	
	Range: 0 - 120	High score = greater neuroticism
EXTRA	Extraversion factor score	
	Range: 0 - 120	High score = greater extraversion
OPEN	Openness factor score	
	Range: 0 - 120	High score = greater openness
AGREE	Agreeableness factor score	
	Range: 0 - 120	High score = greater agreeableness
CONSCIEN	Conscientiousness factor score	
	Range: 0 - 120	High score = greater conscientiousness

Facet scores (six for each factor): For each facet score

Range: 0 - 20 High score = more of characteristic

Neuroticism

ANX	Anxiety
ANGER	Anger
DEPRESS	Depression
SELFCON	Self-consciousness
IMMOD	Immoderation
VULNER	Vulnerability
	Extroversion
FRIEND	Friendliness
GREGAR	Gregariousness
ASSERT	Assertiveness
ACTIVE	Activity level
EXCITE	Excitement seeking
CHEER	Cheerfulness
	Openness
IMAG	Imagination
ARTIST	Artistic interests
EMOTION	Emotionality
ADVENT	Adventurousness
INTELL	Intellect
LIBERAL	Liberalism

	<u>Agreeableness</u>
TRUST	Trust
MORAL	Morality
ALTRU	Altruism
COOP	Cooperation
MODEST	Modesty
SYMPATH	Sympathy
	<u>Conscientiousness</u>
EFFIC	Self-efficacy
ORDER	Orderliness
DUTI	Dutifulness
ACHIEVE	Achievement striving
DISCIP	Self-discipline
CAUTIOUS	Cautiousness

*INTERNATIONAL SHOPPING LIST TASK – IMMEDIATE**

Date Added: 11/1/2017 for US sites
4/1/2018 for non-US Sites

Date Discontinued: 06OCT2020

Reference: Thompson, T.A.C., Wilson, P.H., Snyder, P.J., Pietrzak, R.H., David Darby, D., Maruff, P. & Herman Buschke, H. (2011). Sensitivity and Test–Retest Reliability of the International Shopping List Test in Assessing Verbal Learning and Memory in Mild Alzheimer's Disease. Archives of Clinical Neuropsychology, 26, 412–424.

Rahimi-Golkhandan, S., Maruff, P., Darby, D., & Wilson, P. (January 01, 2012). Barriers to repeated assessment of verbal learning and memory: a comparison of international shopping list task and rey auditory verbal learning test on build-up of proactive interference. Archives of Clinical Neuropsychology : the Official Journal of the National Academy of Neuropsychologists, 27, 7, 790-5.

ISL cor Summary of all correct responses for all 3 ISL trials

Range 0 – 36 High score = good

ISLT1 cor Summary of correct responses for first trial

Range 0 – 12 High score = good

ISLT2 cor Summary of correct responses for second trial

Range 0 – 12 High score = good

ISLT3 cor Summary of correct responses for third trial

Range 0 – 12 High score = good

*INTERNATIONAL SHOPPING LIST TASK – DELAYED**

Date Added: 11/1/2017 for US sites
4/1/2018 for non-US Sites
Date Discontinued: 06OCT2020

References: Thompson, T.A.C., Wilson, P.H., Snyder, P.J., Pietrzak, R.H., David Darby, D., Maruff, P. & Herman Buschke, H. (2011). Sensitivity and Test–Retest Reliability of the International Shopping List Test in Assessing Verbal Learning and Memory in Mild Alzheimer's Disease. Archives of Clinical Neuropsychology, 26, 412–424.

Rahimi-Golkhandan, S., Maruff, P., Darby, D., & Wilson, P. (January 01, 2012). Barriers to repeated assessment of verbal learning and memory: a comparison of international shopping list task and rey auditory verbal learning test on build-up of proactive interference. Archives of Clinical Neuropsychology: the Official Journal of the National Academy of Neuropsychologists, 27, 7, 790-5.

ISRL cor — Summary of correct responses recalled from list after a delay

Range 0 – 12 — High score = good

*GROTON MAZE TIMED CHASE TEST (CHASE THE TARGET)**

Date Added 11/1/2017 for US sites
4/1/2018 for non-US Sites

Date Discontinued: 06OCT2020

Reference: Pietrzak, RH, Maruff, P, Mayes, LC, Roman, SA, Sosa, JA, Snyder, PJ. (2008). An examination of the construct validity and factor structure of the Groton Maze Learning Test, a new measure of spatial working memory, learning efficiency, and error monitoring. Archives of Clinical Neurology, 23, 433-445.

GMCT mps — Number of correct moves per second.

Range 0 - 3 — High score = good

*GROTON MAZE LEARNING TEST (FIND THE HIDDEN PATHWAY)**

Date Added: 11/1/2017 for US sites
4/1/2018 for non-US Sites

Date Discontinued: 06OCT2020

Reference: Pietrzak, RH, Maruff, P, Mayes, LC, Roman, SA, Sosa, JA, Snyder, PJ. (2008). An examination of the construct validity and factor structure of the Groton Maze Learning Test, a new measure of spatial working memory, learning efficiency, and error monitoring. Archives of Clinical Neurology, 23, 433-445.

GMLter — Total number of errors made in attempting to learn the same hidden pathway on five consecutive trials at a single session. (Total number also figured for each of five trials.)

Range 0 - 999 — Low score = good

*GROTON MAZE LEARNING TEST (FIND THE HIDDEN PATHWAY) DELAYED RECALL**

Date Added: 11/1/2017 for US sites
4/1/2018 for non-US Sites

Date Discontinued: 06OCT2020

Reference: Pietrzak, RH, Maruff, P, Mayes, LC, Roman, SA, Sosa, JA, Snyder, PJ. (2008). An examination of the construct validity and factor structure of the Groton Maze Learning Test, a new measure of spatial working memory, learning efficiency, and error monitoring. Archives of Clinical Neurology, 23, 433-445.

GMR ter — Total number of errors made in remembering the maze pathway after a delay

Range 0 - 999 — Low score = good.

*GROTON MAZE LEARNING TEST (FIND THE HIDDEN PATHWAY) DELAYED RECALL REVERSE**

Date Added: 11/1/2017 for US sites
4/1/2018 for non-US Sites

Date Discontinued: 06OCT2020

Reference: Pietrzak, RH, Maruff, P, Mayes, LC, Roman, SA, Sosa, JA, Snyder, PJ. (2008). An examination of the construct validity and factor structure of the Groton Maze Learning Test, a new measure of spatial working memory, learning efficiency, and error monitoring. Archives of Clinical Neurology, 23, 433-445.

GMRV ter — Total number of errors made in attempting to learn the maze pathway backwards after a delay.

Range 0 - 999 — Low score = good

*DETECTION TEST (HAS THE CARD TURNED OVER?)**

Date Added: 11/2017 for US sites
4/1/2018 for non-US Sites

Date Discontinued: 06OCT2020

Reference: Maruff P, Thomas E, Cysique L, Brew B, Collie A, Snyder P, Pietrzak RH (2009). Validity of the CogState brief battery: Relationship to standardized tests and sensitivity to cognitive impairment in mild traumatic brain injury, schizophrenia, and AIDS dementia complex. Archives of Clinical Neuropsychology, 24, 165-178.

DET lmn — Speed of performance; mean of the log 10 transformed reaction times for correct responses.

Range 2.001-6 — Low score = good

*IDENTIFICATION TEST (IS THE CARD RED?)**

Date Added: 11/1/2017 for US sites
4/1/2018 for non-US Sites

Date Discontinued: 06OCT2020

Reference: Maruff P, Thomas E, Cysique L, Brew B, Collie A, Snyder P, Pietrzak RH (2009). Validity of the CogState brief battery: Relationship to standardized tests and sensitivity to cognitive impairment in mild traumatic brain injury, schizophrenia, and AIDS dementia complex. Archives of Clinical Neuropsychology, 24, 165-178.

IDN lmn — Speed of performance; mean of the log 10 transformed reaction times for correct responses.

Range 2.001-6 — Low score = good

*ONE CARD LEARNING TEST (HAVE YOU SEEN THIS CARD BEFORE?)**

Date Added: 11/1/2017 for US sites
4/1/2018 for non-US Sites

Date Discontinued: 06OCT2020

Reference: Maruff P, Thomas E, Cysique L, Brew B, Collie A, Snyder P, Pietrzak RH (2009). Validity of the CogState brief battery: Relationship to standardized tests and sensitivity to cognitive impairment in mild traumatic brain injury, schizophrenia, and AIDS dementia complex. Archives of Clinical Neuropsychology, 24, 165-178.

OCL acc — Accuracy of performance; arcsine transformation of the square root of the proportion of correct responses.

Range 0-1.57 — High score = good

*ONE-BACK MEMORY (IS THE CARD THE SAME AS THE PREVIOUS CARD?)**

Date Added: 11/1/2017 for US sites
4/1/2018 for non-US Sites

Date Discontinued: 06OCT2020

Reference: Maruff P, Thomas E, Cysique L, Brew B, Collie A, Snyder P, Pietrzak RH (2009). Validity of the CogState brief battery: Relationship to standardized tests and sensitivity to cognitive impairment in mild traumatic brain injury, schizophrenia, and AIDS dementia complex. Archives of Clinical Neuropsychology, 24, 165-178.

ONB lmn — Speed of performance; mean of the log 10 transformed reaction times for correct responses

Range 2.001-6 — Low score = good

*** See Cogstate File Format Specification and Data Description PDF for more detail.**

BOSTON NAMING TEST - 30 (ODD NUMBERED ITEMS)

(not used at Japan sites)

Date Added: 1/09

Date Discontinued: 06OCT2020

References: Kaplan, E., Goodglass, H., & Weintraub, S. (1983). *Boston Naming Test scoring booklet*. Philadelphia: Lea & Febiger.

Goodglass, H., & Kaplan, E. (1983). *The assessment of aphasia and related disorders* (2nd ed.). Philadelphia: Lea & Febiger.

Mack, W. J., Freed, D. M., Williams, B. W., & Henderson, V. W. (1992). Boston Naming Test: Shortened versions for use in Alzheimer's disease. *Journal of Gerontology: Psychological Sciences,* 45, P154-P158.

Fisher, N. J., Tierney, M. C., Snow, W. G., & Szalai, J. P. (1999). Odd/even short forms of the Boston Naming Test: Preliminary geriatric norms. *Clinical Neuropsychologist,* 13, 359-364.

Begin at item 1 and present all 30 (odd numbered) items in order. Allow 20 seconds for each response. If participants give a response that indicates a misperception of the picture, administer the printed stimulus cue. Allow 20 seconds for response. If response following stimulus cue is incorrect, the printed phonemic cue is given. The total score is the number of items named correctly to include those named following given stimulus cues.

BOSTON Total correct

Range: 0 - 30 High score = good

Korean Version BOSTON NAMING TEST – 30 ITEMS * used only at Korean site(s)

Date Added: 1/18
Date Discontinued: 06OCT2020

References: Kim, H. H. and Na, D. L. (1999). Normative data on the Korean version of the Boston Naming Test. *Journal of Clinical and Experimental Neuropsychology,* 21;127-133.

Begin at item 1 and present all 30 items in order. Allow 20 seconds for each response. If participants give a response that indicates a misperception of the picture, administer the printed stimulus cue. Allow 20 seconds for response. If response following stimulus cue is incorrect, the printed phonemic cue is given. The total score is the number of items named correctly to include those named following given stimulus cues. Stimulus items used for Korean version are culturally appropriate to Korea and entirely different from the stimulus items selected from the Kaplan and Goodglass Boston Naming Test.

BOSTON Total correct

Range: 0 – 30 High score = good

MULTILINGUAL NAMING TEST
(used only at DIAN Japan sites)

Date Added: 4/2016 for Japan sites only

Date Discontinued: 06OCT2020

References: Ivanova I, Salmon DP, Gollan TH. (2013) The Multilingual Naming Test In Alzheimer's Disease: Clues to the Origin of Naming Impairments. *J Int Neuropsychol Soc.* 19:272-283

Gollan TH, Weissburger G, Runnqvist E, Montoya RI, Cera CM. (2011) Self-ratings of spoken language dominance: A Multilingual Naming Test (MINT) and preliminary norms for young and aging Spanish-English bilinguals. *Bilingualism: Language and Cognition.* 13;215-8.

Begin at item 1 and present all 32 items in order. Allow 20 seconds for each response. If participants give a response that indicates a misperception of the picture, administer the printed semantic cue. Depending on participant's response, a prompt may be given for a "more specific", "more general" or "another" name. If the participant gives the correct name but indicates that is not the object "It's not a peacock", a prompt may be given "Do you know the name?" If the participant does not give the correct response, the printed phonemic cue is given. The total score is the number of items named correctly without prompts or following semantic cues.

MINTOTS Total correct.

Range: 0 – 32 High Score = good

VERSION HISTORY

Version	Date	Summary
1.5	01JAN2019	Latest Version
1.6	22DEC2022	• Updated to note discontinuation of Cogstate Tests, IPIP and ELSMEM.<br>• Changed title from "DIAN PSYCHOMETRIC CODEBOOK" to "DIAN COGCORE DATA CODEBOOK."<br>• General formatting updates.<br>• Added table of contents.
1.7		• Added eCOA Tests.<br>• General formatting updates.

Cogstate File Format Specification

**Protocol**	DIANObs
**Version**	2.0

Table of Contents

1 Preface 3
2 Cogstate Tests 3
3 Outcome Variables 5
3.1 All outcome variables 5
4 Cogstate Standard Data File Format 6
5 Statistical Analyses 7
5.1 Missing Data 7
5.2 Test Completion 7
5.3 Test Data Integrity 7
5.4 Completion and Integrity Flag Columns – Data Extract Design 8

Tables & Figures

Table 1: Test Information and Cognitive Domain Assessed 3
Table 2: Outcome Variables Reported for Each Test 5
Table 3: Cogstate Battery Data File Format 6
Table 4: Test Completion Criteria 7

1 Preface

This document describes the Cogstate data set. Data managers, programmers, and statisticians can refer to this document to understand Cogstate data.

- Section 2 contains information on the Cogstate tests in your study, including the primary outcome measure for each test.
- Section 3 describes the variables reported in the data set.
- Section 4 describes the computerized data file format.
- Section 5 contains information about statistical analysis.

2 Cogstate Tests

Table 1 provides a description of Cogstate tests. Note that a test code (TCode) is used to identify each Cogstate test in the data set.

While there are many outcome variables populated in the data file, each test has a primary outcome variable that Cogstate recommend is used for analysis. The primary outcome variable, along with the corresponding variable code in the data file, has been provided in the below table.

For a detailed description of our tests, please refer to our Task Description document.

Table 1: Test Information and Cognitive Domain Assessed

Cogstate Test (TCode)	Cognitive Domain and Instructions	Primary Outcome Measure (Variable Code)	Interpretation
Detection Test (DET)	Psychomotor Function<br><br>Has the card turned over?	Speed of performance; mean of the log_{10} transformed reaction times for correct responses (lmn)	Lower score = better performance
Chase Test (GMCT)	Visual Motor Control<br><br>Chase the target.	Number of correct moves per second while chasing the target (mps)	Higher score = better performance
Groton Maze Learning Test (GML)	Executive Function<br><br>Find the hidden pathway.	Total number of errors made when learning the same hidden pathway across the consecutive learning trials (ter)	Lower score = better performance
Groton Maze Learning Test – Delayed Recall (GMR)	Memory<br><br>Remember the hidden pathway learned previously in the battery.	Total number of errors made when remembering the maze pathway after a delay (ter)	Lower score = better performance
Groton Maze Learning Test – Reverse Recall (GMRV)	Memory<br><br>Remember the hidden pathway learned previously in the battery in the reverse direction.	Total number of errors made when remembering the maze pathway in the reverse direction after a delay (ter)	Lower score = better performance

Cogstate Test (TCode)	Cognitive Domain and Instructions	Primary Outcome Measure (Variable Code)	Interpretation
Identification Test (IDN)	Attention<br><br>Is the card red?	Speed of performance; mean of the $\log_{10}$ transformed reaction times for correct responses (lmn)	Lower score = better performance
International Shopping List Test (ISL)	Verbal Learning<br><br>Tell me the items on the shopping list.	Number of correct responses made when remembering the word list on three consecutive trials (cor)	Higher score = better performance
International Shopping List Test – Delayed Recall (ISRL)	Memory<br><br>Tell me the items on the shopping list that you learned earlier.	Number of correct responses made when remembering the word list after a delay (cor)	Higher score = better performance
One Card Learning Test (OCL)	Visual Learning<br><br>Have you seen this card before?	Accuracy of performance; arcsine square root proportion correct (acc)	Higher score = better performance
One Back Test (ONB)*	Working Memory<br><br>Is this card the same as the previous card?	Speed of performance; mean of the $\log_{10}$ transformed reaction times for correct responses (lmn)	Lower score = better performance
One Back Test (ONB)*	Working Memory<br><br>Is this card the same as the previous card?	Accuracy of performance; arcsine square root proportion correct (acc)<br>*Note: if measuring change over time, speed of performance should be used, not accuracy.*	Higher score = better performance

** Note: for the One Back test, both speed of performance and accuracy can be used for analysis, depending on the study population and research questions of interest; if you are measuring change over time, speed of performance should be used, not accuracy.*

3 Outcome Variables

3.1 All outcome variables

The different Cogstate tests produce data for different outcome variables.

Table 2 lists the outcome variables (shown as the abbreviations used in the data extract) for each of the Cogstate tests.

Table 2: Outcome Variables Reported for Each Test

TCode	GMLidx	mps	dur	ter	ler	rer	per	lmn	lsd	acc	cor	err	presnt	cmv	rth	sti	res	Completion	Integrity
DET	-	-	-	-	-	-	-	✓	✓	✓	✓	✓	✓	-	-	✓	-	✓	✓
GMCT	-	✓	✓	✓	-	✓	✓	-	-	-	-	-	-	✓	✓	-	-	-	-
GML	✓	✓	✓	✓	✓	✓	✓	-	-	-	-	-	-	✓	✓	-	-	✓	✓
GML1	✓	✓	✓	✓	✓	✓	✓	-	-	-	-	-	-	✓	✓	-	-	-	-
GML2	✓	✓	✓	✓	✓	✓	✓	-	-	-	-	-	-	✓	✓	-	-	-	-
GML3	✓	✓	✓	✓	✓	✓	✓	-	-	-	-	-	-	✓	✓	-	-	-	-
GML4	✓	✓	✓	✓	✓	✓	✓	-	-	-	-	-	-	✓	✓	-	-	-	-
GML5	✓	✓	✓	✓	✓	✓	✓	-	-	-	-	-	-	✓	✓	-	-	-	-
GMR	✓	✓	✓	✓	✓	✓	✓	-	-	-	-	-	-	✓	✓	-	-	✓	-
GMRV	✓	✓	✓	✓	✓	✓	✓	-	-	-	-	-	-	✓	✓	-	-	✓	-
IDN	-	-	-	-	-	-	-	✓	✓	✓	✓	✓	✓	-	-	✓	-	✓	✓
ISL	-	-	✓	-	-	-	-	-	-	✓	✓	✓	-	-	-	✓	✓	✓	-
ISL1	-	-	✓	-	-	-	-	-	-	✓	✓	✓	-	-	-	✓	✓	-	-
ISL2	-	-	✓	-	-	-	-	-	-	✓	✓	✓	-	-	-	✓	✓	-	-
ISL3	-	-	✓	-	-	-	-	-	-	✓	✓	✓	-	-	-	✓	✓	-	-
ISRL	-	-	✓	-	-	-	-	-	-	✓	✓	✓	-	-	-	✓	✓	✓	✓
OCL	-	-	-	-	-	-	-	✓	✓	✓	✓	✓	✓	-	-	✓	-	✓	✓
ONB	-	-	-	-	-	-	-	✓	✓	✓	✓	✓	✓	-	-	✓	-	✓	✓

4 Cogstate Standard Data File Format

The Cogstate battery data output is a delimited ASCII file. Each row in the data set represents one test in the Cogstate test battery for a single subject at a single test session. Tests are identified in the data set by a test code (TCode).

A description of each column heading in the data set is presented in Table 3.

Please note that test session details and demographic data are common to all rows (i.e., reported for all tests at all time-points). The format of certain fields in the standard data file below may be customized. Details are found in the File Format Agreement document provided as an addendum to this specification.

Table 3: Cogstate Battery Data File Format

Variable Code	Data Type	Description	Range of Values
SiteID	Character/numeric	Site ID	n/a
cg_id	Character/numeric	Subject ID	n/a
BYear	Numeric	Year of birth	n/a
Hand	Character	Dominant hand	Right/Left
cg_sex	Character	Sex	Male/Female
TDate	Date	Test date	n/a
TTime	Time	Test time	n/a
Sessn	Character/numeric	Scheduled visit	n/a
TCode	Character/numeric	Test code (see Table 1)	n/a
GMLidx	Numeric	GML test index	0 to 20
mps	Numeric	Moves per second	0.0001 to 5
dur	Numeric	Duration of test (ms)	0 to 99999
ter	Numeric	Total errors (count)	0 to 999
ler	Numeric	Legal errors (count)	0 to 999
rer	Numeric	Rule break errors (count)	0 to 999
per	Numeric	Perseverative errors (count)	0 to 999
lmn	Numeric	Speed of performance (log10 msec)	2.001 to 6
lsd	Numeric	Standard deviation of speed of performance	0 to 3
acc	Numeric	Accuracy (arcsin sqrt proportion)	0 to 1.5708
cor	Numeric	Correct responses (count)	0 to 999
err	Numeric	Errors (count)	0 to 999
presnt	Numeric	Presentation count (Correct + Errors)	0 to 99
cmv	Numeric	Correct moves	0 to 140
rth	Numeric	Return to head moves	0 to 999
sti	Numeric	Stimuli (count)	0 to 999
res	Numeric	Responses (count)	0 to 999
protocolId	Character	Protocol ID	n/a
Completion	Numeric	Test completion flag (0 = pass; 1 = fail); see section 5.2	0 or 1
Integrity	Numeric	Test integrity flag (0 = pass; 1 = fail); see section 5.3	0 or 1

5 Statistical Analyses

5.1 Missing Data

There are two instances when test information will not be provided in the data set: 1) when the subject misses a study visit/testing session entirely, and, 2) when the subject is present for a study visit/testing session but does not perform one or more of the tests.

5.2 Test Completion

Test completion refers to criteria that determine whether a sufficient number of responses were recorded during the administration of a test to allow the computation of reliable performance measures.
Test completion criteria are assigned before study start up and are the same for all constructs or compounds under investigation.

The data set includes a “Completion” column that indicates whether each test administration met the completion criteria. In this column, a value of “0” or “1” is used to indicate whether a score derived from a test administration was deemed sufficiently complete.

0 = test administration met completion criteria.
1 = test administration did not meet completion criteria.

The definition of completion for each test is detailed in Table 4. Cogstate recommends removing test administrations that fail completion criteria from any statistical analyses.

Table 4: Test Completion Criteria

Test Code	Default Number of Trials	Completion Criteria
DET	35	27
GML	140	140
GMR	28	28
GMRV	28	28
IDN	30	23
ISL	36	36
ISRL	12	12
OCL	80	60
ONB	31	24

5.3 Test Data Integrity

Test data integrity is a measure of whether or not a subject performed in accord with the test requirements. When a particular test administration fails to meet criteria for data integrity, this suggests with high probability that the observed score may not reflect the study population or the effect of the compound under investigation.

The criteria for test data integrity are derived statistically and assigned *a priori* to study start-up by the Cogstate Science Team such that when trained and supervised appropriately, subjects from the relevant study population will typically achieve the defined criteria for each test. Given that test data integrity criteria are based on probabilistic estimates, it is possible that individual subjects who understand the test requirements, are well motivated, and are well supervised have a performance that fails test data integrity. Infrequent and random failures of test data integrity do not threaten the validity of cognitive data in a clinical trial.

The data extract can include a column that indicates whether the test administration passed data integrity. In this column, entitled “Integrity”, a value of “0” or “1” is used to indicate whether or not a subject performed in accord with the test requirements.

0 = test administration met integrity criteria.
1 = test administration did not meet integrity criteria.

Note: Cogstate suggests that a sensitivity analysis be run with test data integrity failures excluded if the integrity failure rate is over 10%.

5.4 Completion and Integrity Flag Columns – Data Extract Design

Test data integrity criteria are applied only when a sufficient number of responses were recorded to allow computation of reliable performance measures (i.e., for data that satisfy test completion criteria). Thus, there are three possible outcomes associated with test completion and integrity failure flags in a data extract:

- A particular test administration can have a “0” in both of the “Completion” and “Integrity” columns (i.e., indicating that both criteria were passed).
- A particular test administration can have a “0” in the “Completion” column and have a “1” in the “Integrity” column (i.e., an integrity failure).
- A particular test administration can have “1” in the “Completion” column and have a blank in the “Integrity” column (i.e., a completion failure).

D I A N
Dominantly Inherited
Alzheimer Network

DIAN DF17

IMAGING CORE
Methods and Definitions
v1.0

November 2023

Table of Contents

Introduction and Contact Information 4
Data Archiving and Quality Control 5
Neuroradiological Interpretations 5
Data Distribution and Data Freezes 6
Recent and Upcoming Data Freeze Information: 7
DIAN DF7 May 2014 7
DIAN DF8 July 2014 (PET Unified Processing Pipeline) 7
DIAN DF9 April 2015 (PET Unified Processing Pipeline) 7
DIAN DF10 May 2016 (FreeSurfer v5.3) 7
DIAN DF11 November 2016 (White Matter Hyperintensity detection) 8
DIAN DF12 November 2017 8
DIAN DF13 November 2018 8
DIAN DF14 December 2019 9
DIAN DF15 November 2020 9
DIAN DF16 September 2022 9
DIAN DF17 September 2023 10
MRI Imaging and Processing 10
FreeSurfer: Quality Control Measures 10
MR Scanner Comparison 11
FreeSurfer: Correcting Subcortical and Cortical Volumes for Head Size 12
Instructions for Normalization of MRI Freesurfer-derived Cortical Volumes 13
FreeSurfer: Additional Regional Calculations 13
ADAD Cortical Signature for Cortical Thickness 14
Individual Longitudinal Reports 15
FreeSurfer: Default Variables & Biostatistics Correlates 15
White Matter Lesion Processing and Analysis 19
PET Processing 21
Using Manual ROI Data 22
Centiloid Conversion for Amyloid PET 23
Cutoff Values for Amyloid Positivity 24
Tau PET Imaging Analysis 25
PET Variable Nomenclature 25
PET Processing Variables & Biostatistics Correlates 26
References 31

Introduction and Contact Information

The Dominantly Inherited Alzheimer's Network (DIAN) Imaging Core works alongside the Administration Core and Clinical Core and has a primary role of acquisition and processing of imaging data. This Data Dictionary serves as a reference manual for researchers requesting or using imaging data generated by the Imaging Core.

Data are available for access in several permutations:

- Derived variables in spreadsheets (SAS or Excel)
- Source (DICOM) and derived data via web download from the
  - Open Access Series of Imaging Studies (OASIS) www.oasis-brains.org
  - sFTP with approved data request
- ClinPortal synchronization project (in progress, contact BioStats core for further details)

Further resources, including updated copies of this Data Dictionary, are available online at our web site: https://sites.google.com/site/benzingerlabwustl/

Researchers should cite the following DIAN imaging methods paper when using source images or processed imaging data:

McKay, N. S., Gordon, B. A., Hornbeck, R. C., Jack, C. R., Koeppe, R., Flores, S., ... & Dominantly Inherited Alzheimer Network. (2022). Neuroimaging within the Dominantly Inherited Alzheimer's Network (DIAN): PET and MRI. *bioRxiv*.

Imaging Core Director:
Tammie Benzinger, MD, PhD
benzingert@wustl.edu

Regulatory Manager:
Deborah Koudelis
delanod@wustl.edu
314-747-3876

Imaging Core Project Manager:
Russ Hornbeck
russ@wustl.edu
314-362-6905

Data Archiving and Quality Control

All imaging data is stored in the DIAN Central Archive, the DCA (Marcus, Olsen et al. 2007). DICOM-based transceivers at our scanners automatically send images to the DCA within minutes of collection. The DCA data is housed on a ZFS RAID-based system with 100% mirroring for disaster recovery and full back up. To facilitate data access, a web-based archive and visualization system has been developed and is now maintained by Dan Marcus, PhD (Marcus, Olsen et al. 2007). Before the DCA makes the image data available for processing or extraction, the data undergoes a series of quality control steps. The quality of the MRI data is assured by a rigorous quality assurance (QA) and preventative maintenance (PM) program that has been in operation for our research scanners for more than a decade. QA/PM tests are performed by a combination of a dedicated Siemens field service engineer and the MRI and PET chief technologists. At the scanner, the sequence parameters are verified by the operating technologist and images are reviewed for motion and repeated if needed which are documented both on a study procedure form and captured electronically at the time of archiving. Multiple additional QC steps are then employed during each phase of the processing. Further details of this integrated workflow and QC for volumetric MRI and PET imaging are provided below. Post-processed data generated via DCA processing (more details below) are also synchronized with the portion of the database residing in Biostatistics.

Neuroradiological Interpretations

MR sequences in the standard protocol allow us to quantify pathological changes due to small vessel disease, including assessment of white matter hyperintensity, microhemorrhage, and prior stroke. All MR studies acquired are interpreted by board-certified neuroradiologists at Mayo Clinic. Upon receipt in the DCA, an automated email notification is sent to the readers and the study is placed in a reading queue. Readers have the option of viewing the images directly using a built-in image viewer or of downloading to a local workstation. Readings are entered directly into the DCA by the radiologist. Abnormal findings are flagged and referred to the Imaging Core Leader (Dr. Benzinger) and study PI (Dr. Bateman) for follow up. In cooperation with Barnes-Jewish Hospital, we have developed a HIPAA compliant workflow that allows for scans with abnormal findings to be added to the participant's electronic medical record (if desired) and/or for a CD with an image viewer and interpretation to be provided to the participant and his personal physician (if outside our network).

Data Distribution and Data Freezes

Data are shared with investigators by several mechanisms.

Requests for ADRC (non-DIAN) data (including raw imaging data) should be logged at the Knight ADRC website:
http://alzheimer.wustl.edu/Research/ResourceRequest.htm

Requests for DIAN data should be logged online at:
https://dian.wustl.edu/our-research/observational-study/dian-observational-study-investigator-resources/data-request-terms-and-instructions/

DICOM is available to outside investigators via the Open Access Series of Imaging Studies (OASIS) project. www.oasis-brains.org

DICOM is available to engaged study investigators at Washington University and to investigators with an approved resource request via sFTP

Users of the DICOM data notice PET reconstructions utilizing a 128 or 256 size matrix (128mtx or 256mtx). This is a scan reconstruction specific to WU and should not be used when analyzing DIAN data.

Processed volumetric MRI and PET data is available from the Biostatistics Core for all investigators with an approved resource request.

Processed volumetric MRI and PET data is deposited on a regular basis. The contents of this manual describe the data deposited cumulatively for the DIAN project through 2023. A detailed description of currently available data and imaging methods are provided in McKay et al. (2022). For PET and MR imaging protocols please refer to the technical procedures manuals for each modality. To receive the DIAN imaging manuals, further clarification of the processing methods used, or if specialized processing is required, please contact a Core Leader or Co-Leader.

Data Freeze Archive Information:

DIAN DF7 May 2014

- Contains 510 MRI, 402 FS-PiB, 447 FS-FDG, 33 manual-PiB, 46 manual-FDG

DIAN DF8 July 2014 (PET Unified Processing Pipeline)

- DIAN DF8 PET data has been (re)processed using the Imaging Core Laboratory's PET Unified Processing pipeline (PUP). This pipeline allows for better target registration and reduction of motion artifact which recovers imaging sessions whose ROI analyses could only be processed manually. Reprocessing with PUP yields superior longitudinal imaging data. PUP will also allow for di novo atlas registration and refined (template and manually drawn) ROI using the same engine
- Contains 574 MRI, 487 FS-PiB, 515 FS-FDG, 28 manual-PiB, 41 manual-FDG
- Longitudinal Summary
  - ASL: 169 subjects with 1 scan, 18 subjects with more than 1 scan
  - MRI: 193 with 1 scan, 161 with more than 1 scan.
  - FDG: 194 with 1 scan, 148 with more than 1 scan
  - PIB: 192 with 1 scan, 139 with more than 1 scan

DIAN DF9 April 2015 (PET Unified Processing Pipeline)

- Contains 632 MRI, 492 FS-PiB, 522 FS-FDG, 78 manual-PiB, 35 manual-FDG
- FS statistics do not include intracranial volume (ICV) values due to known issues with FreeSurfer version 5.1 calculations of ICV
- Longitudinal Summary
  - MRI: 201 with 1 scan, 172 with more than 1 scan
  - FDG: 191 with 1 scan, 142 with more than one scan
  - PIB: 187 with 1 scan, 131 with more than 1 scan

DIAN DF10 May 2016 (FreeSurfer v5.3)

- Imaging IDS will be anonymized
- FreeSurfer Cubic Spline Interpolation set to off (0) for fewer edits, reprocessing
- FreeSurfer 5.3-HCP-patch used for MRI statistics
- Corrected and restored Intracranial Volume values (ICV)
- PUP processing with scanner specific spatial filtering
- Longitudinal Imaging Summary (Scans Available)

	v00	v01	v02	v03	v04	v05	v06
**MRI**	407	111	75	117	20	8	2
**PiB**	375	95	69	117	19	6	2
**FDG**	387	103	72	123	21	6	2

DIAN DF11 November 2016 (White Matter Hyperintensity detection)

- An SPM based Lesion Segmentation Tool (LST) pipeline has been added to detect and segment T2 hyperintense lesions in FLAIR images. Originally developed for the segmentation of MS lesions, it has proven useful for the segmentation of brain lesions in Alzheimer's disease
- FreeSurfer 5.3-HCP-patch used for MRI statistics
- Longitudinal Imaging Summary (Scans Available)

	v00	v01	v02	v03	v04	v05	v06
**MRI**	426	112	83	124	24	20	11
**PiB**	394	97	78	124	22	21	8
**FDG**	406	104	77	130	25	21	8

DIAN DF12 November 2017

- SPM based Lesion Segmentation Tool (LST) pipeline used to detect and segment T2 hyperintense lesions in FLAIR images
- FreeSurfer 5.3-HCP-patch used for MRI statistics
- Longitudinal Imaging Summary (Scans Available)

	v00	v01	v02	v03	v04	v05	v06
**MRI**	480	112	104	136	31	47	20
**PiB**	438	97	88	132	29	37	14
**FDG**	451	104	87	139	32	40	15

DIAN DF13 November 2018

- SPM based Lesion Segmentation Tool (LST) pipeline used to detect and segment T2 hyperintense lesions in FLAIR images
- FreeSurfer 5.3-HCP-patch used for MRI statistics
- Longitudinal Imaging Summary (Scans Available)

	v00	v01	v02	v03	v04	v05	v06	v07	v08
**MRI**	499	118	119	136	44	58	25	23	5
**PiB**	467	104	104	132	36	58	22	19	6
**FDG**	476	111	97	139	39	58	21	21	6

DIAN DF14 December 2019

- SPM based Lesion Segmentation Tool (LST) pipeline used to detect and segment T2 hyperintense lesions in FLAIR images
- FreeSurfer 5.3-HCP-patch used for MRI statistics
- PET Unified Pipeline (PUP) used for PET statistics
- Longitudinal Imaging Summary (Scans Available)

	v00	v01	v02	v03	v04	v05	v06	v07	v08	v09	v10
**MRI**	556	129	169	143	67	64	42	50	13	9	2
**PiB**	521	112	137	137	47	61	32	43	14	9	2
**FDG**	522	120	128	143	47	61	27	38	12	7	2

DIAN DF15 November 2020

- SPM based Lesion Segmentation Tool (LST) pipeline used to detect and segment T2 hyperintense lesions in FLAIR images
- FreeSurfer 5.3-HCP-patch used for MRI statistics
- PET Unified Pipeline (PUP) used for PET statistics
- Longitudinal Imaging Summary (Scans Available)

	v00	v01	v02	v03	v04	v05	v06	v07	v08	v09	v10
**MRI**	563	132	175	145	76	66	50	51	13	16	6
**PiB**	530	113	139	137	51	63	36	44	14	15	5
**FDG**	531	121	129	144	48	63	30	39	12	14	6

DIAN DF16 September 2022

- SPM based Lesion Segmentation Tool (LST) pipeline used to detect and segment T2 hyperintense lesions in FLAIR images
- FreeSurfer 5.3-HCP-patch used for MRI statistics
- PET Unified Pipeline (PUP) used for PET statistics
- Addition of tau tracers 18F-Flortaucipir (AV-1451) and 18F-MK-6240
- Longitudinal Imaging Summary (Scans Available)

	v00	v01	v02	v03	v04	v05	v06	v07	v08	v09	v10	v11	v12
**MRI**	576	125	185	149	94	67	66	512	24	29	15	2	5
**PiB**	524	104	140	132	66	64	47	45	13	25	11	2	3
**FDG**	528	119	130	140	56	58	32	36	8	16	10	1	2
**MK-6240**	3	3	2	3	1	2	1	3	1	0	0	0	0
**AV-1451**	23	12	6	11	9	6	9	5	7	3	1	1	0

DIAN DF17 September 2023

- SPM based Lesion Segmentation Tool (LST) pipeline used to detect and segment T2 hyperintense lesions in FLAIR images
- FreeSurfer 5.3-HCP-patch used for MRI statistics
- PET Unified Pipeline (PUP) used for PET statistics
- Addition of tau tracers 18F-Flortaucipir (AV-1451) and 18F-MK-6240
- Longitudinal Imaging Summary (Scans Available)

	v00	v01	v02	v03	v04	v05	v06	v07	v08	v09	v10	v11	v12	v13	v14
**MRI**	652	140	200	159	113	77	79	58	32	33	19	7	9	1	0
**PIB**	598	121	152	151	77	70	60	52	20	29	14	7	7	0	0
**FDG**	559	127	138	154	63	65	38	41	12	18	10	1	3	0	0
**MK-6240**	34	1	5	4	7	3	1	8	6	6	3	2	1	0	0
**T80**	27	1	15	9	13	9	14	9	6	10	7	4	3	1	0
**PET**	1218	250	310	318	160	147	113	110	44	63	34	14	14	1	0

MRI Imaging and Processing

Note: The following paragraphs outline processing details useful for citation in manuscripts using DIAN Imaging Core data.

Structural MRI acquisition was performed using the Alzheimer Disease Neuroimaging Initiative (ADNI) protocol (Jack et al., 2008, Jack et al., 2010). Participating sites were required to pass initial and regular follow-up quality control assessments to insure acquisition conformity. Each participant received an accelerated 3D sagittal T1-weighted MPRAGE on a 3T scanner. A high quality, whole-brain image with 1.1x1.1x1.2 mm voxels was acquired in approximately 5-6 minutes. Before analysis, images were screened for artifacts and protocol compliance by the ADNI imaging core.

Prior to December 31, 2019, MRI processing was performed on Dell PowerEdge servers using Intel Xeon processors with Cent OS 5.5. Currently, all new processing/reprocessing will take place on AWS using virtual processing containers to maintain OS stability for the duration of the study

[*FOR MANUSCRIPTS, SELECT ONLY THE SCANNER(S) FROM WHICH YOUR DATA WERE DERIVED*]

FreeSurfer (http://surfer.nmr.mgh.harvard.edu/) analysis involves cortical reconstruction and volumetric segmentation of T1 weighted images.

The technical details of these procedures are described in prior publications (Fischel et al., 2012 see https://surfer.nmr.mgh.harvard.edu/fswiki/FreeSurferMethodsCitation). The processing pipeline includes motion correction and segmentation of the subcortical white matter and deep gray matter volumetric structures on a T1 weighted image (Fischl et al., 2002), intensity normalization, registration to a spherical atlas which utilizes individual cortical folding patterns to match cortical geometry across participants (Fischl et al., 1999b), and parcellation of the cerebral cortex into units based on gyral and sulcal structure based upon the Desikan atlas (Desikan et al., 2006).

All 3.0T MRI imaging data was reprocessed using FreeSurfer 5.3-HCP-patch.

FreeSurfer: Quality Control Measures

Individuals responsible for processing imaging data are trained in the FreeSurfer quality control (QC) measures developed by the Imaging Core. During the QC process, the rater will locate all FreeSurfer errors in the cortical parcellation and subcortical segmentation that meet a certain size based on the QC criteria. An error that meets the QC criteria will require manual intervention (editing the FreeSurfer) and/or will fail quality control. There are two main types of QC FreeSurfer errors: inclusion and exclusion. Inclusion errors are identified as non-brain regions (dura, skull, etc.) that are being assessed as part of the brain and exclusion errors are identified as brain regions that are excluded from the cortical/subcortical classification.
FreeSurfer QC criteria is as follows:

- Dural inclusion, gray matter exclusion, sulcus inclusion, cerebellum inclusion will require edits if equal to or larger than 120 voxels.
- Cerebellum, subcortical and hippocampus segmentation exclusion will fail quality control if greater than 120 voxels. Before failing, other errors will be fixed if possible, which may fix segmentation exclusions.
- White matter exclusion will require edits if equal to or larger than 60 voxels.
- Lateral ventricle segmented incorrectly will require edits if equal to or larger than 300 voxels.

- Generally, three attempts will be made to fix FreeSurfer errors if the error persists after performing edits. After the third attempt, the FreeSurfer will either pass or fail QC depending on the size of the error after multiple rounds of edits

MR Scanner Comparison

Siemens TIM Trio 3T and Siemens Biograph 3T mMR
In order to transition the cohort from the Siemens TIM Trio 3T MRI to the Siemens Biograph 3T mMR, a direct correlation was performed in a subset of our participants. For the scanner validation, a total of 69 participants with a mean age of 65.9 years (CDR 0-0.5) received both the Trio and mMR MRI within two weeks. Of the 69 participants, 67 participants were CN (CDR 0) and 2 participants had a diagnosis of mild symptomatic AD (CDR 0.5). FreeSurfer v5.1 was used to segment the brain into various regions of interest (ROI) for quantitative analysis.

For the left hippocampal volume as measured by Trio and the PET MR, the estimated concordance correlation coefficient (CCC) on the raw data is 0.83 with a 95% CI from 0.73 to 0.89, and after the standardization, the estimated CCC is 0.83 with a 95% CI from 0.74 to 0.90. For the right hippocampus volume as measured by Trio and the PET MR, the estimated CCC on the raw data is 0.79 with a 95% CI from 0.67 to 0.87, and after the standardization, the estimated CCC is still 0.79 with a 95% CI from 0.67 to 0.87.

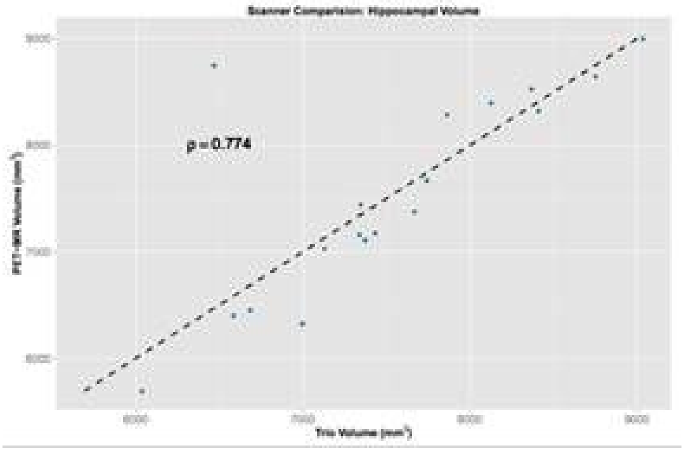

Figure 1: Hippocampal volume comparison of FreeSurfer derived hippocampal volumes from the Siemens TIM Trio 3T MRI and the Siemens Biograph mMR 3T PET-MR.

Because of the two potential outliers on the hippocampal volumes data (Fig. 1), rank-based CCC was performed on these measures. The rank-based CCC for left hippocampal volume is 0.92 with a 95% CI from 0.86 to 0.95, and the rank-based CCC for right hippocampal volume is 0.91 with a 95% CI from 0.86 to 0.95; both indicating excellent rank-based reproducibility of measuring hippocampal volumes. These findings are within the reported test-retest reliability range for repeat MRI visits on the same scanner (Han et al., 2006).

FreeSurfer: Correcting Subcortical and Cortical Volumes for Head Size

It is recommended that the MRI regional volumes be corrected for head size (intracranial volume, ICV) in order to have correct comparisons. This does not apply to cortical thickness measures, as cortical thickness does not significantly vary with head size. The normalization process applies to each individual ROI and is sample specific. Please note if participants are removed from the data set the normalizations on the subcortical volumes will need to be re-run.

Note: Volume normalization must be repeated <u>every time</u> a participant is added or removed from the sample.

An analysis of the ICV estimate for each participant was performed on a longitudinal cohort (Fig. 2). All participants had MRI scans using a 3T scanner and were processed with FreeSurfer 5.3. Within a participant, ICV can vary from baseline more than 5% with a mean participant standard deviation of 15.75 cm ^{3}.

Relevant publication for the head-size correction:
Randy L. Buckner, Denise Head, Jamie Parker, Anthony F. Fotenos, Daniel Marcus, John C. Morris, and Abraham Z. Snyder A unified approach for morphometric and functional data analysis in young, old, and demented adults using automated atlas-based head size normalization: reliability and validation against manual measurement of total intracranial volume. **Neuroimage, 2004**

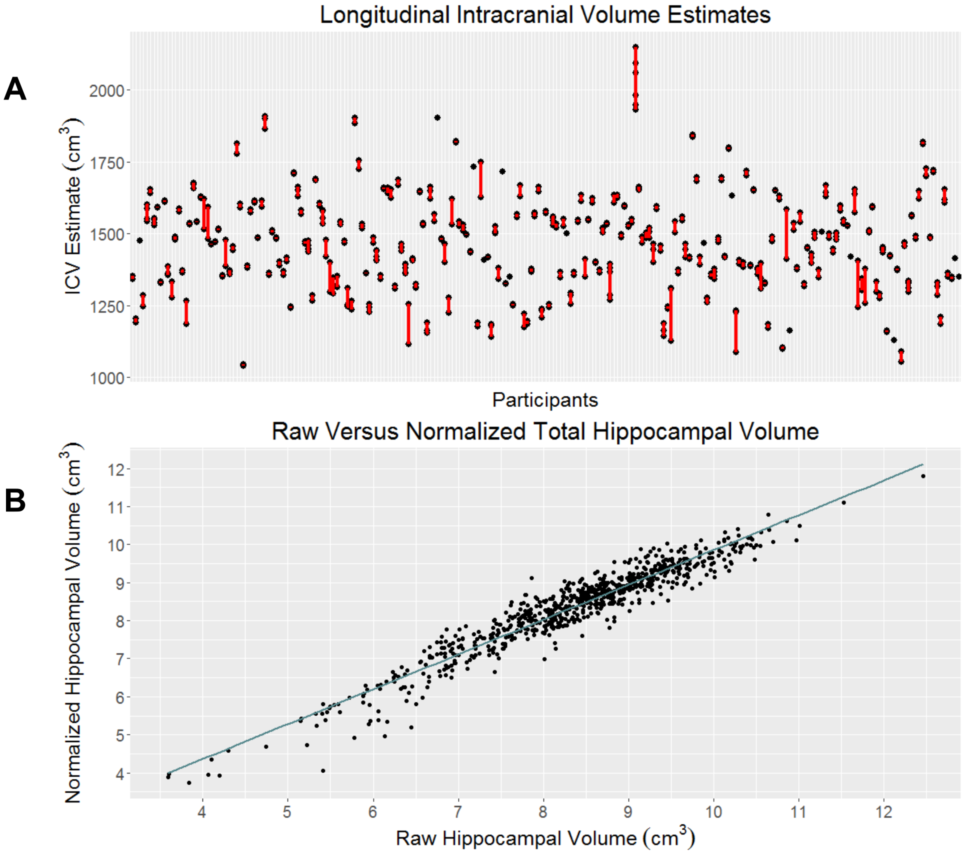

Figure 2. (A) ICV estimate for each participant in a longitudinal study. Each black circle represents an MR session and the red line represents a longitudinal participant. (B) The relationship between the raw hippocampal volume and the hippocampal volume normalized by ICV.

Instructions for Normalization of MRI Freesurfer-derived Cortical Volumes

Normalization Calculation:

1. Compute mean ICV for sample
2. Compute regression with ICV as independent variable and an ROI as dependent variable to obtain B (NOT Beta) weight
3. Compute: Normalized = raw volume – (B-weight * (ss ICV – mean ICV))
   Note: "ss" = single subject's

Table 1 below shows a snapshot from the **SPSS** output for the linear regression. In this example you would use the B value highlighted in red for the correction factor. This will be repeated for each given ROI.

Table 1

Coefficients[a]

Model 1	Unstandardized Coefficients		Standardized Coefficients	t	Sig.
	B	Std. Error	Beta		
(Constant)	2718.207	343.943		7.903	.000
ICV	-1.513E-5	.000	-.008	-.068	.946

a. Dependent Variable: transtemp

This procedure is repeated for each subcortical & cortical ROI volume the investigator is interested in.
Note: We do not normalize the cortical thickness measures, only cortical & subcortical volumes.

FreeSurfer: Additional Regional Calculations

Regional FreeSurfer outputs can be combined to generate multiple global brain measures that researchers may find useful (see https://surfer.nmr.mgh.harvard.edu/fswiki/MorphometryStats):

Whole Brain Volume = *Cortex* + *CorticalWhiteMatter* + *SubCortGray*

Cortex = lhCortex + rhCortex

Total CorticalWhiteMatter = *lhCorticalWhiteMatterVol* + *rhCorticalWhiteMatterVol*

SubCortGray = summation of thalamus, caudate, hippocampus, amygdala, accumbens, ventral DC, substantia nigra (if there). This is a simple voxel count of structures identified as subcortical GM.
(NOTE: SubCortGray excludes brain stem with 5.2.)

Total Ventricular Volume = left and right lateral inferior lateral ventricles + 3rd + 4th + 5th ventricles)

ADAD Cortical Signature for Cortical Thickness

A cortical thickness summary measure has been defined by the weighted average of the left and right hemisphere ADAD cortical signature thickness values (Fig 3, Dincer et. al. 2020)

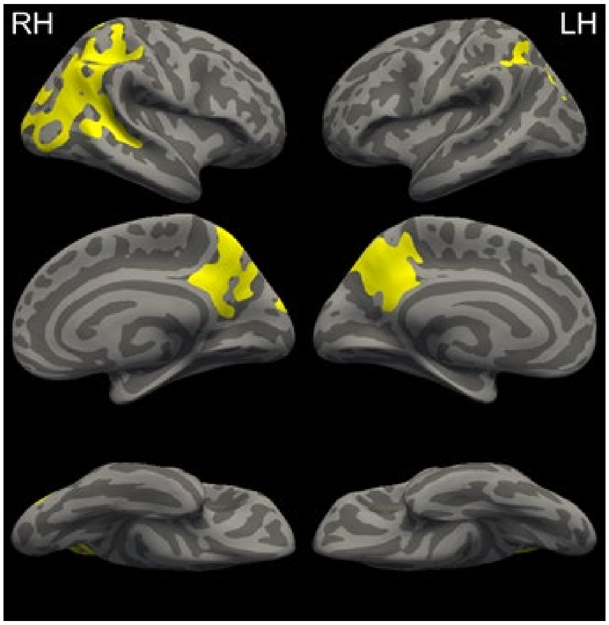

Figure 3: The AD cortical signature depicting cortical thickness differences between the sporadic AD and CN groups including age and sex as covariates. The blue color are the significant vertices.

Individual Longitudinal Reports

Individual longitudinal participant reports (ILP) for volumetric MRI are available. In this example (Fig.4), adjusted hippocampal volume is greater than one standard deviation from our Super-Norm cohort (cognitively normal and biomarker normal) (Table 2). The ILP reports are available for specific participants upon request and may be useful for multicore conferences and case discussions.

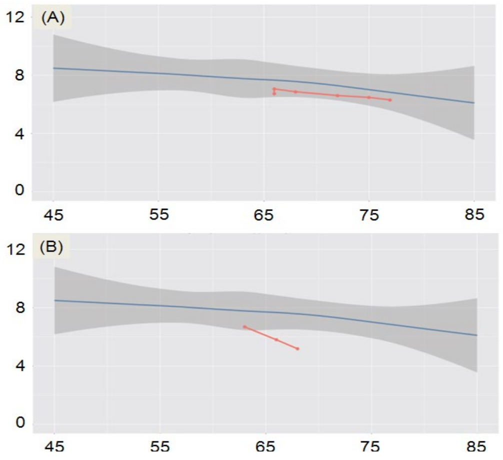

Figure 4: Individual Longitudinal Participant (ILP) reports. x-axis is age (in years). y-axis is normalized hippocampal volume (cm^3). (A) Example of a participant who first had imaging at age 66. Over the course of 6 visits, both clinical status and hippocampal volumes remained normal. (B) Example of a participant who was cognitively normal (CDR 0) at first visit, but who progressed to a diagnosis of AD (CDR 0.5) by the last imaging visit.

Table 2: "Super-Normal" cohort (CN and biomarker negative) and "True AD" cohorts.

Demographic	Super-Normal	AD	*p*-value
n	106	64	-
**Age (SD) years**	71.84 (5.02)	74.81 (5.10)	<0.001
**Education (SD) years**	15.35 (2.61)	14.44 (2.82)	0.038
**% Male**	47.2	48.4	0.874
**APOE4 Status**	E4–=79, E4+ =23, NA = 4	E4–=14, E4+=46, NA=4	<0.001

"NA" denotes cases where ApoE genotyping is not currently available (is in progress).

FreeSurfer: Default Variables & Biostatistics Correlates

MRI Freesurer Default Variable	New Biostat Standardizations
3rd-Ventricle	MR_TOTV_THIRDVENT
4th-Ventricle	MR_TOTV_FOURTHVENT
5th-Ventricle	MR_TOTV_FIFTHVENT
Brain-Stem	MR_TOTV_BRAINSTEM
CC_Anterior	MR_TOTV_CRPCLM_ANT
CC_Central	MR_TOTV_CRPCLM_CNTRL
CC_Mid_Anterior	MR_TOTV_CRPCLM_MID_ANT
CC_Mid_Posterior	MR_TOTV_CRPCLM_MID_POST
CC_Posterior	MR_TOTV_CRPCLM_POST
CortexVol	MR_TOTV_CORTEX
CSF	MR_TOTV_CSF
IntraCranialVol	MR_TOTV_INTRACRANIAL
non-WM-hypointensities	MR_TOTV_NONWMHYPOINTENSITIES
Optic-Chiasm	MR_TOTV_OPTICHIASM
SubCortGrayVol	MR_TOTV_SUBCORTGRAY
TotalGrayVol	MR_TOTV_GRAY
WM-hypointensities	MR_TOTV_WMHYPOINTENSITIES

lh_bankssts_thickness	MR_LT_SSTSBANK
lh_caudalanteriorcingulate_thickness	MR_LT_CAUDANTCNG
lh_caudalmiddlefrontal_thickness	MR_LT_CAUDMIDFRN
lh_cuneus_thickness	MR_LT_CUNEUS
lh_entorhinal_thickness	MR_LT_ENTORHINAL
lh_frontalpole_thickness	MR_LT_FRNPOLE
lh_fusiform_thickness	MR_LT_FUSIFORM
lh_inferiorparietal_thickness	MR_LT_INFRPRTL
lh_inferiortemporal_thickness	MR_LT_INFRTMP
lh_insula_thickness	MR_LT_INSULA
lh_isthmuscingulate_thickness	MR_LT_ISTHMUSCNG
lh_lateraloccipital_thickness	MR_LT_LATOCC
lh_lateralorbitofrontal_thickness	MR_LT_LATORBFRN
lh_lingual_thickness	MR_LT_LINGUAL
lh_medialorbitofrontal_thickness	MR_LT_MEDORBFRN
lh_middletemporal_thickness	MR_LT_MIDTMP
lh_paracentral_thickness	MR_LT_PARACNTRL
lh_parahippocampal_thickness	MR_LT_PARAHPCMPL
lh_parsopercularis_thickness	MR_LT_PARAOPRCLRS
lh_parsorbitalis_thickness	MR_LT_PARSORBLS
lh_parstriangularis_thickness	MR_LT_PARSTRNGLRS
lh_pericalcarine_thickness	MR_LT_PERICLCRN
lh_postcentral_thickness	MR_LT_POSTCNTRL
lh_posteriorcingulate_thickness	MR_LT_POSTCNG
lh_precentral_thickness	MR_LT_PRECNTRL
lh_precuneus_thickness	MR_LT_PRECUNEUS
lh_rostralanteriorcingulate_thickness	MR_LT_ROSANTCNG
lh_rostralmiddlefrontal_thickness	MR_LT_ROSMIDFRN
lh_superiorfrontal_thickness	MR_LT_SUPERFRN
lh_superiorparietal_thickness	MR_LT_SUPERPRTL
lh_superiortemporal_thickness	MR_LT_SUPERTMP
lh_supramarginal_thickness	MR_LT_SUPRAMRGNL
lh_temporalpole_thickness	MR_LT_TMPPOLE
lh_transversetemporal_thickness	MR_LT_TRANSTMP
rh_bankssts_thickness	MR_RT_SSTSBANK
rh_caudalanteriorcingulate_thickness	MR_RT_CAUDANTCNG
rh_caudalmiddlefrontal_thickness	MR_RT_CAUDMIDFRN
rh_cuneus_thickness	MR_RT_CUNEUS
rh_entorhinal_thickness	MR_RT_ENTORHINAL
rh_frontalpole_thickness	MR_RT_FRNPOLE
rh_fusiform_thickness	MR_RT_FUSIFORM
rh_inferiorparietal_thickness	MR_RT_INFRPRTL
rh_inferiortemporal_thickness	MR_RT_INFRTMP
rh_insula_thickness	MR_RT_INSULA
rh_isthmuscingulate_thickness	MR_RT_ISTHMUSCNG
rh_lateraloccipital_thickness	MR_RT_LATOCC
rh_lateralorbitofrontal_thickness	MR_RT_LATORBFRN
rh_lingual_thickness	MR_RT_LINGUAL
rh_medialorbitofrontal_thickness	MR_RT_MEDORBFRN
rh_middletemporal_thickness	MR_RT_MIDTMP
rh_paracentral_thickness	MR_RT_PARACNTRL
rh_parahippocampal_thickness	MR_RT_PARAHPCMPL
rh_parsopercularis_thickness	MR_RT_PARAOPRCLRS

rh_parsorbitalis_thickness	MR_RT_PARSORBLS
rh_parstriangularis_thickness	MR_RT_PARSTRNGLRS
rh_pericalcarine_thickness	MR_RT_PERICLCRN
rh_postcentral_thickness	MR_RT_POSTCNTRL
rh_posteriorcingulate_thickness	MR_RT_POSTCNG
rh_precentral_thickness	MR_RT_PRECNTRL
rh_precuneus_thickness	MR_RT_PRECUNEUS
rh_rostralanteriorcingulate_thickness	MR_RT_ROSANTCNG
rh_rostralmiddlefrontal_thickness	MR_RT_ROSMIDFRN
rh_superiorfrontal_thickness	MR_RT_SUPERFRN
rh_superiorparietal_thickness	MR_RT_SUPERPRTL
rh_superiortemporal_thickness	MR_RT_SUPERTMP
rh_supramarginal_thickness	MR_RT_SUPRAMRGNL
rh_temporalpole_thickness	MR_RT_TMPPOLE
rh_transversetemporal_thickness	MR_RT_TRANSTMP
Left-Accumbens-area	MR_LV_ACCUMBENS
Left-Amygdala	MR_LV_AMYGDALA
Left-Caudate	MR_LV_CAUD
Left-Cerebellum-Cortex	MR_LV_CBLL_CORTEX
Left-Cerebellum-White-Matter	MR_LV_CBLL_WM
Left-choroid-plexus	MR_LV_CHORPLEX
Left-Hippocampus	MR_LV_HIPPOCAMPUS
Left-Inf-Lat-Vent	MR_LV_INFLATVENT
Left-Lateral-Ventricle	MR_LV_LATVENT
Left-non-WM-hypointensities	MR_LV_NONWMHYPOINTENSITIES
Left-Pallidum	MR_LV_PALLIDUM
Left-Putamen	MR_LV_PUTAMEN
Left-Thalamus-Proper	MR_LV_THALAMUS
Left-VentralDC	MR_LV_VENTRALDC
Left-vessel	MR_LV_VESSEL
Left-WM-hypointensities	MR_LV_WMHYPOINTENSITIES
lh_bankssts_volume	MR_LV_SSTSBANK
lh_caudalanteriorcingulate_volume	MR_LV_CAUDANTCNG
lh_caudalmiddlefrontal_volume	MR_LV_CAUDMIDFRN
lh_cuneus_volume	MR_LV_CUNEUS
lh_entorhinal_volume	MR_LV_ENTORHINAL
lh_frontalpole_volume	MR_LV_FRNPOLE
lh_fusiform_volume	MR_LV_FUSIFORM
lh_inferiorparietal_volume	MR_LV_INFRPRTL
lh_inferiortemporal_volume	MR_LV_INFRTMP
lh_insula_volume	MR_LV_INSULA
lh_isthmuscingulate_volume	MR_LV_ISTHMUSCNG
lh_lateraloccipital_volume	MR_LV_LATOCC
lh_lateralorbitofrontal_volume	MR_LV_LATORBFRN
lh_lingual_volume	MR_LV_LINGUAL
lh_medialorbitofrontal_volume	MR_LV_MEDORBFRN
lh_middletemporal_volume	MR_LV_MIDTMP
lh_paracentral_volume	MR_LV_PARACNTRL
lh_parahippocampal_volume	MR_LV_PARAHPCMPL
lh_parsopercularis_volume	MR_LV_PARAOPRCLRS
lh_parsorbitalis_volume	MR_LV_PARSORBLS
lh_parstriangularis_volume	MR_LV_PARSTRNGLRS
lh_pericalcarine_volume	MR_LV_PERICLCRN

lh_postcentral_volume	MR_LV_POSTCNTRL
lh_posteriorcingulate_volume	MR_LV_POSTCNG
lh_precentral_volume	MR_LV_PRECNTRL
lh_precuneus_volume	MR_LV_PRECUNEUS
lh_rostralanteriorcingulate_volume	MR_LV_ROSANTCNG
lh_rostralmiddlefrontal_volume	MR_LV_ROSMIDFRN
lh_superiorfrontal_volume	MR_LV_SUPERFRN
lh_superiorparietal_volume	MR_LV_SUPERPRTL
lh_superiortemporal_volume	MR_LV_SUPERTMP
lh_supramarginal_volume	MR_LV_SUPRAMRGNL
lh_temporalpole_volume	MR_LV_TMPPOLE
lh_transversetemporal_volume	MR_LV_TRANSTMP
lhCortexVol	MR_LV_CORTEX
lhCorticalWhiteMatterVol	MR_LV_CORTICALWM
rh_bankssts_volume	MR_RV_SSTSBANK
rh_caudalanteriorcingulate_volume	MR_RV_CAUDANTCNG
rh_caudalmiddlefrontal_volume	MR_RV_CAUDMIDFRN
rh_cuneus_volume	MR_RV_CUNEUS
rh_entorhinal_volume	MR_RV_ENTORHINAL
rh_frontalpole_volume	MR_RV_FRNPOLE
rh_fusiform_volume	MR_RV_FUSIFORM
rh_inferiorparietal_volume	MR_RV_INFRPRTL
rh_inferiortemporal_volume	MR_RV_INFRTMP
rh_insula_volume	MR_RV_INSULA
rh_isthmuscingulate_volume	MR_RV_ISTHMUSCNG
rh_lateraloccipital_volume	MR_RV_LATOCC
rh_lateralorbitofrontal_volume	MR_RV_LATORBFRN
rh_lingual_volume	MR_RV_LINGUAL
rh_medialorbitofrontal_volume	MR_RV_MEDORBFRN
rh_middletemporal_volume	MR_RV_MIDTMP
rh_paracentral_volume	MR_RV_PARACNTRL
rh_parahippocampal_volume	MR_RV_PARAHPCMPL
rh_parsopercularis_volume	MR_RV_PARAOPRCLRS
rh_parsorbitalis_volume	MR_RV_PARSORBLS
rh_parstriangularis_volume	MR_RV_PARSTRNGLRS
rh_pericalcarine_volume	MR_RV_PERICLCRN
rh_postcentral_volume	MR_RV_POSTCNTRL
rh_posteriorcingulate_volume	MR_RV_POSTCNG
rh_precentral_volume	MR_RV_PRECNTRL
rh_precuneus_volume	MR_RV_PRECUNEUS
rh_rostralanteriorcingulate_volume	MR_RV_ROSANTCNG
rh_rostralmiddlefrontal_volume	MR_RV_ROSMIDFRN
rh_superiorfrontal_volume	MR_RV_SUPERFRN
rh_superiorparietal_volume	MR_RV_SUPERPRTL
rh_superiortemporal_volume	MR_RV_SUPERTMP
rh_supramarginal_volume	MR_RV_SUPRAMRGNL
rh_temporalpole_volume	MR_RV_TMPPOLE
rh_transversetemporal_volume	MR_RV_TRANSTMP
rhCortexVol	MR_RV_CORTEX
rhCorticalWhiteMatterVol	MR_RV_CORTICALWM
Right-Accumbens-area	MR_RV_ACCUMBENS
Right-Amygdala	MR_RV_AMYGDALA
Right-Caudate	MR_RV_CAUD

Right-Cerebellum-Cortex	MR_RV_CBLL_CORTEX
Right-Cerebellum-White-Matter	MR_RV_CBLL_WM
Right-choroid-plexus	MR_RV_CHORPLEX
Right-Hippocampus	MR_RV_HIPPOCAMPUS
Right-Inf-Lat-Vent	MR_RV_INFLATVENT
Right-Lateral-Ventricle	MR_RV_LATVENT
Right-non-WM-hypointensities	MR_RV_NONWMHYPOINTENSITIES
Right-Pallidum	MR_RV_PALLIDUM
Right-Putamen	MR_RV_PUTAMEN
Right-Thalamus-Proper	MR_RV_THALAMUS
Right-VentralDC	MR_RV_VENTRALDC
Right-vessel	MR_RV_VESSEL
Right-WM-hypointensities	MR_RV_WMHYPOINTENSITIES

White Matter Lesion Processing and Analysis

The preferred method for quantifying white matter damage is to obtained white matter (WM) hyperintensity volumes on FLAIR scans using the lesion segmentation toolbox (LST, Schmidt et al., 2012) implemented within SPM8. WM hyperintensity volumes were generated using a FLAIR and T1 scan by the LST (http://www.applied-statistics.de/lst.html). See LST user manual for more details. Outputs from the LST are in the same resolution as the T1 (e.g. usually 1x1x1 mm). This program was validated by comparing results against manual tracing from 16 individuals with multiple sclerosis (shown in figure 5). The dice overlap coefficient (dice=0.503) was highest with an automated lesion threshold of 0.3 and results were highly correlated between the two methods (R^2=0.934). The automated LST program systematically identifies slightly larger lesion than manual tracing. The data releases contain total white matter hyperintensity volumes.

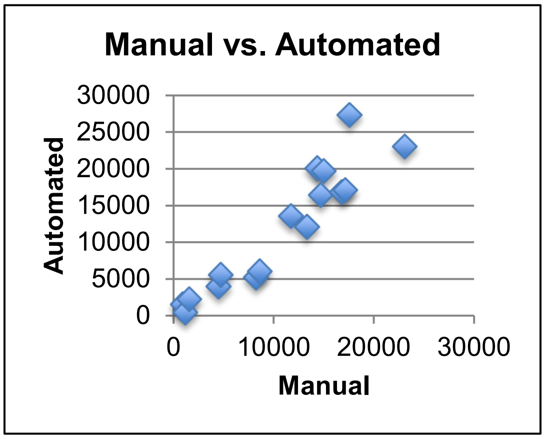

Figure 5. Manual and automated tracing WM lesion of 16 individuals with multiple sclerosis

A second potential method to quantify lesions utilizes WM hypointensity volume obtained on a T1 using the FreeSurfer segmentation. WM hypointensity volumes are provided as an output of the FreeSurfer structural pipeline. This method is currently ***NOT VALIDATED*** and it is recommended that these values should ***NOT*** be used for any analyses.

Additionally, T2/FLAIR scans may receive a qualitative rating of white matter damage (Fazekas) based on the clinical rating of periventricular and deep white matter damage by a neurologist. The Fazekas rating scale is scored according to: Fazekas 0: None or a single punctate WM hyperintensity lesion, Fazekas 1: Multiple

punctate lesions, Fazekas 2: Beginning confluency of lesions (bridging), Fazekas 3: Large confluent lesions. These white matter hyperintensity from a FLAIR scan, hypointensities from a T1, and Fazekas scores were examined in a cohort of 186 unique participants. Quality control on the scans was performed prior to processing.

Included below are two comparisons between FreeSurfer (T1 WM hypointensity) and LST results (Flair, WM hyperintensity). Figure 6 shows the difference in WM hypointensity (shown in red) and hyperintensity (shown in blue) volume between the two methods divided into categories of their Fazekas clinical rating. Figure 7 shows the correlation (R =0.91) between the FreeSurfer and LST volumes. Note that although the correlation is high, the volumes are massively different. This suggests that both are useful to identify relatively levels of pathology, but that FreeSurfer is not accurately identifying lesions. We are currently examining dice coefficients to see how closely the two metrics overlap. The WM hypointensity volume derived from FLAIRs using SPM as described above is the recommended variable to use to characterize white matter damage.

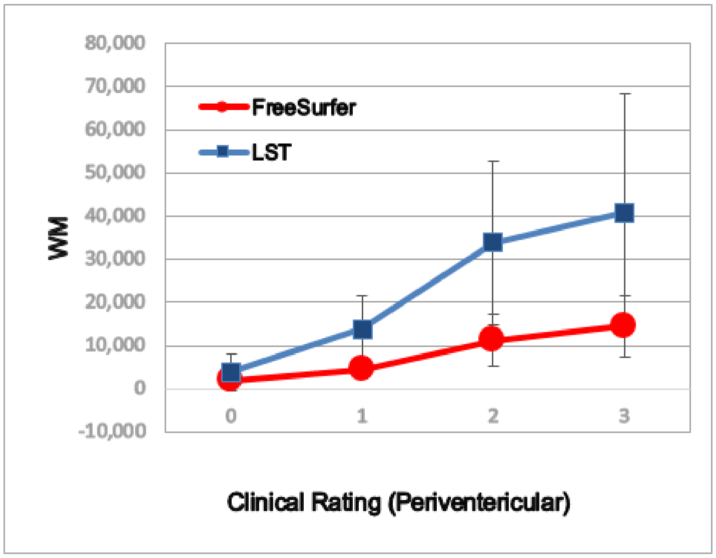

Figure 6: WM lesion obtained FreeSurfer and LST as a function of Fazekas clinical rating

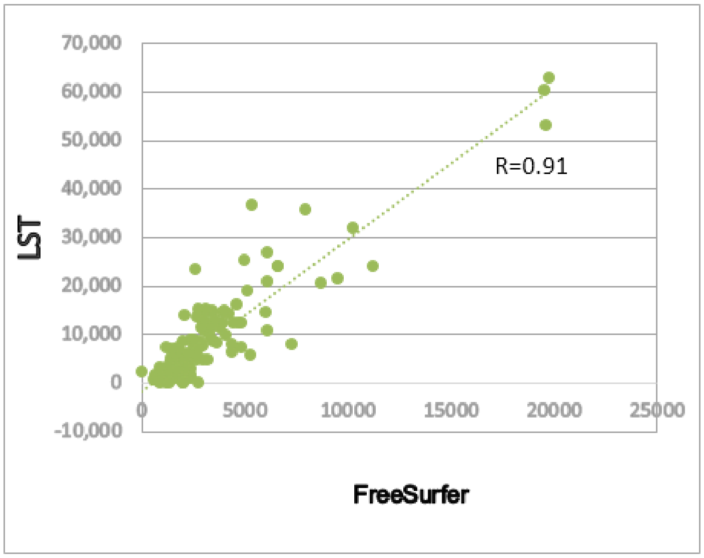

Figure 7: WM lesion obtained by FreeSurfer and LST

PET Processing

Each site underwent an initial evaluation by the ADNI PET QC site to ensure compliance with a common [11C]-Pittsburgh Compound B (PiB), [18F]-Flurodeoxyglucose (FDG), [18F]-Flortaucipir (AV-1451), and [18F]-MK-6240 PET protocol. Amyloid imaging was performed with a bolus injection of approximately 15 mCi of PiB. Dynamic imaging acquisition started either at injection for 70 minutes or 40 minutes post-injection for 30 minutes. For analysis, the PiB PET data between 40 to 70 minutes were used. Metabolic imaging with FDG-PET was performed with a 3D dynamic acquisition began 40 minutes after a bolus injection of approximately 5 mCi of FDG and lasted for 20 minutes. Tau imaging was performed with a bolus injection of approximately 8.7 mCi of AV-1451 or 5.1 mCi of MK-6240. Dynamic imaging acquisition started either at injection for 105 minutes or 75 minutes post injection for 30 minutes for AV-1451, or at injection for 110 minutes or 75 minutes post injection for 35 minutes for MK-6240. All PET images were quality controlled by the ADNI QC team.

PET imaging analyses are performed using the PET unified pipeline (PUP, https://github.com/ysu001/PUP) (Su 2013, Su 2015). PET images are smoothed to achieve a common spatial resolution of 8mm to minimize inter-scanner differences (Joshi et al., 2009). Inter-frame motion correction for the dynamic PET images is performed using standard image registration techniques (Hajnal et al., 1995) (Eisenstein et al., 2012). PET-MR registration is performed using a vector-gradient algorithm (VGM) (Rowland et al., 2005) in a symmetric fashion (i.e. average transformation for PET->MR and inverse of MR->PET was used as the final transformation matrix). By default, regional PET processing is performed based on FreeSurfer segmentation (using wmparc.mgz as the region definition), and each FreeSurfer region is analyzed. When FreeSurfer processing of matching MPRAGE image fails, PET processing then switch to analysis using expert defined manual regions. During manual processing, only a small set of regions are defined and analyzed based on previously defined rules (Mintun 2006) (Figure 8). The PET processing pipeline generates both reports of regional measurements as well as an SUVR image in the individual FreeSurfer space.

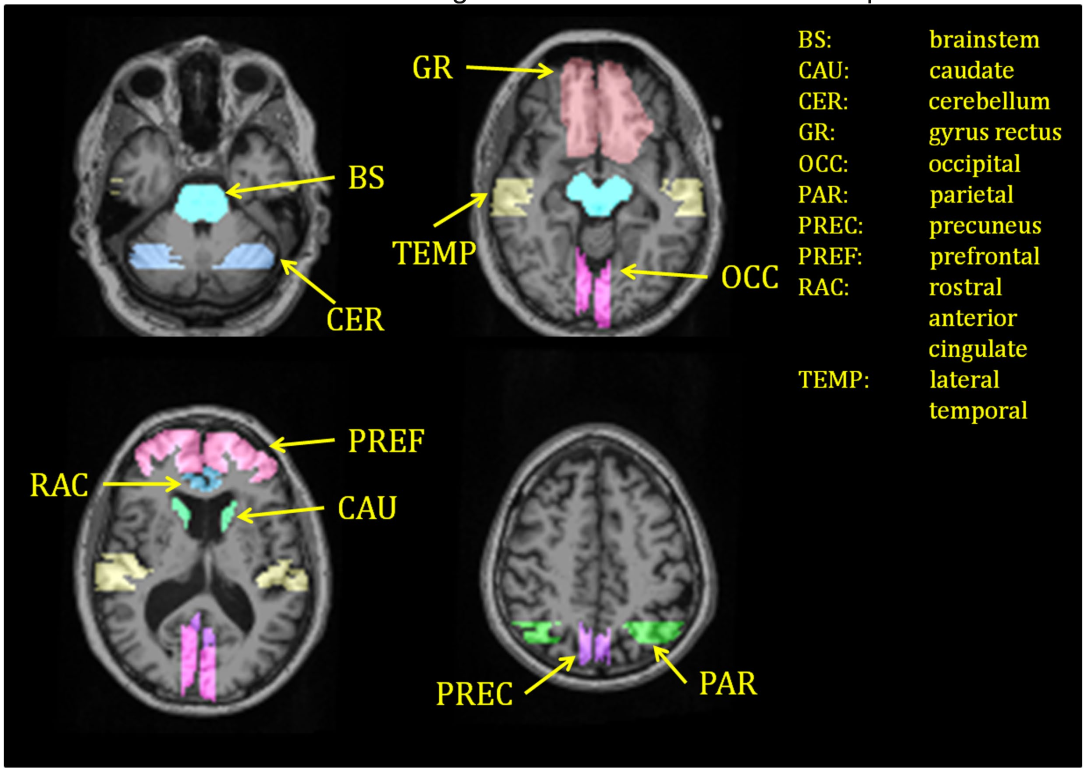

Figure 8. Example of manually defined regions on one individual.

Partial Volume Correction

As PET images have low spatial resolution, measured signals are distorted by partial volume effects (PVE). The distortion caused by PVE is a function of the size and shape of the region of interest in addition to spatial resolution of the images. In longitudinal studies, the impact of PVE is further confounded by brain atrophy due to aging and pathological changes. To account for these distortions, correction technique is implemented in our processing pipeline using a regional spread function (RSF) (Rousset 1998) based approach (Su 2015). We have demonstrated that the RSF technique was able to improve PET quantification and achieve better sensitivity to longitudinal changes in amyloid burden (Su 2015, 2016). Our standard PET processing includes results both with and without RSF partial volume correction, however, in manual processing only results without partial volume correction are available due to insufficient anatomical information as required by the RSF technique. Also, SUVR voxel-wise images are only available without partial volume correction in current analysis.

Amyloid PET Imaging Analysis

To assess global amyloid burden based on amyloid PET imaging data, the arithmetic mean of BP_{ND} or SUVRs from precuneus (PREC), prefrontal cortex (PREF), gyrus rectus (GR), and lateral temporal (TEMP) regions are defined as the mean cortical binding potential (MCBP) or mean cortical SUVR (MCSUVR). In FreeSurfer based processing, PREC is defined as the combined left and right hemisphere ctx-precuneus, PREF is defined as the left and right combined ctx-superiorfrontal and ctx-rostralmiddlefrontal regions, GR is defined as the left and right combined ctx-lateralorbitofrontal and ctx-medialorbitofrontal regions, and TEMP is defined as the left and right combined ctx-superialtemporal and ctx-middletemporal regions (Figure 9) (Su 2013). In manual region based processing, these four regions are defined based on expert experiences as illustrated in Figure 8 (Mintun 2006). Although manually defined regions and FreeSurfer regions only have a moderate degree of spatial overlap, the resulting global amyloid burden index had a high degree of agreement (Su 2013).

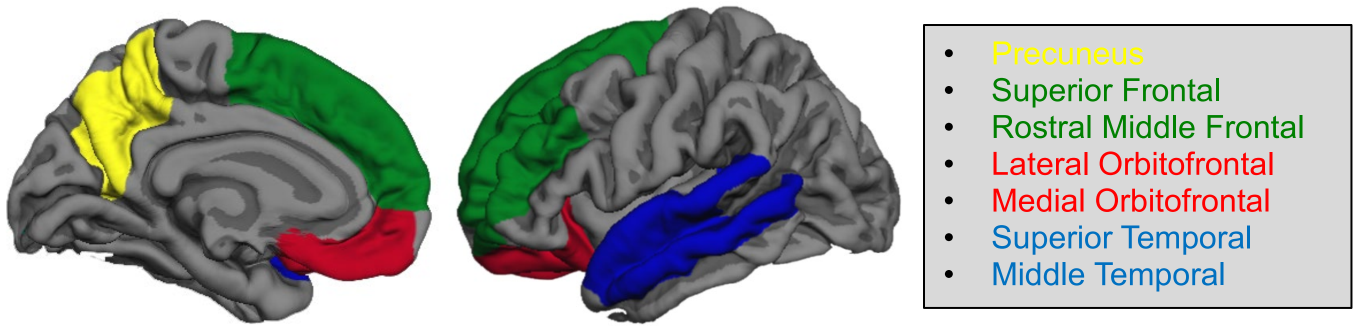

Figure 9: FreeSurfer regions for global amyloid burden index (MCBP, MCSUVR, etc.) calculation.

Using Manual ROI Data

When FreeSurfer processing of the matched MPRAGE fails, the Imaging Core will register the matched MPRAGE to the Tourneau 711-2B atlas in 222 space. Following registration, ROIs for the brainstem, gyrus rectus, prefrontal, lateral temporal, parietal, cerebellum, caudate, occipital, precuneus, and rostral AC are

manually hand-drawn on the registered T1 as described in Mintun 2006. These manual ROIs are then used for PET processing.

Whenever manual ROIs are required to process PET data for a visit, the Imaging Core will also process all within-subject longitudinal PET visits using manual ROIs to ensure consistency across visits. If a timepoint for a particular subject used manual ROIs in your analysis, Imaging Core **strongly recommends** all longitudinal data for that specific participant use the manual ROI data as well, regardless if some of the other timepoints have FreeSurfer-based PET processing. This will eliminate variance caused by differences in processing procedures.

In general, partial volume corrected (rsf) results should be used for analyses when few scans fail FreeSurfer processing. However, manual ROI PET data does not include partial volume correction due to insufficient anatomical information required by the RSF technique. Additionally, it is desirable to minimize data loss and systematic bias as many of the reasons for FreeSurfer failure (e.g., motion artefacts, high prevalence of white matter disease, etc.) are common in AD progression. For these reasons, if using both manual ROI and FreeSurfer-based PET data for an analysis, we **strongly recommend** using FreeSurfer-based data *without* partial volume correction (i.e., variables without the 'rsf' labelling). No analysis should ever combine partial volume corrected FreeSurfer-based PET data and non-partial volume corrected manual ROI-based PET data.

Centiloid Conversion for Amyloid PET

Differences in the amyloid imaging tracer, the PET acquisition, and the analysis protocol across different studies introduce considerable variability within amyloid PET imaging. This variability leads to difficulties in comparing and interpreting amyloid burden results reported from different groups (Klunk et al, 2015). To achieve comparable results, the Centiloid Working Group established a standardized scale called Centiloid to convert mean cortical SUVR and BP into a Centiloid measure of *global amyloid disposition*. If you want to use regional values, consult the Imaging Core.

The procedure and requirements to define the Centiloid scale is documented in detail in the initial Centiloid paper (Klunk et al 2015). To summarize, the Centiloid scale is defined by two anchor points: the mean amyloid burden measurement of a young control group with no amyloid pathology in their brain, represented as 0 in the Centiloid scale, and the mean amyloid burden of an AD group, represented as 100 in the Centiloid scale (level 1 calibration).   Subsequently, a Deming regression and a linear transformation are performed to calibrate the tracer and the local processing methods to the Centiloid scale (i.e. level 2 calibration). Currently, PiB has been calibrated to the Centiloid scale for both non-partial volume and partial volume correction (rsf) using standard PUP (see equations below).

The PiB-Centiloid equations were defined using a subset of the Global Alzheimer's Association Information Network dataset (GAAIN, http://www.gaain.org) for PiB (40-70 minutes post-injection) SUVR and BP measures and were processed with cerebellar cortex, whole cerebellum, or brainstem as the reference region. Different reference regions have an impact on the mean Centiloid values and we suggest using the cerebellar cortex as the reference region since it has the least variability in the young control cohort (eq. 1 through 4). We found that our implementation of the standard Centiloid analysis is strongly correlated with the published Centiloid measures for the GAAIN data set ($r^2 = 0.99$).

As any tracer or processing method can be scaled to Centiloid, new equations will be derived as new amyloid tracers and data become available. Centiloid equations may be recalibrated in the future to follow consensus of the research community.

Centiloid Equations (TOT_CORTMEAN)	
$^{PiB}\mathbf{Centiloid}_{BP} = 122.5 \times PiB\_4070\_BP - 6.2$	Eq.1
$^{PiB}\mathbf{Centiloid}_{SUVR} = 102.8 \times PiB\_4070\_SUVR - 112.2$	Eq.2
$^{PiB}\mathbf{Centiloid}_{BPRSF} = 52.0 \times PiB\_4070\_BP\_RSF - 4.2$	Eq.3
$^{PiB}\mathbf{Centiloid}_{SUVRRSF} = 40.7 \times PiB\_4070\_SUVR\_RSF - 42.9$	Eq.4

Cutoff Values for Amyloid Positivity

Traditionally, the cutoff for amyloid positivity has been established as MCBP>0.18 based on manually processed PiB data (Mintun 2006). We also established that the same cutoff could be used for FreeSurfer processing generated MCBP based on a study population of 77 participants (Su 2013). Based on this dataset, the cutoff for MCSUVRRSF was determined to be 1.42, the cutoff values for additional versions of global amyloid burden measurements that would generate best matched amyloid positivity classification as using manual MCBP=0.18 are also determined.

Amyloid Positivity Cutoffs - Cerebellar Cortex Reference Region	
PIB MCBP	0.18
PIB MCBP RSF	0.37
PIB MCSUVR	1.31
PIB MCSUVR RSF	1.42

Amyloid Positivity Cutoffs – Brainstem Reference Region	
PIB MCSUVR BS	0.79
PIB MCSUVR RSF BS	0.72

The Centiloid is a continuous scale. A Centiloid of 20 correlates with moderate neuritic plaques at autopsy. A Centiloid of 20 or higher was used as the cutoff for amyloid positivity in the AHEAD 3-45 clinical trial (Pemberton et al., 2022). Investigators should consult the literature for selecting an appropriate cut-off for their purposes.

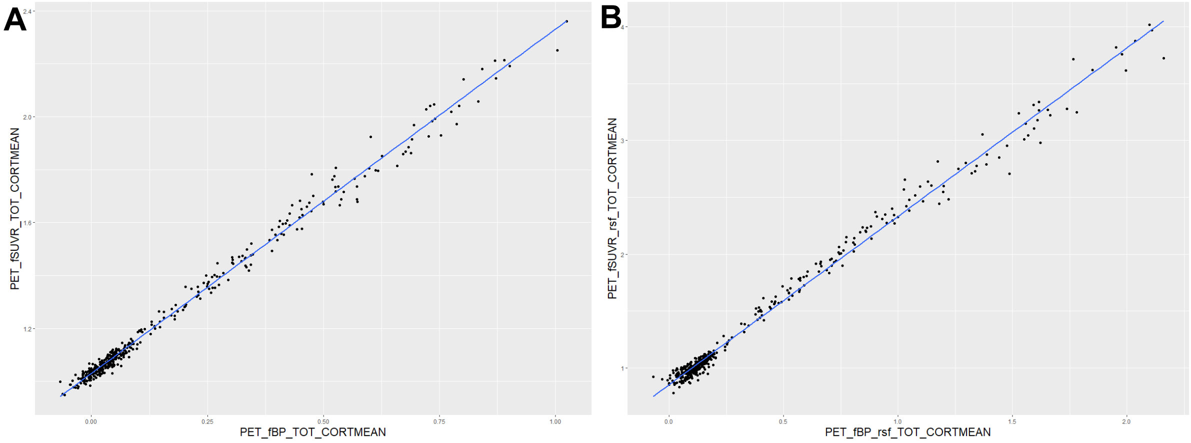

Figure 10. Comparison between PIB mean cortical BP and SUVR non-partial volume (A) and partial volume corrected (B).

Conversion Equation between BP and SUVR
**PIB MCSUVR** = $1.307 * \text{PIB MCBP} + 1.0289$
**PIB MCSUVRRSF** = $1.490 * \text{PIB MCBPRSF} + 0.848$

Tau PET Imaging Analysis

[18F]-Flortaucipir (AV-1451), previously known as T807 (Chien et al., 2014), and [18F]-MK-6240 (Hostetler et al., 2016) are currently used for *in vivo* imaging of tau pathology in DIAN studies. Standard procedure for tau PET processing is identical to amyloid PET processing except for the time window used for quantification (80-100 minutes for AV-1451, and 90-110 minutes for MK-6240).

Cutoff Values for Tau Positivity

A tau summary measure, labeled as ‘tauopathy’ in the data release, has been defined by our group to be the arithmetic mean of the partial volume corrected SUVRs from the amygdala, entorhinal cortex, inferior temporal region, and lateral occipital cortex defined using Freesurfer (Mishra et al., 2017). A tau positivity cutoff value of 1.22 has been defined using the cerebellar cortex as the reference region.

PET Variable Nomenclature

Our data naming convention provides a standard for listing the region and the processing method. Left and right brain structures use L and R. When left and right are averaged together the suffix includes the designation TOT. Six prefixes are used:

Data Type	Definition	Example Name
mBP_	manually calculated Binding Potential	mBP_TOT_ACCUMBENS
mSUVR_	manually calculated SUVR	mSUVR_TOT_ACCUMBENS
fBP_	FreeSurfer calculated Binding Potential	fBP_TOT_ACCUMBENS

fBP_rsf_	FreeSurfer calculated, partial volume corrected Binding Potential	fBP_rsf_TOT_ACCUMBENS
fSUVR_	FreeSurfer calculated SUVR	fSUVR_TOT_ACCUMBENS
fSUVR_rsf_	FreeSurfer calculated, partial volume corrected SUVR, the gold standard	fSUVR_rsf_TOT_ACCUMBENS

Tracer	Definition	Example Name
PiB	[11C]-Pittsburg Compound B	PiB_fSUVR_TOT_ACCUMBENS
FDG	[18F]-Fluodeoxyglucose	FDG_fSUVR_TOT_ACCUMBENS
AV-1451	[18F]-Flortaucipir	T80_fSUVR_TOT_ACCUMBENS
MK-6240	[18F]-MK-6240	M62_fSUVR_TOT_ACCUMBENS

The prefixes (tracer+processed_outcome) are applied to the SAS correlate suffix to create a descriptive SAS compliant name.

- **PiB_mBP_TOT_CTX_ROSANTCNG** is the [11C]PiB BP calculated using the average of the **hand-drawn** right and left rostral anterior cingulate.
- **PiB_ fSUVR_rsf_TOT_CTX_PRECUNEUS** is the [11C] PiB partial volume corrected SUVR of the gray matter in both the right and left FreeSurfer precuneus.
- **FDG_fSUVR_rsf_TOT_WM_PRECUNEUS** is the [18F] FDG partial volume corrected SUVR of the white matter calculated using the average activity in both the right and left FreeSurfer precuneus.
- **FDG_fBP_TOT_CORTMEAN** is the [18F] FDG average BP of the four MCBP cortical structures using FreeSurfer regions (TOTFS_PREFRN, TOTFS_TMP, TOTFS_GYREC, TOT_CTX_PRECUNEUS).

PET Processing Variables & Biostatistics Correlates

Structure Name	Biostat Standardizations
Accumbens_area	TOT_ACCUMBENS
Amygdala	TOT_AMYGDALA
Brain_Stem	TOT_BRAINSTEM
Caudate	TOT_CAUD
CC_Anterior	CRPCLM_ANT
CC_Central	CRPCLM_CNTRL
CC_Mid_Anterior	CRPCLM_MID_ANT
CC_Mid_Posterior	CRPCLM_MID_POST
CC_Posterior	CRPCLM_POST
Cerebellum_Cortex	TOT_CBLL_CORTEX
Cerebellum_White_Matter	TOT_CBLL_WM
choroid_plexus	TOT_CHORPLEX
ctx_bankssts	TOT_CTX_SSTSBANK
ctx_caudalanteriorcingulate	TOT_CTX_CAUDANTCNG
ctx_caudalmiddlefrontal	TOT_CTX_CAUDMIDFRN
ctx_corpuscallosum	TOT_CTX_CRPCLM
ctx_cuneus	TOT_CTX_CUNEUS
ctx_entorhinal	TOT_CTX_ENTORHINAL
ctx_frontalpole	TOT_CTX_FRNPOLE
ctx_fusiform	TOT_CTX_FUSIFORM
ctx_inferiorparietal	TOT_CTX_INFERPRTL
ctx_inferiortemporal	TOT_CTX_INFERTMP
ctx_insula	TOT_CTX_INSULA
ctx_isthmuscingulate	TOT_CTX_ISTHMUSCNG

ctx_lateraloccipital	TOT_CTX_LATOCC
ctx_lateralorbitofrontal	TOT_CTX_LATORBFRN
ctx_lh_bankssts	L_CTX_SSTSBANK
ctx_lh_caudalanteriorcingulate	L_CTX_CAUDANTCNG
ctx_lh_caudalmiddlefrontal	L_CTX_CAUDMIDFRN
ctx_lh_corpuscallosum	L_CTX_CRPCLM
ctx_lh_cuneus	L_CTX_CUNEUS
ctx_lh_entorhinal	L_CTX_ENTORHINAL
ctx_lh_frontalpole	L_CTX_FRNPOLE
ctx_lh_fusiform	L_CTX_FUSIFORM
ctx_lh_inferiorparietal	L_CTX_INFRPRTL
ctx_lh_inferiortemporal	L_CTX_INFRTMP
ctx_lh_insula	L_CTX_INSULA
ctx_lh_isthmuscingulate	L_CTX_ISTHMUSCNG
ctx_lh_lateraloccipital	L_CTX_LATOCC
ctx_lh_lateralorbitofrontal	L_CTX_LATORBFRN
ctx_lh_lingual	L_CTX_LINGUAL
ctx_lh_medialorbitofrontal	L_CTX_MEDORBFRN
ctx_lh_middletemporal	L_CTX_MIDTMP
ctx_lh_paracentral	L_CTX_PARACNTRL
ctx_lh_parahippocampal	L_CTX_PARAHPCMPL
ctx_lh_parsopercularis	L_CTX_PARSOPRCLRS
ctx_lh_parsorbitalis	L_CTX_PARSORBLS
ctx_lh_parstriangularis	L_CTX_PARSTRNGLRS
ctx_lh_pericalcarine	L_CTX_PERICLCRN
ctx_lh_postcentral	L_CTX_POSTCNTRL
ctx_lh_posteriorcingulate	L_CTX_POSTCNG
ctx_lh_precentral	L_CTX_PRECNTRL
ctx_lh_precuneus	L_CTX_PRECUNEUS
ctx_lh_rostralanteriorcingulate	L_CTX_ROSANTCNG
ctx_lh_rostralmiddlefrontal	L_CTX_ROSMIDFRN
ctx_lh_superiorfrontal	L_CTX_SUPERFRN
ctx_lh_superiorparietal	L_CTX_SUPERPRTL
ctx_lh_superiortemporal	L_CTX_SUPERTMP
ctx_lh_supramarginal	L_CTX_SUPRAMRGNL
ctx_lh_temporalpole	L_CTX_TMPPOLE
ctx_lh_transversetemporal	L_CTX_TRANSTMP
ctx_lingual	TOT_CTX_LINGUAL
ctx_medialorbitofrontal	TOT_CTX_MEDORBFRN
ctx_middletemporal	TOT_CTX_MIDTMP
ctx_paracentral	TOT_CTX_PARACNTRL
ctx_parahippocampal	TOT_CTX_PARAHPCMPL
ctx_parsopercularis	TOT_CTX_PARSOPCLRS
ctx_parsorbitalis	TOT_CTX_PARSORBLS
ctx_parstriangularis	TOT_CTX_PARSTRNGLS
ctx_pericalcarine	TOT_CTX_PERICLCRN
ctx_postcentral	TOT_CTX_POSTCNTRL
ctx_posteriorcingulate	TOT_CTX_POSTCNG
ctx_precentral	TOT_CTX_PRECNTRL
ctx_rh_bankssts	R_CTX_SSTSBANK
ctx_rh_caudalanteriorcingulate	R_CTX_CAUDANTCNG
ctx_rh_caudalmiddlefrontal	R_CTX_CAUDMIDFRN
ctx_rh_corpuscallosum	R_CTX_CRPCLM
ctx_rh_cuneus	R_CTX_CUNEUS
ctx_rh_entorhinal	R_CTX_ENTORHINAL
ctx_rh_frontalpole	R_CTX_FRNPOLE
ctx_rh_fusiform	R_CTX_FUSIFORM
ctx_rh_inferiorparietal	R_CTX_INFPRTL
ctx_rh_inferiortemporal	R_CTX_INFTMP

ctx_rh_insula	R_CTX_INSULA
ctx_rh_isthmuscingulate	R_CTX_ISTHMUSCNG
ctx_rh_lateraloccipital	R_CTX_LATOCC
ctx_rh_lateralorbitofrontal	R_CTX_LATORBFRN
ctx_rh_lingual	R_CTX_LINGUAL
ctx_rh_medialorbitofrontal	R_CTX_MEDORBFRN
ctx_rh_middletemporal	R_CTX_MIDTMP
ctx_rh_paracentral	R_CTX_PARACNTRL
ctx_rh_parahippocampal	R_CTX_PARAHPCMPL
ctx_rh_parsopercularis	R_CTX_PARSOPRCLRS
ctx_rh_parsorbitalis	R_CTX_PARSORBLS
ctx_rh_parstriangularis	R_CTX_PARSTRNGLRS
ctx_rh_pericalcarine	R_CTX_PERICLCRN
ctx_rh_postcentral	R_CTX_POSTCNTRL
ctx_rh_posteriorcingulate	R_CTX_POSTCNG
ctx_rh_precentral	R_CTX_PRECNTRL
ctx_rh_precuneus	R_CTX_PRECUNEUS
ctx_rh_rostralanteriorcingulate	R_CTX_ROSANTCNG
ctx_rh_rostralmiddlefrontal	R_CTX_ROSMIDFRN
ctx_rh_superiorfrontal	R_CTX_SUPERFRN
ctx_rh_superiorparietal	R_CTX_SUPERPRTL
ctx_rh_superiortemporal	R_CTX_SUPERTMP
ctx_rh_supramarginal	R_CTX_SUPRAMRGNL
ctx_rh_temporalpole	R_CTX_TMPPOLE
ctx_rh_transversetemporal	R_CTX_TRANSTMP
ctx_rostralanteriorcingulate	TOT_CTX_ROSANTCNG
ctx_rostralmiddlefrontal	TOT_CTX_ROSMIDFRN
ctx_superiorfrontal	TOT_CTX_SUPERFRN
ctx_superiorparietal	TOT_CTX_SUPERPRTL
ctx_superiortemporal	TOT_CTX_SUPERTMP
ctx_supramarginal	TOT_CTX_SUPRAMRGNL
ctx_temporalpole	TOT_CTX_TMPPOLE
ctx_transversetemporal	TOT_CTX_TRANSTMP
Hippocampus	TOT_HIPPOCAMPUS
Left_Accumbens_area	L_ACCUMBENS
Left_Amygdala	L_AMYGDALA
Left_Caudate	L_CAUD
Left_Cerebellum_Cortex	L_CTX_CBLL
Left_Cerebellum_White_Matter	L_WM_CBLL
Left_choroid_plexus	L_CHORPLEX
Left_Hippocampus	L_HIPPOCAMPUS
Left_Pallidum	L_PALLIDUM
Left_Putamen	L_PUTAMEN
Left_Substancia_Nigra	L_SUBSTNCA_NGRA
Left_Thalamus_Proper	L_THALAMUS
Left_UnsegmentedWhiteMatter	L_WM_UNSEGMENTED
Left_VentralDC	L_VENTRALDC
OCC_FS	TOTFS_OCC
Pallidum	TOT_PALLIDUM
Putamen	TOT_PUTAMEN
Right_Accumbens_area	R_ACCUMBENS
Right_Amygdala	R_AMYGDALA
Right_Caudate	R_CAUD
Right_Cerebellum_Cortex	R_CTX_CBLL
Right_Cerebellum_White_Matter	R_WM_CBLL
Right_choroid_plexus	R_CHORPLEX
Right_Hippocampus	R_HIPPOCAMPUS
Right_Pallidum	R_PALLIDUM
Right_Putamen	R_PUTAMEN

Right_Substancia_Nigra	R_SUBSTNCA_NGRA
Right_Thalamus_Proper	R_THALAMUS
Right_UnsegmentedWhiteMatter	R_WM_UNSEGMENTED
Right_VentralDC	R_VENTRALDC
Substancia_Nigra	TOT_SUBSTNCA_NGRA
Thalamus_Proper	TOT_THALAMUS_PRPR
UnsegmentedWhiteMatter	TOT_WM_UNSEGMENTED
VentralDC	TOT_VENTRALDC
wm_bankssts	TOT_WM_SSTSBNK
wm_caudalanteriorcingulate	TOT_WM_CAUDANTCNG
wm_caudalmiddlefrontal	TOT_WM_CAUDMIDFRN
wm_corpuscallosum	TOT_WM_CRPCLM
wm_cuneus	TOT_WM_CUNEUS
wm_entorhinal	TOT_WM_ENTORHINAL
wm_frontalpole	TOT_WM_FRNPOLE
wm_fusiform	TOT_WM_FUSIFORM
wm_inferiorparietal	TOT_WM_INFERPRTL
wm_inferiortemporal	TOT_WM_INFERTMP
wm_insula	TOT_WM_INSULA
wm_isthmuscingulate	TOT_WM_ISTHMUSCNG
wm_lateraloccipital	TOT_WM_LATOCC
wm_lateralorbitofrontal	TOT_WM_LATORBFRN
wm_lh_bankssts	L_WM_SSTSBANK
wm_lh_caudalanteriorcingulate	L_WM_CAUDANTCNG
wm_lh_caudalmiddlefrontal	L_WM_CAUDMIDFRN
wm_lh_corpuscallosum	L_WM_CRPCLM
wm_lh_cuneus	L_WM_CUNEUS
wm_lh_entorhinal	L_WM_ENTORHINAL
wm_lh_frontalpole	L_WM_FRNPOLE
wm_lh_fusiform	L_WM_FUSIFORM
wm_lh_inferiorparietal	L_WM_INFPRTL
wm_lh_inferiortemporal	L_WM_INFTMP
wm_lh_insula	L_WM_INSULA
wm_lh_isthmuscingulate	L_WM_ISTHMUSCNG
wm_lh_lateraloccipital	L_WM_LATOCC
wm_lh_lateralorbitofrontal	L_WM_LATORBFRN
wm_lh_lingual	L_WM_LINGUAL
wm_lh_medialorbitofrontal	L_WM_MEDORBFRN
wm_lh_middletemporal	L_WM_MIDTMP
wm_lh_paracentral	L_WM_PARACNTRL
wm_lh_parahippocampal	L_WM_PARAHPCMPL
wm_lh_parsopercularis	L_WM_PARSOPRCLRS
wm_lh_parsorbitalis	L_WM_PARSORBLS
wm_lh_parstriangularis	L_WM_PARSTRIANGLRS
wm_lh_pericalcarine	L_WM_PERICLCRN
wm_lh_postcentral	L_WM_POSTCNTRL
wm_lh_posteriorcingulate	L_WM_POSTCNG
wm_lh_precentral	L_WM_PRECNTRL
wm_lh_precuneus	L_WM_PRECUNEUS
wm_lh_rostralanteriorcingulate	L_WM_ROSANTCNG
wm_lh_rostralmiddlefrontal	L_WM_ROSMIDFRN
wm_lh_superiorfrontal	L_WM_SUPERFRN
wm_lh_superiorparietal	L_WM_SUPERPRTL
wm_lh_superiortemporal	L_WM_SUPERTMP
wm_lh_supramarginal	L_WM_SUPRAMRGNL
wm_lh_temporalpole	L_WM_TMPPOLE
wm_lh_transversetemporal	L_WM_TRANSTMP
wm_lingual	TOT_WM_LINGUAL
wm_medialorbitofrontal	TOT_WM_MEDORBFRN

wm_middletemporal	TOT_WM_MIDTMP
wm_paracentral	TOT_WM_PARACNTRL
wm_parahippocampal	TOT_WM_PARAHPCMPL
wm_parsopercularis	TOT_WM_PARSOPRCLRS
wm_parsorbitalis	TOT_WM_PARSORBLS
wm_parstriangularis	TOT_WM_PARSTRNGLRS
wm_pericalcarine	TOT_WM_PERICLCRN
wm_postcentral	TOT_WM_POSTCNTRL
wm_posteriorcingulate	TOT_WM_POSTCNG
wm_precentral	TOT_WM_PRECNTRL
wm_precuneus	TOT_WM_PRECUNEUS
wm_rh_bankssts	R_WM_SSTSBANK
wm_rh_caudalanteriorcingulate	R_WM_CAUDANTCNG
wm_rh_caudalmiddlefrontal	R_WM_CAUDMIDFRN
wm_rh_corpuscallosum	R_WM_CRPCLM
wm_rh_cuneus	R_WM_CUNEUS
wm_rh_entorhinal	R_WM_ENTORHINAL
wm_rh_frontalpole	R_WM_FRNPOLE
wm_rh_fusiform	R_WM_FUSIFORM
wm_rh_inferiorparietal	R_WM_INFERIORPRTL
wm_rh_inferiortemporal	R_WM_INFERIORTMP
wm_rh_insula	R_WM_INSULA
wm_rh_isthmuscingulate	R_WM_ISTHMUSCNG
wm_rh_lateraloccipital	R_WM_LATOCC
wm_rh_lateralorbitofrontal	R_WM_LATORBFRN
wm_rh_lingual	R_WM_LINGUAL
wm_rh_medialorbitofrontal	R_WM_MEDORBFRN
wm_rh_middletemporal	R_WM_MIDTMP
wm_rh_paracentral	R_WM_PARACNTRL
wm_rh_parahippocampal	R_WM_PARAHPCMPL
wm_rh_parsopercularis	R_WM_PARSOPRCLRS
wm_rh_parsorbitalis	R_WM_PARSORBLS
wm_rh_parstriangularis	R_WM_PARSTRNGLRS
wm_rh_pericalcarine	R_WM_PERICLCRN
wm_rh_postcentral	R_WM_POSTCNTRL
wm_rh_posteriorcingulate	R_WM_POSTCNG
wm_rh_precentral	R_WM_PRECNTRL
wm_rh_precuneus	R_WM_PRECUNEUS
wm_rh_rostralanteriorcingulate	R_WM_ROSANTCNG
wm_rh_rostralmiddlefrontal	R_WM_ROSMIDFRN
wm_rh_superiorfrontal	R_WM_SUPERFRN
wm_rh_superiorparietal	R_WM_SUPERPRTL
wm_rh_superiortemporal	R_WM_SUPERTMP
wm_rh_supramarginal	R_WM_SUPRAMRGNL
wm_rh_temporalpole	R_WM_TMPPOLE
wm_rh_transversetemporal	R_WM_TRANSTMP
wm_rostralanteriorcingulate	TOT_WM_ROSANTCNG
wm_rostralmiddlefrontal	TOT_WM_ROSMIDFRN
wm_superiorfrontal	TOT_WM_SUPERFRN
wm_superiorparietal	TOT_WM_SUPERPRTL
wm_superiortemporal	TOT_WM_SUPERTMP
wm_supramarginal	TOT_WM_SUPRAMRGNL
wm_temporalpole	TOT_WM_TMPPOLE
wm_transversetemporal	TOT_WM_TRANSTMP
ctx_precuneus	TOT_CTX_PRECUNEUS
GR_FS	TOTFS_GYREC
PREF_FS	TOTFS_PREFRN
TEMP_FS	TOTFS_TMP
MCBP	TOT_CORTMEAN

References

Chien, D. T., Bahri, S., Szardenings, A. K., Walsh, J. C., Mu, F., Xia, C., Su, M.-Y., Shankle, W. R., Elizarov, A., & Kolb, H. C. Early clinical PET imaging results with the novel PHF-tau radioligand [18F]-T807. J Alzheimer's Dis 2014; 34: 457-468. https://doi.org/10.3233/JAD-122059.

Dincer, A., Gordon, B. A., Hari-Raj, A., Keefe, S. J., Flores, S., McKay, N. S., … L.S. Benzinger, T. Comparing cortical signatures of atrophy between late-onset and autosomal dominant Alzheimer disease. *Neuroimage Clin* 2020; 28: 102491. https://doi.org/10.1016/j.nicl.2020.102491

Eisenstein, S.A., Koller, J.M., Piccirillo, M., Kim, A., Antenor-Dorsey, J.A., Videen, T.O., Snyder, A.Z., Karimi, M., Moerlein, S.M., Black, K.J., Perlmutter, J.S., Hershey, T. Characterization of extrastriatal D2 in vivo specific binding of [(1)(8)F](N-methyl)benperidol using PET. *Synapse* 2012; 66: 770-780.

Fischl B. FreeSurfer. *Neuroimage* 2012; 62(2):774-781. PMCID: PMC: 222857

Frouin V, Comtat C, Reilhac A, Gre´goire MC. Correction of Partial-Volume Effect for PET Striatal Imaging: Fast Implementation and Study of Robustness. *J Nucl Med* 2002; 43:1715–1726

Jack CR et al. The Alzheimer's disease neuroimaging initiative (ADNI): MRI methods. *J Magn Reson Imaging* 2008; 27: 685–691.

Jack CR Jr et al. Update on the Magnetic Resonance Imaging core of the Alzheimer's Disease Neuroimaging Initiative. *Alzheimers Dement* 2010; 6: 212–220.

Hajnal, J.V., Saeed, N., Soar, E.J., Oatridge, A., Young, I.R., Bydder, G.M., 1995. A registration and interpolation procedure for subvoxel matching of serially acquired MR images. *J Comput Assist Tomogr* 19, 289-296.

Han X, Jovicich J, Salat D, van der Kouwe A, Quinn B, Czanner S, Busa E, Pacheco J, Albert M, Killiany R, Maguire P, Rosas D, Makris N, Dale A, Dickerson B, Fischl B. Reliability of MRI-derived measurements of human cerebral cortical thickness: the effects of field strength, scanner upgrade and manufacturer. *Neuroimage* 2006;32(1):180-94. Epub 2006/05/03. doi: 10.1016/j.neuroimage.2006.02.051. PubMed PMID: 16651008.

Hostetler E. D., Waliji, A. M., Zeng, Z., Miller, P., Bennacef, I., Salinas, C., … Evelhoch, J. L. Preclinical characterization of 18F-MK-6240, a promising PET tracer for *in vivo* quantification of human neurofibrillary tangles. *J Nuc Med* 2016*;* 57: 1599-1606.

Joshi, A., Koeppe, R.A., Fessler, J.A. Reducing between scanner differences in multi-center PET studies. *Neuroimage* 2009; 46: 154-159.

Marcus, DS, Archie, KA, Olsen, T, Ramaratnam, M. The open source neuroimaging research enterprise. *J. Digital Imaging* 2007; 20:130-138.

McCormick LM, Ziebell S, Nopoulos P, Cassell M, Andreasen NC, Brumm M. Anterior Cingulate Cortex: An MRI-based parcellation method. *NeuroImage* 2006; 32:1167-1175.

McKay, N. S., Gordon, B. A., Hornbeck, R. C., Jack, C. R., Koeppe, R., Flores, S., ... & Dominantly Inherited Alzheimer Network. Neuroimaging within the Dominantly Inherited Alzheimer's Network (DIAN): PET and MRI. *bioRxiv* 2022.

Mintun MA, LaRossa GN, Sheline YI, Dence CS, Lee SY, Mach RH, Klunk WE, Mathis CA, DeKosky ST, Morris JC. [11C]PIB in a nondemented population: Potential antecedent marker of Alzheimer disease. *Neurology* 2006; 67; 446-452

Mishra, S., Gordon, B. A., Su, Y., Christensen, J., Friedrichsen, K., Jackson, K., Benzinger, T. L. S. (2017). AV-1451 PET imaging of tau pathology in preclinical Alzheimer disease: Defining a summary measure. *Neuroimage* 2017; 161: 171-178. https://doi.org/10.1016/j.neuroimage.2017.07.050.

Morris, JC, Roe, CM, Xiong, C, Fagan, AM, Goate, AM, Holtzman, DM, Mintun, MA. APOE predicts amyloid-beta but not tau Alzheimer pathology in cognitively normal aging. *Ann Neurol* 2010; 67:122-131.

Pemberton, HG, Collij, LE, Heeman, F, Bollack, A, Shekari, M, Salvadó, G, Alves, IL, Garcia DV, Battle, M, Buckley, C, Stephens, AW, Bullich, S, Garibotto, V, Barkhof, F, Gispert, JD, Farrar, G. Quantificiation of amyloid PET for future clinical use: a state-of-the-art review. *European Journal of Nuclear Medicine and Molecular Imaging* 2022; 49: 3508-3528.

Robb, R.A., Hanson, D.P., Karwoski, R.A., Larson, A.G., Workman, E.L., Stacy, M.C. Analyze: a comprehensive, operator-interactive software package for multidimensional medical image display and analysis. *Comput Med Imaging Graph* 1989; 13:433-454.

Rousset O, Ma Y, Evans A. Correction for partial volume effects in PET: principle and validation. *J Nucl Med* 1998; 39: 904–911.

Rousset O, Zaidi H. Correction of partial volume effects in emission tomography. In: Zaidi H, ed- itor. Quantitative analysis of nuclear medicine images. New York: Springer; 2006. p. 236–271.

Rowland DJ, Garbow JR, Laforest R, Snyder AZ. 2005 Registration of [18F]FDG microPET and small-animal MRI. *Nucl Med Biol* 2005; 32: 567-572

Schmidt P, Gaser C, Arsic M, Buck D, Förschler A, Berthele A, Hoshi M, Ilg R, Schmid VJ, Zimmer C, Hemmer B, Mühlau M. An automated tool for detection of FLAIR-hyperintense white-matter lesions in Multiple Sclerosis. *Neuroimage* 2012; 59(4): 3774-3783

Su Y, D'Angelo GM, Vlassenko AG, Zhou GF, Snyder AZ, Marcus DS, et al. Quantitative Analysis of PiB-PET with FreeSurfer ROIs. *PLoS One* 2013; 8(11): e73377. PMCID: 3819320

Su Y, Blazey TM, Snyder AZ, Raichle ME, Marcus DS, Ances BM, Bateman RJ, Cairns NJ, Aldea P, Cash L, Christensen JJ, Friedrichsen K, Hornbeck RC, Farrar AM, Owen CJ, Mayeux R, Brickman AM, Klunk W, Price JC, Thompson PM, Ghetti B, Saykin AJ, Sperling RA, Johnson KA, Schofield PR, Buckles V, Morris JC, Benzinger TL, Network DIA. Partial volume correction in quantitative amyloid imaging. *Neuroimage* 2015; 107: 55-64. PMCID: 4300252.

Su Y, Blazey TM, Owen CJ, Christensen JJ, Friedrichsen K, Joseph-Mathurin N, Wang Q, Hornbeck RC, Ances BM, Snyder AZ, Cash LA, Koeppe RA, Klunk WE, Galasko D, Brickman AM, McDade E, Ringman JM, Thompson PM, Saykin AJ, Ghetti B, Sperling RA, Johnson KA, Salloway SP, Schofield PR, Masters CL, Villemagne VL, Fox NC, Forster S, Chen K, Reiman EM, Xiong C, Marcus DS, Weiner MW, Morris JC, Bateman RJ, Benzinger TL, Dominantly Inherited Alzheimer N. Quantitative Amyloid Imaging in Autosomal Dominant Alzheimer's Disease: Results from the DIAN Study Group. *PLoS One* 2016; 11(3): +`e0152082. PMCID: PMC4807073.

Talairach J, Tournoux P. Co-planar Stereotaxic Atlas of the Human Brain: 3-D Proportional System: An Approach to Cerebral Imaging. Stuttgart, Germany: Thieme Medical Publishers; 1988.

NEUROPATHOLOGY DATA SET

Data Element Dictionary

Version 11, September 2020

Copyright© 2006, 2008, 2014, 2020 University of Washington

Created and published by the Neuropathology Steering Committee of the ADC Program and the National Alzheimer's Coordinating Center (Walter A. Kukull, PhD, Director). All rights reserved.

This publication was funded by the National Institutes of Health through the National Institute on Aging (Cooperative Agreement U01 AG016976)

This DED last modified April 13, 2021.

NACC
National Alzheimer's Coordinating Center

Glossary of terms

**Question number**	Indicates order of appearance on the NACC Neuropathology form
**Data element name**	For non-fixed format files, data element name must match exactly.
**Version**	11
**NP form question**	The question as it appears on the NACC Neuropathology Form
**Length of field**	Length of this data element
**Column positions**	For fixed-field formats, column numbers for this data element
**Data type**	Data element type as numerical or character
**Allowable codes**	List of codes with mapping instructions
**Blanks**	Instructions for blanks
**Skips**	Instructions for skip patterns
**Comments**	Other instructions as needed

Form header

Question number	0
Data element name	**ADCID**
Version	11
NP question	Center ID
Length of field	2
Column positions	1 – 2
Data type	Numeric
Allowable codes	1-99; use your Center ID as displayed in submission system or Portal
Comment	**Note: ADCID is replaced by a randomly generated NACCADC in research data sets generated by NACC.**

NP Form

Question number	1
Data element name	**PTID**
Version	11
NP question	UDS/MDS patient ID
Length of field	10
Column positions	4 – 13
Data type	Character
Allowable codes	Follow your Center's UDS/MDS patient ID scheme.
Comment	UDS/MDS patient ID must be unique within data set from your Center (no duplicates). UDS/MDS patient ID for each subject must be the same at each data submission; UDS/MDS patient ID cannot change once it has been assigned by your Center. **PTID** is the same for a given subject at both the UDS data freeze and Neuropathology data submission.<br>**NOTE: PTID is replaced by a randomly generated NACCID in research data sets generated by NACC.**

Question number	2a
Data element name	**NPFORMMO**
Version	11
NP question	Date form completed: month
Length of field	2
Column positions	15 – 16
Data type	Numeric
Allowable codes	1 – 12

Question number	2b
Data element name	**NPFORMDY**
Version	11
NP question	Date form completed: day
Length of field	2
Column positions	18 – 19
Data type	Numeric
Allowable codes	1 – 31

Question number	2c
Data element name	**NPFORMYR**
Version	11
NP question	Date form completed: year
Length of field	4
Column positions	21 – 24
Data type	Numeric
Allowable codes	2001 – current year

Question number	3
Data element name	**NPID**
Version	11
NP question	Neuropath ID
Length of field	10
Column positions	26 – 35
Data type	Character
Allowable codes	Any text or numbers except for single quotes, double quotes, ampersands, or percentage signs.

Question number	4
Data element name	**NPSEX**
Version	11
NP question	Subject's sex
Length of field	1
Column positions	37 – 37
Data type	Numeric
Allowable codes	1 = Male<br>2 = Female

Question number	5
Data element name	**NPDAGE**
Version	11
NP form question	Age at death
Length of field	3
Column positions	39 – 41
Data type	Numeric
Allowable codes	0–130

Question number	6a
Data element name	**NPDODMO**
Version	11
NP form question	Date of death: month
Length of field	2
Column positions	43 – 44
Data type	Numeric
Allowable codes	1 – 12

Question number	6b
Data element name	**NPDODDY**
Version	11
NP form question	Date of death: day
Length of field	2
Column positions	46 – 47
Data type	Numeric
Allowable codes	1 – 31

Question number	6c
Data element name	**NPDODYR**
Version	11
NP form question	Date of death: year
Length of field	4
Column positions	49 – 52
Data type	Numeric
Allowable codes	1984 – current year

Question number	7
Data element name	**NPPMIH**
Version	11
NP form question	Postmortem interval
Length of field	4
Column positions	54 – 57
Data type	Numeric
Allowable codes	00.0 – 98.9<br>99.9 = unknown

Question number	8
Data element name	**NPFIX**
Version	11
NP form question	Fixative
Length of field	1
Column positions	59 – 59
Data type	Numeric
Allowable codes	1 = Formalin<br>2 = Paraformaldehyde<br>7 = Other specify

Question number	8a
Data element name	**NPFIXX**
Version	11
NP form question	Fixative other specify
Length of field	30
Column positions	61 – 90
Data type	Character
Allowable codes	Any text or numbers except for single quotes, double quotes, ampersands, or percentage signs.
Blanks	Blank if #8, NPFIX, ≠ 7 (Other)

Question number	9a
Data element name	**NPWBRWT**
Version	11
NP form question	Whole brain weight
Length of field	4
Column positions	92 – 95
Data type	Numeric
Allowable codes	100 – 2500<br>9999 = unknown

Question number	9b
Data element name	**NPWBRF**
Version	11
NP form question	Fresh or fixed weight
Length of field	1
Column positions	97 – 97
Data type	Numeric
Allowable codes	1 = Fresh<br>2 = Fixed<br>8 = N/A

Question number	9c1
Data element name	**NPGRCCA**
Version	11
NP form question	Severity of gross findings — cerebral cortex atrophy
Length of field	1
Column positions	99 – 99
Data type	Numeric
Allowable codes	0 = None<br>1 = Mild<br>2 = Moderate<br>3 = Severe<br>8 = Not assessed<br>9 = Missing/unknown

Question number	9c2
Data element name	**NPGRLA**
Version	11
NP form question	Severity of gross findings — lobar atrophy
Length of field	1
Column positions	101–101
Data type	Numeric
Allowable codes	0 = None<br>1 = Yes<br>8 = Not assessed<br>9 = Missing/unknown

Question number	9c3
Data element name	**NPGRHA**
Version	11
NP form question	Severity of gross findings — hippocampus atrophy
Length of field	1
Column positions	103–103
Data type	Numeric
Allowable codes	0 = None<br>1 = Mild<br>2 = Moderate<br>3 = Severe<br>8 = Not assessed<br>9 = Missing/unknown

Question number	9c4
Data element name	**NPGRSNH**
Version	11
NP form question	Severity of gross findings — substantia nigra hypopigmentation
Length of field	1
Column positions	105–105
Data type	Numeric
Allowable codes	0 = None<br>1 = Mild<br>2 = Moderate<br>3 = Severe<br>8 = Not assessed<br>9 = Missing/unknown

Question number	9c5
Data element name	**NPGRLCH**
Version	11
NP form question	Severity of gross findings — l. ceruleus hypopigmentation
Length of field	1
Column positions	107–107
Data type	Numeric
Allowable codes	0 = None<br>1 = Mild<br>2 = Moderate<br>3 = Severe<br>8 = Not assessed<br>9 = Missing/unknown

Question number	9c6
Data element name	**NPAVAS**
Version	11
NP form question	Severity of gross findings — atherosclerosis (of the circle of Willis)
Length of field	1
Column positions	109–109
Data type	Numeric
Allowable codes	0 = None<br>1 = Mild<br>2 = Moderate<br>3 = Severe<br>8 = Not assessed<br>9 = Missing/unknown

Question number	10a
Data element name	**NPTAN**
Version	11
NP form question	Tau antibody
Length of field	1
Column positions	111–111
Data type	Numeric
Allowable codes	1 = Non-phospho specific<br>2 = PHF1<br>3 = CP13<br>4 = AT8<br>7 = Other specify<br>8 = Not assessed

Question number	10a1
Data element name	**NPTANX**
Version	11
NP form question	Tau antibody other specify
Length of field	30
Column positions	113–142
Data type	Character
Allowable codes	Any text or numbers except for single quotes, double quotes, ampersands, or percentage signs.
Blanks	Blank if #10a, NPTAN, ≠ 7 (Other)

Question number	10b
Data element name	**NPABAN**
Version	11
NP form question	Amyloid beta antibody
Length of field	1
Column positions	144–144
Data type	Numeric
Allowable codes	1 = 4G8<br>2 = 10D5<br>7 = Other (specify)<br>8 = Not assessed

Question number	10b1
Data element name	**NPABANX**
Version	11
NP form question	Amyloid beta antibody other specify
Length of field	30
Column positions	146–175
Data type	Character
Allowable codes	Any text or numbers except for single quotes, double quotes, ampersands, or percentage signs.
Blanks	Blank if #10b, NPABAN, ≠ 7 (Other)

Question number	10c
Data element name	**NPASAN**
Version	11
NP form question	Alpha synuclein antibody
Length of field	1
Column positions	177–177
Data type	Numeric
Allowable codes	1 = Non-phospho specific (e.g., LB509)<br>2 = Phospho-specific (e.g., pSYN#64)<br>7 = Other (specify)<br>8 = Not assessed

Question number	10c1
Data element name	**NPASANX**
Version	11
NP form question	Alpha synuclein antibody other specify
Length of field	30
Column positions	179 – 208
Data type	Character
Allowable codes	Any text or numbers except for single quotes, double quotes, ampersands, or percentage signs.
Blanks	Blank if #10c, NPASAN, ≠ 7 (Other)

Question number	10d
Data element name	**NPTDPAN**
Version	11
NP form question	TDP-43 antibody
Length of field	1
Column positions	210 – 210
Data type	Numeric
Allowable codes	1 = Non-phospho specific<br>2 = Phospho-specific<br>7 = Other (specify)<br>8 = Not assessed

Question number	10d1
Data element name	**NPTDPANX**
Version	11
NP form question	TDP-43 antibody other specify
Length of field	30
Column positions	212 – 241
Data type	Character
Allowable codes	Any text or numbers except for single quotes, double quotes, ampersands, or percentage signs.
Blanks	Blank if #10d, NPTDPAN, ≠ 7 (Other)

Question number	10e1
Data element name	**NPHISMB**
Version	11
NP form question	Histochemical stains — modified Bielschowsky
Length of field	1
Column positions	243 – 243
Data type	Numeric
Allowable codes	0 = No<br>1 = Yes

Question number	10e2
Data element name	**NPHISG**
Version	11
NP form question	Histochemical stains — Gallyas
Length of field	1
Column positions	245 – 245
Data type	Numeric
Allowable codes	0 = No<br>1 = Yes

Question number	10e3
Data element name	**NPHISSS**
Version	11
NP form question	Histochemical stains — other silver stain
Length of field	1
Column positions	247 – 247
Data type	Numeric
Allowable codes	0 = No<br>1 = Yes

Question number	10e4
Data element name	**NPHIST**
Version	11
NP form question	Histochemical stains — thioflavin
Length of field	1
Column positions	249 – 249
Data type	Numeric
Allowable codes	0 = No<br>1 = Yes

Question number	10e5
Data element name	**NPHISO**
Version	11
NP form question	Histochemical stains — other
Length of field	1
Column positions	251 – 251
Data type	Numeric
Allowable codes	0 = No<br>1 = Yes

Question number	10ex
Data element name	**NPHISOX**
Version	11
NP form question	Histochemical stains — other specify
Length of field	30
Column positions	253 – 282
Data type	Character
Allowable codes	Any text or numbers except for single quotes, double quotes, ampersands, or percentage signs.
Blanks	Blank if #10e5, NPHISO, ≠ 1 (Yes)

Question number	11a
Data element name	**NPTHAL**
Version	11
NP form question	Thal phase for amyloid plaques (A Score)
Length of field	1
Column positions	284 – 284
Data type	Numeric
Allowable codes	0 = Phase 0 (A0)<br>1 = Phase 1 (A1)<br>2 = Phase 2 (A1)<br>3 = Phase 3 (A2)<br>4 = Phase 4 (A3)<br>5 = Phase 5 (A3)<br>8 = Not assessed<br>9 = Missing/unknown

Question number	11b
Data element name	**NPBRAAK**
Version	11
NP form question	Braak stage for neurofibrillary degeneration (B score)
Length of field	1
Column positions	286 – 286
Data type	Numeric
Allowable codes	0 = Stage 0: AD-type neurofibrillary degeneration not present (B0)<br>1 = Stage I (B1)<br>2 = Stage II (B1)<br>3 = Stage III (B2)<br>4 = Stage IV (B2)<br>5 = Stage V (B3)<br>6 = Stage VI (B3)<br>7 = The presence of a tauopathy (other than aging/AD) precludes Braak staging<br>8 = Not assessed<br>9 = Missing/unknown

Question number	11c
Data element name	**NPNEUR**
Version	11
NP form question	CERAD score for density of neocortical neuritic plaques (C score)
Length of field	1
Column positions	288 – 288
Data type	Numeric
Allowable codes	0 = No neuritic plaques (C0)<br>1 = Sparse neuritic plaques (C1)<br>2 = Moderate neuritic plaques (C2)<br>3 = Frequent neuritic plaques (C3)<br>8 = Not assessed<br>9 = Missing/unknown

Question number	11d
Data element name	**NPADNC**
Version	11
NP form question	NIA-AA ADNC
Length of field	1
Column positions	290 – 290
Data type	Numeric
Allowable codes	0 = Not AD<br>1 = Low ADNC<br>2 = Intermediate ADNC<br>3 = High ADNC<br>8 = Not assessed<br>9 = Missing/unknown

Question number	11e1
Data element name	**NPDIFF**
Version	11
NP form question	CERAD semi-quantitative score for diffuse plaques
Length of field	1
Column positions	292 – 292
Data type	Numeric
Allowable codes	0 = No diffuse plaques<br>1 = Sparse diffuse plaques<br>2 = Moderate diffuse plaques<br>3 = Frequent diffuse plaques<br>8 = Not assessed<br>9 = Missing/unknown

Question number	11e2
Data element name	**NPAMY**
Version	11
NP form question	Cerebral amyloid angiopathy
Length of field	1
Column positions	294 – 294
Data type	Numeric
Allowable codes	0 = None<br>1 = Mild<br>2 = Moderate<br>3 = Severe<br>8 = Not assessed<br>9 = Missing/unknown

Question number	12a
Data element name	**NPINF**
Version	11
NP form question	Old infarcts observed grossly, including lacunes
Length of field	1
Column positions	296 – 296
Data type	Numeric
Allowable codes	0 = No<br>1 = Yes<br>8 = Not assessed<br>9 = Missing/unknown
Skip	If #12a, NPINF, ≠ 1, then skip to #12b, NPHEMO

Question number	121a
Data element name	**NPINF1A**
Version	11
NP form question	Number of infarcts — cerebral cortex
Length of field	2
Column positions	298 – 299
Data type	Numeric
Allowable codes	0 – 87<br>88 = N/A<br>99 = Unknown
Blanks	Blank if #12a, NPINF, ≠ 1 (Yes)

Question number	121b
Data element name	**NPINF1B**
Version	11
NP form question	Largest Infarct — cerebral cortex
Length of field	4
Column positions	301 – 304
Data type	Numeric
Allowable codes	0.0–20.0<br>88.8 = N/A<br>99.9 = Unknown/missing
Blanks	Blank if #12a, NPINF, ≠ 1 (Yes)<br>Blank if #121a, NPINF1A, = 0, 88, or 99

Question number	121d
Data element name	**NPINF1D**
Version	11
NP form question	Second largest infarct — cerebral cortex
Length of field	4
Column positions	306 – 309
Data type	Numeric
Allowable codes	0.0–20.0<br>88.8 = N/A<br>99.9 = Unknown/missing
Blanks	Blank if #12a, NPINF, ≠ 1 (Yes)<br>Blank if #121a, NPINF1A, < 2, or = 88 or 99

Question number	121f
Data element name	**NPINF1F**
Version	11
NP form question	Third largest infarct — cerebral cortex
Length of field	4
Column positions	311 – 314
Data type	Numeric
Allowable codes	0.0–20.0<br>88.8 = N/A<br>99.9 = Unknown/missing
Blanks	Blank if #12a, NPINF, ≠ 1 (Yes)<br>Blank if #121a, NPINF1A, < 3, or = 88 or 99

Question number	122a
Data element name	**NPINF2A**
Version	11
NP form question	Number of infarcts — subcortical cerebral white matter and periventricular white matter
Length of field	2
Column positions	316 – 317
Data type	Numeric
Allowable codes	0–87<br>88 = N/A<br>99 = Unknown
Blanks	Blank if #12a, NPINF, ≠ 1 (Yes)

Question number	122b
Data element name	**NPINF2B**
Version	11
NP form question	Largest infarct — white matter
Length of field	4
Column positions	319 – 322
Data type	Numeric
Allowable codes	0.0–20.0<br>88.8 = N/A<br>99.9 = Unknown/missing
Blanks	Blank if #12a, NPINF, ≠ 1 (Yes)<br>Blank if #122a, NPINF2A, = 0, 88, or 99

Question number	122d
Data element name	**NPINF2D**
Version	11
NP form question	Second largest infarct — white matter
Length of field	4
Column positions	324 – 327
Data type	Numeric
Allowable codes	0.0–20.0<br>88.8 = N/A<br>99.9 = Unknown/missing
Blanks	Blank if #12a, NPINF, ≠ 1 (Yes)<br>Blank if #122a, NPINF2A, < 2, or = 88 or 99

Question number	122f
Data element name	**NPINF2F**
Version	11
NP form question	Third largest infarct — white matter
Length of field	4
Column positions	329–332
Data type	Numeric
Allowable codes	0.0–20.0<br>88.8 = N/A<br>99.9 = Unknown/missing
Blanks	Blank if #12a, NPINF, ≠ 1 (Yes)<br>Blank if #122a, NPINF2A, < 3, or = 88 or 99

Question number	123a
Data element name	**NPINF3A**
Version	11
NP form question	Number of infarcts — deep cerebral gray matter or internal capsule
Length of field	2
Column positions	334–335
Data type	Numeric
Allowable codes	0–87<br>88 = N/A<br>99 = Unknown
Blanks	Blank if #12a, NPINF, ≠ 1 (Yes)

Question number	123b
Data element name	**NPINF3B**
Version	11
NP form question	Largest infarct — deep cerebral gray matter or internal capsule
Length of field	4
Column positions	337–340
Data type	Numeric
Allowable codes	0.0–20.0<br>88.8 = N/A<br>99.9 = Unknown/missing
Blanks	Blank if #12a, NPINF, ≠ 1 (Yes)<br>Blank if #123a, NPINF3A, = 0, 88, or 99

Question number	123d
Data element name	**NPINF3D**
Version	11
NP form question	Second largest infarct — deep cerebral gray matter or internal capsule
Length of field	4
Column positions	342 – 345
Data type	Numeric
Allowable codes	0.0–20.0<br>88.8 = N/A<br>99.9 = Unknown/missing
Blanks	Blank if #12a, NPINF, ≠ 1 (Yes)<br>Blank if #123a, NPINF3A, < 2, or = 88 or 99

Question number	123f
Data element name	**NPINF3F**
Version	11
NP form question	Third largest infarct — deep cerebral gray matter or internal capsule
Length of field	4
Column positions	347 – 350
Data type	Numeric
Allowable codes	0.0–20.0<br>88.8 = N/A<br>99.9 = Unknown/missing
Blanks	Blank if #12a, NPINF, ≠ 1 (Yes)<br>Blank if #123a, NPINF3A, < 3, or = 88 or 99

Question number	124a
Data element name	**NPINF4A**
Version	11
NP form question	Number of infarcts — brainstem or cerebellum
Length of field	2
Column positions	352 – 353
Data type	Numeric
Allowable codes	0 – 87<br>88 = N/A<br>99 = Unknown
Blanks	Blank if #12a, NPINF, ≠ 1 (Yes)

Question number	124b
Data element name	**NPINF4B**
Version	11
NP form question	Largest Infarct — brainstem or cerebellum
Length of field	4
Column positions	355 – 358
Data type	Numeric
Allowable codes	0.0–20.0<br>88.8 = N/A<br>99.9 = Unknown/missing
Blanks	Blank if #12a, NPINF, ≠ 1 (Yes)<br>Blank if #124a, NPINF4A, = 0, 88, or 99

Question number	124d
Data element name	**NPINF4D**
Version	11
NP form question	Second largest infarct — brainstem or cerebellum
Length of field	4
Column positions	360 – 363
Data type	Numeric
Allowable codes	0.0–20.0<br>88.8 = N/A<br>99.9 = Unknown/missing
Blanks	Blank if #12a, NPINF, ≠ 1 (Yes)<br>Blank if #124a, NPINF4A, < 2, or = 88 or 99

Question number	124f
Data element name	**NPINF4F**
Version	11
NP form question	Third largest infarct — brainstem or cerebellum
Length of field	4
Column positions	365 – 368
Data type	Numeric
Allowable codes	0.0–20.0<br>88.8 = N/A<br>99.9 = Unknown/missing
Blanks	Blank if #12a, NPINF, ≠ 1 (Yes)<br>Blank if #124a, NPINF4A, < 3, or = 88 or 99

Question number	12b
Data element name	**NPHEMO**
Version	11
NP form question	Were single or multiple old hemorrhages observed grossly?
Length of field	1
Column positions	370 – 370
Data type	Numeric
Allowable codes	0 = No<br>1 = Yes<br>8 = Not assessed<br>9 = Missing/unknown
Skip	If #12b, NPHEMO, ≠ 1, then skip to #12c, NPOLD

Question number	12b1
Data element name	**NPHEMO1**
Version	11
NP form question	Subdural or epidural hemorrhage
Length of field	1
Column positions	372 – 372
Data type	Numeric
Allowable codes	0 = No<br>1 = Yes<br>8 = Not assessed<br>9 = Missing/unknown
Blanks	Blank if #12b, NPHEMO, ≠ 1 (Yes)

Question number	12b2
Data element name	**NPHEMO2**
Version	11
NP form question	Primary parenchymal hemorrhage
Length of field	1
Column positions	374 – 374
Data type	Numeric
Allowable codes	0 = No<br>1 = Yes<br>8 = Not assessed<br>9 = Missing/unknown
Blanks	Blank if #12b, NPHEMO, ≠ 1 (Yes)

Question number	12b3
Data element name	**NPHEMO3**
Version	11
NP form question	Secondary parenchymal hemorrhage (e.g., tumor, vascular malformation)
Length of field	1
Column positions	376 – 376
Data type	Numeric
Allowable codes	0 = No<br>1 = Yes<br>8 = Not assessed<br>9 = Missing/unknown
Blanks	Blank if #12b, NPHEMO, ≠ 1 (Yes)

Question number	12c
Data element name	**NPOLD**
Version	11
NP form question	Old microinfarcts, not observed grossly
Length of field	1
Column positions	378 – 378
Data type	Numeric
Allowable codes	0 = No<br>1 = Yes<br>8 = Not assessed<br>9 = Missing/unknown
Skip	If #12c, NPOLD, ≠ 1, then skip to #12d, NPOLDD

Question number	12c1
Data element name	**NPOLD1**
Version	11
NP form question	Number of old microinfarcts — cerebral cortex
Length of field	1
Column positions	380 – 380
Data type	Numeric
Allowable codes	0 = 0<br>1 = 1<br>2 = 2<br>3 = 3 or more<br>8 = Not assessed<br>9 = Missing/unknown
Blanks	Blank if #12c, NPOLD, ≠ 1 (Yes)

Question number	12c2
Data element name	**NPOLD2**
Version	11
NP form question	Number of old microinfarcts — subcortical and periventricular white matter
Length of field	1
Column positions	382 – 382
Data type	Numeric
Allowable codes	0 = 0<br>1 = 1<br>2 = 2<br>3 = 3 or more<br>8 = Not assessed<br>9 = Missing/unknown
Blanks	Blank if #12c, NPOLD, ≠ 1 (Yes)

Question number	12c3
Data element name	**NPOLD3**
Version	11
NP form question	Number of old microinfarcts — subcortical gray matter
Length of field	1
Column positions	384 – 384
Data type	Numeric
Allowable codes	0 = 0<br>1 = 1<br>2 = 2<br>3 = 3 or more<br>8 = Not assessed<br>9 = Missing/unknown
Blanks	Blank if #12c, NPOLD, ≠ 1 (Yes)

Question number	12c4
Data element name	**NPOLD4**
Version	11
NP form question	Number of old microinfarcts — brainstem and cerebellum
Length of field	1
Column positions	386 – 386
Data type	Numeric
Allowable codes	0 = 0<br>1 = 1<br>2 = 2<br>3 = 3 or more<br>8 = Not assessed<br>9 = Missing/unknown
Blanks	Blank if #12c, NPOLD, ≠ 1 (Yes)

Question number	12d
Data element name	**NPOLDD**
Version	11
NP form question	Old cerebral microbleeds
Length of field	1
Column positions	388 – 388
Data type	Numeric
Allowable codes	0 = No<br>1 = Yes<br>8 = Not assessed<br>9 = Missing/unknown
Skip	If 12d, NPOLDD, ≠ 1, then skip to #12e, NPARTER

Question number	12d1
Data element name	**NPOLDD1**
Version	11
NP form question	Number of microbleeds — cerebral cortex
Length of field	1
Column positions	390 – 390
Data type	Numeric
Allowable codes	0 = 0<br>1 = 1<br>2 = 2<br>3 = 3 or more<br>8 = Not assessed<br>9 = Missing/unknown
Blanks	Blank if #12d, NPOLDD, ≠ 1 (Yes)

Question number	12d2
Data element name	**NPOLDD2**
Version	11
NP form question	Number of microbleeds — subcortical and periventricular white matter
Length of field	1
Column positions	392 – 392
Data type	Numeric
Allowable codes	0 = 0<br>1 = 1<br>2 = 2<br>3 = 3 or more<br>8 = Not assessed<br>9 = Missing/unknown
Blanks	Blank if #12d, NPOLDD, ≠ 1 (Yes)

Question number	12d3
Data element name	**NPOLDD3**
Version	11
NP form question	Number of microbleeds — subcortical gray matter
Length of field	1
Column positions	394 – 394
Data type	Numeric
Allowable codes	0 = 0<br>1 = 1<br>2 = 2<br>3 = 3 or more<br>8 = Not assessed<br>9 = Missing/unknown
Blanks	Blank if #12d, NPOLDD, ≠ 1 (Yes)

Question number	12d4
Data element name	**NPOLDD4**
Version	11
NP form question	Number of microbleeds — brainstem and cerebellum
Length of field	1
Column positions	396 – 396
Data type	Numeric
Allowable codes	0 = 0<br>1 = 1<br>2 = 2<br>3 = 3 or more<br>8 = Not assessed<br>9 = Missing/unknown
Blanks	Blank if #12d, NPOLDD, ≠ 1 (Yes)

Question number	12e
Data element name	**NPARTER**
Version	11
NP form question	Arteriolosclerosis
Length of field	1
Column positions	398 – 398
Data type	Numeric
Allowable codes	0 = None<br>1 = Mild<br>2 = Moderate<br>3 = Severe<br>8 = Not assessed<br>9 = Missing/unknown

Question number	12f
Data element name	**NPWMR**
Version	11
NP form question	White matter rarefaction
Length of field	1
Column positions	400 – 400
Data type	Numeric
Allowable codes	0 = None<br>1 = Mild<br>2 = Moderate<br>3 = Severe<br>8 = Not assessed<br>9 = Missing/unknown

Question number	12g
Data element name	**NPPATH**
Version	11
NP form question	Other pathologic changes related to ischemic or vascular disease not previously specified
Length of field	1
Column positions	402 – 402
Data type	Numeric
Allowable codes	0 = No<br>1 = Yes<br>8 = Not assessed<br>9 = Missing/unknown
Skip	If #12g, NPPATH, ≠ 1, then skip to #13, NPLBOD

Question number	12g1
Data element name	**NPNEC**
Version	11
NP form question	Laminar necrosis
Length of field	1
Column positions	404 – 404
Data type	Numeric
Allowable codes	0 = No<br>1 = Yes<br>8 = Not assessed<br>9 = Missing/unknown
Blanks	Blank if #12g, NPPATH, ≠ 1 (Yes)

Question number	12g2
Data element name	**NPPATH2**
Version	11
NP form question	Acute neuronal necrosis
Length of field	1
Column positions	406 – 406
Data type	Numeric
Allowable codes	0 = No<br>1 = Yes<br>8 = Not assessed<br>9 = Missing/unknown
Blanks	Blank if #12g, NPPATH, ≠ 1 (Yes)

Question number	12g3
Data element name	**NPPATH3**
Version	11
NP form question	Acute/subacute gross infarcts
Length of field	1
Column positions	408 – 408
Data type	Numeric
Allowable codes	0 = No<br>1 = Yes<br>8 = Not assessed<br>9 = Missing/unknown
Blanks	Blank if #12g, NPPATH, ≠ 1 (Yes)

Question number	12g4
Data element name	**NPPATH4**
Version	11
NP form question	Acute/subacute microinfarcts
Length of field	1
Column positions	410 – 410
Data type	Numeric
Allowable codes	0 = No<br>1 = Yes<br>8 = Not assessed<br>9 = Missing/unknown
Blanks	Blank if #12g, NPPATH, ≠ 1 (Yes)

Question number	12g5
Data element name	**NPPATH5**
Version	11
NP form question	Acute/subacute gross hemorrhage
Length of field	1
Column positions	412 – 412
Data type	Numeric
Allowable codes	0 = No<br>1 = Yes<br>8 = Not assessed<br>9 = Missing/unknown
Blanks	Blank if #12g, NPPATH, ≠ 1 (Yes)

Question number	12g6
Data element name	**NPPATH6**
Version	11
NP form question	Acute/subacute microhemorrhage
Length of field	1
Column positions	414 – 414
Data type	Numeric
Allowable codes	0 = No<br>1 = Yes<br>8 = Not assessed<br>9 = Missing/unknown
Blanks	Blank if #12g, NPPATH, ≠ 1 (Yes)

Question number	12g7
Data element name	**NPPATH7**
Version	11
NP form question	Vascular malformation of any type
Length of field	1
Column positions	416 – 416
Data type	Numeric
Allowable codes	0 = No<br>1 = Yes<br>8 = Not assessed<br>9 = Missing/unknown
Blanks	Blank if #12g, NPPATH, ≠ 1 (Yes)

Question number	12g8
Data element name	**NPPATH8**
Version	11
NP form question	Aneurysm of any type
Length of field	1
Column positions	418 – 418
Data type	Numeric
Allowable codes	0 = No<br>1 = Yes<br>8 = Not assessed<br>9 = Missing/unknown
Blanks	Blank if #12g, NPPATH, ≠ 1 (Yes)

Question number	12g9
Data element name	**NPPATH9**
Version	11
NP form question	Vasculitis of any type
Length of field	1
Column positions	420 – 420
Data type	Numeric
Allowable codes	0 = No<br>1 = Yes<br>8 = Not assessed<br>9 = Missing/unknown
Blanks	Blank if #12g, NPPATH, ≠ 1 (Yes)

Question number	12g10
Data element name	**NPPATH10**
Version	11
NP form question	CADASIL
Length of field	1
Column positions	422 – 422
Data type	Numeric
Allowable codes	0 = No<br>1 = Yes<br>8 = Not assessed<br>9 = Missing/unknown
Blanks	Blank if #12g, NPPATH, ≠ 1 (Yes)

Question number	12g11
Data element name	**NPPATH11**
Version	11
NP form question	Mineralization of blood vessels
Length of field	1
Column positions	424 – 424
Data type	Numeric
Allowable codes	0 = No<br>1 = Yes<br>8 = Not assessed<br>9 = Missing/unknown
Blanks	Blank if #12g, NPPATH, ≠ 1 (Yes)

Question number	12g12
Data element name	**NPPATHO**
Version	11
NP form question	Other ischemic or vascular pathology
Length of field	1
Column positions	426 – 426
Data type	Numeric
Allowable codes	0 = No<br>1 = Yes
Blanks	Blank if #12g, NPPATH, ≠ 1 (Yes)

Question number	12gx
Data element name	**NPPATHOX**
Version	11
NP form question	Other ischemic or vascular pathology specify
Length of field	30
Column positions	428 – 457
Data type	Character
Allowable codes	Any text or numbers except for single quotes, double quotes, ampersands, or percentage signs
Blanks	Blank if #12g, NPPATH, ≠ 1 (Yes)<br>Blank if #12g12, NPPATHO, ≠ 1 (Yes)

Question number	13
Data element name	**NPLBOD**
Version	11
NP form question	Is there evidence of Lewy body pathology
Length of field	1
Column positions	459 – 459
Data type	Numeric
Allowable codes	0 = No<br>1 = Brainstem predominant<br>2 = Limbic (transitional)<br>3 = Neocortical (diffuse)<br>4 = Amygdala predominant<br>5 = Olfactory bulb<br>8 = Not assessed<br>9 = Missing/unknown

Question number	14
Data element name	**NPNLOSS**
Version	11
NP form question	Neuron loss in the substantia nigra
Length of field	1
Column positions	461 – 461
Data type	Numeric
Allowable codes	0 = None<br>1 = Mild<br>2 = Moderate<br>3 = Severe<br>8 = Not assessed<br>9 = Missing/unknown

Question number	15
Data element name	**NPHIPSCL**
Version	11
NP form question	Hippocampal sclerosis (CA1 and/or subiculum)
Length of field	1
Column positions	463 – 463
Data type	Numeric
Allowable codes	0 = None<br>1 = Unilateral<br>2 = Bilateral<br>3 = Present but laterality not assessed<br>8 = Not assessed<br>9 = Missing/unknown

Question number	16a
Data element name	**NPTDPA**
Version	11
NP form question	Distribution of TDP-43 immunoreactive inclusions — spinal cord
Length of field	1
Column positions	465 – 465
Data type	Numeric
Allowable codes	0 = No<br>1 = Yes<br>8 = Not assessed<br>9 = Missing/unknown

Question number	16b
Data element name	**NPTDPB**
Version	11
NP form question	Distribution of TDP-43 immunoreactive inclusions — amygdala
Length of field	1
Column positions	467 – 467
Data type	Numeric
Allowable codes	0 = No<br>1 = Yes<br>8 = Not assessed<br>9 = Missing/unknown

Question number	16c
Data element name	**NPTDPC**
Version	11
NP form question	Distribution of TDP-43 immunoreactive inclusions — hippocampus
Length of field	1
Column positions	469 – 469
Data type	Numeric
Allowable codes	0 = No<br>1 = Yes<br>8 = Not assessed<br>9 = Missing/unknown

Question number	16d
Data element name	**NPTDPD**
Version	11
NP form question	Distribution of TDP-43 immunoreactive inclusions — entorhinal/inferior temporal cortex
Length of field	1
Column positions	471 – 471
Data type	Numeric
Allowable codes	0 = No<br>1 = Yes<br>8 = Not assessed<br>9 = Missing/unknown

Question number	16e
Data element name	**NPTDPE**
Version	11
NP form question	Distribution of TDP-43 immunoreactive inclusions — neocortex
Length of field	1
Column positions	473 – 473
Data type	Numeric
Allowable codes	0 = No<br>1 = Yes<br>8 = Not assessed<br>9 = Missing/unknown

Question number	17a
Data element name	**NPFTDTAU**
Version	11
NP form question	FTLD with tau pathology (FTLD-tau) or other tauopathy
Length of field	1
Column positions	475 – 475
Data type	Numeric
Allowable codes	0 = No<br>1 = Yes<br>8 = Not assessed<br>9 = Missing/unknown
Skip	If #17a, NPFTDTAU, ≠ 1, then skip to #17c, NPFTDTDP

Question number	17b1
Data element name	**NPPICK**
Version	11
NP form question	FTLD-tau subtype — FTLD-tau Pick's (PiD)
Length of field	1
Column positions	477–477
Data type	Numeric
Allowable codes	0 = No<br>1 = Yes<br>8 = Not assessed<br>9 = Missing/unknown
Blanks	Blank if #17a, NPFTDTAU, ≠ 1 (Yes)

Question number	17b2
Data element name	**NPFTDT2**
Version	11
NP form question	FTLD-tau subtype — other 3R tauopathy (includes *MAPT* mutation tauopathy)
Length of field	1
Column positions	479–479
Data type	Numeric
Allowable codes	0 = No<br>1 = Yes<br>8 = Not assessed<br>9 = Missing/unknown
Blanks	Blank if #17a, NPFTDTAU, ≠ 1 (Yes)

Question number	17b3
Data element name	**NPCORT**
Version	11
NP form question	FTLD-tau subtype — FTLD-tau corticobasal degeneration (CBD)
Length of field	1
Column positions	481–481
Data type	Numeric
Allowable codes	0 = No<br>1 = Yes<br>8 = Not assessed<br>9 = Missing/unknown
Blanks	Blank if #17a, NPFTDTAU, ≠ 1 (Yes)

Question number	17b4
Data element name	**NPPROG**
Version	11
NP form question	FTLD-tau subtype — FTLD-tau progressive supranuclear palsy (PSP)
Length of field	1
Column positions	483 – 483
Data type	Numeric
Allowable codes	0 = No<br>1 = Yes<br>8 = Not assessed<br>9 = Missing/unknown
Blanks	Blank if #17a, NPFTDTAU ≠ 1 (Yes)

Question number	17b5
Data element name	**NPFTDT5**
Version	11
NP form question	FTLD-tau subtype — argyrophilic grains
Length of field	1
Column positions	485 – 485
Data type	Numeric
Allowable codes	0 = No<br>1 = Yes<br>8 = Not assessed<br>9 = Missing/unknown
Blanks	Blank if #17a, NPFTDTAU, ≠ 1 (Yes)

Question number	17b6
Data element name	**NPFTDT6**
Version	11
NP form question	FTLD-tau subtype — other 4R tauopathy (includes sporadic multiple systems tauopathy, globular glial tauopathy, *MAPT* mutation tauopathy)
Length of field	1
Column positions	487– 487
Data type	Numeric
Allowable codes	0 = No<br>1 = Yes<br>8 = Not assessed<br>9 = Missing/unknown
Blanks	Blank if #17a, NPFTDTAU, ≠ 1 (Yes)

Question number	17b7
Data element name	**NPFTDT7**
Version	11
NP form question	FTLD-tau subtype — chronic traumatic encephalopathy
Length of field	1
Column positions	489 – 489
Data type	Numeric
Allowable codes	0 = No<br>1 = Yes<br>8 = Not assessed<br>9 = Missing/unknown
Blanks	Blank if #17a, NPFTDTAU, ≠ 1 (Yes)

Question number	17b8
Data element name	**NPFTDT8**
Version	11
NP form question	FTLD-tau subtype — amyotrophic lateral sclerosis (ALS)/Parkinsonism-dementia Complex of Guam
Length of field	1
Column positions	491 – 491
Data type	Numeric
Allowable codes	0 = No<br>1 = Yes<br>8 = Not assessed<br>9 = Missing/unknown
Blanks	Blank if #17a, NPFTDTAU, ≠ 1 (Yes)

Question number	17b9
Data element name	**NPFTDT9**
Version	11
NP form question	FTLD-tau subtype — tangle dominant disease
Length of field	1
Column positions	493 – 493
Data type	Numeric
Allowable codes	0 = No<br>1 = Yes<br>8 = Not assessed<br>9 = Missing/unknown
Blanks	Blank if #17a, NPFTDTAU, ≠ 1 (Yes)

Question number	17b10
Data element name	**NPFTDT10**
Version	11
NP form question	FTLD-tau subtype — other 3R + 4R tauopathy (includes unclassifiable, focal, glial only, *MAPT* mutation tauopathy, NOS)
Length of field	1
Column positions	495 – 495
Data type	Numeric
Allowable codes	0 = No<br>1 = Yes<br>8 = Not assessed<br>9 = Missing/unknown
Blanks	Blank if #17a, NPFTDTAU, ≠ 1 (Yes)

Question number	17c
Data element name	**NPFTDTDP**
Version	11
NP form question	FTLD with TDP-43 pathology (FTLD-TDP)
Length of field	1
Column positions	497 – 497
Data type	Numeric
Allowable codes	0 = No<br>1 = Yes<br>8 = Not assessed<br>9 = Missing/unknown

Question number	17d
Data element name	**NPALSMND**
Version	11
NP form question	ALS/motor neuron disease (MND) present
Length of field	1
Column positions	499 – 499
Data type	Numeric
Allowable codes	0 = No<br>1 = Yes, with TDP-43 inclusions in motor neurons<br>2 = Yes, with FUS inclusions in motor neurons<br>3 = Yes, with SOD1 inclusions in motor neurons<br>4 = Yes, with other inclusions<br>5 = Yes, with no specific inclusions<br>8 = Not assessed<br>9 = Missing/unknown

Question number	17e
Data element name	**NPOFTD**
Version	11
NP form question	Other FTLD
Length of field	1
Column positions	501 – 501
Data type	Numeric
Allowable codes	0 = No<br>1 = Yes<br>8 = Not assessed<br>9 = Missing/unknown
Skip	If #17e, FPOFTD, ≠ 1, then skip to #18a, NPARTAG

Question number	17f1
Data element name	**NPOFTD1**
Version	11
NP form question	Other FTLD subtype — atypical FTLD-U (aFTLD-U)
Length of field	1
Column positions	503 – 503
Data type	Numeric
Allowable codes	0 = No<br>1 = Yes<br>8 = Not assessed<br>9 = Missing/unknown
Blanks	Blank if #17e, NPOFTD, ≠ 1 (Yes)

Question number	17f2
Data element name	**NPOFTD2**
Version	11
NP form question	Other FTLD subtype — NIFID (neuronal intermediate filament inclusions disease)
Length of field	1
Column positions	505 – 505
Data type	Numeric
Allowable codes	0 = No<br>1 = Yes<br>8 = Not assessed<br>9 = Missing/unknown
Blanks	Blank if #17e, NPOFTD, ≠ 1 (Yes)

Question number	17f3
Data element name	**NPOFTD3**
Version	11
NP form question	Other FTLD subtype — BIBD (basophilic inclusion body disease)
Length of field	1
Column positions	507–507
Data type	Numeric
Allowable codes	0 = No<br>1 = Yes<br>8 = Not assessed<br>9 = Missing/unknown
Blanks	Blank if #17e, NPOFTD, ≠ 1 (Yes)

Question number	17f4
Data element name	**NPOFTD4**
Version	11
NP form question	Other FTLD subtype — FTLD-UPS (ubiquitin-proteasome system [ubiquitin or p62 positive, tau/TDP-43/FUS negative inclusions])
Length of field	1
Column positions	509–509
Data type	Numeric
Allowable codes	0 = No<br>1 = Yes<br>8 = Not assessed<br>9 = Missing/unknown
Blanks	Blank if #17e, NPOFTD, ≠ 1 (Yes)

Question number	17f5
Data element name	**NPOFTD5**
Version	11
NP form question	Other FTLD subtype — FTLD-NOS (includes dementia lacking distinctive histology (DLDH) and FTLD with no inclusions (FTLD-NI) detected by tau, TDP-43, or ubiquitin/p62 IHC)
Length of field	1
Column positions	511–511
Data type	Numeric
Allowable codes	0 = No<br>1 = Yes<br>8 = Not assessed<br>9 = Missing/unknown
Blanks	Blank if #17e, NPOFTD, ≠ 1 (Yes)

Question number	18a
Data element name	**NPARTAG**
Version	11
NP form question	Is ARTAG pathology present?
Length of field	1
Column positions	513-513
Data type	Numeric
Allowable codes	0 = No<br>1 = Yes<br>8 = Not assessed<br>9 = Missing/unknown
Skip	If #18a, NPARTAG, ≠ 1 (Yes), skip to 19a NPPDXA

Question number	18b
Data element name	**NPATGSEV**
Version	11
NP form question	Overall severity of ARTAG pathology
Length of field	1
Column positions	515-515
Data type	Numeric
Allowable codes	0 = None<br>1 = Mild<br>2 = Moderate<br>3 = Severe<br>8 = Not assessed
Blanks	Blank if #18a, NPARTAG ≠ 1 (Yes)

Question number	18c
Data element name	**NPATGAMY**
Version	11
NP form question	Is ARTAG pathology present in the AMYGDALA?
Length of field	1
Column positions	517-517
Data type	Numeric
Allowable codes	0 = No<br>1 = Yes<br>8 = Not assessed<br>9 = Missing/unknown
Skip	If #18c, NPATGAMY, ≠ 1 (Yes), skip to 18e NPATGFRN
Blanks	Blank if #18a, NPARTAG ≠ 1 (Yes)

Question number	18d1
Data element name	**NPATGAM1**
Version	11
NP form question	Localization of ARTAG pathology in the amygdala: Subpial
Length of field	1
Column positions	519-519
Data type	Numeric
Allowable codes	0 = None<br>1 = Focal<br>2 = Widespread<br>8 = Not assessed<br>9 = Missing/unknown
Blanks	Blank if #18a, NPARTAG ≠ 1 (Yes) or #18c, NPATGAMY ≠ 1 (Yes)

Question number	18d2
Data element name	**NPATGAM2**
Version	11
NP form question	Localization of ARTAG pathology in the amygdala: Subependymal
Length of field	1
Column positions	521-521
Data type	Numeric
Allowable codes	0 = None<br>1 = Focal<br>2 = Widespread<br>8 = Not assessed<br>9 = Missing/unknown
Blanks	Blank if #18a, NPARTAG ≠ 1 (Yes) or #18c, NPATGAMY ≠ 1 (Yes)

Question number	18d3
Data element name	**NPATGAM3**
Version	11
NP form question	Localization of ARTAG pathology in the amygdala: Gray matter
Length of field	1
Column positions	523-523
Data type	Numeric
Allowable codes	0 = None<br>1 = Focal<br>2 = Widespread<br>8 = Not assessed<br>9 = Missing/unknown
Blanks	Blank if #18a, NPARTAG ≠ 1 (Yes) or #18c, NPATGAMY ≠ 1 (Yes)

Question number	18d4
Data element name	**NPATGAM4**
Version	11
NP form question	Localization of ARTAG pathology in the amygdala: White matter
Length of field	1
Column positions	525-525
Data type	Numeric
Allowable codes	0 = None<br>1 = Focal<br>2 = Widespread<br>8 = Not assessed<br>9 = Missing/unknown
Blanks	Blank if #18a, NPARTAG ≠ 1 (Yes) or #18c, NPATGAMY ≠ 1 (Yes)

Question number	18d5
Data element name	**NPATGAM5**
Version	11
NP form question	Localization of ARTAG pathology in the amygdala: Perivascular
Length of field	1
Column positions	527-527
Data type	Numeric
Allowable codes	0 = None<br>1 = Focal<br>2 = Widespread<br>8 = Not assessed<br>9 = Missing/unknown
Blanks	Blank if #18a, NPARTAG ≠ 1 (Yes) or #18c, NPATGAMY ≠ 1 (Yes)

Question number	18e
Data element name	**NPATGFRN**
Version	11
NP form question	Is ARTAG pathology present in the FRONTAL NEOCORTEX?
Length of field	1
Column positions	529-529
Data type	Numeric
Allowable codes	0 = No<br>1 = Yes<br>8 = Not assessed<br>9 = Missing/unknown
Skip	If #18e, NPATGFRN, ≠ 1 (Yes), skip to 19a NPPDXA
Blanks	Blank if #18a, NPARTAG ≠ 1 (Yes) or #18c, NPATGAMY ≠ 1 (Yes)

Question number	18f1
Data element name	**NPATGFR1**
Version	11
NP form question	Localization of ARTAG pathology in the frontal neocortex: Subpial
Length of field	1
Column positions	531-531
Data type	Numeric
Allowable codes	0 = None<br>1 = Focal<br>2 = Widespread<br>8 = Not assessed<br>9 = Missing/unknown
Blanks	Blank if #18a, NPARTAG ne 1 (Yes) or #18e, NPATGFRN ne 1 (Yes)

Question number	18f2
Data element name	**NPATGFR2**
Version	11
NP form question	Localization of ARTAG pathology in the frontal neocortex: Gray matter
Length of field	1
Column positions	533-533
Data type	Numeric
Allowable codes	0 = None<br>1 = Focal<br>2 = Widespread<br>8 = Not assessed<br>9 = Missing/unknown
Blanks	Blank if #18a, NPARTAG ne 1 (Yes) or #18e, NPATGFRN ne 1 (Yes)

Question number	18f3
Data element name	**NPATGFR3**
Version	11
NP form question	Localization of ARTAG pathology in the frontal neocortex: White matter
Length of field	1
Column positions	535-535
Data type	Numeric
Allowable codes	0 = None<br>1 = Focal<br>2 = Widespread<br>8 = Not assessed<br>9 = Missing/unknown
Blanks	Blank if #18a, NPARTAG ne 1 (Yes) or #18e, NPATGFRN ne 1 (Yes)

Question number	18f4
Data element name	**NPATGFR4**
Version	11
NP form question	Localization of ARTAG pathology in the frontal neocortex: Perivascular
Length of field	1
Column positions	537-537
Data type	Numeric
Allowable codes	0 = None<br>1 = Focal<br>2 = Widespread<br>8 = Not assessed<br>9 = Missing/unknown
Blanks	Blank if #18a, NPARTAG ne 1 (Yes) or #18e, NPATGFRN ne 1 (Yes)

Question number	19a
Data element name	**NPPDXA**
Version	11
NP form question	Pigment-spheroid degeneration/NBIA
Length of field	1
Column positions	539-539
Data type	Numeric
Allowable codes	0 = No<br>1 = Yes<br>8 = Not assessed<br>9 = Missing/unknown

Question number	19b
Data element name	**NPPDXB**
Version	11
NP form question	Multiple system atrophy
Length of field	1
Column positions	541-541
Data type	Numeric
Allowable codes	0 = No<br>1 = Yes<br>8 = Not assessed<br>9 = Missing/unknown

Question number	19c
Data element name	**NPPDXC**
Version	11
NP form question	Prion disease
Length of field	1
Column positions	543-543
Data type	Numeric
Allowable codes	0 = No<br>1 = Yes<br>8 = Not assessed<br>9 = Missing/unknown

Question number	19d
Data element name	**NPPDXD**
Version	11
NP form question	Trinucleotide disease (Huntington disease, SCA, other)
Length of field	1
Column positions	545-545
Data type	Numeric
Allowable codes	0 = No<br>1 = Yes<br>8 = Not assessed<br>9 = Missing/unknown

Question number	19e
Data element name	**NPPDXE**
Version	11
NP form question	Malformation of cortical development
Length of field	1
Column positions	547-547
Data type	Numeric
Allowable codes	0 = No<br>1 = Yes<br>8 = Not assessed<br>9 = Missing/unknown

Question number	19f
Data element name	**NPPDXF**
Version	11
NP form question	Metabolic/storage disorder of any type
Length of field	1
Column positions	549-549
Data type	Numeric
Allowable codes	0 = No<br>1 = Yes<br>8 = Not assessed<br>9 = Missing/unknown

Question number	19g
Data element name	**NPPDXG**
Version	11
NP form question	WM disease, leukodystrophy
Length of field	1
Column positions	551-551
Data type	Numeric
Allowable codes	0 = No<br>1 = Yes<br>8 = Not assessed<br>9 = Missing/unknown

Question number	19h
Data element name	**NPPDXH**
Version	11
NP form question	WM disease, multiple sclerosis or other demyelinating disease
Length of field	1
Column positions	553-553
Data type	Numeric
Allowable codes	0 = No<br>1 = Yes<br>8 = Not assessed<br>9 = Missing/unknown

Question number	19i
Data element name	**NPPDXI**
Version	11
NP form question	Contusion/traumatic brain injury of any type, acute
Length of field	1
Column positions	555-555
Data type	Numeric
Allowable codes	0 = No<br>1 = Yes<br>8 = Not assessed<br>9 = Missing/unknown

Question number	19j
Data element name	**NPPDXJ**
Version	11
NP form question	Contusion/traumatic brain injury of any type, Chronic
Length of field	1
Column positions	557-557
Data type	Numeric
Allowable codes	0 = No<br>1 = Yes<br>8 = Not assessed<br>9 = Missing/unknown

Question number	19k
Data element name	**NPPDXK**
Version	11
NP form question	Neoplasm, primary
Length of field	1
Column positions	559-559
Data type	Numeric
Allowable codes	0 = No<br>1 = Yes<br>8 = Not assessed<br>9 = Missing/unknown

Question number	19l
Data element name	**NPPDXL**
Version	11
NP form question	Neoplasm, metastatic
Length of field	1
Column positions	561-561
Data type	Numeric
Allowable codes	0 = No<br>1 = Yes<br>8 = Not assessed<br>9 = Missing/unknown

Question number	19m
Data element name	**NPPDXM**
Version	11
NP form question	Infectious process of any type (encephalitis, abscess, etc.)
Length of field	1
Column positions	563-563
Data type	Numeric
Allowable codes	0 = No<br>1 = Yes<br>8 = Not assessed<br>9 = Missing/unknown

Question number	19n
Data element name	**NPPDXN**
Version	11
NP form question	Herniation, any site
Length of field	1
Column positions	565-565
Data type	Numeric
Allowable codes	0 = No<br>1 = Yes<br>8 = Not assessed<br>9 = Missing/unknown

Question number	19o
Data element name	**NPPDXO**
Version	11
NP form question	Trisomy 21/Down syndrome
Length of field	1
Column positions	567-567
Data type	Numeric
Allowable codes	0 = No<br>1 = Yes<br>8 = Not assessed<br>9 = Missing/unknown

Question number	19p
Data element name	**NPPDXP**
Version	11
NP form question	AD-related genes
Length of field	1
Column positions	569-569
Data type	Numeric
Allowable codes	0 = No<br>1 = Yes<br>8 = Not assessed<br>9 = Missing/unknown

Question number	19q
Data element name	**NPPDXQ**
Version	11
NP form question	FTLD-related genes
Length of field	1
Column positions	571-571
Data type	Numeric
Allowable codes	0 = No<br>1 = Yes<br>8 = Not assessed<br>9 = Missing/unknown

Question number	19r
Data element name	**NPPDXR**
Version	11
NP form question	First other pathologic diagnosis
Length of field	1
Column positions	573-573
Data type	Numeric
Allowable codes	0 = No<br>1 = Yes

Question number	19r1
Data element name	**NPPDXRX**
Version	11
NP form question	First other pathologic diagnosis specify
Length of field	30
Column positions	575-604
Data type	Character
Allowable codes	Any text or numbers except for single quotes, double quotes, ampersands, or percentage signs
Blanks	Blank if #19r, NPPDXR, = 0 (No)

Question number	19s
Data element name	**NPPDXS**
Version	11
NP form question	Second other pathologic diagnosis
Length of field	1
Column positions	606-606
Data type	Numeric
Allowable codes	0 = No<br>1 = Yes

Question number	19s1
Data element name	**NPPDXSX**
Version	11
NP form question	Second other pathologic diagnosis specify
Length of field	30
Column positions	608-637
Data type	Character
Allowable codes	Any text or numbers except for single quotes, double quotes, ampersands, or percentage signs
Blanks	Blank if #19s, NPPDXS, = 0 (No)

Question number	19t
Data element name	**NPPDXT**
Version	11
NP form question	Third other pathologic diagnosis
Length of field	1
Column positions	639-639
Data type	Numeric
Allowable codes	0 = No<br>1 = Yes

Question number	19t1
Data element name	**NPPDXTX**
Version	11
NP form question	Third other pathologic diagnosis specify
Length of field	30
Column positions	641-670
Data type	Character
Allowable codes	Any text or numbers except for single quotes, double quotes, ampersands, or percentage signs
Blanks	Blank if #19t, NPPDXT, = 0 (No)

Question number	20a
Data element name	**NPBNKA**
Version	11
NP form question	Banked frozen brain or half brain
Length of field	1
Column positions	672-672
Data type	Numeric
Allowable codes	0 = No<br>1 = Yes<br>9 = Missing/unknown

Question number	20b
Data element name	**NPBNKB**
Version	11
NP form question	Banked frozen wedge of cerebellum or other sample for future DNA prep
Length of field	1
Column positions	674-674
Data type	Numeric
Allowable codes	0 = No<br>1 = Yes<br>9 = Missing/unknown

Question number	20c
Data element name	**NPBNKC**
Version	11
NP form question	Formalin- or paraformaldehyde-fixed brain
Length of field	1
Column positions	676-676
Data type	Numeric
Allowable codes	0 = No<br>1 = Yes<br>9 = Missing/unknown

Question number	20d
Data element name	**NPBNKD**
Version	11
NP form question	Paraffin-embedded blocks of brain regions
Length of field	1
Column positions	678-678
Data type	Numeric
Allowable codes	0 = No<br>1 = Yes<br>9 = Missing/unknown

Question number	20e
Data element name	**NPBNKE**
Version	11
NP form question	Banked postmortem CSF
Length of field	1
Column positions	680-680
Data type	Numeric
Allowable codes	0 = No<br>1 = Yes<br>9 = Missing/unknown

Question number	20f
Data element name	**NPBNKF**
Version	11
NP form question	Banked postmortem blood or serum
Length of field	1
Column positions	682-682
Data type	Numeric
Allowable codes	0 = No<br>1 = Yes<br>9 = Missing/unknown

Question number	20g
Data element name	**NPBNKG**
Version	11
NP form question	Banked DNA
Length of field	1
Column positions	684-684
Data type	Numeric
Allowable codes	0 = No<br>1 = Yes<br>9 = Missing/unknown

Question number	20h
Data element name	**NPFAUT**
Version	11
NP form question	Full autopsy performed
Length of field	1
Column positions	686-686
Data type	Numeric
Allowable codes	0 = No<br>1 = Yes<br>9 = Missing/unknown

Question number	20h1
Data element name	**NPFAUT1**
Version	11
NP form question	If full autopsy, first major finding
Length of field	60
Column positions	688-747
Data type	Character
Allowable codes	Any text or numbers except for single quotes, double quotes, ampersands, or percentage signs
Blanks	Blank if #20h, NPFAUT, ≠ 1 (Yes)

Question number	20h2
Data element name	**NPFAUT2**
Version	11
NP form question	If full autopsy, second major finding
Length of field	60
Column positions	749-808
Data type	Character
Allowable codes	Any text or numbers except for single quotes, double quotes, ampersands, or percentage signs
Blanks	Blank if #20h, NPFAUT, ≠ 1 (Yes)

Question number	20h3
Data element name	**NPFAUT3**
Version	11
NP form question	If full autopsy, third major finding
Length of field	60
Column positions	810-869
Data type	Character
Allowable codes	Any text or numbers except for single quotes, double quotes, ampersands, or percentage signs
Blanks	Blank if #20h, NPFAUT, ≠ 1 (Yes)

Question number	20h4
Data element name	**NPFAUT4**
Version	11
NP form question	If full autopsy, fourth major finding
Length of field	60
Column positions	871-930
Data type	Character
Allowable codes	Any text or numbers except for single quotes, double quotes, ampersands, or percentage signs
Blanks	Blank if #20h, NPFAUT, ≠ 1 (Yes)

Question number	21
Data element name	**FORMVER**
Version	11
NP form question	Form version
Length of field	2
Column positions	932-933
Data type	Numeric
Allowable codes	11

Calculation of EYOs

Part I: Three Types of Age of Symptom Onset and the Master Database

(I) The Decline Age (or the mean of the decline ages)—Age of symptom onset for individuals who are already symptomatic: CDR global score (referred to as CDR hereafter) is used to define Symptomatic and Asymptomatic. At each visit, a CDR score will be determined by the clinician. CDR 0 is considered asymptomatic. While CDR 0.5 or above is considered symptomatic. At each visit, if a CDR of 0.5 or above is given by the clinician at study entry, the clinician will determine a *decline age* using standardized list of symptoms to explore the earliest age of symptom onset. This decline age is thereafter defined as age of symptom onset. In very rare cases that a CDR 0.5 or above is determined at a visit but the corresponding decline age is not provided, then the individual's age at the visit where the 1st CDR of 0.5 or above is given will be used as the decline age (age of symptom onset). The DIAN observational study is designed in a way such that, at each post-baseline visit, the clinician is blinded to an individuals' data collected in the previous visit/visits to increase the independence and accuracy of evaluation. That means at all the visits where a CDR of 0.5 or above is given, a decline age will also be provided. Therefore, multiple decline ages are typically available for individuals with multiple visits with CDRs of 0.5 or above. In these cases, the mean of these decline ages for each individual will be calculated and used as the age of symptom onset for that individual. Hereafter, we generally refer to this type of age of symptom onset as "**the mean of decline ages"**.

If an individual had a CDR of 0.5 at one visit and a CDR of 0 at the following visit, then the previous CDR 0.5 will be considered as false symptomatic signal and a decline age will not be established until there are consecutive scores of CDR >0 and no further CDR 0 scores.

(II) Mean Mutation Age of Symptom Onset: the mean of the onset ages for individuals with the same mutation. A master database has been built by the DIAN observational study team. This database includes almost 4000 unique persons with ADAD and known mutations. Of them, about 1500 had known age of onset, and these ages of onset are used to calculate the mean mutation age of onset.

(III) Parental or Proxy Age of Symptom Onset: Individual's parental age of onset. In the unlikely event that an individual has two parents (consanguinity) that carry the same mutation, then the parental age of onset will be the mean of both parents' onset ages. In the event a Parental age of onset cannot be determined, the closest relative with an age of onset will be used as a proxy for age of symptom onset.

The Master Database: The master database will be updated so that any recently discovered new mutations, additional individuals with established mutations, or updated ages of onset for those already in the database can be included. Once the database is updated, the mean mutation age of symptom onset or parental age of symptom onset will be re-calculated to increase accuracy. Although the parental age of onset typically does not require any calculation since in most cases only one parental age of onset is available. Subsequently, the estimated

years from symptom onset (EYO) will also be re-calculated. That means the EYOs of the same person may change due to the updating of the master database. Therefore, the same person's EYOs at the same visits, say, in Data Freeze 07, can be different from those in Data Freeze 13.

It is anticipated that the master database will be updated every 3-4 years. Before a new update, all the data freeze will use the same mean mutation age of symptom onset and thus leads to the same EYOs for the same visits of the same person.

Part II: Mutation/Parental EYO

Mutation/Parental EYO calculation (Names as dian_mutpar_eyo in DF17): This EYO is calculated based on (1) mean mutation age of symptom onset and (2) parental age of symptom onset in the following ordered steps (applied to both mutation carriers and non-carriers):

(i) At any visit, EYO equals to the visit age minus the mean mutation age of symptom onset (EYO=Visit age – Mean mutation age of symptom onset) if the individual's mutation is known and the mean mutation age of symptom onset for this individual's mutation is available in the master database.

(ii) If any given individual's mutation is not available in the master database (e.g. the mutation has not been previously reported or other member age of onset not available) then at any visit, EYO equals to the visit age minus the parental age of symptom onset (EYO=Visit age – parental age of symptom onset).

Every DIAN participant has at least one of these two pieces of information, thus every participant will have this type of EYO values.

Part III: DIAN EYO

DIAN EYO (named as DIAN_EYO in DF17): The DIAN EYO improves the accuracy of the EYO estimation by incorporating an individual's actual decline age in determining their EYO as opposed to just using the mean mutation or parental age of symptom onset.

DIAN EYO Calculation (applied to both mutation carriers and non-carriers)**:**

(i) For symptomatic individuals with reported decline ages, their DIAN EYO= Visit age – the mean of decline ages.

(ii) For asymptomatic individuals, their DIAN EYO = their Mutation/Parental EYO.

DIAN EYO is dynamic and will be updated for each data freeze to reflect individual's symptomatic status change during follow-up.
